# Synthesis of perhalogenated silylboranes (X = Cl, I) and their application in regiodivergent alkene silaboration

**DOI:** 10.1039/d5sc06234a

**Published:** 2025-09-10

**Authors:** Jan Heller, Christoph D. Buch, Alexander V. Virovets, Eugenia Peresypkina, Hans-Wolfram Lerner, Felipe Fantuzzi, Matthias Wagner

**Affiliations:** a Institut für Anorganische und Analytische Chemie, Goethe-Universität Frankfurt Max-von-Laue-Straße 7 D-60438 Frankfurt am Main Germany matthias.wagner@chemie.uni-frankfurt.de; b School of Chemistry and Forensic Science, University of Kent Park Wood Rd Canterbury CT2 7NH UK

## Abstract

Silaboration of olefins is a synthetically valuable and atom-economic mode of functionalization; however, it typically requires transition-metal catalysis. We have overcome this requirement by using highly reactive perhalogenated silylboranes, X_2_B–SiX_3_ (X = Cl, I), for which we herein report a straightforward synthesis, a full characterization, and their key properties. Access to this compound class was enabled by substantial improvement in the synthesis protocol for our previously published compound [Et_4_N][I_3_B–SiI_3_], now available on a 40 g scale *via* only two steps. Cation exchange with Li[Al(OC(CF_3_)_3_)_4_] affords the mixture Li[I_3_B–SiI_3_]/I_2_B–SiI_3_/LiI, serving as a synthetic equivalent of the elusive pure I_2_B–SiI_3_. Its chlorine analogue Cl_2_B–SiCl_3_ is accessible as a distillable liquid *via* treatment of [Et_4_N][I_3_B–SiI_3_] with GaCl_3_. For both perhalogenated silylboranes, various Lewis base adducts Do·X_2_B–SiX_3_ were obtained in excellent yields and structurally characterized by X-ray diffraction (Do = SMe_2_, Py, PPh_3_, IDipp; IDipp = 1,3-bis(2,6-diisopropylphenyl)-1,3-dihydro-2*H*-imidazol-2-ylidene). We demonstrated that Me_2_S·I_2_B–SiI_3_ undergoes efficient 1,2-silaboration of the challenging, non-activated substrate ethylene at rt with 0.1 eq. BI_3_ as promoter. In contrast, Li[I_3_B–SiI_3_]/I_2_B–SiI_3_/LiI effects a quantitative, unprecedented 1,1-silaboration of cyclohexene at rt. This remarkable reactivity switch was elucidated by experimental and quantum-chemical studies of the underlying steric and electronic factors.

## Abstract 2

Perhalogenated silylboranes X_2_B–SiX_3_ (X = Cl, I) were prepared in three steps on gram scale. They enable catalyst-free olefin silaboration, including unique 1,1-addition to cyclohexene, with broad functionalization potential.

## Graphical abstract

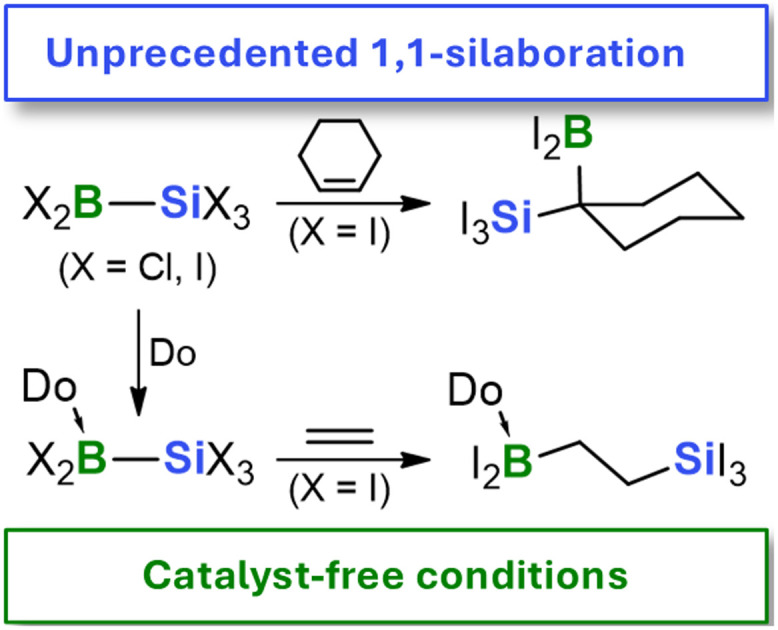

## Introduction

**Fig. 1:**
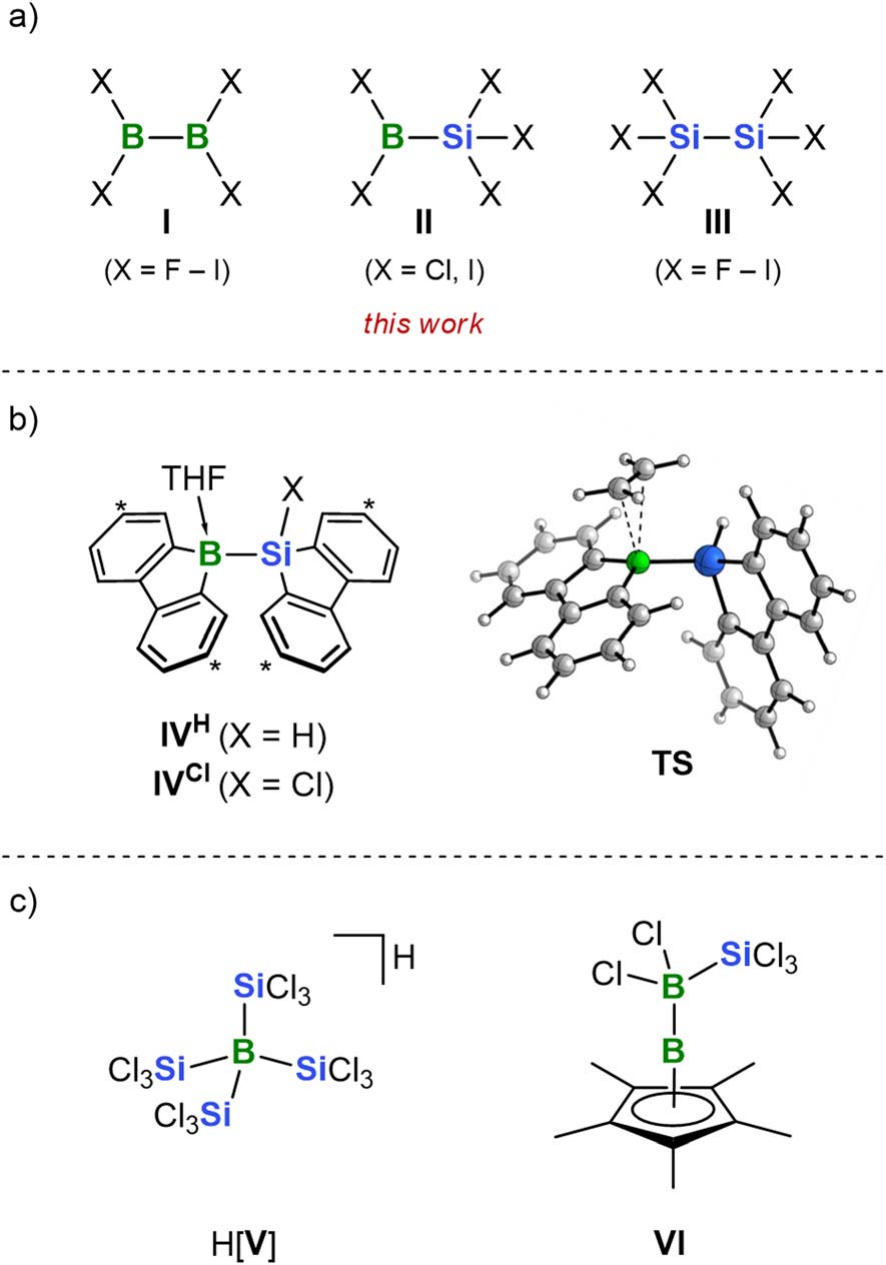
(a) Perhalogenated diboranes(4) (I), disilanes (III), and the perhalogenated silylboranes (II) studied in this work. (b) Previously studied silylboranes (IV) capable of undergoing uncatalyzed silaboration reactions (C atoms marked with asterisks bear *t*Bu substituents); computed transition state (TS) for the silaboration of ethylene with IV^H^ (*t*Bu groups have been omitted in the calculations). (c) Silylborates H[V] and VI bearing some structural similarity with II.

Once considered exotic and of limited utility, perhalogenated diborane(4) and disilane compounds (I, III; Fig. 1a) have recently emerged as valuable building blocks for purposes ranging from organic synthesis to materials development.^1–6^ The direct bond between two Lewis-acidic sites in I and III, each bearing good leaving groups, presents both challenges and opportunities: on the one hand, this unique combination promotes spontaneous disproportionation and vigorous decomposition upon exposure to air and moisture.^7–10^ On the other hand, it enables uncatalyzed diboration reactions using I,^11–17^ the *in situ* generation of versatile [SiX_3_]^−^ nucleophiles from III upon simple halide addition,^18,19^ and extensive late-stage derivatization at the B–X and Si–X bonds of the primary products. Thus, in contrast to the abundant bis(pinacolato)diboron (pinB–Bpin), whose B atoms are electronically tamed by O=B π-donation and serve primarily as transmetallation partners in Suzuki–Miyaura cross-couplings,^20–22^ type-I halogenoboranes are tailored for applications where the B atoms are to remain as property-determining functional units in the final molecule.^23–29^ Likewise, the Si_2_X_6_/X^−^ trichlorosilylation system and the controlled disproportionation of Si_2_X_6_ with NR_3_ (ref. 30–32) have proven valuable for the synthesis of extensively derivatizable organosilanes,^33–36^ oligotetrelanes,^37–42^ and silicon clusters.^43–50^ In contrast to I, III undergoes no spontaneous 1,2-additions to unsaturated organic substrates, and theoretical studies predict a prohibitively high activation barrier without a catalyst.^51,52^

Given the indispensable role of borylated^53,54^ and silylated^55^ building blocks in synthesis, it is desirable to combine both types of functional groups within a single building block, for which silylboranes of the type R_2_B–SiR_3_ are the most obvious candidates.^56–60^ Electronically stabilized representatives such as the prominent pinB–SiMe_2_Ph typically require activation by (precious) metal complexes prior to addition across C=C double^61–63^ or C≡C triple bonds.^64–72^ In only a few cases, the addition of a (Lewis) base (KO*t*Bu,^73,74^ KN(SiMe_3_)_2_,^75^ PR_3_,^76^ pyridines^77,78^) has been sufficient to replace the transition metal catalyst in silaboration reactions. Yet, a significant proportion of base-catalyzed silylborane transformations results in incorporation of either the boryl^74,79–84^ or the silyl^85–91^ group,^92^ while the respective counterpart is discarded. So far, a single uncatalyzed silaboration reaction has been reported, employing compounds IV^H^ and IV^Cl^ in THF (Fig. 1b).^93,94^ Key to this transformation is the incorporation of both the B and Si atoms of IV into planar heterofluorene scaffolds, which—compared to pinB–SiMe_2_Ph—enhances their exposure to the unsaturated substrate while reducing π-electron donation into the vacant B(p_z_) orbital (quantum-chemical calculations exclude a promoting effect of the THF ligand on B–Si-bond cleavage; *cf.* the transition state TS of olefin silaboration shown in Fig. 1b). Based on this background and the high reactivity of I and III, we reasoned that the perhalogenated silylborane II (Fig. 1a) as a silaboration reagent should uniquely combine a strong tendency towards B–Si heterolysis and diverse opportunities for subsequent derivatization. Herein, we demonstrate that type-II compounds with X = Cl, I can indeed be readily synthesized on a multigram scale. We provide a full characterization of their B-adducts with various Lewis bases and show that the Cl derivative Cl_2_B–SiCl_3_ can even be isolated in its free form as a distillable liquid. Notably, we disclose that both uncatalyzed 1,2- and rare 1,1-addition reactions to alkenes have been achieved. Only a few previously reported compounds share structural or electronic features with II. Among them are the borate H[V] and the *nido* cluster VI (Fig. 1c).^95,96^ Furthermore, the molecular structure of the anion [Cl_3_B–SiCl_3_]^−^ has been determined through single crystal X-ray structure analysis of the salt [(TMS_2_N)SiCl_2_–B(*η*^5^-C_5_Me_5_)][Cl_3_B–SiCl_3_] (TMS = Me_3_Si).^97^

## Results and discussion

The synthesis of B_2_X_4_ (I) dates back to 1925, but for decades remained the domain of specialists capable of mastering the technically challenging gas-phase protocols of the time.^98–100^ A major breakthrough came in 1981, when Nöth *et al.* obtained B_2_Br_4_ in good yields by converting B_2_(OMe)_4_ with BBr_3_ through a convenient solution-phase synthesis.^101^ In 2017, Braunschweig *et al.* extended this approach to the other perhalogenated diboranes(4) *via* solution-phase reactions of B_2_Br_4_ with SbF_3_, GaCl_3_, and BI_3_.^5^

Si_2_Cl_6_, a side product of several large-scale processes in the silicon industry,^102^ is commercially available; quantitative Cl/F exchange with SbF_3_ affords Si_2_F_6_.^103^ The perbromo- and periododisilanes are accessible from Si_2_Ph_6_ by Ph/X exchange with MeC(O)X/AlX_3_ (X = Br, I).^104^

**Scheme 1:**
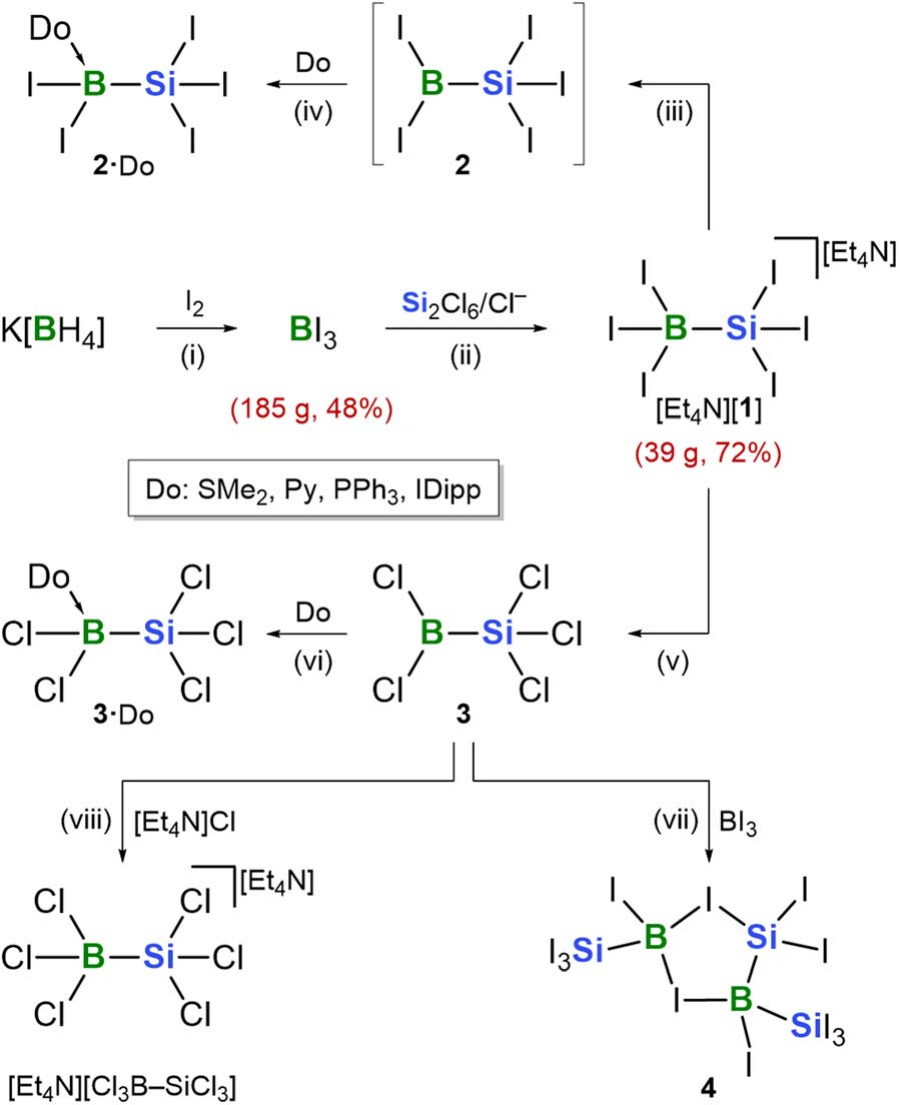
Optimized synthesis of [Et_4_N][1], enabling multigram-scale access to this key starting material. Conversion of the poorly soluble salt [Et_4_N][1] into moderately soluble neutral donor adducts 2·Do (Py: pyridine; IDipp: 1,3-bis(2,6-diisopropylphenyl)-1,3-dihydro-2*H*-imidazol-2-ylidene). Formation of the liquid perchlorinated silylborane 3 and its adducts 3·Do. Reaction of 3 with BI_3_ does not furnish 2, but yields the five-membered ring compound 4, characterized by X-ray crystallography. Reagents and conditions: (i) 1.25 eq. I_2_, *n*-heptane, 80 °C, min. 10 h, 48% yield; (ii) 1 eq. [Et_4_N]Cl, 0.5 eq. Si_2_Cl_6_, 0.05 eq. BI_3_, CH_2_Cl_2_, rt, 24 h, 72% yield; (iii) 1.1 eq. Li[Al(OC(CF_3_)_3_)_4_], *o*DFB, rt, 24 h; (iv) 1.1 eq. Do: SMe_2_, Py, PPh_3_, IDipp, CH_2_Cl_2_, rt, 24 h, SMe_2_ = 91%, Py = 83%, PPh_3_ = 87%, IDipp = 71% yield; (v) *Method A*: 2.1 eq. GaCl_3_, 80 °C, 1 h, 96% yield; *Method B*: 2.1 eq. GaCl_3_, *o*DFB, rt, 15 min; (vi) 1.0 eq. Do: SMe_2_, Py, PPh_3_, IDipp, *o*DFB, rt, 15 min, SMe_2_ = 94%, Py = 95%, PPh_3_ = 91%, IDipp = 89% yield; (vii) 2 eq. BI_3_, *o*DFB, rt, 15 min, 95% yield; (viii) 1 eq. [Et_4_N]Cl, *o*DFB, rt, 15 min, 92% yield.

Analogous to how B_2_Br_4_ and Si_2_Cl_6_ grant access to their respective compound classes, the salt [Et_4_N][I_3_B–SiI_3_] ([Et_4_N][1]; Scheme 1) serves as a key starting material for developing perhalogenated silylboranes. Several years ago, we first reported [Et_4_N][1], primarily to demonstrate the *in situ* formation of [SiCl_3_]^−^ as the reactive intermediate in the Si_2_Cl_6_/Cl^−^ trichlorosilylation system *via* Lewis-adduct formation with BX_3_.^19^ Our study revealed that (excess) BI_3_ is a more effective trapping reagent than BCl_3_, because it is the stronger Lewis acid and outcompetes coexisting Si_2_Cl_6_ for coordination with [SiCl_3_]^−^, thus suppressing the formation of unwanted oligosilane side products.^18^ By thoroughly optimizing the original protocol, the yield of [Et_4_N][1] was increased from ≈45% to ≈70%, and the synthesis was scaled to ≈40 g (Scheme 1). A key improvement is the addition of a second portion of BI_3_ (0.1 eq.) toward the end of the reaction, following the initial addition of 2 eq. BI_3_. This prevents contamination of [Et_4_N][1] with [Et_4_N][I_2_ClB–SiI_3_], previously described as an ‘unknown side product’; its identity has now been unequivocally confirmed by X-ray crystallography (Fig. S102). This finding laid the foundation for a systematic exploration of perhalogenated silylboranes.

### Syntheses of new compounds

The quantitative Si–Cl/Si–I exchange during the formation of [Et_4_N][1] is advantageous, as iodinated products crystallize more readily in pure form than their chlorinated congeners. However, in combination with the salt-like nature of [Et_4_N][1], this results in extremely low solubility, posing challenges for subsequent transformations. As initial derivatizations, we consequently replaced one I^−^ ligand in [Et_4_N][1] with neutral donor ligands (Do) bearing solubilizing substituents. To this end, suspensions of [Et_4_N][1] and Krossing's salt (Li[Al(OC(CF_3_)_3_)_4_])^105^ in CH_2_Cl_2_ were treated with the respective ligand at rt [Do: SMe_2_, pyridine (Py), PPh_3_, 1,3-bis(2,6-diisopropylphenyl)-1,3-dihydro-2*H*-imidazol-2-ylidene (IDipp); Scheme 1]. After filtration, colorless crystals of the corresponding adducts 2·Do readily grew from the filtrate (yields: SMe_2_ = 91%; Py = 83%; PPh_3_ = 87%; IDipp = 71%). In stark contrast to BI_3_, which is sensitive to sunlight, 2·Do exhibit remarkable photostability, with no signs of decomposition upon light exposure.

Cation-exchange using Krossing's salt precipitated LiI as an insoluble byproduct instead of releasing soluble [Et_4_N]I, thereby driving the quantitative I^−^/Do substitution and facilitating the isolation of pure 2·Do. Most of the second byproduct, [Et_4_N][Al(OC(CF_3_)_3_)_4_], remained in the mother liquor; residual traces adhering to the crystals of 2·Do were removed by rinsing with *ortho*-difluorobenzene (*o*DFB). To characterize the primary [Et_4_N]^+^/Li^+^ cation-exchange product, an equimolar mixture of [Et_4_N][1] and Li[Al(OC(CF_3_)_3_)_4_] was stirred in *o*DFB. The resulting solid, which proved insoluble in all common inert solvents, was analyzed by solid-state ^11^B and ^29^Si NMR spectroscopy. The data indicated the presence of Li[1] along with free 2 (and already eliminated LiI; see the SI for more details). Given that the insolubility of the free silylborane I_2_B–SiI_3_ (2) precluded its isolation and characterization in pure form, we next turned our attention to the synthesis of its perchlorinated congener Cl_2_B–SiCl_3_ (3; Scheme 1).

The targeted full I/Cl substitution was straightforwardly achieved by stirring [Et_4_N][1] with 2 eq. GaCl_3_—either as a solid mixture that gradually liquefied upon intermittent heating to 80 °C (*Method A*), or in *o*DFB (*Method B*). Neat 3 (*Method A*) or its calibrated *o*DFB solution (*Method B*) was obtained by gas-phase transfer of all volatiles under static vacuum at rt into a glass vessel cooled with liquid N_2_.^106^ The colorless donor adducts 3·Do were harvested in crystalline form after stirring 3 and Do in *o*DFB for 15 min at rt (Scheme 1; yields: SMe_2_ = 94%; Py = 95%; PPh_3_ = 91%; IDipp = 89%).

As noted above, the perchlorinated analogue [Et_4_N][Cl_3_B–SiCl_3_] of [Et_4_N][1] is not accessible through trapping of *in situ*-generated [SiCl_3_]^−^ with BCl_3_. With the free silylborane 3 in hand, we have now demonstrated that its reaction with [Et_4_N]Cl in *o*DFB affords [Et_4_N][Cl_3_B–SiCl_3_] in >90% yield (Scheme 1). This confirms that the BCl_3_-based trapping experiment has failed not due to an inherent instability of [Cl_3_B–SiCl_3_]^−^, but rather because of interfering side reactions that dominate in the mixture BCl_3_/Si_2_Cl_6_/[Et_4_N]Cl.

As a final approach, we attempted to access pure 2*via* Cl/I exchange at 3 using 2 eq. BI_3_ in *o*DFB. Instead of the target compound, we obtained the five-membered ring species 4 in good yields (Scheme 1). Its solid-state structure provides valuable insight to rationalize fundamental reactivity patterns of perhalogenated silylboranes (see below).

### NMR-spectroscopic, mass-spectrometric, and X-ray-crystallographic characterization of new compounds^107^

Liquid-phase NMR spectra were recorded at rt in CD_2_Cl_2_ and on a sample of neat 3.

The free perchlorinated silylborane 3 gives rise to a singlet at 63.7 ppm in the ^11^B NMR spectrum and to a 1 : 1 : 1 : 1 quartet at −8.2 ppm in the ^29^Si NMR spectrum (^1^*J*(B,Si) ≈ 200 Hz; Fig. S24, S25).

Tetracoordinate species typically show ^11^B NMR signals in the high-field region of the spectrum. The specific chemical shift values are governed by two main factors: (i) the electron density at the ^11^B nucleus, which reflects the donor strength of the coordinating ligand, and (ii) shielding effects arising from the magnetic anisotropy of the electron shells of the donor atoms, which are especially pronounced for heavier donors.^108^ To sidestep a comparative evaluation of magnetic anisotropy effects, we restrict our analysis to 2·Py *vs.*2·IDipp (2nd-row donor atoms; *δ*(^11^B) = −24.8 *vs.* −37.1) and 2·SMe_2_*vs.*2·PPh_3_ (3rd-row donor atoms; *δ*(^11^B) = −31.8 *vs.* −40.6). The observed trends align with the expectation that the more stable adducts are formed with IDipp and PPh_3_, respectively. The chlorinated compounds 3·Py *vs.*3·IDipp (*δ*(^11^B) = 3.7 *vs.* −4.4) and 3·SMe_2_*vs.*3·PPh_3_ (*δ*(^11^B) = 1.4 *vs.* −3.4) exhibit the same chemical-shift trends within each pair as observed for the corresponding 2·Do adducts. However, all four signals appear at markedly lower field, which we attribute to decreased magnetic anisotropy shielding when going from the BI_2_(Do) to the BCl_2_(Do) fragments. The ^29^Si NMR resonances of 2·Do and 3·Do were not detectable in the solution spectra, owing to unresolved ^1^*J*(B,Si) coupling and broadening induced by the quadrupolar ^11^B nucleus (*S* = 3/2).^108^ The ^31^P NMR spectra of 2·PPh_3_ and 3·PPh_3_ are characterized by multiplet resonances at −7.2 and 2.1 ppm, respectively.

Electron ionization (EI) mass spectra were recorded for the full series of donor adducts 2·Do and 3·Do (introduced as solids). In most cases, we observed the molecular ion peak [(Do)X_2_B–SiX_3_]˙^+^ and/or the peak corresponding to the donor-free silylborane [X_2_B–SiX_3_]˙^+^, typically with low intensity (X = Cl, I; see the SI for details). Most adducts appear to release their neutral Do ligand under the measurement conditions—except for IDipp, which resists elimination. Prominent fragmentation products included [(Do)BSiX_4_]^+^/[BSiX_4_]^+^ and [(Do)BX_2_]^+^. The latter may arise either by [SiX_3_]˙ loss from the parent ion or *via* a concerted pathway: [SiX_2_] extrusion from [(Do)X_2_B–SiX_3_]˙^+^, followed by X˙ elimination from the resulting [(Do)BX_3_]˙^+^ intermediate. This, in turn, raises the question—relevant for later reactivity studies—of whether neutral 2·Do and 3·Do might undergo thermal [SiX_2_] extrusion. To probe this, 2·IDipp was heated with the silylene-trapping reagent 2,3-dimethyl-1,3-butadiene (DMB; 10 eq.) in *o*DFB at 100 °C for 10 days in a flame-sealed NMR tube. [SiI_2_] was subsequently identified by GC-MS as its cycloadduct, 1,1-diiodo-3,4-dimethyl-1-silacyclopent-3-ene (Fig. S2).^109^ Consistently, the reaction mixture showed an ^11^B NMR signal corresponding to the byproduct BI_3_·IDipp formed through reductive elimination at the Si(iv) center of 2·IDipp (−77.3 ppm; confirmed by comparison with an authentic sample and X-ray crystal structure analysis of a single crystal grown in the NMR tube).

**Fig. 2:**
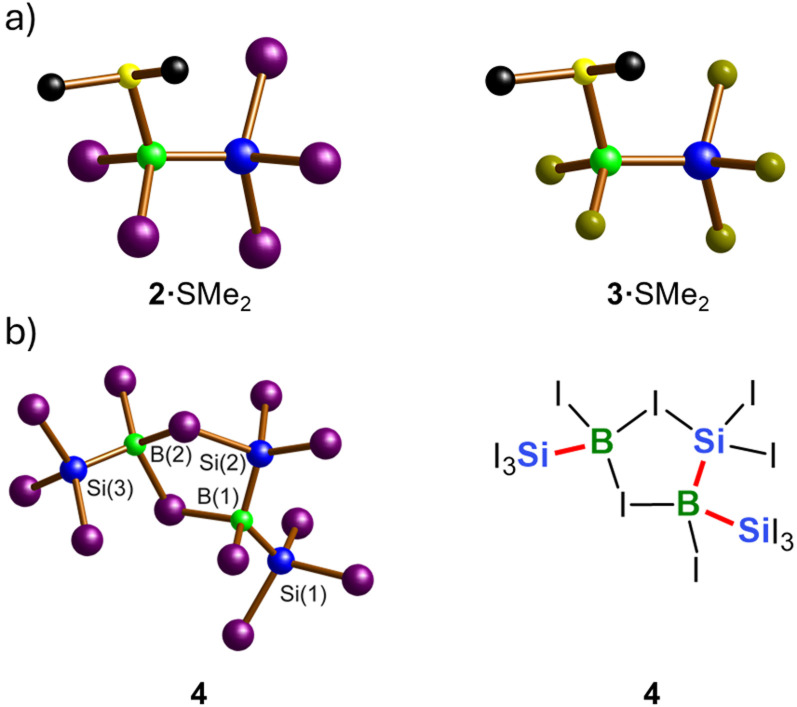
(a) Solid-state structures of 2·SMe_2_ (β-polymorph; left) and 3·SMe_2_ (right); (b) solid-state structure of the (S,S)-diastereomer of 4 (left) and its corresponding structural formula, with one BSi and one BSi_2_ moiety highlighted in red (right). H atoms are omitted for clarity. Color code: B: green, C: black, Si: blue, S: yellow, Cl: yellow-green, I: violet.

All eight adducts 2·Do and 3·Do were structurally characterized by X-ray crystallography (Fig. S103–S106 and S109–S112).^110^ Given the particular relevance of 2·SMe_2_ to silaboration reactions (see below), the molecular structures of this compound and its perchlorinated congener 3·SMe_2_ are shown as representative examples in Fig. 2a. All B–Si-bond lengths in 2·Do/3·Do fall within a narrow range of 2.005(3) to 2.043(4) Å, indicating that this parameter is not significantly influenced by either the nature of Do or the halogen ligand. In contrast, the B–Do bond lengths and the degree of pyramidalization at the B atom in the X_2_BSi fragments support the *a priori* expectations that (i) SMe_2_ is the weakest among the four donors Do, and (ii) the iodinated compound 2 is more Lewis acidic than its chlorinated analogue 3.^111^

Each molecule of 4 contains two chiral B atoms, giving rise to four possible diastereomers (Fig. 2b, left). In the lattice of the examined single crystal, two diastereomers [(S,S)/(S,R)] occupy the same crystallographic site in a 73 : 27 ratio, which leads to partial disorder involving the B(2)–Si(3) unit. This disorder, together with the comparatively weak scattering contribution of the light B atoms relative to the multiple heavy I atoms, limits the precision with which the B-atom positions and associated structural parameters can be determined. The five-membered ring in 4 features bridging I atoms (B–μ(I)–B and B–μ(I)–Si), resulting in tetracoordinate rather than tricoordinate, electron-deficient B sites (Fig. 2b, left).^112^ This feature prompts speculation that the extremely insoluble species 2 may adopt a polymeric or dimeric structure in the solid state, possibly featuring B_2_I_2_Si five-membered rings, with a single I atom replacing the Si(1)I_3_ substituent. Moreover, the presence of both a BSi and a BSi_2_ moiety in 4 (indicated by red-colored bonds in Fig. 2b, right) suggests that our methodology may provide access not only to perhalogenated silylboranes but also to disilylboranes.

### Silaboration reactions with 2·Do and 3·Do

One of the primary motivations for developing 2·Do and 3·Do was to create highly reactive silaboration reagents that allow for the simultaneous introduction of both derivatizable functional groups, ideally under catalyst-free conditions. Ethylene was selected as the initial model substrate for two main reasons: (i) its gaseous nature and lack of activating substituents make its silaboration particularly challenging, and (ii) the expected products are highly symmetric molecules with low molecular weight, which facilitates analysis by NMR spectroscopy and mass spectrometry.^61,94^

**Scheme 2:**
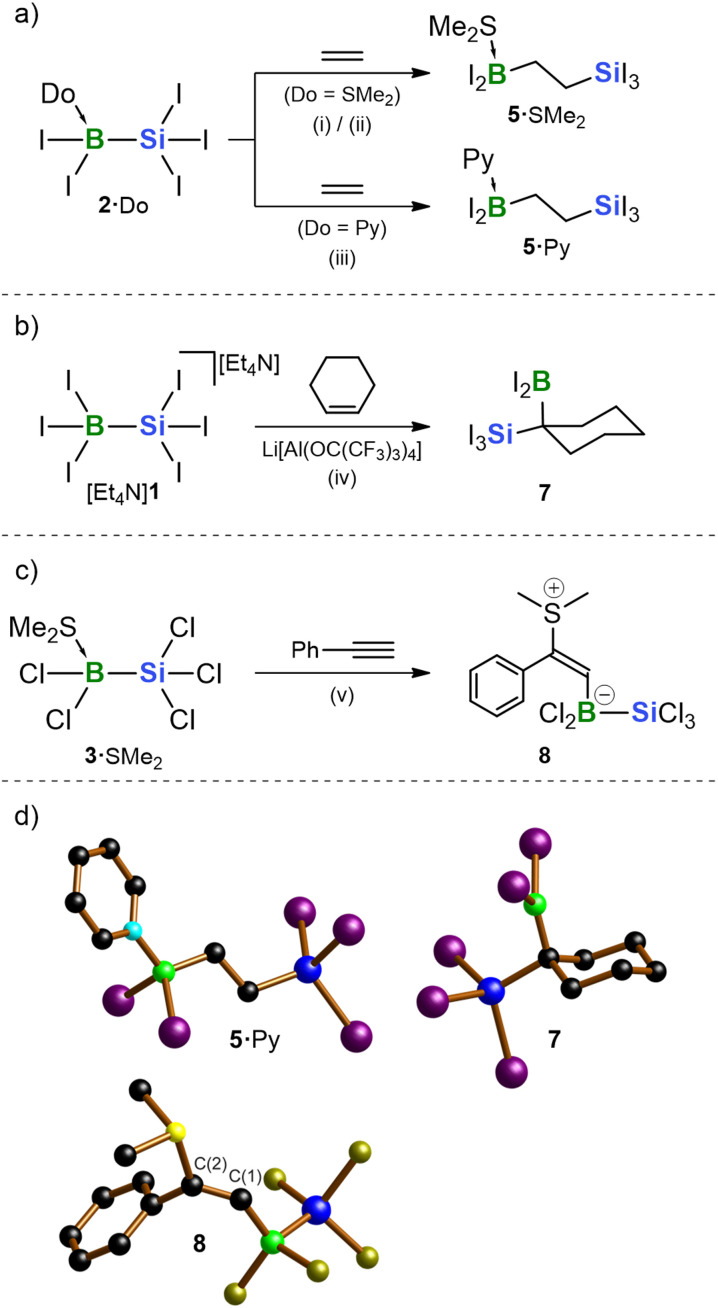
(a) 1,2-Silaborations of ethylene with 2·SMe_2_ or 2·Py afford 5·SMe_2_ or 5·Py. (b) 1,1-Silaboration of cyclohexene with [Et_4_N]1/Li[Al(OC(CF_3_)_3_)_4_] furnishes 7. (c) Reaction of phenylacetylene with 3·SMe_2_ leads to the addition of the 3/SMe_2_ Lewis pair across the C≡C bond to give 8. (d) Solid-state structures of 5·Py, 7, and 8; H atoms are omitted for clarity. Color code: B: green, C: black, N: pale blue, Si: blue, S: yellow, Cl: yellow-green; I: violet. Reagents and conditions: (i) exc. ethylene, CD_2_Cl_2_, 80 °C, 6 d, 98% yield; (ii) exc. ethylene, 0.1 eq. BI_3_, CD_2_Cl_2_, rt, 12 h, 85% yield; (iii) exc. ethylene, *o*DFB, 120 °C, 20 d, 97% yield; (iv) 10 eq. cyclohexene, 1 eq. Li[Al(OC(CF_3_)_3_)_4_], *o*DFB, rt, 15 min, 99% yield; (v) 5 eq. phenylacetylene, CH_2_Cl_2_, rt, 1 d, 37% yield.

The reactions were carried out by heating an excess of ethylene with 2·Do or 3·Do in CD_2_Cl_2_ or *o*DFB in sealed NMR tubes (Table 1 and Scheme 2a). *o*DFB was used when high temperatures and/or prolonged reaction times posed a risk of I/Cl exchange with CD_2_Cl_2_; for NMR measurements, *o*DFB was replaced with CD_2_Cl_2_ prior to measurement.

**Table 1:** Conditions and product distributions for the reactions of the adducts 2·Do or 3·Do with an excess of ethylene in sealed NMR tubes

Adduct	Conditions	Product(s)
2·SMe_2_	CD_2_Cl_2_, 6 d, 80 °C	5·SMe_2_, 98%
2·**SMe_2_/0.1 BI_3_**	**CD_2_Cl_2_, 12 h, rt**	5·**SMe_2_, 85%**
2·Py	*o*DFB, 20 d, 120 °C	5·Py, 97%
2·PPh_3_	*o*DFB, 20 d, 120 °C	5·PPh_3_, BI_3_·PPh_3_^b^
2·IDipp	*o*DFB, 6 d, 100 °C	BI_3_·IDipp^b^
3·SMe_2_	CD_2_Cl_2_, 31 d, 80 °C	6·SMe_2_, BCl_3_·SMe_2_^b^
3·Py	*o*DFB, 7 d^a^, 120 °C^a^	BCl_3_·Py^b^
3·PPh_3_	*o*DFB, 17 d, 120 °C	BCl_3_·PPh_3_^b^
3·IDipp	*o*DFB, 7 d^a^, 120 °C^a^	BCl_3_·IDipp^b^

a After the initial heating period, heating was continued at 140 °C for 1 day and at 160 °C for 1 day.

b The reaction mixture contained unconsumed starting material.

To evaluate general reactivity trends, we employed the pure silylborane adducts without added promoters. Under these conditions, 2·SMe_2_ underwent quantitative conversion with ethylene to afford the 1,2-silaboration product Me_2_S·I_2_B–C_2_H_4_–SiI_3_ (5·SMe_2_; Table 1 and Scheme 2a). This transformation proceeded to completion at 80 °C within 6 days. Likewise, 2·Py gave similarly high yields, albeit under even harsher conditions (120 °C, 20 d). At similar temperatures and reaction times, 2·PPh_3_ was only partially consumed; the fraction that reacted generated both the silaboration product 5·PPh_3_ and the thermolysis product BI_3_·PPh_3_. Among the perchlorinated analogues, only 3·SMe_2_ produced a notable amount of the corresponding 1,2-silaboration product. However, this transformation took five times longer than the reaction of 2·SMe_2_ and furnished Me_2_S·Cl_2_B–C_2_H_4_–SiCl_3_ (6·SMe_2_; Table 1) contaminated with residual starting material and the side product BCl_3_·SMe_2_. No silaboration was observed for the other adducts 2·Do and 3·Do; instead, they formed varying amounts of BX_3_·Do, likely due to thermally induced [SiX_2_] extrusion, as discussed above and corroborated by our previous trapping experiments with DMB. To promote the reaction between 2·SMe_2_ and ethylene, BI_3_ (0.1 eq.) was added to the mixture. Now, silaboration proceeded smoothly at rt within 12 h, affording 5·SMe_2_ in high yields (85%; entry 2 in Table 1). This final result of our systematic screening thus offers a practical and efficient access route to this promising functionalized building block. Notably, neither the Li[1]/2/LiI mixture nor free 3 provided further improvement, as both led to pronounced side reactions, presumably including ethylene polymerization. We further emphasize that haloboration did not compete with silaboration under any of the tested conditions.

Based on these experimental findings, two key principles emerge to guide further synthetic applications: (i) the highly reactive free species 2 and 3 must be tamed by adduct formation with a suitable donor to prevent non-selective transformations. In this regard, the soft ligand SMe_2_ performs best in terms of product selectivity while still allowing for reasonable temperatures and reaction times—especially when 0.1 eq. of BI_3_ is added as a promoter, which likely generates small concentrations of the free Lewis acid 2*in situ*. (ii) The iodinated adducts are more effective in silaborations than their chlorinated congeners. From that, we offer the following mechanistic interpretations: (i) silaborations with 2·Do and 3·Do are apparently not initiated by donor-induced B–Si-bond cleavage. Instead, displacement of Do by π-bonded ethylene must precede the 1,2-addition step (comparable B···olefin complexes have been structurally characterized by Yamaguchi *et al.*^113^). (ii) To maximize the interaction between the vacant B(p_z_) orbital and the π-electron cloud of ethylene, competing π-backbonding from X to B must be minimized, which accounts for the superior suitability of X = I (2-type compounds) over Cl (3-type compounds). A comprehensive quantum-chemical assessment of the overall reaction mechanism is provided below.

In a second reactivity test, a mixture of 2·SMe_2_ and the internal olefin cyclohexene in *o*DFB was heated to 120 °C for 24 days. Subsequent ^11^B NMR analysis of the sample showed essentially one signal at −11.6 ppm, indicating quantitative and selective conversion. As such forcing reaction conditions lack practical relevance, efforts were directed toward significantly accelerating the reaction prior to detailed product analysis. In this instance, the addition of BI_3_ as a promoter did not prove beneficial. However, a successful outcome was ultimately achieved using an equimolar mixture of [Et_4_N][1] and Li[Al(OC(CF_3_)_3_)_4_] in *o*DFB, which effected complete conversion within only 15 min at rt. It is evident that the increased kinetic protection of the C=C double bond in this case suppresses unwanted side reactions, even in the absence of any donor ligand apart from the residual I^−^ ions. More remarkably, olefin internalization exerts a decisive effect on regioselectivity: the reaction with cyclohexene selectively afforded the 1,1-silaboration product 7 rather than the 1,2-isomer (Scheme 2).^114^ Such a transformation is unprecedented—not only in silaboration but also in the related diboration or disilylation of olefins.^115^

In a final test experiment, phenylacetylene was chosen as the third representative substrate. Since the iodinated 2·SMe_2_ led to complex product mixtures, we turned to the chlorinated analogue 3·SMe_2_, which underwent complete conversion at rt after 1 day. From the reaction mixture, the zwitterionic species 8 crystallized in 37% yield (Scheme 2). Unlike 5·Do and 7 (Do = SMe_2_, Py), 8 is not generated *via* silaboration but instead represents the typical outcome of a concerted reaction between a free thioether Lewis base and a free borane Lewis acid acting on the same C≡C triple bond.^116–122^ This finding thus supports our earlier assumption that replacement of the B-bonded donor Do with the unsaturated substrate constitutes the initial step in the reactions of 2·Do and 3·Do. In the case of olefin substrates, both a boryl and a silyl group are introduced into the molecule. Yet, with phenylacetylene, the B–Si bond remains intact, and SMe_2_ is instead transferred to the substrate. In 8, the B atom is attached to the terminal position of the resulting olefin, while the SMe_2_ substituent resides near the phenyl ring. This can be explained by the fact that the positive charge accumulated on the carbon framework during electrophilic borylation is better stabilized by resonance at the α-position relative to the phenyl ring.

### NMR-spectroscopic and X-ray crystallographic characterization of 5·Do, 7, and 8 (ref. 107)

The ^11^B NMR spectra of the 1,2-silaboration products, 5·SMe_2_ and 5·Py, exhibit resonances at −18.9 and −14.0 ppm, respectively, consistent with the presence of tetracoordinate B nuclei.^108^ In contrast, the ^11^B NMR signal of the 1,1-silaboration product 7 appears at 53.5 ppm, indicative of a tricoordinate B center.^108^ The ^29^Si NMR shifts of 5·SMe_2_, 5·Py, and 7 are very similar with values of −115.1, −114.8, and −122.8 ppm, respectively. Furthermore, all three compounds give rise to signals exclusively in the aliphatic region of their ^1^H NMR spectra, confirming complete consumption of the C=C double bonds present in the starting materials. The resonances of the axial and equatorial H atoms within the cyclohexyl moiety of compound 7 are distinctly resolved, indicating that bulky substituents on the saturated ring act as effective conformational locks on the NMR timescale.^123,124^ The ^11^B NMR spectrum of 8 is characterized by a resonance at −2.6 ppm. As in the cases of 2·Do and 3·Do, the ^29^Si NMR signal of the B-bonded Si atom is broadened beyond detection. A singlet at *δ*(^1^H) = 7.35, together with a corresponding broad resonance at *δ*(^13^C) = 163.1, is consistent with the presence of an olefinic fragment in 8.

The molecular structures of 5·SMe_2_ (Fig. S115), 5·Py, and 7 (Scheme 2d), confirm their proposed identities as 1,2- and 1,1-silaboration products, respectively. The C–C-bond length in 5·Py falls within the typical single-bond range (1.533(4) Å), as do all C–C bonds in 7. As expected,^123,124^ the bulkier SiI_3_ substituent occupies an equatorial position, whereas the less bulky BI_2_ group adopts an axial orientation in the cyclohexane ring of 7. In contrast to compound 4 (Fig. 2), there is no B–μ(I)–Si bridge in 7; rather, the boryl group remains trigonal-planar coordinated. Nonetheless, the vacant B(p_z_) orbital may acquire some electron density from the occupied Si–C σ orbital, reminiscent of the well-known stabilization of carbenium ions bearing β-positioned silyl groups (see below).^125^ The C(1)–C(2) distance in compound 8 is 1.335(5) Å, characteristic of a double bond (Scheme 2d). The S and B atoms adopt a mutual *E* configuration, with the sterically demanding (silyl)boryl substituent located at the terminal position of the styrene core.

### Quantum-chemical calculations rationalizing the 1,2- *vs.* 1,1-silaboration of ethylene *vs.* cyclohexene to give 5·SMe_2_*vs.*7

**Fig. 3:**
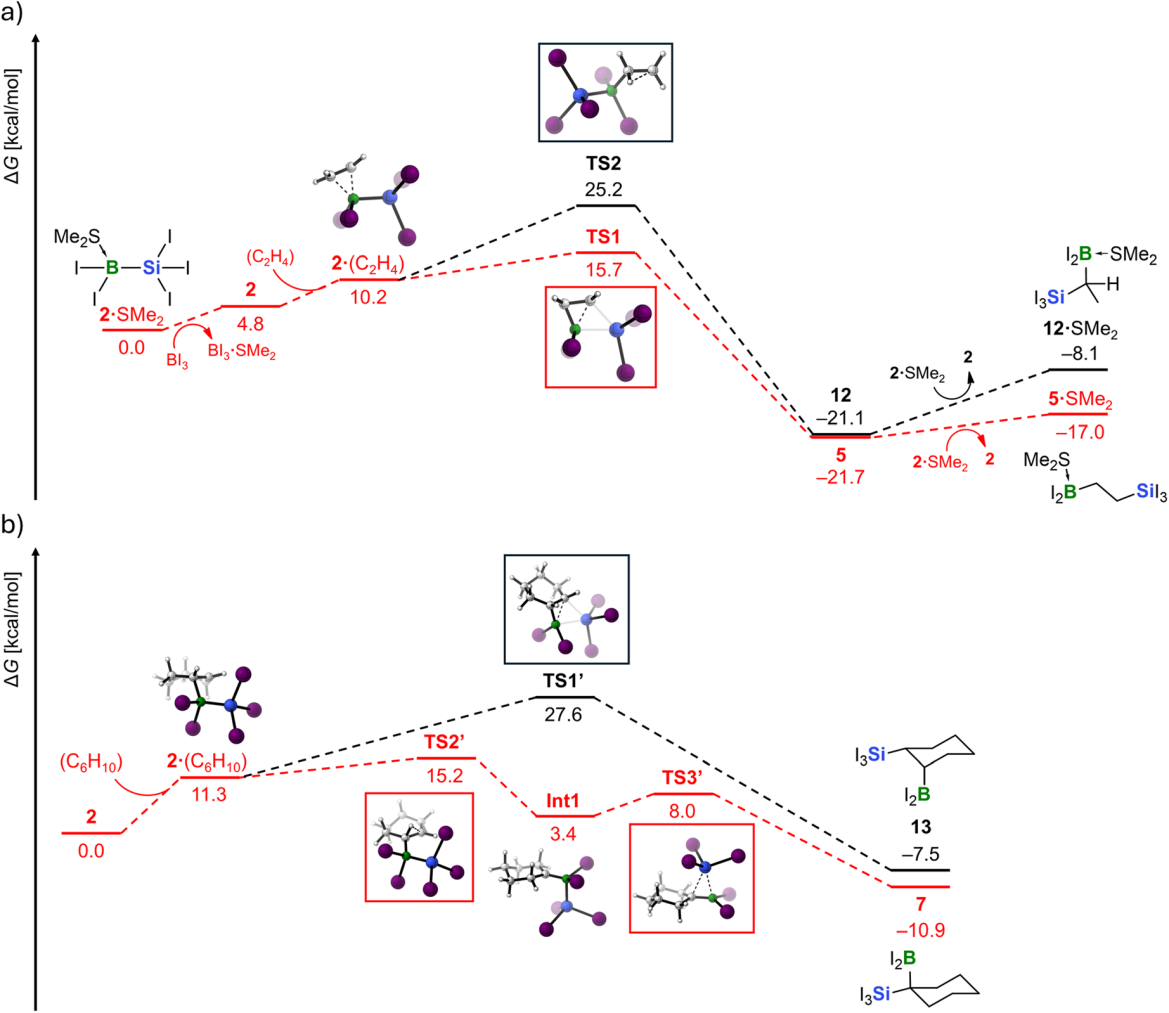
Computed reaction mechanisms for (a) the observed 1,2-silaboration of ethylene with 2·SMe_2_/0.1 BI_3_ (red) *vs.* the not observed 1,1-silaboration pathway (black) and (b) the observed 1,1-silaboration of cyclohexene with 2 (red) *vs.* the not observed 1,2-silaboration pathway (black). Color code: H: white, B: green, C: grey, Si: blue, I: violet. The Gibbs free energy changes (Δ*G*) were computed at the SMD(DCM)/PBE0-D3(BJ)/def2-QZVPPD level of theory, using geometries optimized at the SMD(DCM)/PBE0-D3(BJ)/def2-SVPD level. Note: compounds 9–11 appear in the SI as part of theoretically examined but energetically unfavorable alternative mechanisms.

For the reactions of 2·SMe_2_ and [Et_4_N][1]/Li[Al(OC(CF_3_)_3_)_4_] with the olefins, two scenarios were examined: 1,2-silaboration and 1,1-silaboration. Potentially competing haloboration pathways^126^ as well as, for 2·SMe_2_, the hypothetical addition of the Me_2_S/I_2_B–SiI_3_ Lewis pair to ethylene, were also considered but found to be irrelevant (see the SI for corresponding reaction pathways, activation barriers, and reaction energies). Fig. 3 shows the plausible silaboration sequences for ethylene (a) and cyclohexene (b). As a first important result, the experimentally observed products correspond to pathways that are both kinetically and thermodynamically favored (highlighted in red).

In the reaction of 2·SMe_2_ with ethylene, the SMe_2_ donor must first dissociate to generate a vacant coordination site at the B atom for olefin binding. The dissociation requires an energy input of 14.6 kcal mol^−1^ (*cf.* Fig. S134). However, the presence of BI_3_ renders the *in situ* release of the active silaboration reagent 2 significantly less endergonic (2·SMe_2_ + BI_3_ → 2 + BI_3_·SMe_2_; Δ*G* = 4.8 kcal mol^−1^). Subsequent ethylene binding to free 2 is endergonic by an additional 5.4 kcal mol^−1^. The resulting intermediate, 2·(C_2_H_4_), features a strongly pyramidalized B atom [∑(I–B–I/Si) = 320.2°]; the ethylene ligand remains essentially planar.^127^ The reaction proceeds *via* transition state TS1, characterized by B–Si-bond cleavage and the concerted formation of a C–Si bond. The 1,2-silaboration product 5 lies −21.7 kcal mol^−1^ below the starting materials, with an overall activation barrier of only 15.7 kcal mol^−1^. In the final step, 5 acquires an SMe_2_ ligand from 2·SMe_2_ to afford 5·SMe_2_ and free 2 with a similar endoergicity as observed in the case of 2·SMe_2_/BI_3_, explaining why only minor amounts of BI_3_ are necessary to promote the silaboration at rt. The alternative 1,1-silaboration of ethylene to furnish 12 would have to proceed *via* the much higher–energy transition state TS2 (Δ*G*^‡^ = 25.2 kcal mol^−1^ relative to the starting materials) and is thus not observed.

Due to the modified protocol used for the silaboration of cyclohexene, dissociation of SMe_2_ is not an issue here. Instead, free 2 can directly interact with the added olefin. Formation of the primary olefin complex 2·(C_6_H_10_) is somewhat more endergonic than in the case of ethylene (Δ*G* = 11.3 *vs.* 5.4 kcal mol^−1^), which can be attributed partly to steric factors and partly to a more pronounced reorganization energy: while the B atom in 2·(C_6_H_10_) is comparably pyramidalized as in 2·(C_2_H_4_), one B-bonded C atom now also deviates significantly from planarity [∑(C–C–C/H) = 348.2°].^127^ Starting from 2·(C_6_H_10_), two subsequent transition states are most relevant: TS1′ leads, *via* an overall activation barrier of 27.6 kcal mol^−1^, to the (experimentally unobserved) *syn*-1,2-silaboration product 13 (Δ*G* = −7.5 kcal mol^−1^). In contrast, TS2′, which lies 12.4 kcal mol^−1^ lower in energy than TS1′, corresponds to a 1,2-hydride shift leading to intermediate Int1. Subsequent 1,2-silyl migration *via* the low-lying TS3′ furnishes the experimentally obtained 1,1-silaboration product 7, with an overall reaction energy of Δ*G* = −10.9 kcal mol^−1^.

**Fig. 4:**
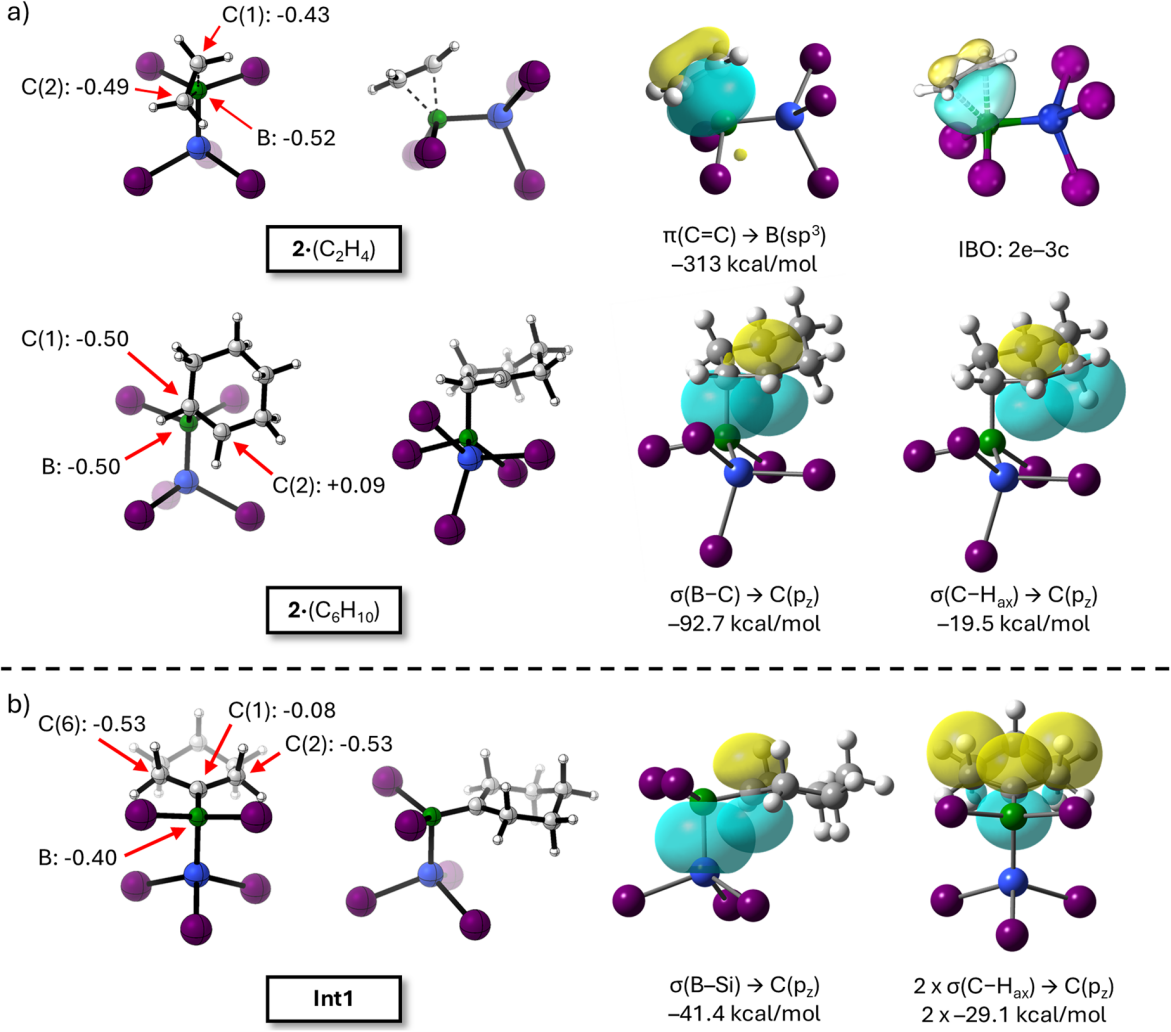
NBO and IBO analyses rationalizing the divergent silaboration pathways of ethylene and cyclohexene.^129^ Color code: H: white, B: green, C: grey, Si: blue, I: violet. (a) Left: optimized structures of 2·(C_2_H_4_) (top) and 2·(C_6_H_10_) (bottom) with selected NBO charges given in elementary charges (e); right: key orbital interactions with associated stabilization energies; top right: IBO representation of the two-electron–three-center (2e–3c) bond in 2·(C_2_H_4_). (b) Left: Optimized structure of Int1 with selected NBO charges given in elementary charges (e); right: hyperconjugative interactions stabilizing the carbenium ion, including the β-silicon effect and C–H_ax_ σ donation; SMD(DCM)/PBE0-D3(BJ)/def2-SVPD level of theory.

The differing regioselectivities observed in the silaborations of ethylene and cyclohexene arise as early as in intermediates 2·(C_2_H_4_) and 2·(C_6_H_10_): In 2·(C_2_H_4_), the ethylene coordination is near symmetric with B–C distances of 1.837 and 1.868 Å; the C(2)–C(1)–B–Si torsion angle is 23.0°, which represents an ideal conformation for an ensuing 1,2-silyl shift (Fig. 4a, left). According to a Natural Bond Orbital (NBO) analysis,^128^ all three atoms—B, C(1), and C(2)—carry negative partial charges of −0.52, −0.43, and −0.49 e, respectively. Intermediate 2·(C_2_H_4_) can thus be described as a σ-type donor–acceptor complex, in which charge is transferred from the occupied π-orbital of the olefin to the vacant orbital at the B atom, resulting in a substantial interaction energy of –313 kcal mol^−1^.^129^ Notably, an Intrinsic Bond Orbital (IBO)^130^ analysis even suggests the presence of a C–B–C two-electron–three-center (2e–3c) bond, with relative contributions of 29.4% (B), 35.4% (C(1)), and 34.7% (C(2); Fig. 4a, right). In 2·(C_6_H_10_), olefin binding is markedly unsymmetric, likely due to the higher steric bulk of cyclohexene relative to ethylene (Fig. 4a, left):^131^ a short σ bond is found between the B center and the pyramidalized C(1) atom (1.829 Å), while the distance to the still planar C(2) atom is significantly longer (B⋯C = 2.356 Å). Concomitantly, the torsion angle C(2)–C(1)–B–Si is increased to 52.0°, thereby disfavoring a 1,2-silyl shift due to the longer Si⋯C(2) distance that would have to be traversed in the corresponding transition state. While the NBO charges on B and C(1) in 2·(C_6_H_10_) remain comparably negative to those in 2·(C_2_H_4_), C(2) now carries a positive charge of +0.09*e*. Cyclohexene can accommodate the steric constraints, as the carbenium ion at C(2) is stabilized by both the +I effect of the alkyl substituent and hyperconjugative interactions^132–134^ between its vacant p_z_ orbital and the neighboring B–C and C–H_ax_ σ bonds with contributions worth –92.7 and –19.5 kcal mol^−1^ (ref. 129; Fig. 4a, right; see section 6.3.6 in the SI for a comparison with 3·(C_6_H_10_) where an NBO analysis reveals that 3 is coordinated primarily through a conventional, symmetric π→B interaction, most likely reflecting the lower Lewis acidity of 3). Similar hyperconjugative interactions as described for 2·(C_6_H_10_) are also present in the rearrangement intermediate Int1—this time between the carbenium ion's p_z_ orbital and the B–Si σ bond or two equivalent C–H_ax_ σ bonds (relative energy contributions: –41.4 and 2 × –29.1 kcal mol^−1^, respectively; Fig. 4b, right). The former interaction corresponds to the well-known β-effect of a silyl group.^125^ The overall stabilizing influence of steric and electronic factors makes Int1 thermodynamically more favorable than 2·(C_6_H_10_). In summary, the distinct regioselectivities in ethylene and cyclohexene silaboration originate from substrate-dependent binding geometries to 2: symmetric coordination of ethylene facilitates direct 1,2-silaboration, whereas the unsymmetric activation of cyclohexene favors a stepwise 1,1-pathway *via* a stabilized carbenium-ion intermediate. The computed energy profiles and bonding analyses offer a coherent explanation for the experimentally observed selectivities and underscore the critical influence of steric and electronic substrate effects in directing the specific silaboration pathway.

## Conclusions

The addition of a reactant X–Y across a C=C double bond is a perfectly atom-economic transformation. When employing versatile (orthogonal) functional groups for X and Y, the primary addition products can be made valuable platforms for a wide range of applications. This is particularly true for X–Y-type reactants featuring covalently bonded boryl and silyl groups: both substituents are not only among the most versatile handles for downstream functionalization, but also play key roles as property-defining units in organic functional materials. Consequently, there is a growing demand for the development of novel silaboration reactions and tailored silylborane reagents R_2_B–SiR_3_. We have now found a way to make perhalogenated derivatives (R = Cl, I) readily accessible on a multigram scale—both as free Lewis acids (*e.g.*, Cl_2_B–SiCl_3_) and as Lewis base adducts (Do·R_2_B–SiR_3_; Do = SMe_2_, Py, PPh_3_, IDipp). These developments create a versatile platform with the following key features: (i) Me_2_S·I_2_B–SiI_3_ and the *in situ*-generated mixture Li[I_3_B–SiI_3_]/I_2_B–SiI_3_/LiI react directly with olefins in silaboration reactions without the need for a catalyst, which is virtually without precedent.^135^ (ii) Cyclohexene undergoes a 1,1-addition reaction—so far unobserved not only for silaborations, but also for diboration and disilylation reactions. Combined experimental and quantum-chemical studies revealed that the steric demand of cyclohexene renders symmetrical coordination of the olefin to the B site unfavorable and instead promotes the formation of a zwitterionic B^⊖^(sp^3^)–C(sp^3^)–C^⊕^(sp^2^) fragment as a key entry point for the 1,1-silaboration cascade. While such a motif is prohibitively high in energy for ethylene, the carbenium center in the zwitterionic cyclohexene intermediate is efficiently stabilized through a combination of positive inductive (+I) and hyperconjugative effects. (iii) The halide substituents on the introduced boryl and silyl units enable diverse late-stage derivatizations—an aspect of particular importance when these functional groups are not merely used for transmetallation purposes in C–C-coupling reactions, but are instead retained as property-defining elements in the final molecule. (iv) Bulk Cl_2_B–SiCl_3_ can be distilled without decomposition. Considering that Si_2_Cl_6_ has been successfully used for the gas-phase deposition of silicon thin films,^136^ and B_2_F_4_ for their boron doping,^137,138^ Cl_2_B–SiCl_3_ emerges as a promising single-source precursor for semiconductor fabrication. Taken together, these findings pave the way for the future utilization of perhalogenated silylboranes in both synthesis (i–iii) and materials science (iv).

## Author contributions

J. H. performed the experimental studies and characterized all new compounds. C. D. B. performed the quantum-chemical calculations. A. V. V. and E. P. performed the X-ray crystal structure analyses of all compounds. H.-W. L., F. F. and M. W. supervised the project. The manuscript was written by J. H., C. D. B. and M. W. and edited by all co-authors.

## Conflicts of interest

There are no conflicts to declare.

## Supplementary Material

SC-016-D5SC06234A-s001

SC-016-D5SC06234A-s002

SC-016-D5SC06234A-s003

## Data Availability

CCDC 2470869, 2470870, 2470871, 2470872, 2470873, 2470874, 2470875, 2470876, 2470877, 2470878, 2470879, 2470880, 2470881, 2470882, 2470883, 2470884, 2470885, 2470886, 2470887, 2470888, and 2470889, contain the supplementary crystallographic data for this paper.^139*a*–*u*^ The data supporting this article have been included as part of the SI. Supplementary information is available. See DOI: https://doi.org/10.1039/d5sc06234a.
