# Supplementary material for: Synthesis of perhalogenated silylboranes (X = Cl, I) and their application in regiodivergent alkene silaboration
Source: Chem Sci. 2025 Sep 10;16(43):20329–43. doi: 10.1039/d5sc06234a (PMC12501792; doi:10.1039/d5sc06234a)
Supplement: SC-016-D5SC06234A-s002 [file SC-016-D5SC06234A-s002.pdf]

Supplementary Information (SI) for Chemical Science.
This journal is © The Royal Society of Chemistry 2025

Supporting Information

Catalyst-Free 1,1- and 1,2-Silaboration of Olefins Enabled by Perhalogenated Silylboranes

Jan Heller,+[a] Christoph Buch,+[a] Alexander Virovets,[a] Eugenia Peresypkina,[a] Hans-Wolfram Lerner,[a] Felipe Fantuzzi[b] and Matthias Wagner*[a]

[a] *Institut für Anorganische und Analytische Chemie, Goethe-Universität Frankfurt, Max-von-Laue-Straße 7, D-60438 Frankfurt (Main), Germany*

[b] *School of Chemistry and Forensic Science, University of Kent, Park Wood Rd, Canterbury CT2 7NH, UK*

*To whom correspondence should be addressed. Email: matthias.wagner@chemie.uni-frankfurt.de

Table of contents

1. Experimental details and characterization data S6
   1.1. Synthesis of BI_3 .... S7
   1.2. Synthesis of $[Et_4N][I_3B–SiI_3]$ ($[Et_4N][1]$) S8
      1.2.1. Synthesis of $[Et_4N][(I_{2.03}/Cl_{0.97})B–SiI_3]$ S9
   1.3. Cation exchange on $[Et_4N][1]$ with $Li[Al(OC(CF_3)_3)_4]$ S10
   1.4. Synthesis of the adducts $Do·I_2B–SiI_3$ (2·Do; Do = SMe_2, Py, PPh_3, IDipp) S11
      1.4.1. Characterization data of $Me_2S·I_2B–SiI_3$ ($2·SMe_2$) S11
      1.4.2. Characterization data of $Py·I_2B–SiI_3$ (2·Py) S12
      1.4.3. Characterization data of $Ph_3P·I_2B–SiI_3$ ($2·PPh_3$) S12
      1.4.4. Characterization data of $IDipp·I_2B–SiI_3$ (2·IDipp) S13
      1.4.5. Overview of ^{11}B and ^{29}Si NMR shifts of $Do·I_2B–SiI_3$ (2·Do) S13
   1.5. Synthesis of $Cl_2B–SiCl_3$ (3) S14
   1.6. Synthesis of $(I_2B–SiI_3)_2·SiI_2$ (4) S15
   1.7. Synthesis of $[Et_4N][Cl_3B–SiCl_3]$ S16
   1.8. Synthesis of the adducts $Do·Cl_2B–SiCl_3$ (3·Do; Do = SMe_2, Py, PPh_3, IDipp) S17
      1.8.1. Characterization data of $Me_2S·Cl_2B–SiCl_3$ ($3·SMe_2$) S17
      1.8.2. Characterization data of $Py·Cl_2B–SiCl_3$ (3·Py) S18
      1.8.3. Characterization data of $Ph_3P·Cl_2B–SiCl_3$ ($3·PPh_3$) S18
      1.8.4. Characterization data of $IDipp·Cl_2B–SiCl_3$ (3·IDipp) S19
      1.8.5. Overview of ^{11}B and ^{29}Si NMR shifts of $Do·Cl_2B–SiCl_3$ (3·Do) and $Cl_2B–SiCl_3$ (3) S19
   1.9. Synthesis of $BI_3·PPh_3$ S20
   1.10. Synthesis of $BI_3·IDipp$ S21
   1.11. Reactivities of the silylborane adducts $Do·I_2B–SiI_3$ (2·Do) and $Do·Cl_2B–SiICl_3$ (3·Do) S22
      1.11.1. Reaction of $Me_2S·I_2B–SiI_3$ ($2·SMe_2$) with ethylene to furnish $Me_2S·I_2B–C_2H_4–SiI_3$ ($5·SMe_2$) S22
      1.11.2. Reaction of $Py·I_2B–SiI_3$ (2·Py) with ethylene to give $Py·I_2B–C_2H_4–SiI_3$ (5·Py) S23
      1.11.3. Reaction of $Ph_3P·I_2B–SiI_3$ ($2·PPh_3$) with ethylene S24
      1.11.4. Reaction of $IDipp·I_2B–SiI_3$ (2·IDipp) with ethylene S24
      1.11.5. Reaction of $IDipp·I_2B–SiI_3$ (2·IDipp) with 2,3-dimethyl-1,3-butadiene S25
      1.11.6. Reaction of $Me_2S·Cl_2B–SiCl_3$ ($3·SMe_2$) with ethylene S27
      1.11.7. Reaction of $Py·Cl_2B–SiCl_3$ (3·Py) with ethylene S27
      1.11.8. Reaction of $Ph_3P·Cl_2B–SiCl_3$ ($3·PPh_3$) with ethylene S28
      1.11.9. Reaction of $IDipp·Cl_2B–SiCl_3$ (3·IDipp) with ethylene S28
   1.12. Stability of the silylborane adducts $Do·I_2B–SiI_3$ (2·Do) under sunlight exposure S28
   1.13. Reaction of 2 with cyclohexene to give $(I_2B–)(I_3Si–)C_6H_{10}$ (7) S29

1.14. Reaction of $Me_2S·I_2B–SiI_3$ ($2·SMe_2$) with cyclohexene S29
1.15. Reaction of $BI_3·SMe_2$ with ethylene to give $Me_2S·I_2B–C_2H_4–I$ S30
1.16. Reaction of $Me_2S·Cl_2B–SiCl_3$ ($3·SMe_2$) with phenylacetylene to give 8 .... S31
2. Plots of NMR spectra S32
2.1. NMR spectrum of BI_3 .... S32
2.2. NMR spectrum of $[Et_4N][1]$ S32
2.3. NMR spectrum of $[Et_4N][(I_{2.03}/Cl_{0.97})B–SiI_3]$ S33
2.4. NMR spectra of Li[1], 2/LiI S33
2.5. NMR spectra of $Me_2S·I_2B–SiI_3$ ($2·SMe_2$) S34
2.6. NMR spectra of $Py·I_2B–SiI_3$ (2·Py) S35
2.7. NMR spectra of $Ph_3P·I_2B–SiI_3$ ($2·PPh_3$) S36
2.8. NMR spectra of $IDipp·I_2B–SiI_3$ (2·IDipp) S37
2.9. NMR spectra of $Cl_2B–SiCl_3$ (3) S39
2.10. NMR spectra of $(I_2B–SiI_3)_2·SiI_2$ (4) S40
2.11. NMR spectra of $[Et_4N][Cl_3B–SiCl_3]$ S41
2.12. NMR spectra of $Me_2S·Cl_2B–SiCl_3$ ($3·SMe_2$) S42
2.13. NMR spectra of $Py·Cl_2B–SiCl_3$ (3·Py) S43
2.14. NMR spectra of $Ph_3P·Cl_2B–SiCl_3$ ($3·PPh_3$) S44
2.15. NMR spectra of $IDipp·Cl_2B–SiCl_3$ (3·IDipp) S45
2.16. NMR spectra of $BI_3·PPh_3$ S47
2.17. NMR spectra of $BI_3·IDipp$ S48
2.18. NMR spectra of $Me_2S·I_2B–C_2H_4–SiI_3$ ($5·SMe_2$) S50
2.19. NMR spectra of $Py·I_2B–C_2H_4–SiI_3$ (5·Py) S52
2.20. NMR spectra recorded on the reaction mixture of $Ph_3P·I_2B–SiI_3$ ($2·PPh_3$) with ethylene .. S54
2.21. NMR spectrum recorded on the reaction mixture of $IDipp·I_2B–SiI_3$ (2·IDipp) with ethylene S55
2.22. NMR spectra recorded on the reaction mixture of $IDipp·I_2B–SiI_3$ (2·IDipp) with 2,3-dimethyl-1,3-butadiene S55
2.23. NMR spectra recorded on the reaction mixture of $Me_2S·Cl_2B–SiCl_3$ ($3·SMe_2$) with ethylene S56
2.24. NMR spectrum recorded on the reaction mixture of $Py·Cl_2B–SiCl_3$ (3·Py) with ethylene .. S57
2.25. NMR spectra of $BCl_3·Py$ S57
2.26. NMR spectrum recorded on the reaction mixture of $Ph_3P·Cl_2B–SiCl_3$ ($3·PPh_3$) with ethylene S59
2.27. NMR spectrum recorded on the reaction mixture of $IDipp·Cl_2B–SiCl_3$ (3·IDipp) with ethylene S59
2.29. NMR spectra of $(I_2B–)(I_3Si–)C_6H_{10}$ (7) S60

2.30. NMR spectra recorded on the reaction mixture of $Me_2S{\cdot}I_2B–SiI_3$ ($2{\cdot}SMe_2$) with cyclohexene S61
2.31. NMR spectra of $Me_2S{\cdot}I_2B–C_2H_4–I$ S62
2.32. NMR spectra of 8 .... S63
3. Plots of mass spectra S66
3.1. Mass spectrum of $Me_2S{\cdot}I_2B–SiI_3$ ($2{\cdot}SMe_2$) S66
3.2. Mass spectrum of $Py{\cdot}I_2B–SiI_3$ ($2{\cdot}Py$) S67
3.3. Mass spectrum of $Ph_3P{\cdot}I_2B–SiI_3$ ($2{\cdot}PPh_3$) S68
3.4. Mass spectrum of $IDipp{\cdot}I_2B–SiI_3$ ($2{\cdot}IDipp$) S69
3.5. Mass spectrum of $Cl_2B–SiCl_3$ (3) S70
3.6. Mass spectrum of $[Et_4N][Cl_3B–SiCl_3]$ S70
3.7. Mass spectrum of $Me_2S{\cdot}Cl_2B–SiCl_3$ ($3{\cdot}SMe_2$) S71
3.8. Mass spectrum of $Py{\cdot}Cl_2B–SiCl_3$ ($3{\cdot}Py$) S72
3.9. Mass spectrum of $Ph_3P{\cdot}Cl_2B–SiCl_3$ ($3{\cdot}PPh_3$) S73
3.10. Mass spectrum of $IDipp{\cdot}Cl_2B–SiCl_3$ ($3{\cdot}IDipp$) S74
3.11. Mass spectrum of $BI_3{\cdot}PPh_3$ S74
3.12. Mass spectrum of $BI_3{\cdot}IDipp$ S75
3.13. Mass spectrum of $Py{\cdot}I_2B–C_2H_4–SiI_3$ ($5{\cdot}Py$) S75
3.14. Mass spectrum of $(I_2B–)(I_3Si–)C_6H_{10}$ (7) S75
3.15. Mass spectrum of $Me_2S{\cdot}I_2B–C_2H_4–I$ S75
3.16. Mass spectrum of 8 .... S76
4. Single-crystal X-ray structure analysis S77
4.1. Crystal Structure of $[Et_4N][(I_{2.03}/Cl_{0.97})B–SiI_3]$ S89
4.2. Crystal Structure of dimorphic $Me_2S{\cdot}I_2B–SiI_3$ ($2{\cdot}SMe_2$) S90
4.3. Crystal Structure of $Py{\cdot}I_2B–SiI_3$ ($2{\cdot}Py$) S91
4.4. Crystal Structure of dimorphic $Ph_3P{\cdot}I_2B–SiI_3$ ($2{\cdot}PPh_3$) S92
4.5. Crystal Structure of $IDipp{\cdot}I_2B–SiI_3$ ($2{\cdot}IDipp$) S93
4.6. Crystal Structure of $(I_2B–SiI_3)_2{\cdot}SiI_2$ (4) S93
4.7. Crystal Structure of $[Et_4N][Cl_3B–SiCl_3]$ S94
4.8. Crystal Structure of $Me_2S{\cdot}Cl_2B–SiCl_3$ ($3{\cdot}SMe_2$) S95
4.9. Crystal Structure of $Py{\cdot}Cl_2B–SiCl_3$ ($3{\cdot}Py$) S95
4.10. Crystal Structure of $Ph_3P{\cdot}Cl_2B–SiCl_3$ ($3{\cdot}PPh_3$) S96
4.11. Crystal Structure of $IDipp{\cdot}Cl_2B–SiCl_3$ ($3{\cdot}IDipp$) S97
4.12. Crystal Structure of $BI_3{\cdot}PPh_3$ S98
4.13. Crystal Structure of $BI_3{\cdot}IDipp$ S98
4.14. Crystal Structure of $Me_2S{\cdot}I_2B–C_2H_4–SiI_3$ ($5{\cdot}SMe_2$) S99

4.15. Crystal Structure of Py·I_2B–C_2H_4–SiI_3 (5·Py) S99
4.16. Crystal Structure of (I_2B–)(I_3Si–)C_6H_{10} (7) S100
4.17. Crystal Structure of Me_2S·I_2B–C_2H_4–I S100
4.18. Crystal Structure of 8 .... S101
5. X-ray powder diffraction S102
5.1. X-ray powder diffractometry on Me_2S·I_2B–SiI_3 (2·SMe_2) S102
5.2. X-ray powder diffractometry on Me_2S·I_2B–C_2H_4–SiI_3 (5·SMe_2) S103
5.3. X-ray powder diffractometry on (I_2B–)(I_3Si–)C_6H_{10} (7) S103
6. Computational details S104
6.1. Computed mechanism of the reaction of 2·SMe_2 with ethylene S104
6.1.1. Adduct formation of 2 with C_2H_4 .... S105
6.1.2. 1,2-silaboration S105
6.1.3. 1,2-iodoboration S106
6.1.4. 1,2-thiaboration S107
6.1.5. 1,1-silaboration S110
6.1.6. ΔG profiles of SMe_2 adduct formations S111
6.1.7. Summarized mechanism of 2·SMe_2 with C_2H_4 .... S113
6.2. Computed mechanism of the reaction of 2 with cyclohexene S113
6.2.1. Adduct formation of 2 with C_6H_{10} S113
6.2.2. 1,2 hydride shift in 2·C_6H_{10} and subsequent 1,2 silyl shift S114
6.2.3. Transition state for 1,2-silaboration in 2·C_6H_{10} S116
6.2.4. Summarized mechanism of the reaction of 2·with C_6H_{10} S118
6.3. Natural bond orbital (NBO) analysis S118
6.3.1. NBO analysis of 2·C_2H_4 .... S118
6.3.2. IBO analysis of 2·C_2H_4 .... S120
6.3.3. NBO analysis of 2·C_6H_{10} S121
6.3.4. NBO analysis of Int1 .... S123
6.3.5. NBO analysis of 7 .... S124
6.3.6 NBO analysis of 3·C_6H_{10} S125
6.4. Computed structures and corrected free energies S128
7. References S132

1. Experimental details and characterization data

General considerations

All reactions, manipulations, and analyses were carried out under exclusion of air and moisture using Schlenk techniques or a glovebox.

$Li[Al(OC(CF_3)_3)_4]$ was synthesized according to the procedure published by Krossing.[S1] Commercially available starting materials were used as received. *n*-Heptane and C_6H_6 were dried over Na metal and distilled prior to use. CH_2Cl_2 was distilled from CaH_2 and stored over molecular sieves (3 Å). CD_2Cl_2, C_6D_6, and 1,2-difluorobenzene (*o*DFB) were degassed by three freeze-pump-thaw cycles and stored over molecular sieves (3 Å). Before ethylene was condensed onto a sample, it was passed through a U-shaped tube filled with molecular sieves (3 Å) to remove traces of water.

NMR spectra were recorded at 298 K using the following *Bruker* Spectrometers: *Avance*™ *II 300*, *Avance*™ *III 400 HD,* or *Avance*™ *III 500 HD*. NMR samples were investigated in flame-sealed NMR tubes. Chemical shift values (ppm) are referenced to (residual) solvent signals ($^1H/^{13}C\{^1H\}$; CD_2Cl_2: 5.32 ppm/53.84 ppm; C_6D_6: 7.16 ppm/128.06 ppm[S2]) or to external standards (^{11}B: $F_3B{\cdot}OEt_2$; $^{29}Si\{^1H\}$: $SiMe_4$; $^{31}P\{^1H\}$: H_3PO_4). Resonance assignments were aided by ^{13}C-^1H-HSQC, ^{13}C-^1H-HMBC, ^1H-^1H-NOESY, and ^{29}Si-^1H-HMBC experiments. Resonances of ^{13}C or ^{29}Si atoms attached to B atoms were typically broadened due to the quadrupolar relaxation of the $^{10/11}B$ nuclei. While the corresponding ^{29}Si resonances could not be detected in most cases, the ^{13}C resonances could be localized via ^1H-^{13}C-HMBC NMR experiments. Abbreviations: s = singlet, d = doublet, dd = doublet of doublets, t = triplet, tt = triplet of triplets, q = quartet, qt = quartet of triplets, sept = septet, m = multiplet, br = broad, n.o. = not observed, n.r. = not resolved, $h_{½}$ = full width at half maximum.

MAS NMR experiments were recorded on a Bruker Avance Neo 600 MHz spectrometer equipped with a wide bore Bruker 4.0 mm MAS HX probe, at MAS rates of 9, 10 or 12 kHz at 298 K. The samples were finely ground and packed in 4 mm outer diameter ZrO_2 rotors. For all ^{11}B measurements, a standard Hahn echo pulse sequence ($\pi/2 - \tau - \pi - \tau -$ acquire) was used to minimize probe background signals using a 2 µs $\pi/2$ pulse, τ equal to one rotor period and a recycle delay of 4 or 10 s. ^{11}B chemical shifts were externally referenced to powdered $NaBH_4$, which resonates at –42.06 ppm relative to the primary ^{11}B standard, $F_3B{\cdot}OEt_2$. ^{29}Si spectra were recorded using direct excitation with a 5 µs pulse and a recycle delay of 20 or 40 s. ^{29}Si chemical shifts were externally referenced to powdered $Si(SiMe_3)_4$, using the most deshielded ^{29}Si signal at –9.8 ppm relative to the primary silicon reference, $SiMe_4$.[S3–5]

Mass spectra (EI) were measured using a ***JEOL AccuTOF GCx***. The isotope patterns of the selected ion species were compared to the theoretical pattern calculated from the elemental composition of the ions using the software mMass. All observed patterns show excellent agreement with the simulations.[S6–8]

GC-MS (gas chromatography – mass spectrometry) data were recorded using a *Shimadzu GCMS-QP2010 SE apparatus*. The stationary phase (Restek) had a length of 60 m with an inner diameter of 0.32 mm. The analyte was dissolved in CD_2Cl_2 or *o*DFB prior to the measurement. To avoid overloading the MS, a solvent cut was used. Samples were injected at 200 °C and transferred onto the column with a flow rate of 1.86 mL/min, carried by He gas. The oven was heated to 50 °C for 1 min, the temperature was subsequently increased at a rate of 20 °C/min up to 230 °C and held for 25 min. Finally, the oven temperature was increased again at a rate of 25 °C/min up to 270 °C and held for 5 min. After a certain retention time τ, the substances exited the column and were ionized with 70 eV; cationic fragments were measured within a range of m/z = 35–800 (mass per charge).

Elemental analyses were performed by *Mikroanalytisches Labor Pascher*, D-53424 Remagen.

1.1. Synthesis of BI_3

An efficient reflux condenser was fitted to the central neck of a 2 L three-necked-flask, with the second neck sealed with a stopper and the third neck connected to a Schlenk line (all glass joints should be lubricated with halogen-resistant grease). The apparatus should be protected from direct sunlight to avoid photolysis of BI_3.[S9]

I_2 (500 g, 1.97 mol, 1.25 eq.) was dissolved in *n*-heptane (1.3 L), and neat KBH_4 (85.0 g, 1.58 mol, 1.00 eq.) was added to the resulting solution with stirring. The reaction mixture was gradually heated to 80 °C. During heating, dense white fumes were observed. The temperature was maintained until the fumes dissipated. Stirring was continued at 80 °C until the reaction mixture became colorless (minimum of 10 h); the progress of the reaction was monitored by the fading of the color imparted by the still unconsumed I_2. The resulting solids were separated from the supernatant by Schlenk filtration and repeatedly rinsed with *n*-heptane (4x50 mL) during filtration. Within 2 h, the *n*-heptane was removed from the filtrate by evaporation at rt under reduced pressure into a trap cooled with liquid N_2. Neat BI_3 was obtained as colorless crystals (186 g, 0.48 mol, 48%, based on I_2 and on the formal reaction equation: $KBH_4 + 2\ I_2 \rightarrow BI_3 + 2H_2 + KI$).

^{11}B NMR (96.3 MHz, C_6D_6): δ = –7.4 (s, $h_{½}$ ≈ 30 Hz).

Note: A similar synthesis protocol for BI_3 was reported by Briggs et al. in 1990, affording yields of 2.4 g (35%, based on I_2 and on the formal reaction equation: $KBH_4 + 2\ I_2 \rightarrow BI_3 + 2H_2 + KI$).[S9] To the best of our knowledge, no subsequent procedure has significantly improved either the yield or the quantity of product obtained. In this work, we present an optimized procedure, based on that from Briggs et al., that produces higher yields and larger quantities of pure BI_3. This demonstrates that BI_3 is readily accessible for further synthetic applications.

1.2. Synthesis of $[Et_4N][I_3B–SiI_3]$ ($[Et_4N][1]$)

In an amber glass vessel, $[Et_4N]Cl$ (19.2 g, 116 mmol, 2.00 eq.) was dissolved in CH_2Cl_2 (200 mL). Neat solid BI_3 (45.4 g, 116 mmol, 2.00 eq.) was added to the solution with stirring at rt. An exothermic reaction took place, during which the reaction mixture turned pale yellow and a colorless precipitate formed. Si_2Cl_6 (10.0 mL, 15.6 g, 58.0 mmol, 1.00 eq.) in CH_2Cl_2 (10 mL) was added in one portion to the stirred reaction mixture at rt, resulting in a second exothermic reaction and formation of a clear solution. A second portion of BI_3 (2.27 g, 5.80 mmol, 0.10 eq.) was added with stirring rt. After 24 h of stirring at rt, a colorless solid had formed. The solid was isolated by filtration, rinsed repeatedly with CH_2Cl_2 (5x10 mL), and dried in vacuo. $[Et_4N]$[**1**] was obtained as colorless solid (38.7 g, 41.6 mmol, 72%).

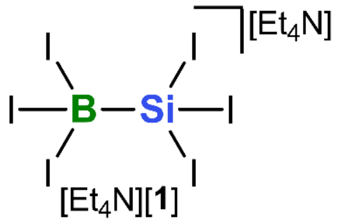

$[Et_4N]$[**1**]

^{11}B NMR (96.3 MHz, CD_2Cl_2): δ = –60.1 (s, $h_{½}$ ≈ 35 Hz).

^{29}Si NMR (59.6 MHz, CD_2Cl_2): n.o.

Solid-state ^{11}B NMR (192.6 MHz, MAS, spinning rate: 9 kHz): δ = –60 (s, $h_{½}$ ≈ 2500 Hz).

Solid-state ^{29}Si NMR (119.2 MHz, MAS, spinning rate: 10 kHz): δ = –81 (s, $h_{½}$ ≈ 1210 Hz).

Note: Due to the poor solubility of $[Et_4N]$[**1**] and the strong broadening of the silicon resonance resulting from quadrupolar relaxation effects of the adjacent boron atom, no ^{29}Si NMR signals could be observed in solution. Consequently, solid-state NMR spectra of $[Et_4N]$[**1**] were recorded. The more pronounced signal at –81 ppm in the ^{29}Si NMR spectrum (Figure S7) can tentatively be assigned to $[Et_4N]$[**1**], while the signal at –106 ppm likely corresponds to **2** (Figure S7).

Note: The synthesis of $[Et_4N]$[**1**] is based on a protocol published by Teichmann et al.[S10] However, Teichmann's original protocol was conducted on a small scale and furnished a mixture of two products, $[Et_4N]$[**1**] and an initially unknown compound. We have now identified this side product as $[Et_4N][I_2ClB–SiI_3]$ (see chapter 1.2.1). The second portion of BI_3 is required to ensure quantitative conversion of $[Et_4N][I_2ClB–SiI_3]$ to $[Et_4N]$[**1**].

1.2.1. Synthesis of $[Et_4N][(I_{2.03}/Cl_{0.97})B–SiI_3]$

In an amber glass vessel, Et_4NCl (423 mg, 2.55 mmol, 2.00 eq.) was dissolved in CH_2Cl_2 (2 mL) at rt. Neat solid BI_3 (1.00 g, 2.55 mmol, 2.00 eq.) was added with stirring at rt, resulting in an exothermic reaction and the development of a pale yellow color. Subsequently, neat Si_2Cl_6 (0.220 mL, 343 mg, 1.28 mmol, 1.00 eq.) was added in one portion with stirring at rt, triggering a second exothermic reaction and yielding a clear solution. Stirring was stopped at this point, and within a few minutes, colorless crystals formed. The crystals were isolated by filtration, rinsed repeatedly with CH_2Cl_2 (5x1 mL), and dried in vacuo. $[Et_4N][(I_{2.03}/Cl_{0.97})B–SiI_3]$ was obtained as colorless crystals suitable for X-ray diffraction (641 mg, 763 µmol, 60%).

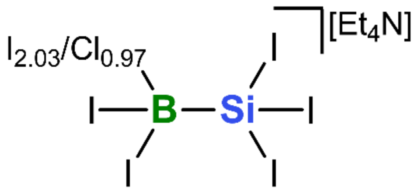

$[Et_4N][(I_{2.03}/Cl_{0.97})B–SiI_3]$

Note: Single crystals of $[Et_4N][(I_{2.03}/Cl_{0.97})B–SiI_3]$ consist of a solid solution of different chlorinated and iodinated species.

^{11}B NMR (96.3 MHz, CD_2Cl_2): δ = –32.5 (s, $h_{½}$ ≈ 36 Hz), –60.1 (s, $h_{½}$ ≈ 30 Hz).

Note: The more pronounced signal at –32.5 ppm in the ^{11}B NMR spectrum can be assigned to $[Et_4N][I_2ClB–SiI_3]$, while the signal at –60.1 ppm corresponds to $[Et_4N]$[**1**].

1.3. Cation exchange on $[Et_4N][1]$ with $Li[Al(OC(CF_3)_3)_4]$

$[Et_4N]$[**1**] (500 mg, 537 µmol, 1.00 eq.) and $Li[Al(OC(CF_3)_3)_4]$ (576 mg, 591 µmol, 1.10 eq.) were suspended in *o*DFB (20 mL) and stirred at rt. After 24 h, all insolubles were separated from the supernatant by filtration. The solids were thoroughly rinsed with *o*DFB (10x2 mL) to remove residual $[Et_4N][Al(OC(CF_3)_3)_4]$ and dried in vacuo. A mixture of Li[**1**] and **2**/LiI was obtained as a colorless solid (265 mg), precluding the determination of a yield.

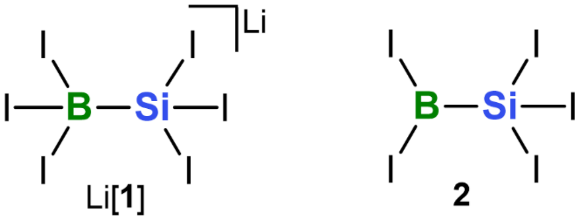

Li[**1**]

2

Note: Due to solubility issues in all inert solvents, solid-state NMR spectra of the mixture of Li[**1**] and **2**/LiI were recorded. The solid-state ^{11}B NMR spectrum shows a signal at 10 ppm (Figure S9), which likely corresponds to **2**/LiI. A second signal at –54 ppm is tentatively assigned to Li[**1**]. The solid-state ^{29}Si NMR spectrum shows a signal at –106 ppm corresponding to **2**/LiI and a signal at –70 ppm assignable to Li[**1**] (Figure S10).

1.4. Synthesis of the adducts Do·I_2B–SiI_3 (2·Do; Do = SMe_2, Py, PPh_3, IDipp)

General procedure: [Et_4N][**1**] (cf. Table S1, 1.00 eq.) and Li[Al(OC(CF_3)$_3$)$_4$] (cf. Table S1, 1.10 eq.) were suspended in CH_2Cl_2 (10 mL), and the respective Lewis basic donor (Do; cf. Table S1, 1.10 eq.) was added with stirring at rt. After 24 h, all insolubles were separated from the supernatant by filtration. The colorless filtrate (in the case of Do = IDipp it was red; IDipp: 1,3-bis(2,6-diisopropylphenyl)-1,3-dihydro-*2H*-imidazol-2-ylidene) was then allowed to evaporate slowly at rt, whereupon colorless crystals formed. These crystals were rinsed with *o*DFB (3x0.5 mL) to remove residual [Et_4N][Al(OC(CF_3)$_3$)$_4$] and dried in vacuo. Compounds of type **2**·Do were obtained as colorless crystals suitable for X-ray diffraction (cf. Table S1, 71–91%).

Note: Single crystals of a second polymorph of **2**·SMe_2 (β) and **2**·PPh_3 (α) were obtained by slow evaporation of the respective *o*DFB solution at rt.

Table S1: Quantities of [Et_4N][**1**], Li[Al(OC(CF_3)$_3$)$_4$], and Do used in the synthesis of **2**·Do.

	[Et_4N][**1**]	Li[Al(OC(CF_3)$_3$)$_4$]	Do	yield of **2**·Do
Do = SMe_2	2.30 g, 2.47 mmol	2.65 g, 2.72 mmol	200 µL, 169 mg, 2.72 mmol	1.65 g, 2.24 mmol, 91%
Do = Py	1.00 g, 1.07 mmol	1.15 g, 1.18 mmol	95.4 µL, 93.5 mg, 1.18 mmol	672 mg, 893 µmol, 83%
Do = PPh_3	500 mg, 537 µmol	576 mg, 591 µmol	155 mg, 591 µmol	435 mg, 465 µmol, 87%
Do = IDipp	500 mg, 537 µmol	576 mg, 591 µmol	230 mg, 591 µmol	406 mg, 382 µmol, 71%

1.4.1. Characterization data of Me_2S·I_2B–SiI_3 (2·SMe_2)

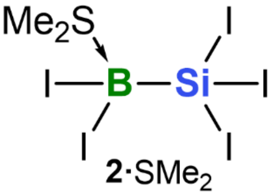

2·SMe_2

^{1}H NMR (500.2 MHz, CD_2Cl_2): δ = 2.73 (s, 6H, Me_2S).

^{11}B NMR (160.5 MHz, CD_2Cl_2): δ = –31.8 (s, $h_{½}$ ≈ 33 Hz).

^{13}C{^{1}H} NMR (125.8 MHz, CD_2Cl_2): δ = 27.3 (Me_2S).

^{29}Si NMR (99.4 MHz, CD_2Cl_2): δ = n.o.

EI-MS(+): selected peaks: *m/z* = 735.5282 ([$C_2H_6BI_5SSi$]$^{\bullet+}$; calcd. 735.5275), 673.5053 ([BI_5Si]$^{\bullet+}$; calcd. 673.5084), 608.6221 ([$C_2H_6BI_4SSi$]$^+$; calcd.: 608.6230), 546.6002 ([BI_4Si]$^+$; calcd.: 546.6040), 326.8363 ([$C_2H_6BI_2S$]$^+$; calcd.: 326.8371).

1.4.2. Characterization data of Py·I_2B–SiI_3 (2·Py)

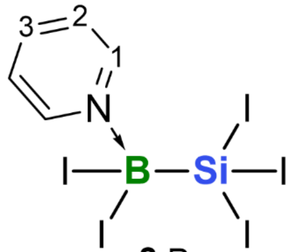

2·Py

^{1}H NMR (500.2 MHz, CD_2Cl_2): δ = 9.78 (d, ^{3}J(H,H) = 6.2 Hz, 2H, H-1), 8.31 (tt, ^{3}J(H,H) = 6.2 Hz, ^{4}J(H,H) = 1.5 Hz, 1H, H-3), 7.83 (m, 2H, H-2).

^{11}B NMR (160.5 MHz, CD_2Cl_2): δ = –24.8 (s, $h_{½}$ ≈ 40 Hz).

^{13}C{^{1}H} NMR (125.8 MHz, CD_2Cl_2): δ = 148.7 (C-1), 144.3 (C-3), 126.9 (C-2).

^{29}Si NMR (99.4 MHz, CD_2Cl_2): δ = n.o.

EI-MS(+): selected peaks: *m/z* = 752.5522 ([$C_5H_5BI_5NSi$]$^{•+}$; calcd.: 752.5507), 625.6459 ([$C_5H_5BI_4NSi$]$^{+}$; calcd.: 625.6463), 546.6051 ([BI_4Si]$^{+}$; calcd.: 546.6040), 343.8591 ([$C_5H_5BI_2N$]$^{+}$; calcd.: 343.8604).

1.4.3. Characterization data of Ph_3P·I_2B–SiI_3 (2·PPh_3)

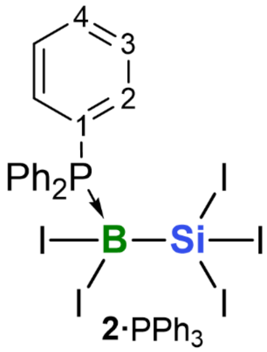

2·PPh_3

^{1}H NMR (500.2 MHz, CD_2Cl_2): δ = 8.00–7.96 (m, 6H, H-2), 7.69–7.65 (m, 3H, H-4), 7.56–7.52 (m, 6H, H-3).

^{11}B NMR (160.5 MHz, CD_2Cl_2): δ = –40.6 (d, ^{1}J(^{11}B,^{31}P) = 113 Hz).

^{13}C{^{1}H} NMR (125.8 MHz, CD_2Cl_2): δ = 135.9 (br, C-2), 133.6 (d, ^{4}J(^{13}C,^{31}P) = 2.9 Hz, C-4), 129.5 (d, ^{3}J(^{13}C,^{31}P) = 11.3 Hz, C-3), 123.7 (very br, C-1; confirmed by ^{1}H-^{13}C-HMBC NMR).

^{29}Si NMR (99.4 MHz, CD_2Cl_2): δ = n.o.

^{31}P NMR (202.5 MHz, CD_2Cl_2): δ = –7.2 (m).

EI-MS(+): selected peaks: *m/z* = 808.7992 ([$C_{18}H_{15}BI_4PSi$]$^{+}$; calcd.: 808.6955), 673.5962 ([BI_5Si]$^{•+}$; calcd.: 673.5084), 546.6757 ([BI_4Si]$^{+}$; calcd.: 546.6040), 526.9785 ([$C_{18}H_{15}BI_2P$]$^{+}$; calcd.: 526.9096).

1.4.4. Characterization data of IDipp·I_2B–SiI_3 (2·IDipp)

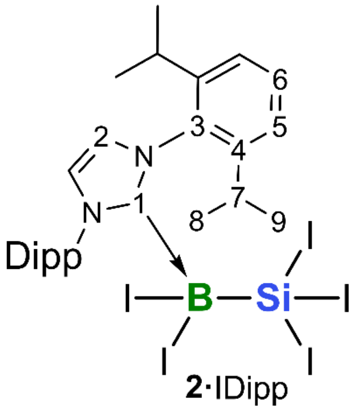

2·IDipp

^{1}H NMR (500.2 MHz, CD_2Cl_2): δ = 7.54 (t, ^3J(H,H) = 7.8 Hz, 2H, H-6), 7.34 (d, ^3J(H,H) = 7.8 Hz, 4H, H-5), 7.18 (s, 2H, H-2), 2.89 (sept, ^3J(H,H) = 6.8 Hz, 4H, H-7), 1.47 (d, ^3J(H,H) = 6.8 Hz, 12H, H-8 or 9), 1.10 (d, ^3J(H,H) = 6.8 Hz, 12H, H-8 or 9).

^{11}B NMR (160.5 MHz, CD_2Cl_2): δ = –37.1 (s, $h_{½}$ ≈ 130 Hz).

^{13}C{^{1}H} NMR (125.8 MHz, CD_2Cl_2): δ = 151.0 (C-1; detected via ^{1}H-^{13}C-HMBC NMR), 146.6 (C-4), 137.2 (C-3), 132.2 (C-6), 127.9 (C-2), 125.8 (C-5), 29.7 (C-7), 26.0 (C-8 or 9), 23.9 (C-8 or 9).

^{29}Si NMR (99.4 MHz, CD_2Cl_2): δ = n.o.

EI-MS(+): selected peaks: m/z = 934.8912 ($[C_{27}H_{36}BI_4N_2Si]^+$; calcd.: 934.8924), 653.1059 ($[C_{27}H_{36}BI_2N_2]^+$; calcd.: 653.1065).

1.4.5. Overview of ^{11}B and ^{29}Si NMR shifts of Do·I_2B–SiI_3 (2·Do)

Table S2: Overview of ^{11}B and ^{29}Si NMR shifts of Do·I_2B–SiI_3 (**2**·Do; Do = SMe_2, Py, PPh_3, IDipp).

	^{11}B NMR shift	^{29}Si NMR shift
Me_2S·I_2B–SiI_3 (**2**·SMe_2)	–31.8 ppm	n.o.
Py·I_2B–SiI_3 (**2**·Py)	–24.8 ppm	n.o.
Ph_3P·I_2B–SiI_3 (**2**·PPh_3)	–40.6 ppm	n.o.
IDipp·I_2B–SiI_3 (**2**·IDipp)	–37.1 ppm	n.o.

1.5. Synthesis of $Cl_2B–SiCl_3$ (3)

Method A: Neat [Et_4N][**1**] (2.70 g, 2.90 mmol, 1.00 eq.) and neat $GaCl_3$ (1.07 g, 6.09 mmol, 2.10 eq.) were added to a vessel, which was subsequently closed and stirred at rt for 1 h. During this period, the mixture was intermittently heated using a heatgun set to 80 °C. The formation of a liquid product was observed immediately upon contact between the two solids. The mixture was frozen using liquid N_2, and the vessel was evacuated. Upon warming to room temperature, the volatile product was transferred under static reduced pressure into a second vessel frozen in liquid N_2. Silylborane **3** was obtained as colorless liquid (ρ = 1.63 g/mL, 370 µL, 603 mg, 2.79 mmol, 96%).

Method B: [Et_4N][**1**] (8.64 g, 9.28 mmol, 1.00 eq.) and $GaCl_3$ (3.43 g, 19.5 mmol, 2.10 eq.) were suspended in *o*DFB (20 mL) and stirred at rt for 15 min. The mixture was frozen using liquid N_2, and the vessel was evacuated. Upon warming to room temperature, the solution was transferred under reduced pressure into a second vessel frozen in liquid N_2. Silylborane **3** was obtained as a solution in *o*DFB (approx. 100 mg/mL) for use in further syntheses.

Note: *o*DFB was used in place of CH_2Cl_2, as the latter is not inert toward compound **3**.

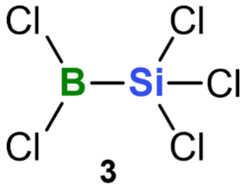

3

^{11}B NMR (96.3 MHz, no solvent): δ = 63.7 (s, $h_{½}$ ≈ 45 Hz).

^{29}Si NMR (79.5 MHz, no solvent): δ = –8.2 (q, $^1J(^{29}Si,^{11}B)$ = approx. 200 Hz).

EI-MS(+): selected peaks: m/z = 169.8823 ($[Cl_4Si]^{•+}$; calcd.: 169.8493), 132.9097 ($[Cl_3Si]^+$; calcd.: 132.8835), 115.9449 ($[BCl_3]^{•+}$; calcd.: 115.9157), 97.9352 ($[Cl_2Si]^{•+}$; calcd.: 97.9146), 80.9641 ($[BCl_2]^+$; calcd.: 80.9468), 62.9604 ($[ClSi]^+$; calcd.: 62.9458).

EA (%): Calculated for BCl_5Si [216.16]: B 5.00, Cl 82.01, Si 12.99; found: B 4.58, Cl 81.5, Si 14.0.

1.6. Synthesis of $(I_2B–SiI_3)_2·SiI_2$ (4)

Method A: Neat solid BI_3 (1.09 g, 2.78 mmol, 2.00 eq.) was added to a solution of **3** (300 mg, 1.39 mmol, 1.00 eq.) in *o*DFB (3 mL) with stirring at rt. The reaction mixture adopted a pale-yellow color, and a pale-yellow precipitate formed. The solid was isolated by filtration, rinsed repeatedly with *o*DFB (3x5 mL), and dried in vacuo. **4** was obtained as a pale-yellow solid (711 mg, 0.44 mmol, 95%).

Method B: A solution of **3** (50 mg, 231 µmol, 1.00 eq.) in *o*DFB (1 mL) was layered in an NMR tube with neat *o*DFB (1 mL) and a solution of BI_3 (181 mg, 462 µmol, 2.00 eq.) in *o*DFB (1 mL). After 1 d, pale-yellow crystals had formed. The crystals were isolated by filtration, rinsed repeatedly with *o*DFB (3x0.5 mL), and dried in vacuo. **4** was obtained as pale-yellow crystals suitable for X-ray diffraction (85.0 mg, 52 µmol, 68%).

Note: The yields were calculated based on the assumption that the formation of **4** requires 3 eq. of **3**.

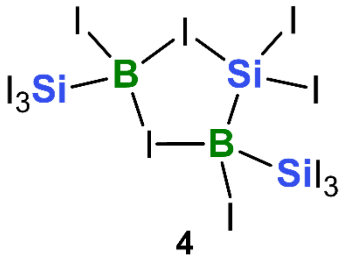

4

Solid-state ^{11}B NMR (192.6 MHz, MAS, spinning rate: 12 kHz): δ = –36 (n.r., $h_{½}$ ≈ 1670 Hz).

Solid-state ^{29}Si NMR (119.2 MHz, MAS, spinning rate: 10 kHz): δ = –108 (n.r., $h_{½}$ ≈ 1720 Hz).

Note: Due to solubility issues in all inert solvents, solid-state NMR spectra of the solid product obtained via Method A were recorded. The solid-state ^{11}B NMR spectrum shows a signal at –36 ppm (Figure S26), likely corresponding to **4**; the two resonances of the chemically inequivalent ^{11}B nuclei are not resolved. Additional minor signals observed at 47 ppm and –12 ppm (Figure S26) are assigned to BCl_3 and BI_3, respectively.[S11]

1.7. Synthesis of $[Et_4N][Cl_3B–SiCl_3]$

A solution of **3** (100 mg, 463 µmol, 1.00 eq.) in *o*DFB (1 mL) was treated with $[Et_4N]Cl$ (76.7 mg, 463 µmol, 1.00 eq.). The solution was stirred at rt for 15 min, after which time the stir bar was removed. The solution was allowed to evaporate slowly at rt, yielding colorless crystals, which were rinsed with *o*DFB (0.5 mL) and dried in vacuo. $[Et_4N][Cl_3B–SiCl_3]$ was obtained as colorless crystals suitable for X-ray diffraction (163 mg, 427 µmol, 92%).

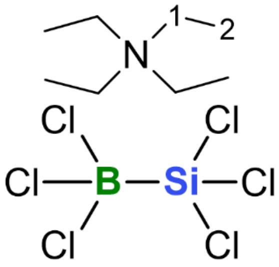

$[Et_4N][Cl_3B–SiCl_3]$

^{1}H NMR (500.2 MHz, CD_2Cl_2): δ = 3.24 (q, ^3J(H,H) = 7.3 Hz, 8H, H-1), 1.35 (tt, ^3J(H,H) = 7.3 Hz, ^3J(H,^{14}N) = 1.9 Hz, 12H, H-2).

^{11}B NMR (160.5 MHz, CD_2Cl_2): δ = 3.0 (s, $h_{½}$ ≈ 26 Hz).

^{13}C{^{1}H} NMR (125.8 MHz, CD_2Cl_2): δ = 53.26 (t, ^1J(^{13}C,^{14}N) = 3.1 Hz, C-1), 7.91 (C-2).

^{29}Si NMR (99.4 MHz, CD_2Cl_2): δ = n.o.

EI-MS(+): selected peaks: *m/z* = 180.8921 ($[BCl_4Si]^+$; calcd.: 180.8589), 169.8834 ($[Cl_4Si]^{\bullet+}$; calcd.: 169.8493), 130.1870 ($[C_8H_{20}N]^+$; calcd.: 130.1596).

Note: The chemical shift value of the ^{11}B NMR signal observed for $[Et_4N][Cl_3B–SiCl_3]$ matches the value reported for $[((TMS)_2N)SiCl_2B(Me_5C_5)][Cl_3B–SiCl_3]$.[S12]

1.8. Synthesis of the adducts Do·Cl_2B–$SiCl_3$ (**3**·Do; Do = SMe_2, Py, PPh_3, IDipp)

General procedure: A solution of **3** (100 mg, 463 µmol, 1.00 eq.) in *o*DFB (1 mL) was treated with the respective Lewis basic donor (Do; cf. Table S3, 1.00 eq.). The solution was stirred at rt for 15 min, after which time the stir bar was removed. The solution was allowed to evaporate slowly at rt, yielding colorless crystals, which were rinsed with *o*DFB (0.5 mL) and dried in vacuo. **3**·Do were obtained as colorless crystals suitable for X-ray diffraction (cf. Table S3, 89–94%).

Table S3: Quantities of Do used in the synthesis of **3**·Do.

	Do	yield of **3**·Do
Do = SMe_2	34.0 µL, 28.9 mg, 465 µmol	121 mg, 435 µmol, 94%
Do = Py	37.3 µL, 36.6 mg, 463 µmol	130 mg, 440 µmol, 95%
Do = PPh_3	121 mg, 461 µmol	201 mg, 420 µmol, 91%
Do = IDipp	180 mg, 463 µmol	249 mg, 412 µmol, 89%

1.8.1. Characterization data of Me_2S·Cl_2B–$SiCl_3$ (**3**·SMe_2)

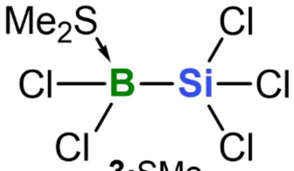

3·SMe_2

^{1}H NMR (500.2 MHz, CD_2Cl_2): δ = 2.55 (s, 6H, Me_2S).

^{11}B NMR (160.5 MHz, CD_2Cl_2): δ = 1.4 (s, $h_{½}$ ≈ 26 Hz).

^{13}C{^{1}H} NMR (125.8 MHz, CD_2Cl_2): δ = 20.5 (Me_2S).

^{29}Si NMR (99.4 MHz, CD_2Cl_2): δ = n.o.

EI-MS(+): selected peaks: m/z = 242.9237 ($[C_2H_6BCl_4SSi]^+$; calcd.: 242.8779), 215.8689 ($[BCl_5Si]^{\bullet+}$; calcd.: 215.8277), 180.8925 ($[BCl_4Si]^+$; calcd.: 180.8589).

1.8.2. Characterization data of Py·Cl_2B–$SiCl_3$ (3·Py)

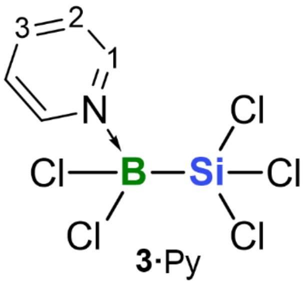

^{1}H NMR (500.2 MHz, CD_2Cl_2): δ = 9.20 (m, 2H, H-1), 8.33 (tt, ^3J(H,H) = 7.8 Hz, ^4J(H,H) = 1.5 Hz, 1H, H-3), 7.90 (m, 2H, H-2).

^{11}B NMR (160.5 MHz, CD_2Cl_2): δ = 3.7 (s, $h_{½}$ ≈ 28 Hz).

^{13}C{^{1}H} NMR (125.8 MHz, CD_2Cl_2): δ = 145.6 (C-1), 144.4 (C-3), 127.1 (C-2).

^{29}Si NMR (99.4 MHz, CD_2Cl_2): δ = n.o.

EI-MS(+): selected peaks: m/z = 259.9433 ($[C_5H_5BCl_4NSi]^+$; calcd.: 259.9012), 160.0151 ($[C_5H_5BCl_2N]^+$; calcd.: 159.9891).

1.8.3. Characterization data of Ph_3P·Cl_2B–$SiCl_3$ (3·PPh_3)

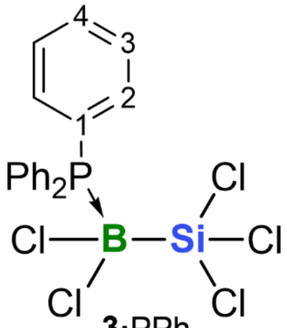

^{1}H NMR (500.2 MHz, CD_2Cl_2): δ = 7.79–7.75 (m, 6H, H-2), 7.70–7.66 (m, 3H, H-4), 7.57–7.53 (m, 6H, H-3).

^{11}B NMR (160.5 MHz, CD_2Cl_2): δ = –3.4 (d, $^1J(^{11}B,^{31}P)$ = 116 Hz).

^{13}C{^{1}H} NMR (125.8 MHz, CD_2Cl_2): δ = 135.2 (d, $^2J(^{13}C,^{31}P)$ = 8.7 Hz, C-2), 133.5 (d, $^4J(^{13}C,^{31}P)$ = 2.8 Hz, C-4), 129.7 (d, $^3J(^{13}C,^{31}P)$ = 11.1 Hz, C-3), 122.5 (d, $^1J(^{13}C,^{31}P)$ = 66.8 Hz, C-1).

^{29}Si NMR (99.4 MHz, CD_2Cl_2): δ = n.o.

^{31}P NMR (202.5 MHz, CD_2Cl_2): δ = 2.1 (m).

EI-MS(+): selected peaks: m/z = 343.0789 ($[C_{18}H_{15}BCl_2P]^+$; calcd.: 343.0383), 262.1262 ($[C_{18}H_{15}P]^{\bullet+}$; calcd.: 262.0911), 215.8597 ($[BCl_5Si]^{\bullet+}$; calcd.: 215.8277).

1.8.4. Characterization data of IDipp·Cl_2B–$SiCl_3$ (3·IDipp)

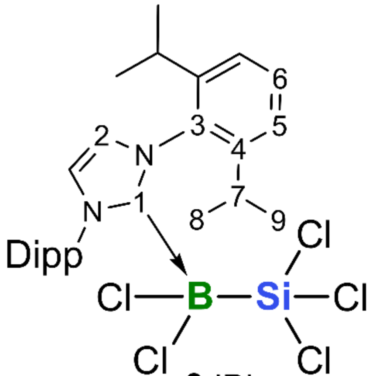

3·IDipp

^{1}H NMR (500.2 MHz, CD_2Cl_2): δ = 7.53 (t, ^{3}J(H,H) = 7.8 Hz, 2H, H-6), 7.33 (d, ^{3}J(H,H) = 7.8 Hz, 4H, H-5), 7.17 (s, 2H, H-2), 2.68 (sept, ^{3}J(H,H) = 6.8 Hz, 4H, H-7), 1.39 (d, ^{3}J(H,H) = 6.8 Hz, 12H, H-8 or 9), 1.13 (d, ^{3}J(H,H) = 6.8 Hz, 12H, H-8 or 9).

^{11}B NMR (160.5 MHz, CD_2Cl_2): δ = –4.4 (s, $h_{½}$ ≈ 90 Hz).

^{13}C{^{1}H} NMR (125.8 MHz, CD_2Cl_2): δ = 156.8 (C-1; detected via ^{1}H-^{13}C-HMBC NMR), 146.0 (C-4), 134.9 (C-3), 131.7 (C-6), 126.8 (C-2), 124.9 (C-5), 29.6 (C-7), 26.1 (C-8 or 9), 22.9 (C-8 or 9).

^{29}Si NMR (99.4 MHz, CD_2Cl_2): δ = n.o.

EI-MS(+): selected peaks: *m/z* = 569.2046 ([$C_{27}H_{36}BCl_4N_2Si$]$^{+}$; calcd.: 569.1475), 469.2870 ([$C_{27}H_{36}BCl_2N_2$]$^{+}$; calcd.: 469.2352).

1.8.5. Overview of ^{11}B and ^{29}Si NMR shifts of Do·Cl_2B–$SiCl_3$ (3·Do) and Cl_2B–$SiCl_3$ (3)

Table S4: Overview of ^{11}B and ^{29}Si NMR shifts of Do·I_2B–SiI_3 (**3**·Do; Do = SMe_2, Py, PPh_3, IDipp) and Cl_2B–$SiCl_3$ (**3**).

	^{11}B NMR shift	^{29}Si NMR shift
Cl_2B–$SiCl_3$ (**3**)	63.7 ppm	–8.2 ppm
Me_2S·Cl_2B–$SiCl_3$ (**3**·SMe_2)	1.4 ppm	n.o.
Py·Cl_2B–$SiCl_3$ (**3**·Py)	3.7 ppm	n.o.
Ph_3P·Cl_2B–$SiCl_3$ (**3**·PPh_3)	–3.4 ppm	n.o.
IDipp·Cl_2B–$SiCl_3$ (**3**·IDipp)	–4.4 ppm	n.o.

1.9. Synthesis of $BI_3 \cdot PPh_3$

A solution of BI_3 (100 mg, 255 µmol, 1.00 eq.) in CH_2Cl_2 (1 mL) was treated with a solution of triphenylphosphine (67.0 mg, 255 µmol, 1.00 eq.) in CH_2Cl_2 (1 mL). The solution was allowed to evaporate slowly at rt, yielding colorless crystals, which were rinsed with CH_2Cl_2 (2x0.5 mL). $BI_3 \cdot PPh_3$ was obtained as colorless crystals (150 mg, 229 µmol, 90%).

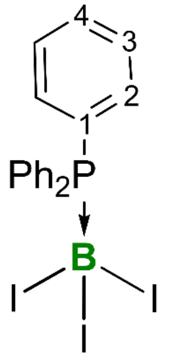

$BI_3 \cdot PPh_3$

^{1}H NMR (500.2 MHz, CD_2Cl_2): δ = 7.94–7.90 (m, 6H, H-2), 7.72–7.68 (m, 3H, H-4), 7.56–7.53 (m, 6H, H-3).

^{11}B NMR (160.5 MHz, CD_2Cl_2): δ = –72.1 (d, $^{1}J(^{11}B, ^{31}P)$ = 125 Hz).

^{13}C{^{1}H} NMR (125.8 MHz, CD_2Cl_2): δ = 135.1 (d, $^{2}J(^{13}C,^{31}P)$ = 7.7 Hz, C-2), 133.4 (d, $^{4}J(^{13}C,^{31}P)$ = 3.0 Hz, C-4), 129.5 (d, $^{3}J(^{13}C,^{31}P)$ = 11.2 Hz, C-3), 124.1 (d, $^{1}J(^{13}C,^{31}P)$ = 70.3 Hz, C-1).

^{29}Si NMR (99.4 MHz, CD_2Cl_2): δ = n.o.

^{31}P NMR (202.5 MHz, CD_2Cl_2): δ = –15.1 (q, $^{1}J(^{31}P,^{11}B)$ = 125 Hz).

EI-MS(+): selected peaks: m/z = 526.9074 ($[C_{18}H_{15}BI_2P]^+$; calcd.: 526.9096), 391.7225 ($[BI_3]^{\bullet+}$; calcd.: 391.7225), 262.0937 ($[C_{18}H_{15}P]^{\bullet+}$; calcd.: 262.0911).

1.10. Synthesis of BI_3·IDipp

A solution of BI_3 (100 mg, 255 µmol, 1.00 eq.) in CH_2Cl_2 (1 mL) was treated with a solution of 1,3-bis(2,6-diisopropylphenyl)-1,3-dihydro-*2H*-imidazol-2-ylidene (IDipp) (99.3 mg, 255 µmol, 1.00 eq.) in CH_2Cl_2 (1 mL). The solution was allowed to evaporate slowly at rt, yielding colorless crystals, which were rinsed with CH_2Cl_2 (2x0.5 mL). BI_3·IDipp was obtained as colorless crystals (170 mg, 218 µmol, 85%).

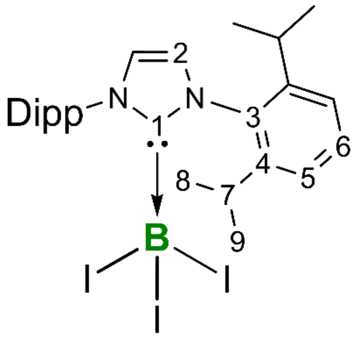

BI_3·IDipp

^{1}H NMR (500.2 MHz, CD_2Cl_2): δ = 7.54 (t, ^3J(H,H) = 7.8 Hz, 2H, H-6), 7.32 (d, ^3J(H,H) = 7.8 Hz, 4H, H-5), 7.20 (s, 2H, H-2), 2.79 (sept, ^3J(H,H) = 6.7 Hz, 4H, H-7), 1.45 (d, ^3J(H,H) = 6.7 Hz, 12H, H-8 or 9), 1.14 (d, ^3J(H,H) = 6.9 Hz, 12H, H-8 or 9).

^{11}B NMR (160.5 MHz, CD_2Cl_2): δ = –77.3 (s, $h_{½}$ ≈ 55 Hz).

^{13}C{^{1}H} NMR (125.8 MHz, CD_2Cl_2): δ = 148.1 (C-1; detected via ^{1}H-^{13}C-HMBC NMR), 146.5 (C-4), 135.9 (C-3), 131.7 (C-6), 126.1 (C-2), 124.9 (C-5), 30.0 (C-7), 26.1 (C-8 or 9), 23.1 (C-8 or 9).

^{29}Si NMR (99.4 MHz, CD_2Cl_2): δ = n.o.

EI-MS(+): selected peaks: m/z = 653.0910 ($[C_{27}H_{36}BI_2N_2]^+$; calcd.: 653.1065).

1.11. Reactivities of the silylborane adducts Do·I_2B–SiI_3 (2·Do) and Do·Cl_2B–$SiCl_3$ (3·Do)

1.11.1. Reaction of Me_2S·I_2B–SiI_3 (2·SMe_2) with ethylene to furnish Me_2S·I_2B–C_2H_4–SiI_3 (5·SMe_2)

Method A: In an NMR tube, **2**·SMe_2 (50 mg, 68 µmol) was suspended in CD_2Cl_2 (0.5 mL). The NMR tube was cooled with liquid N_2 to –196 °C, evacuated, closed, and allowed to reach rt. Afterwards, the NMR tube was filled with an excess of dried ethylene, closed, cooled again with liquid N_2 to –196 °C, and flame-sealed. The flame-sealed NMR tube was heated to 80 °C for 6 d. The NMR tube was opened, and the reaction solution allowed to evaporate slowly at rt, yielding colorless crystals, which were rinsed with *o*DFB (2x0.1 mL) and dried in vacuo. **5**·SMe_2 was obtained as colorless crystals suitable for X-ray diffraction (51 mg, 67 µmol, 98%).

Method B: In an NMR tube, **2**·SMe_2 (50 mg, 68 µmol, 1.0 eq.) was suspended in CD_2Cl_2 (0.5 mL); neat solid BI_3 (2.7 mg, 6.9 µmol, 0.1 eq.) was added to the mixture. The NMR tube was cooled with liquid N_2 to –196 °C, evacuated, closed, and allowed to reach rt. Afterwards, the NMR tube was filled with an excess of dried ethylene, closed, cooled again with liquid N_2 to –196 °C, and flame-sealed. The flame-sealed NMR tube was stored at rt for 12 h. The NMR tube was opened, and the reaction solution was allowed to evaporate slowly at rt, affording colorless crystals, which were rinsed with *o*DFB (2x0.1 mL) and dried in vacuo. **5**·SMe_2 was obtained as colorless crystals suitable for X-ray diffraction (44 mg, 58 µmol, 85%).

Note: Employing Li[**1**], **2**/LiI in place of BI_3 as the promoter did not prove beneficial.

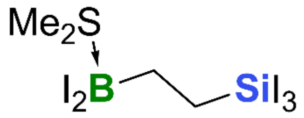

5·SMe_2

^{1}H NMR (500.2 MHz, CD_2Cl_2): δ = 2.51 (s, 6H, Me_2S), 2.39–2.36 (m, 2H, Si–CH_2–), 1.44–1.41 (m, 2H, B–CH_2–).

^{11}B NMR (160.5 MHz, CD_2Cl_2): δ = –18.9 (s, $h_{½}$ ≈ 115 Hz).

^{13}C{^{1}H} NMR (125.8 MHz, CD_2Cl_2): δ = 32.2 (Si–CH$_2$–), 24.8 (Me_2S), 22.8 (B–CH$_2$–; detected via ^{1}H-^{13}C-HSQC/-HMBC NMR experiments).

^{29}Si NMR (99.4 MHz, CD_2Cl_2): δ = –115.1.

1.11.2. Reaction of Py·I_2B–SiI_3 (2·Py) with ethylene to give Py·I_2B–C_2H_4–SiI_3 (5·Py)

In an NMR tube, **2**·Py (50 mg, 66 µmol) was suspended in *o*DFB (0.5 mL). The NMR tube was cooled with liquid N_2 to –196 °C, evacuated, closed, and allowed to reach rt. Afterwards, the NMR tube was filled with an excess of dried ethylene, closed, cooled with liquid N_2 to –196 °C again, and flame-sealed. The flame-sealed NMR tube was heated to 120 °C for 20 d. The NMR tube was opened, and the reaction solution was allowed to evaporate slowly at rt, yielding colorless crystals, which were rinsed with *o*DFB (2x0.1 mL) and dried in vacuo. **5**·Py was obtained as colorless crystals suitable for X-ray diffraction (50 mg, 64 mmol, 97%).

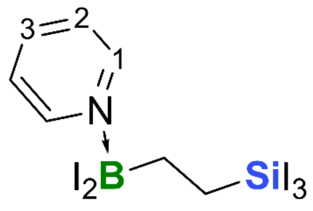

5·Py

^{1}H NMR (500.2 MHz, CD_2Cl_2): δ = 9.43 (d, ^3J(H,H) = 6.4 Hz, 2H, H-1), 8.27 (tt, ^3J(H,H) = 7.8 Hz, ^4J(H,H) = 1.4 Hz, 1H, H-3), 7.80–7.76 (m, 2H, H-2), 2.45–2.42 (m, 2H, Si–CH_2–), 1.63–1.60 (m, 2H, B–CH_2–).

^{11}B NMR (160.5 MHz, CD_2Cl_2): δ = –14.0 (s, $h_{½}$ ≈ 140 Hz).

^{13}C{^{1}H} NMR (125.8 MHz, CD_2Cl_2): δ = 147.2 (C-1), 143.9 (C-3), 126.9 (C-2), 32.6 (Si–*C*H_2–), 27.4 (B–*C*H_2–; detected via ^{1}H-^{13}C-HSQC/-HMBC NMR experiments).

^{29}Si NMR (99.4 MHz, CD_2Cl_2): δ = –114.8.

EI-MS(+): selected peaks: *m*/*z* = 574.6688 ([$C_2H_4BI_4Si$]$^+$; calcd.: 574.6353).

1.11.3. Reaction of $Ph_3P{\cdot}I_2B–SiI_3$ (2·PPh_3) with ethylene

In an NMR tube, **2**·PPh_3 (50 mg, 53 µmol) was suspended in *o*DFB (0.5 mL). The NMR tube was cooled with liquid N_2 to –196 °C, evacuated, closed, and allowed to reach rt. Afterwards, the NMR tube was filled with an excess of dried ethylene, closed, cooled with liquid N_2 to –196 °C again, and flame-sealed. The flame-sealed NMR tube was heated to 120 °C for 20 d.

Note: The ^{11}B NMR spectrum of the reaction solution showed the signal of residual starting material at –40.4 ppm (blue, Figure S60) and of [Et_4N][**1**] at –59.7 ppm (green, Figure S60; quantitative separation of [Et_4N][**1**] from **2**·PPh_3 during the large-scale synthesis of the latter was not achieved due to their similarly poor solubility). The reaction afforded two new boron-containing species. One of them, corresponding to the signal at –72.1 ppm (red, Figure S60), has been identified as $BI_3{\cdot}PPh_3$ (an authentic sample of $BI_3{\cdot}PPh_3$ was synthesized and characterized for comparison; cf. chapter 1.9). The second species gave rise to a broad signal at –25.3 ppm (orange, Figure S60) and is assumed to be the aimed-for ethylene silaboration product. This assumption is supported by the presence of a set of signals in the alkyl region of the ^{1}H NMR spectrum (orange, Figure S59), which is diagnostic for a $Do{\cdot}I_2B–(C_2H_4)–SiI_3$ moiety. Furthermore, upon reaction of **5**·SMe_2 with PPh_3, an ^{11}B NMR signal appears at –24.8 ppm (orange, Figure S60), consistent with the above assignment of the –25.3 ppm resonance to **5**·PPh_3.

1.11.4. Reaction of $IDipp{\cdot}I_2B–SiI_3$ (2·IDipp) with ethylene

In an NMR tube, **2**·IDipp (50 mg, 47 µmol) was suspended in *o*DFB (0.5 mL). The NMR tube was cooled with liquid N_2 to –196 °C, evacuated, closed, and allowed to reach rt. Afterwards, the NMR tube was filled with an excess of dried ethylene, closed, cooled with liquid N_2 to –196 °C again, and flame-sealed. The flame-sealed NMR tube was heated to 100 °C for 6 d.

Note: The ^{11}B NMR spectrum of the reaction solution showed a signal for residual starting material at –36.7 ppm (blue, Figure S61) and a signal at –77.0 ppm (red, Figure S61), attributable to BI_3·IDipp (an authentic sample of BI_3·IDipp was synthesized and characterized for comparison; cf. chapter 1.10).

1.11.5. Reaction of IDipp·I_2B–SiI_3 (2·IDipp) with 2,3-dimethyl-1,3-butadiene

Method A: In an NMR tube, **2**·IDipp (50 mg, 47 µmol, 1.0 eq.) was suspended in CD_2Cl_2 (0.5 mL). Subsequently, 2,3-dimethyl-1,3-butadiene (53 µL, 39 mg, 0.47 mmol, 10 eq.) was added. The NMR tube was cooled with liquid N_2 to –196 °C, evacuated, and flame-sealed. The flame-sealed NMR tube was heated to 100 °C for 8 d.

Note: The ^{11}B NMR spectrum of the reaction solution showed a signal at –4.4 ppm (blue, Figure S62), attributable to **3**·IDipp, and a signal at 1.6 ppm, corresponding to BCl_3·IDipp (red, Figure S62).[S13] The ^{13}C NMR spectrum of the reaction solution showed signals at 128.9 ppm, 29.8 ppm, and 19.5 ppm, matching the published shift values of 1,1-dichloro-3,4-dimethyl-1-silacyclopent-3-ene (Figure S63).[S14–16] GC–MS analysis of the reaction solution revealed the following signals (Figure S1):

τ = 5.59 min, *m*/*z* = 178 ($[CD_2ClI]^+$; calcd.: 177.9);

τ = 7.35 min, *m*/*z* = 270 ($[CD_2I_2]^+$; calcd.: 269.8);

τ = 8.79 min, *m*/*z* = 180 ($[C_6H_{10}Cl_2Si]^+$; calcd.: 180.0).

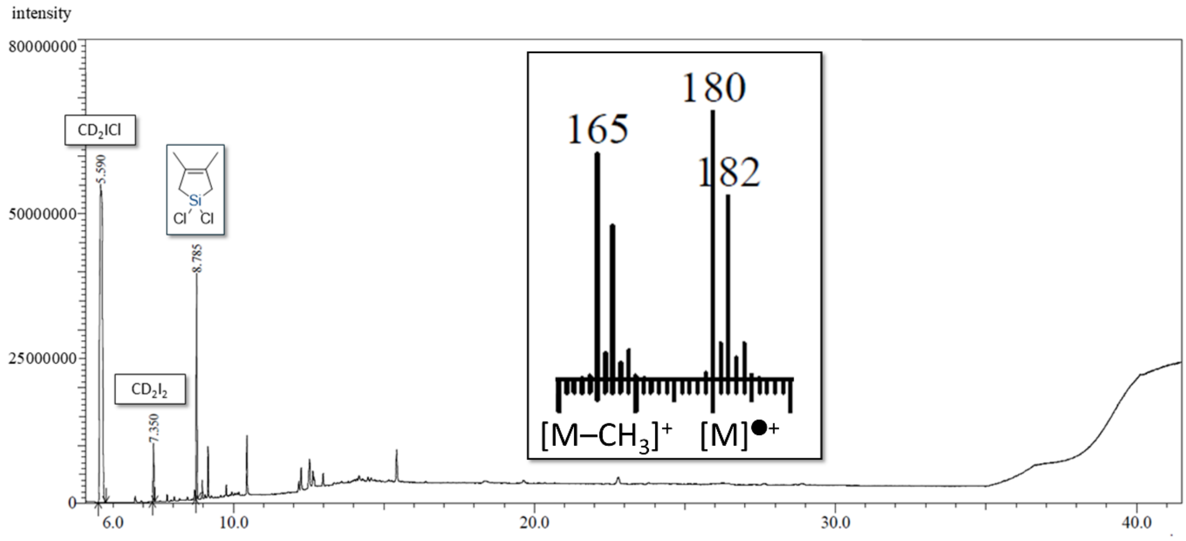

Figure S1: GC-MS chromatogram of the reaction mixture of **2**·IDipp with 2,3-dimethyl-1,3-butadiene in CD_2Cl_2, showing the compounds corresponding to the detected masses at specific retention times. The insert shows the corresponding EI mass spectrum in the region of the molecular-ion peak $[M]^{\bullet+}$.

Method B: In an NMR tube, **2**·IDipp (50 mg, 47 µmol, 1.0 eq.) was suspended in *o*DFB (0.5 mL). Subsequently, 2,3-dimethyl-1,3-butadiene (53 µL, 39 mg, 0.47 mmol, 10 eq.) was added. The NMR tube was cooled with liquid N_2 to –196 °C, evacuated, and flame-sealed. The flame-sealed NMR tube was heated to 100 °C for 10 d.

Note: The ^{11}B NMR spectrum of the reaction solution showed a signal at –76.9 ppm (Figure S64), attributable to BI_3·IDipp. X-ray diffraction allowed to identify a crystalline phase isolated from the reaction solution as BI_3·IDipp, based on unit cell parameters that matched those of an authentic sample of BI_3·IDipp (see Table S9). GC–MS analysis of the reaction solution revealed the following signals (Figure S2):

τ = 11.97 min, *m/z* = 364 ($[C_6H_{10}I_2Si]^{\bullet+}$; calcd.: 363.9);

τ = 14.56 min, *m/z* = 492 ($[C_6H_{11}I_3Si]^{\bullet+}$; calcd.: 491.8).

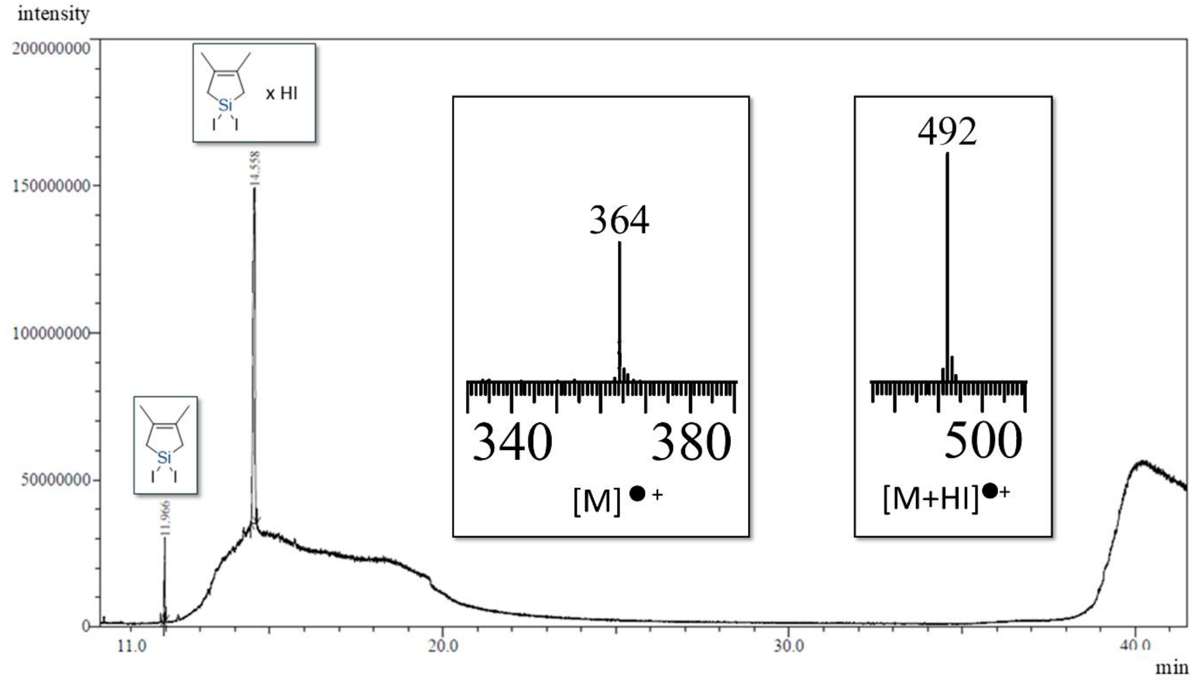

Figure S2: GC-MS chromatogram of the reaction mixture of **2**·IDipp with 2,3-dimethyl-1,3-butadiene in *o*DFB, showing the compounds corresponding to the detected masses at specific retention times. The insert shows the corresponding EI mass spectra in the regions of the molecular-ion peaks $[M]^{\bullet+}$ (left) and $[M+HI]^{\bullet+}$ (right).

1.11.6. Reaction of $Me_2S{\cdot}Cl_2B–SiCl_3$ (3·SMe₂) with ethylene

In an NMR tube, **3**·SMe_2 (25 mg, 90 µmol) was dissolved in CD_2Cl_2 (0.5 mL). The NMR tube was cooled with liquid N_2 to –196 °C, evacuated, closed, and allowed to reach rt. Afterwards, the NMR tube was filled with an excess of dried ethylene, closed, cooled with liquid N_2 to –196 °C again, and flame-sealed. The flame-sealed NMR tube was heated to 80 °C for 31 d.

Note: Neither BCl_3 nor **3** proved effective as promoters for this transformation, in contrast to the ability of BI_3 to promote the iodine-analog reaction.

Note: The ^{11}B NMR spectrum of the reaction solution showed a signal for residual starting material at 1.4 ppm (blue, Figure S66) alongside a signal at 7.4 ppm (red, Figure S66) for $BCl_3{\cdot}SMe_2$ (orange, Figure S66).[S17] A third signal at 9.9 ppm is assumed to arise from the ethylene silaboration product. This assumption is supported by a set of signals in the alkyl region of the ^1H NMR spectrum (orange, Figure S65), which is diagnostic for a $Cl_2B–(C_2H_4)–SiCl_3{\cdot}Do$ moiety. The origin of a minor signal at 3.2 ppm is unknown (purple, Figure S66).

1.11.7. Reaction of $Py{\cdot}Cl_2B–SiCl_3$ (3·Py) with ethylene

In an NMR tube, **3**·Py (25 mg, 85 µmol) was dissolved in *o*DFB (0.5 mL). The NMR tube was cooled with liquid N_2 to –196 °C, evacuated, closed, and allowed to reach rt. Afterwards, the NMR tube was filled with an excess of dried ethylene, closed, cooled with liquid N_2 to –196 °C again, and flame-sealed. The flame-sealed NMR tube was heated to 120 °C for 7 d, then to 140 °C for an additional day, and finally to 160 °C for a further day.

Note: The ^{11}B NMR spectrum of the reaction solution showed a signal for residual starting material at 3.7 ppm (blue, Figure S67) alongside a signal at 8.5 ppm for $BCl_3{\cdot}Py$ (red, Figure S67;[S18] an authentic sample of $BCl_3{\cdot}Py$ was synthesized, as no ^1H or ^{13}C NMR data are available in the literature. The recorded spectra exhibit resolved $^3J_{HB}$ and $^3J_{CB}$ couplings, which is a rare phenomenon; cf. chapter 2.25). The origin of a third signal at –3.7 ppm is unknown (purple, Figure S67).

1.11.8. Reaction of $Ph_3P·Cl_2B–SiCl_3$ (3·PPh_3) with ethylene

In an NMR tube, **3**·PPh_3 (25 mg, 52 µmol) was dissolved in *o*DFB (0.5 mL). The NMR tube was cooled with liquid N_2 to –196 °C, evacuated, closed, and allowed to reach rt. Afterwards, the NMR tube was filled with an excess of dried ethylene, closed, cooled with liquid N_2 to –196 °C again, and flame-sealed. The flame-sealed NMR tube was heated to 120 °C for 17 d.

Note: The ^{11}B NMR spectrum of the reaction solution showed a signal for residual starting material at –3.4 ppm (blue, Figure S71) alongside a signal at 4.0 ppm for $BCl_3·PPh_3$ (red, Figure S71).[S19,20]

1.11.9. Reaction of IDipp·$Cl_2B–SiCl_3$ (3·IDipp) with ethylene

In an NMR tube, **3**·IDipp (25 mg, 41 µmol) was dissolved in *o*DFB (0.5 mL). The NMR tube was cooled with liquid N_2 to –196 °C, evacuated, closed, and allowed to reach rt. Afterwards, the NMR tube was filled with an excess of dried ethylene, closed, cooled with liquid N_2 to –196 °C again, and flame-sealed. The flame-sealed NMR tube was heated to 120 °C for 7 d, then to 140 °C for 1 d, and finally to 160 °C for 1 d.

Note: The ^{11}B NMR spectrum of the reaction solution showed a signal for residual starting material at –4.3 ppm (blue, Figure S72) alongside a signal for BCl_3·IDipp at 1.8 ppm (red, Figure S72).[S13]

1.12. Stability of the silylborane adducts Do·$I_2B–SiI_3$ (2·Do) under sunlight exposure

The periodinated compounds **2**·Do exhibit remarkable photostability, showing no signs of decomposition under sunlight exposure. In stark contrast, BI_3 is sensitive to sunlight, which is visually apparent through the progressive darkening of the sample caused by iodine release (Figure S3).[S9,21,22]

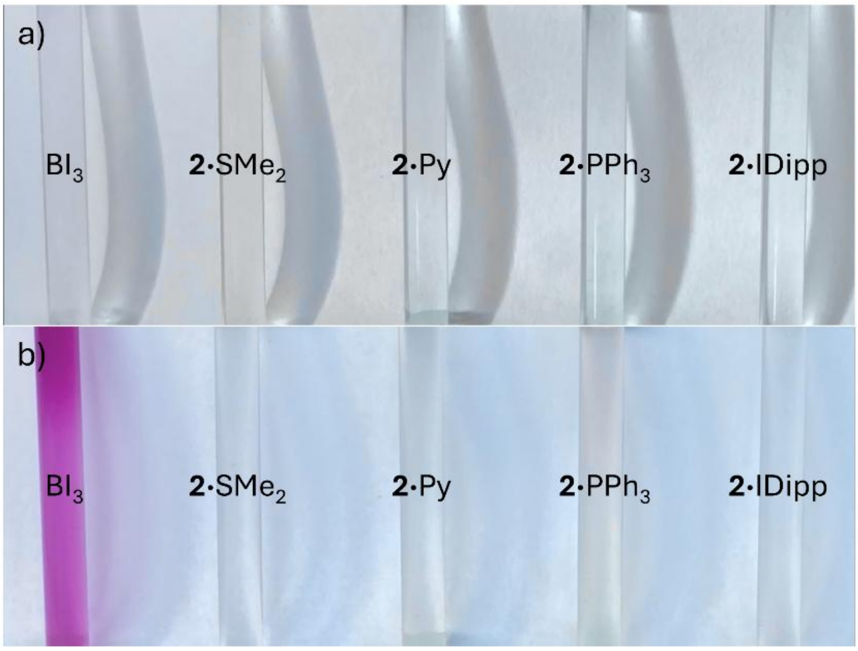

Figure S3: Solutions of BI_3 (NMR tube; C_6H_6) and **2**·Do (NMR tube; CD_2Cl_2): (a) freshly prepared; (b) after exposure to direct sunlight for 1 h at rt.

1.13. Reaction of 2 with cyclohexene to give $(I_2B–)(I_3Si–)C_6H_{10}$ (7)

$[Et_4N]$[**1**] (500 mg, 537 µmol, 1.00 eq.) and $Li[Al(OC(CF_3)_3)_4]$ (576 mg, 591 µmol, 1.10 eq.) were suspended in *o*DFB (10 mL), and cyclohexene (544 µL, 441 mg, 5.37 mmol, 10.0 eq.) was added with stirring at rt. After 15 min of stirring at rt, all volatiles were removed under reduced pressure. The solid residue was extracted into C_6H_6 (3x2 mL). The colorless extract was allowed to evaporate slowly at rt, furnishing colorless crystals, which were rinsed with C_6H_6 (0.5 mL) and dried in vacuo. **7** was obtained as colorless crystals (β-polymorph) suitable for X-ray diffraction (400 mg, 529 µmol, 99%).

Note: Single crystals of the α-polymorph of **7** were obtained by slow evaporation of a solution of **7** in *o*DFB at rt.

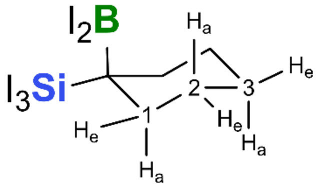

7

^{1}H NMR (500.2 MHz, C_6D_6): δ = 2.80 (dm, ^2J(H,H) ≈ 13.2 Hz, 2 x ^3J(H,H): n.r., 2H, H_e-1), 1.85 (ddd, ^2J(H,H) ≈ 13.2 Hz, ^3J(H,H) ≈ 13.2 Hz, 3.0 Hz, 2H, H_a-1), 1.54–1.48 (m, 2H, H_e-2), 1.41 (dtt, ^2J(H,H) ≈ 13 Hz, ^3J(H,H) ≈ 13 Hz, 3.5 Hz, 2H, H_a-2), 1.27–1.21 (m, 1H, H_e-3), 0.90 (dtt, ^2J(H,H) ≈ 13.0 Hz, ^3J(H,H) ≈ 13.0 Hz, 4.1 Hz, 1H, H_a-3).

^{11}B NMR (160.5 MHz, C_6D_6): δ = 53.5 (s, $h_{½}$ ≈ 260 Hz).

^{13}C{^{1}H} NMR (125.8 MHz, C_6D_6): δ = 49.5 (*C*(–BI_2)(–SiI_3); detected via ^{1}H-^{13}C-HSQC/-HMBC NMR experiments), 35.5 (C-1), 25.9 (C-2), 25.3 (C-3).

^{29}Si NMR (99.4 MHz, C_6D_6): δ = –122.8.

EI-MS(+): selected peaks: *m*/*z* = 628.6754 ($[C_6H_{10}BI_4Si]^+$; calcd.: 628.6823), 408.8766 ($[I_3Si]^+$; calcd.: 408.6903), 81.0704 ($[C_6H_9]^+$; calcd.: 81.0704).

1.14. Reaction of $Me_2S·I_2B–SiI_3$ (2·SMe_2) with cyclohexene

In an NMR tube, **2**·SMe_2 (50 mg, 68 µmol, 1.0 eq.) was suspended in *o*DFB (0.5 mL). Subsequently, cyclohexene (69 µL, 56 mg, 0.68 mmol, 10 eq.) was added. The NMR tube was cooled with liquid N_2 to –196 °C, evacuated, and flame-sealed. The flame-sealed NMR tube was heated to 120 °C for 24 d.

Note: The ^{11}B NMR spectrum of the reaction solution showed a signal at –11.6 ppm (Figure S77), which is assumed to be corresponding to the aimed-for cyclohexene 1,1-silaboration product. This assumption is supported by the presence of an ^{11}B NMR signal at –10.2 ppm (Figure S77), in the ^{11}B NMR spectrum of the reaction mixture of **7** with SMe_2.

1.15. Reaction of BI_3·SMe_2 with ethylene to give Me_2S·I_2B–C_2H_4–I

In an NMR tube, BI_3·SMe_2 (20 mg, 44 µmol, 1.0 eq.) was dissolved in CD_2Cl_2 (0.5 mL). Neat BI_3 (1.7 mg, 4.4 µmol, 0.1 eq.) was added to the solution at rt. The NMR tube was cooled with liquid N_2 to –196 °C, evacuated, closed, and allowed to reach rt. Afterwards, the NMR tube was filled with an excess of dried ethylene, closed, cooled with liquid N_2 to –196 °C again, and flame-sealed. The flame-sealed NMR tube was stored at rt for 5 d. The NMR tube was opened, and the reaction solution was allowed to evaporate slowly at rt, furnishing colorless crystals, which were rinsed with CH_2Cl_2 (2x0.1 mL) and dried in vacuo. Me_2S·I_2B–C_2H_4–I was obtained as colorless crystals suitable for X-ray diffraction (20 mg, 42 µmol, 95%).

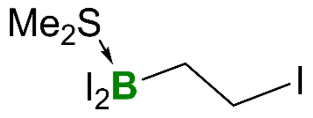

Me_2S·I_2B–C_2H_4–I

^{1}H NMR (500.2 MHz, CD_2Cl_2): δ = 3.50–3.47 (m, 2H, I–C*H*$_2$–), 2.48 (s, 6H, Me_2S), 2.04–2.01 (m, 2H, B–C*H*$_2$–).

^{11}B NMR (160.5 MHz, CD_2Cl_2): δ = –19.4 (s, $h_{½}$ ≈ 70 Hz).

^{13}C{^{1}H} NMR (125.8 MHz, CD_2Cl_2): δ = 34.9 (B–*C*H$_2$–; detected via ^{1}H-^{13}C-HSQC/-HMBC NMR experiments), 24.5 (Me_2S), 6.8 (I–*C*H$_2$–).

EI-MS(+): selected peaks: *m/z* = 419.7509 ($[C_2H_4BI_3]^{•+}$; calcd.: 419.7539), 391.7206 ($[BI_3]^{•+}$; calcd.: 391.7225), 292.8487 ($[C_2H_4BI_2]^{+}$; calcd.: 292.8494), 264.8185 ($[BI_2]^{+}$; calcd.: 264.8180).

Note: Traces of the starting material, BI_3·SMe_2, were found to co-crystallize with the product and remain detectable in the NMR spectra (blue, Figure S78 and Figure S79), comprising approximately 5% of the sample as estimated from the integration of the NMR signals.

1.16. Reaction of $Me_2S{\cdot}Cl_2B–SiCl_3$ (3·SMe$_2$) with phenylacetylene to give 8

A solution of **3**·SMe_2 (200 mg, 719 µmol, 1.00 eq.) in CH_2Cl_2 (5 mL) was treated with phenylacetylene (395 µL, 367 mg, 3.59 mmol, 5.00 eq.). The solution was stirred at rt for 1 d, after which time the stir bar was removed. The solution was allowed to evaporate slowly at rt, furnishing colorless crystals of **8**, which were rinsed with CH_2Cl_2 (3x0.5 mL) and dried in vacuo (100 mg, 263 µmol, 37%).

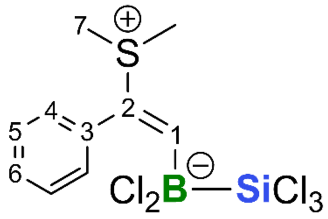

8

^{1}H NMR (300.2 MHz, CD_2Cl_2): δ = 7.57–7.53 (m, 1H, H-6), 7.53–7.48 (m, 2H, H-5), 7.35 (s, 1H, H-1), 7.33–7.29 (m, 2H, H-4), 2.61 (s, 6H, H-7).

^{11}B NMR (96.3 MHz, CD_2Cl_2): δ = –2.6 (s, $h_{½}$ ≈ 110 Hz).

^{13}C{^{1}H} NMR (125.8 MHz, CD_2Cl_2): δ = 163.1 (very br, C-1; confirmed by ^{1}H-^{13}C-HMBC NMR), 132.2 (C-4), 130.8 (C-6), 128.9 (C-5), 128.4 (C-3), 127.3 (C-2), 27.4 (C-7).

^{29}Si NMR (99.4 MHz, CD_2Cl_2): δ = n.o.

EI-MS(+): selected peaks: m/z = 317.8757 ($[C_8H_6BCl_5Si]^{\bullet+}$; calcd.: 317.8749), 182.9959 ($[C_8H_6BCl_2]^+$; calcd.: 182.9939), 134.8825 ($[Cl_3Si]^+$; calcd.: 134.8804), 102.0483 ($[C_8H_6]^{\bullet+}$; calcd.: 102.0470).

Note: The NMR spectra reveal the presence of a second compound, exhibiting a set of signals consistent with a species of similar connectivity as **8** (red, Figure S81 and Figure S83). Integration of the NMR signals indicates that this component accounts for approximately 5% of the sample. After 1 d in solution, the intensity of this second set of signals increases, accompanied by the emergence of signals corresponding to **3**·SMe_2 (Figure S82 and Figure S84). Similar solution behavior has been reported by Stephan *et al.* for the zwitterionic species *E*-(Me_2S–)(Ph–)CCH–B(C_6F_5)$_3$.[S23] These observations indicate an equilibrium between the desired product and its transformation products, which shifts over time and likely accounts for the low isolated yield.

2. Plots of NMR spectra

2.1. NMR spectrum of BI_3

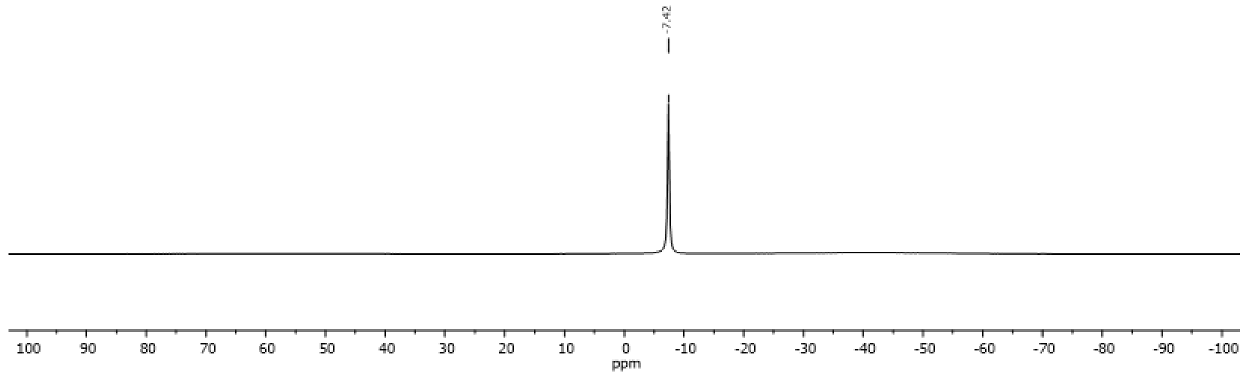

Figure S4: ^{11}B NMR spectrum of BI_3 (96.3 MHz, C_6D_6).

2.2. NMR spectrum of [Et_4N][1]

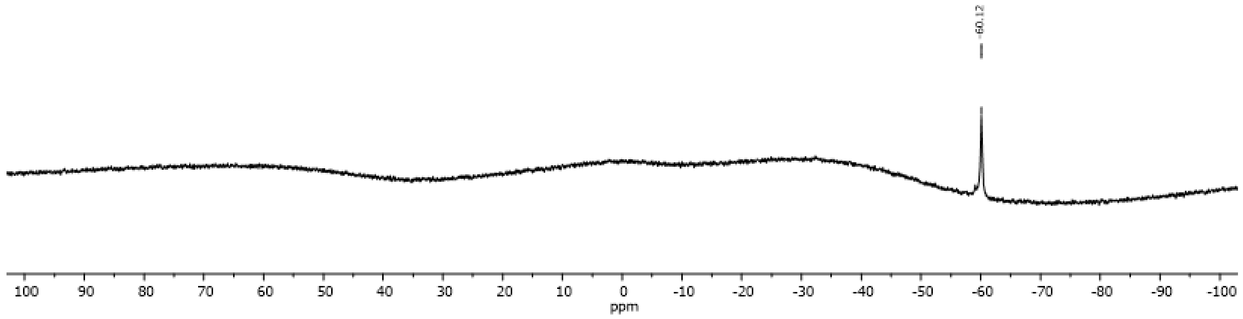

Figure S5: ^{11}B NMR spectrum of [Et_4N][**1**] (96.3 MHz, CD_2Cl_2).

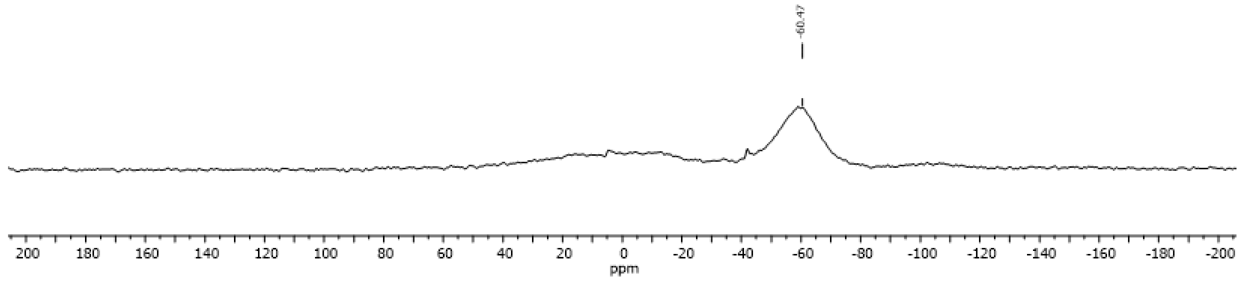

Figure S6: Solid-state ^{11}B NMR spectrum of [Et_4N][**1**] (192.6 MHz, MAS, spinning rate: 9 kHz, recycle delay: 4 s).

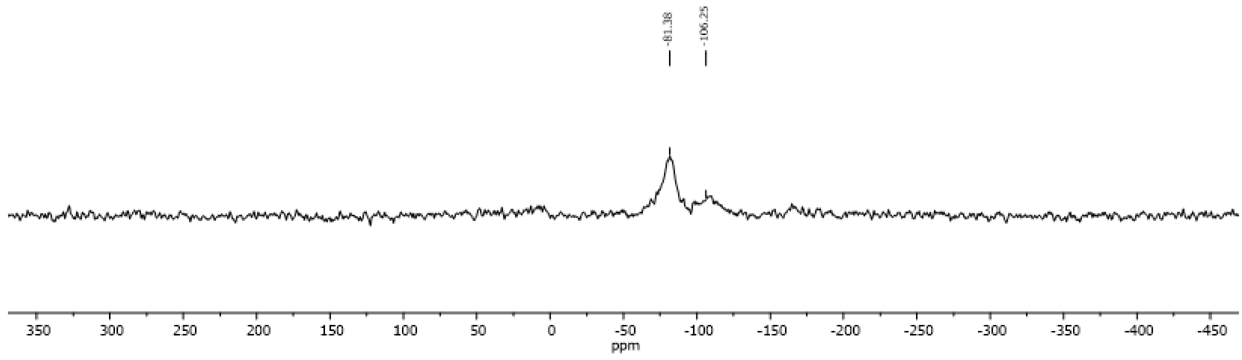

Figure S7: Solid-state ^{29}Si NMR spectrum of [Et_4N][**1**] (119.2 MHz, MAS, spinning rate: 10 kHz, recycle delay: 40 s).

2.3. NMR spectrum of $[Et_4N][(I_{2.03}/Cl_{0.97})B–SiI_3]$

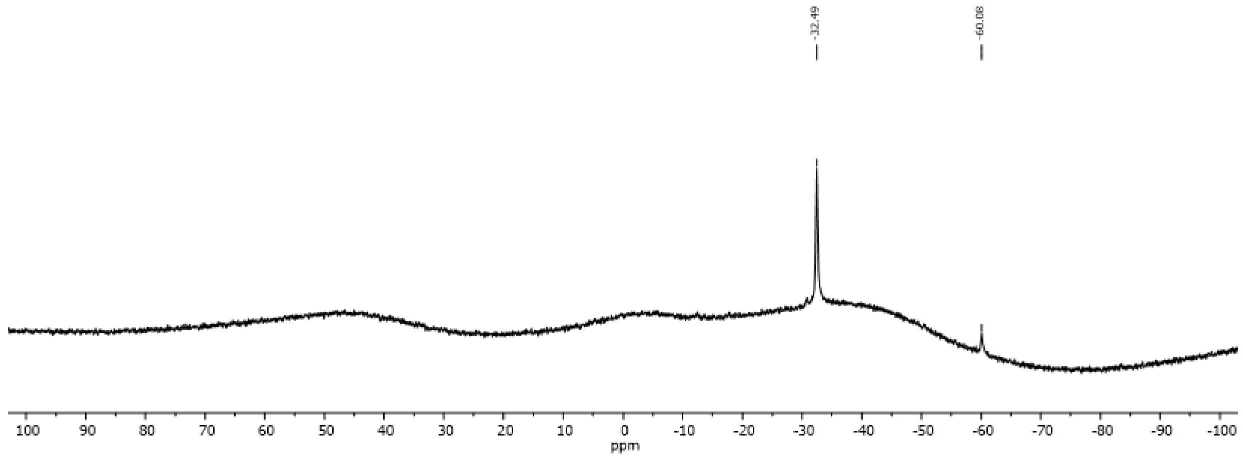

Figure S8: ^{11}B NMR spectrum of $[Et_4N][XI_2B–SiI_3]$ (X = 0.97 Cl, 0.03 I) (96.3 MHz, CD_2Cl_2).

2.4. NMR spectra of Li[1], 2/LiI

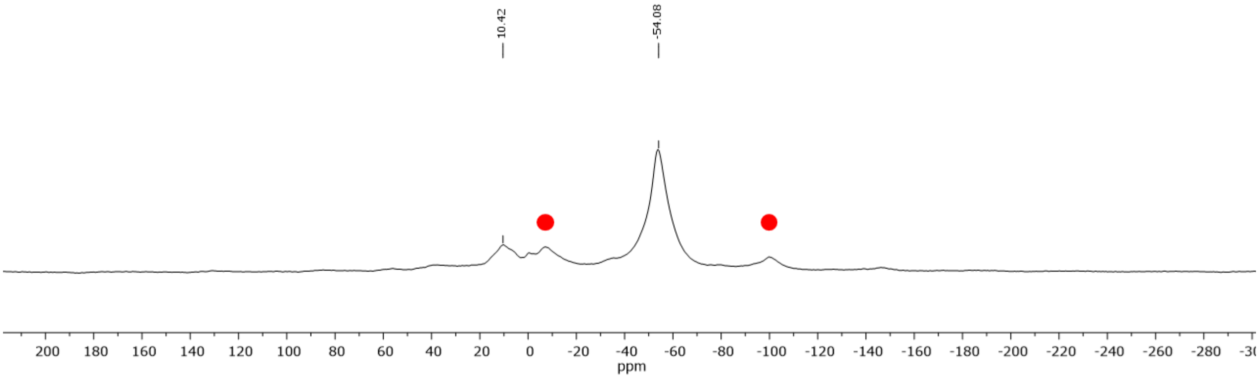

Figure S9: Solid-state ^{11}B NMR spectrum of Li[**1**], **2**/LiI (192.6 MHz, MAS, spinning rate: 9 kHz, recycle delay: 4 s). *Note:* Spinning side bands are indicated in red.

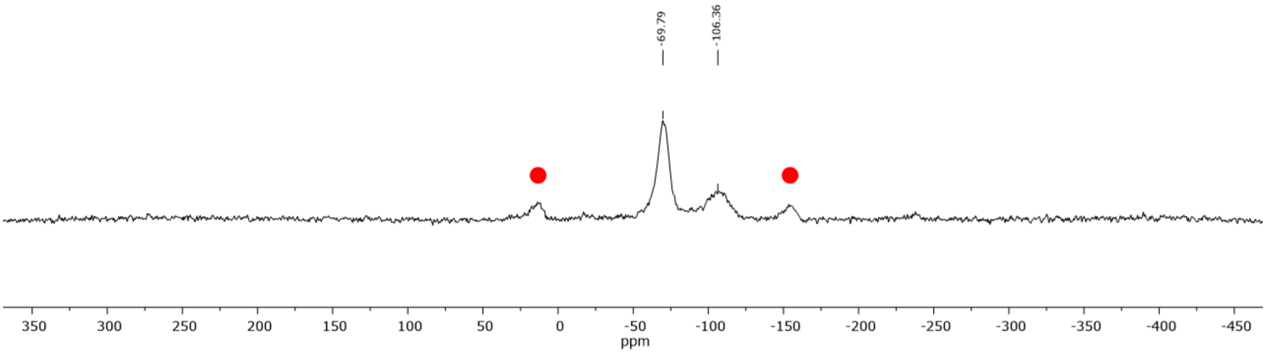

Figure S10: Solid-state ^{29}Si NMR spectrum of Li[**1**], **2**/LiI (119.2 MHz, MAS, spinning rate: 10 kHz, recycle delay: 40 s). *Note:* Spinning side bands are indicated in red.

2.5. NMR spectra of $Me_2S{\cdot}I_2B–SiI_3$ (2·SMe_2)

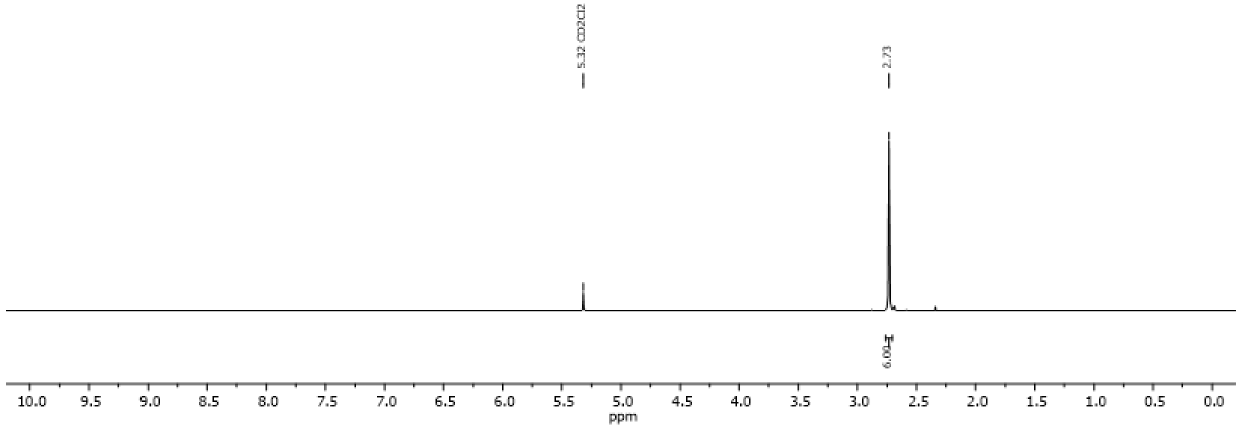

Figure S11: ^{1}H NMR spectrum of **2**·SMe_2 (500.2 MHz, CD_2Cl_2).

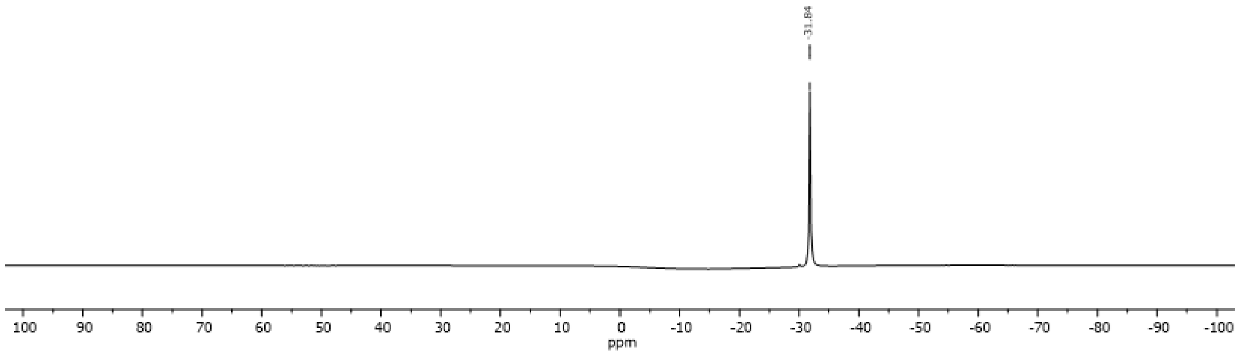

Figure S12: ^{11}B NMR spectrum of **2**·SMe_2 (160.5 MHz, CD_2Cl_2).

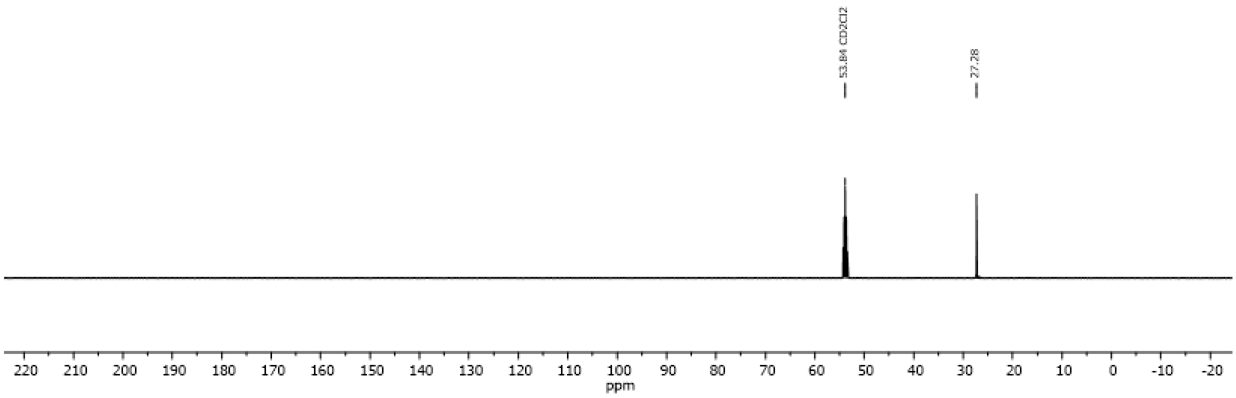

Figure S13: $^{13}C\{^{1}H\}$ NMR spectrum of **2**·SMe_2 (125.8 MHz, CD_2Cl_2).

2.6. NMR spectra of Py·I_2B–SiI_3 (2·Py)

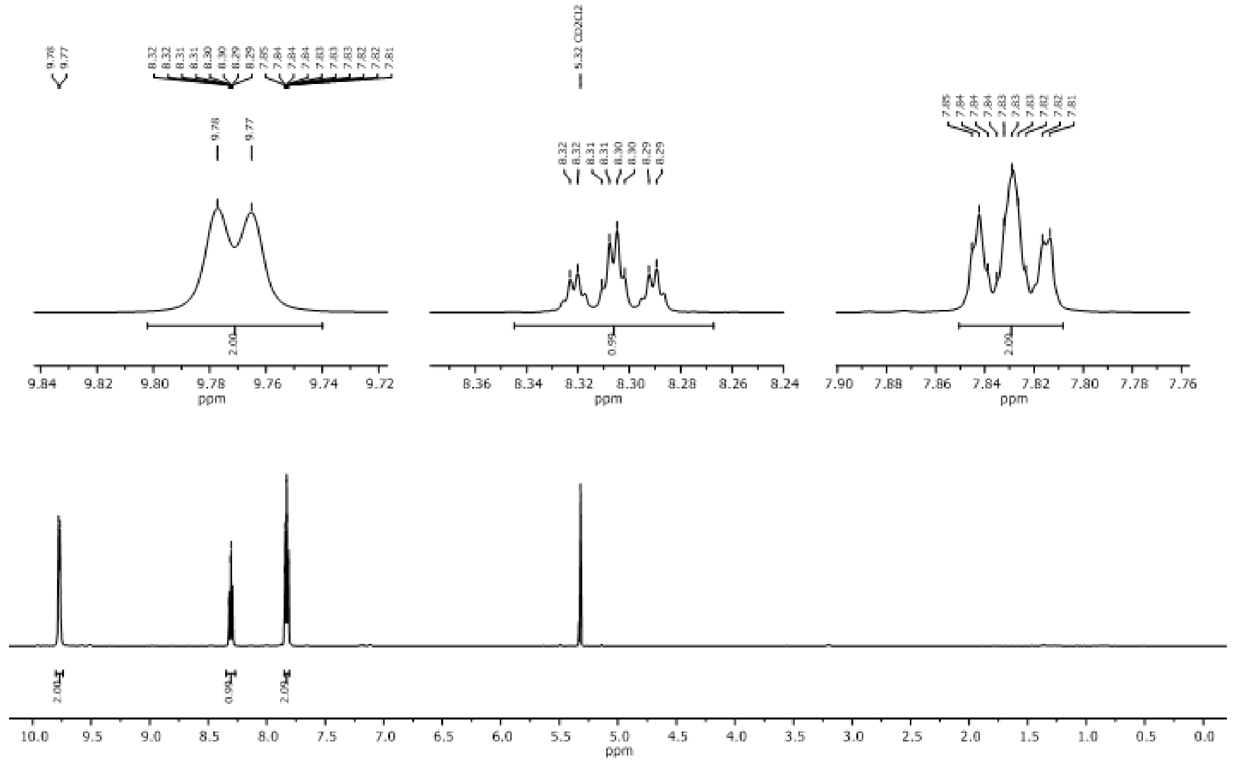

Figure S14: ^{1}H NMR spectrum of **2**·Py (500.2 MHz, CD_2Cl_2).

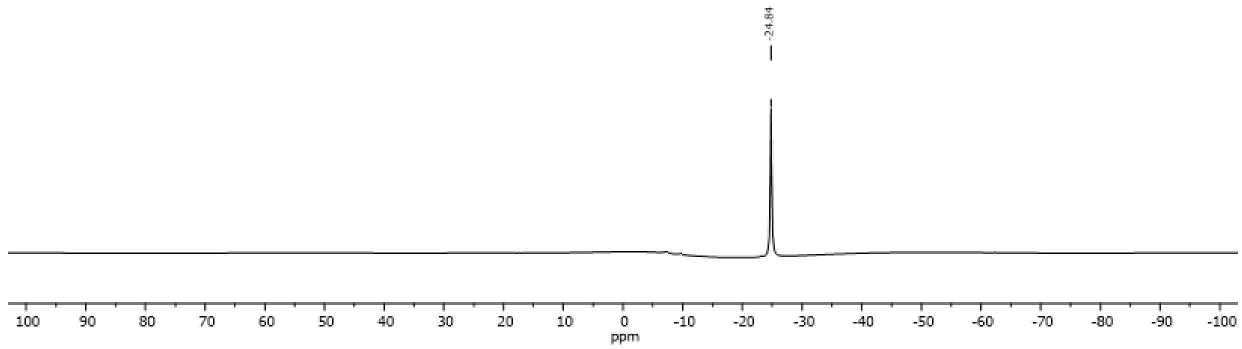

Figure S15: ^{11}B NMR spectrum of **2**·Py (160.5 MHz, CD_2Cl_2).

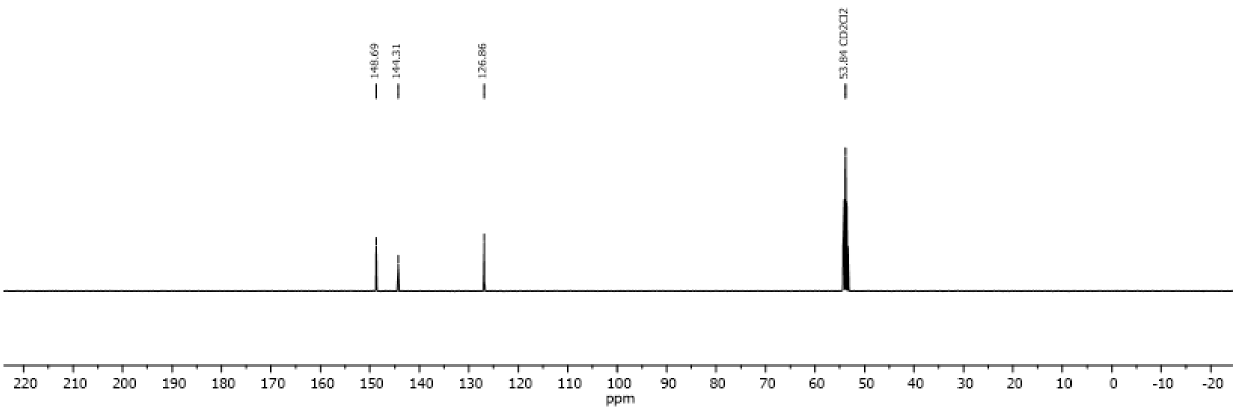

Figure S16: $^{13}C\{^{1}H\}$ NMR spectrum of **2**·Py (125.8 MHz, CD_2Cl_2).

2.7. NMR spectra of $Ph_3P{\cdot}I_2B–SiI_3$ ($2{\cdot}PPh_3$)

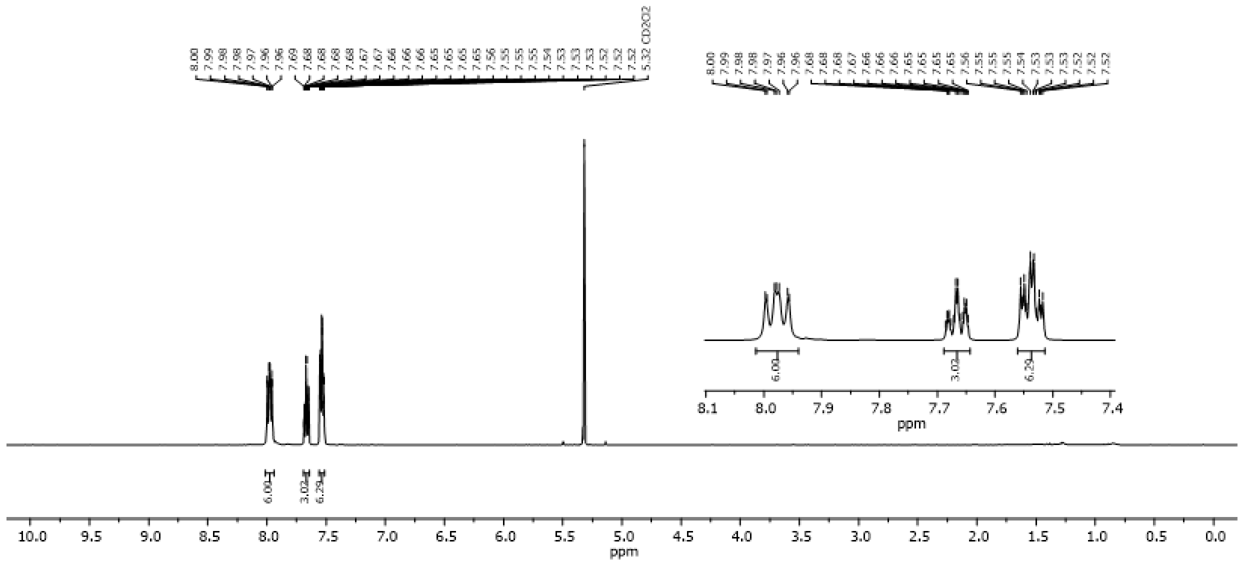

Figure S17: ^1H NMR spectrum of **2**·PPh_3 (500.2 MHz, CD_2Cl_2).

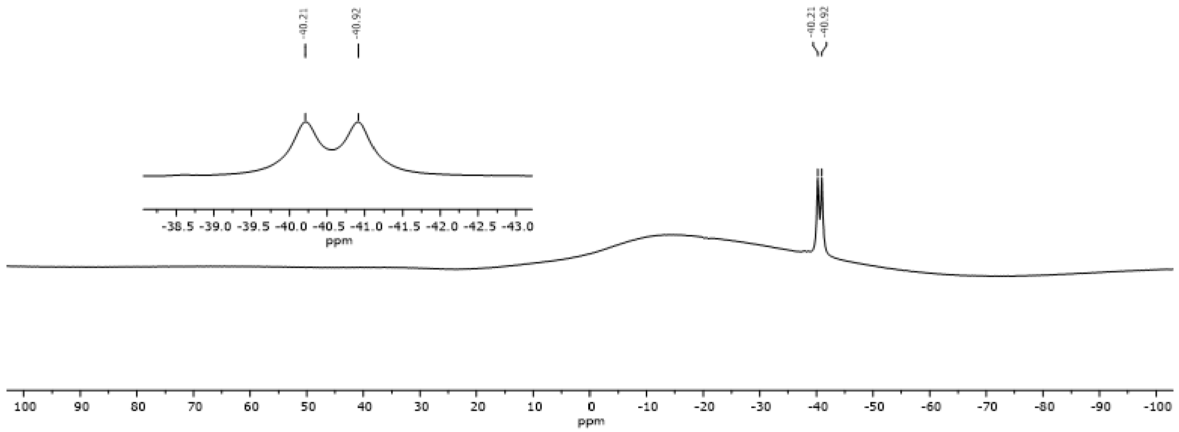

Figure S18: ^{11}B NMR spectrum of **2**·PPh_3 (160.5 MHz, CD_2Cl_2).

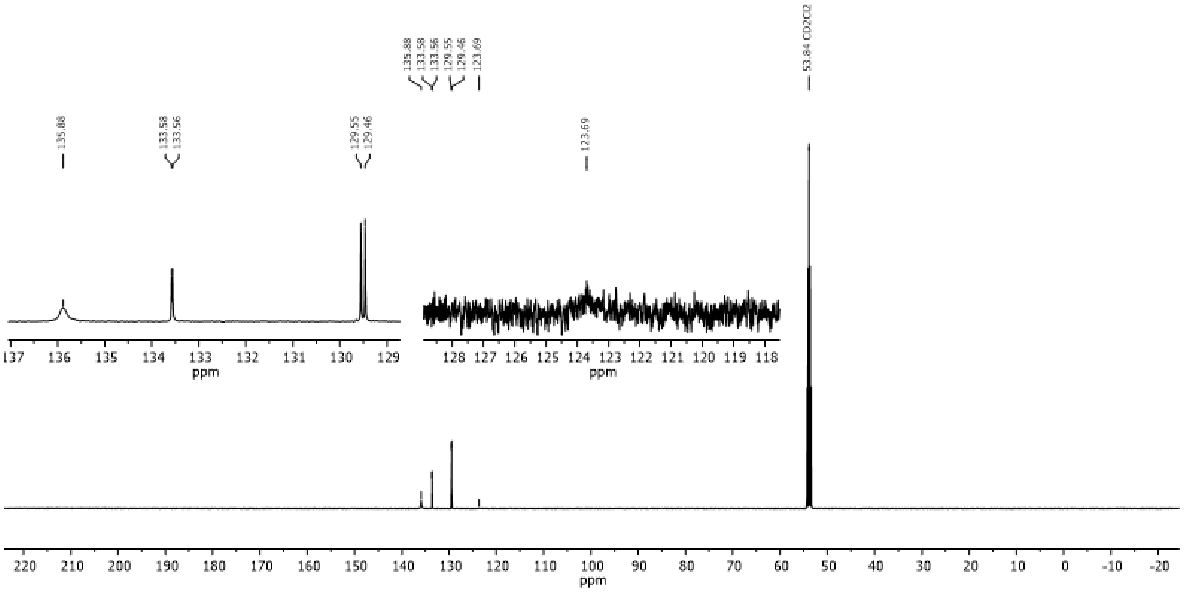

Figure S19: $^{13}C\{^1H\}$ NMR spectrum of **2**·PPh_3 (125.8 MHz, CD_2Cl_2).

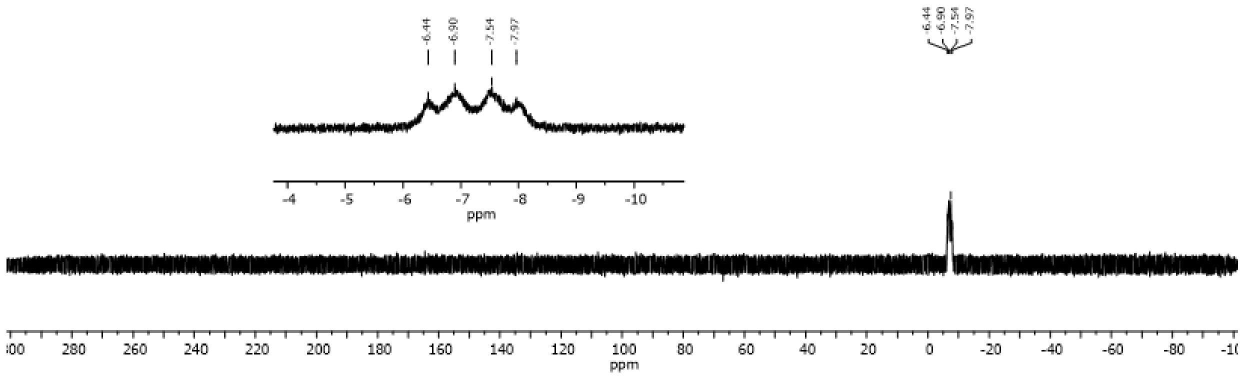

Figure S20: $^{31}P\{^{1}H\}$ NMR spectrum of **2**·PPh_3 (202.5 MHz, CD_2Cl_2).

2.8. NMR spectra of IDipp·I_2B–SiI_3 (2·IDipp)

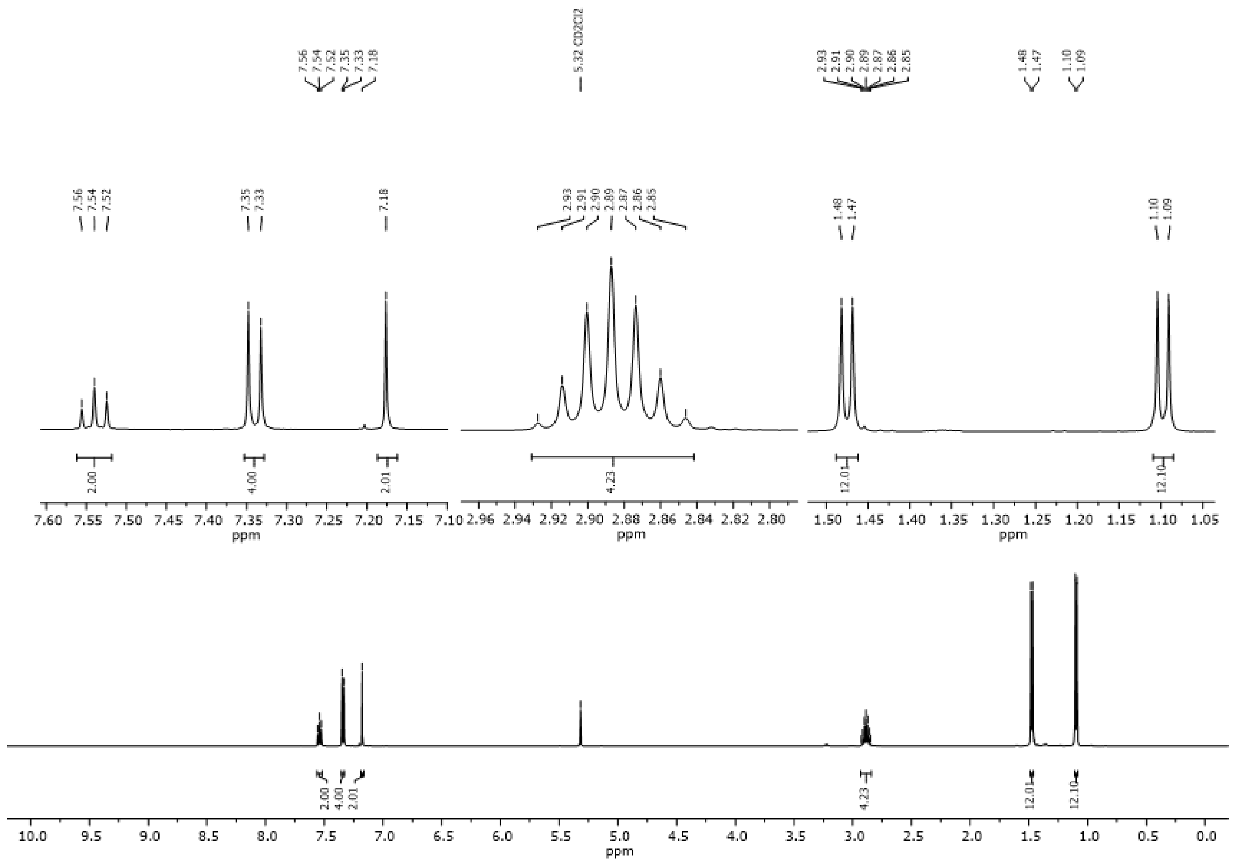

Figure S21: ^{1}H NMR spectrum of **2**·IDipp (500.2 MHz, CD_2Cl_2).

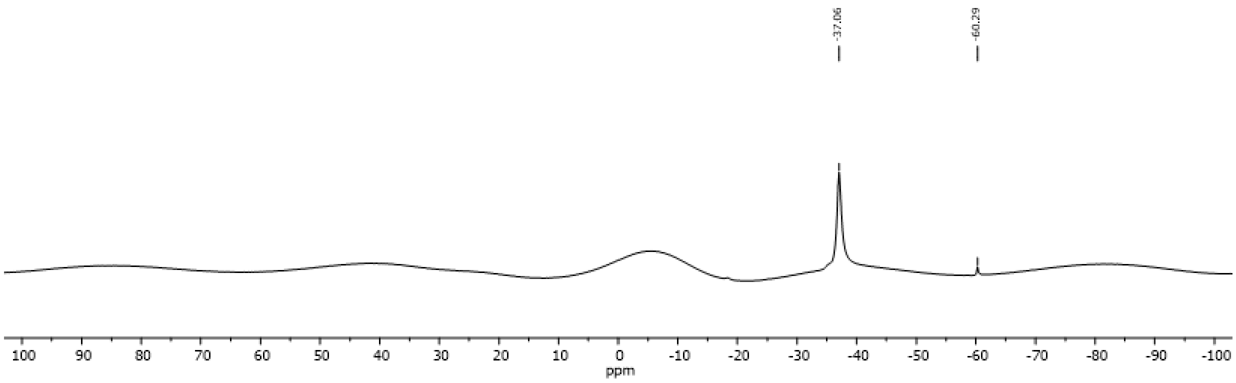

Figure S22: ^{11}B NMR spectrum of **2**·IDipp (160.5 MHz, CD_2Cl_2). *Note:* Due to the similarly poor solubility of [Et_4N][**1**] and **2**·IDipp a small amount of the unconsumed starting material could not be separated from the product and is therefore still visible in the ^{11}B NMR spectrum at –60.3 ppm.

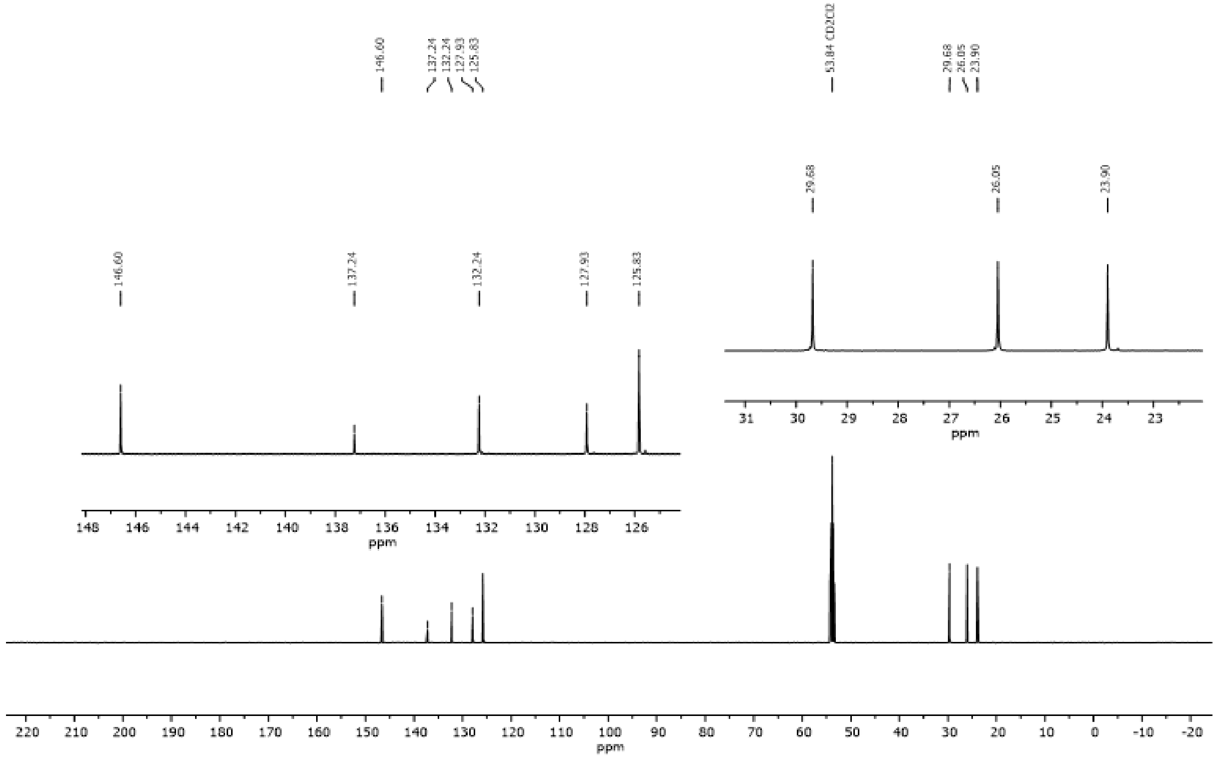

Figure S23: $^{13}C\{^1H\}$ NMR spectrum of **2**·IDipp (125.8 MHz, CD_2Cl_2).

2.9. NMR spectra of $Cl_2B–SiCl_3$ (3)

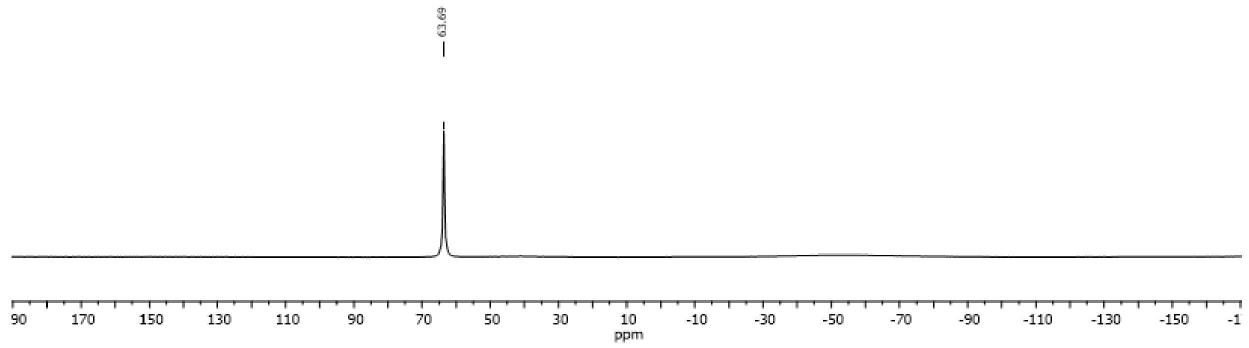

Figure S24: ^{11}B NMR spectrum of **3** (96.3 MHz, no solvent).

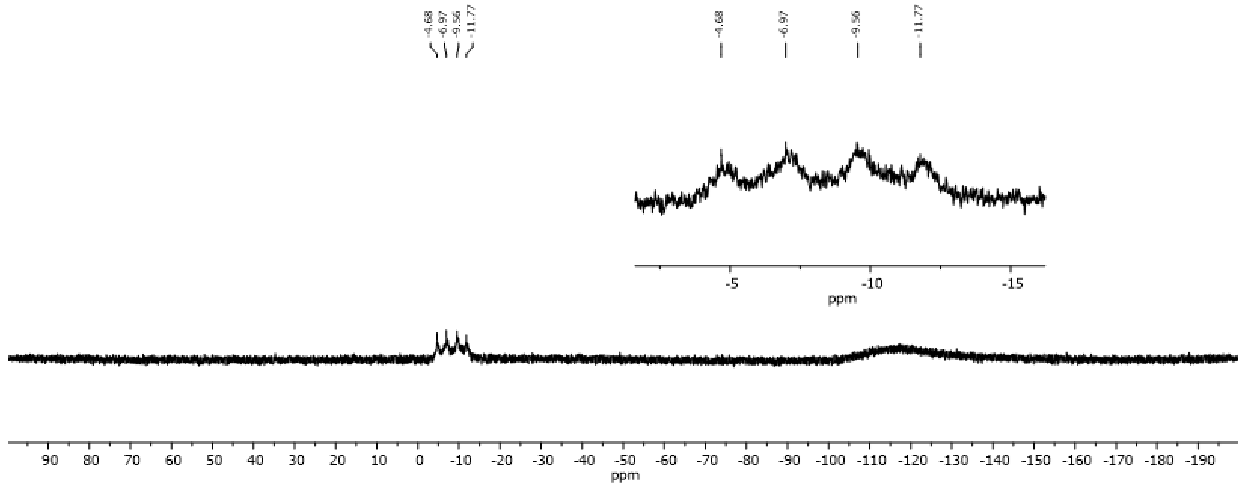

Figure S25: ^{29}Si NMR spectrum of **3** (79.5 MHz, no solvent).

2.10. NMR spectra of $(I_2B–SiI_3)_2·SiI_2$ (4)

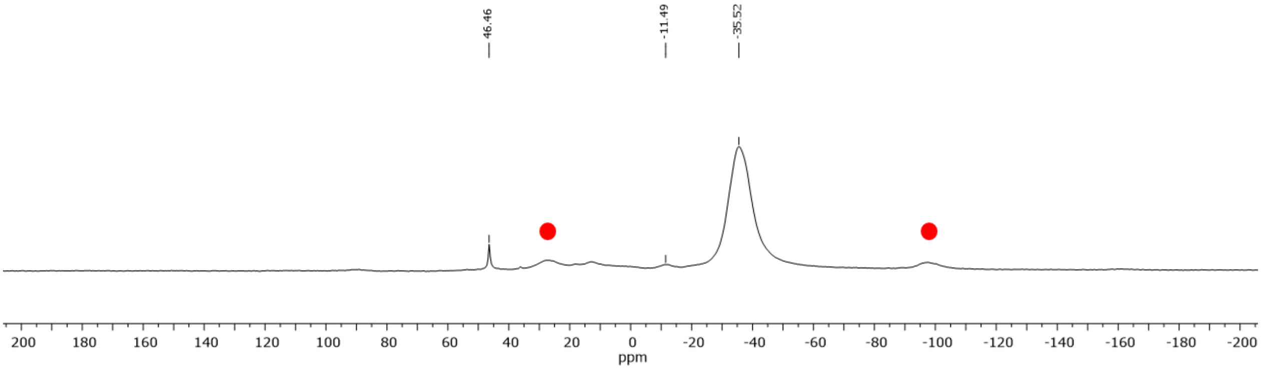

Figure S26: Solid-state ^{11}B NMR spectrum of **4** (192.6 MHz, MAS, spinning rate: 12 kHz, recycle delay: 10 s). *Note:* Spinning side bands are indicated in red.

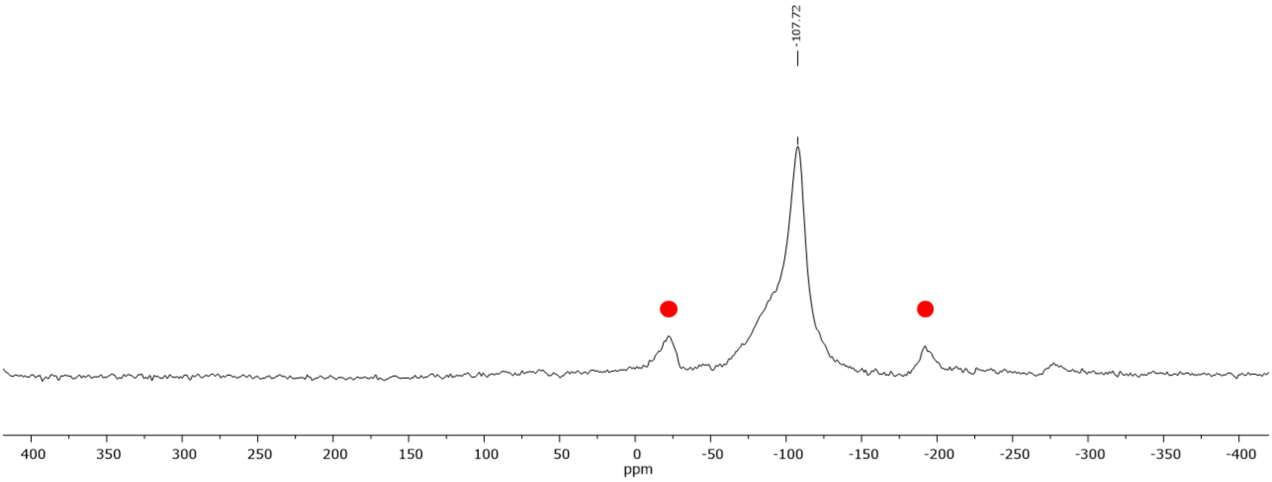

Figure S27: Solid-state ^{29}Si NMR spectrum of **4** (119.2 MHz, MAS, spinning rate: 10 kHz, recycle delay: 20 s). *Note:* Spinning side bands are indicated in red.

2.11. NMR spectra of $[Et_4N][Cl_3B–SiCl_3]$

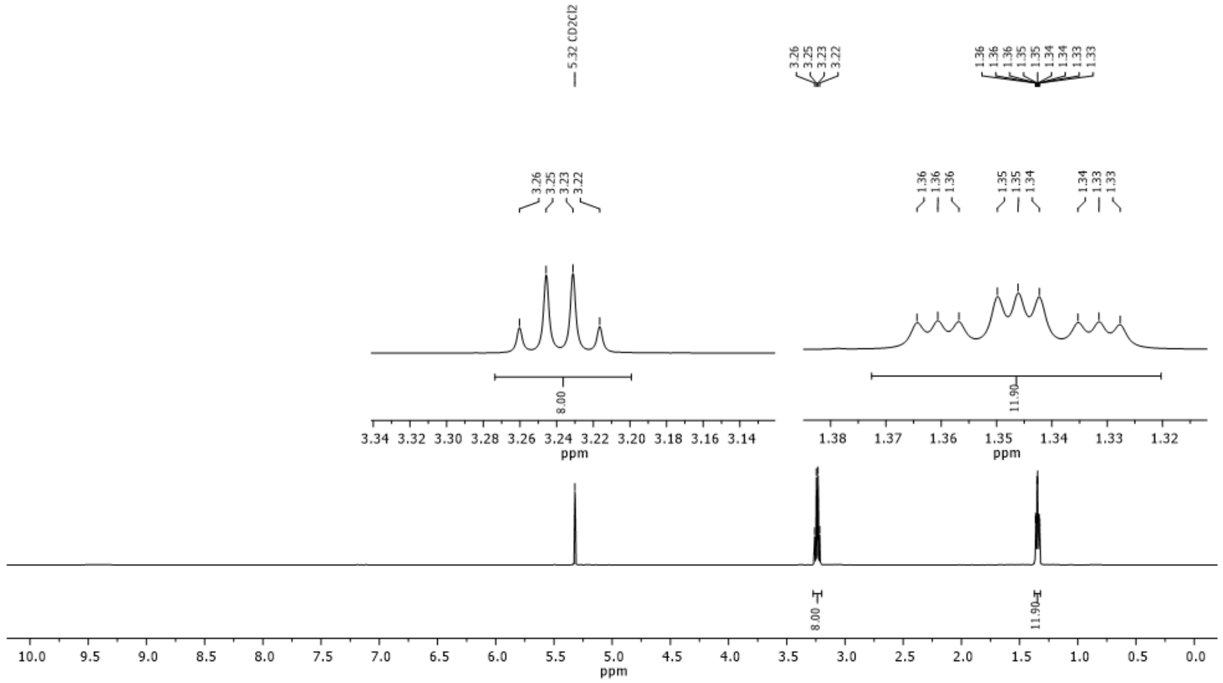

Figure S28: ^{1}H NMR spectrum of $[Et_4N][Cl_3B–SiCl_3]$ (500.2 MHz, CD_2Cl_2).

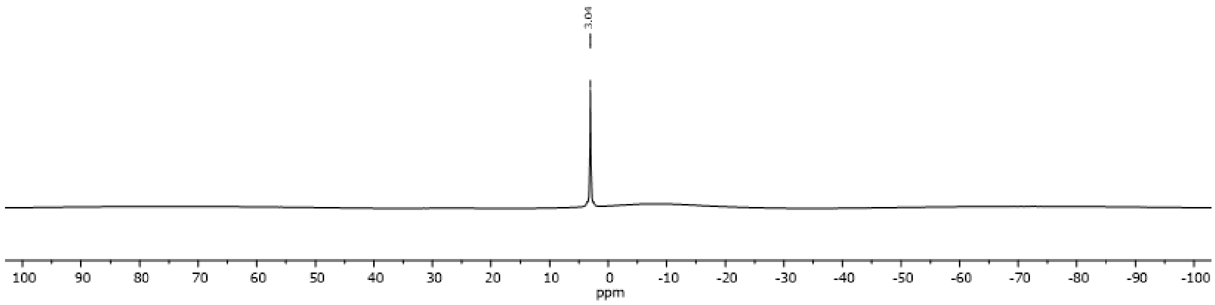

Figure S29: ^{11}B NMR spectrum of $[Et_4N][Cl_3B–SiCl_3]$ (160.5 MHz, CD_2Cl_2).

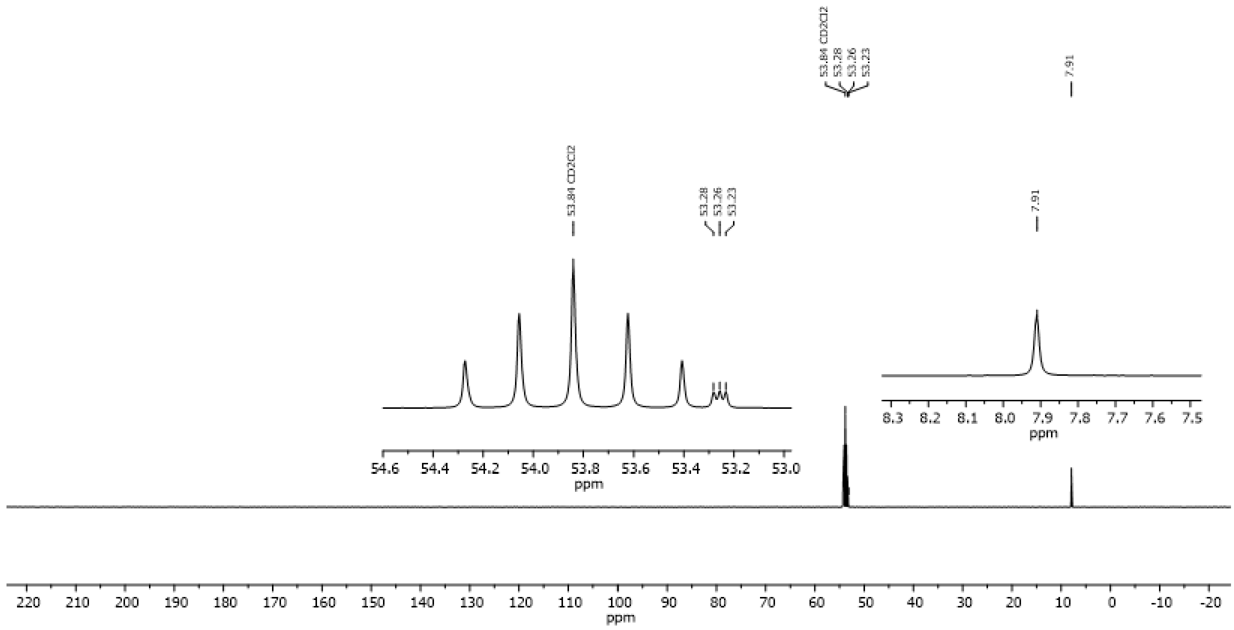

Figure S30: $^{13}C\{^{1}H\}$ NMR spectrum of $[Et_4N][Cl_3B–SiCl_3]$ (125.8 MHz, CD_2Cl_2).

2.12. NMR spectra of $Me_2S{\cdot}Cl_2B–SiCl_3$ (3·SMe_2)

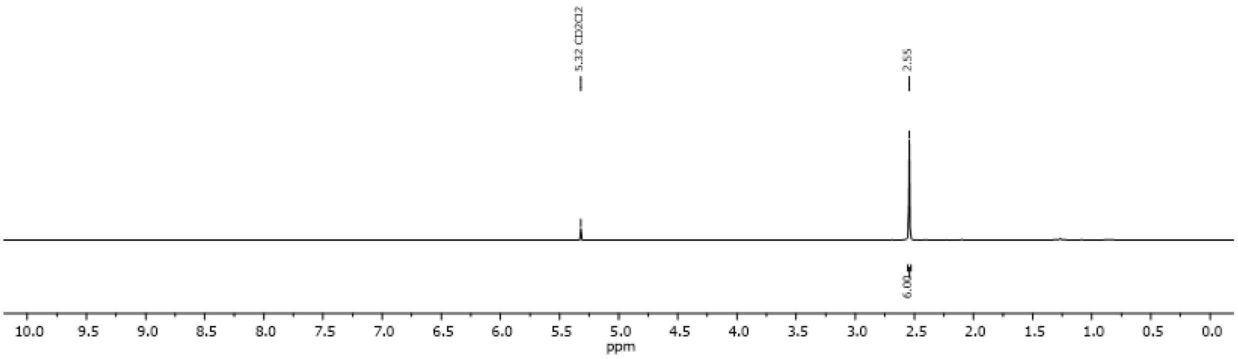

Figure S31: ^{1}H NMR spectrum of **3**·SMe_2 (500.2 MHz, CD_2Cl_2).

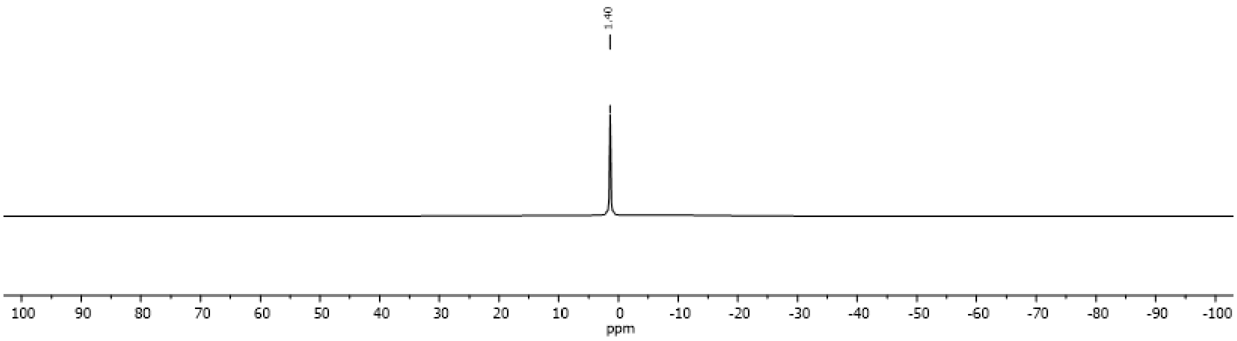

Figure S32: ^{11}B NMR spectrum of **3**·SMe_2 (160.5 MHz, CD_2Cl_2).

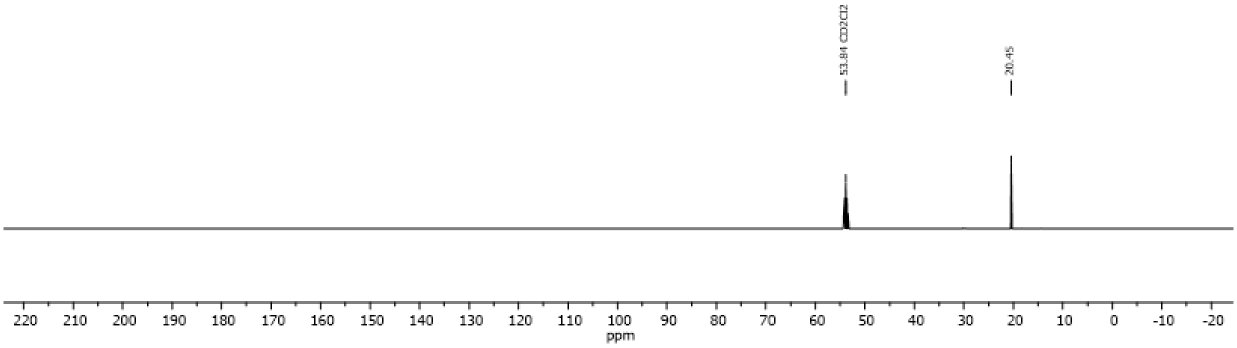

Figure S33: $^{13}C\{^{1}H\}$ NMR spectrum of **3**·SMe_2 (125.8 MHz, CD_2Cl_2).

2.13. NMR spectra of Py·Cl_2B–$SiCl_3$ (3·Py)

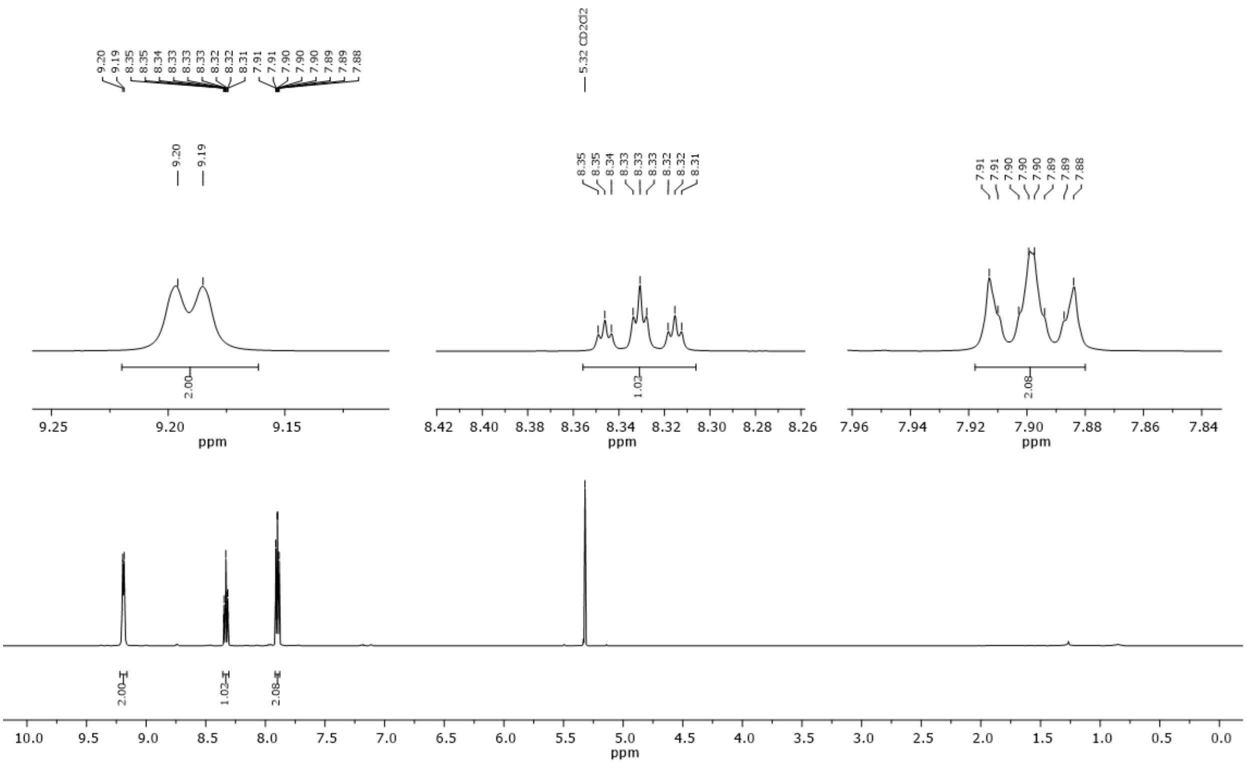

Figure S34: ^{1}H NMR spectrum of **3**·Py (500.2 MHz, CD_2Cl_2).

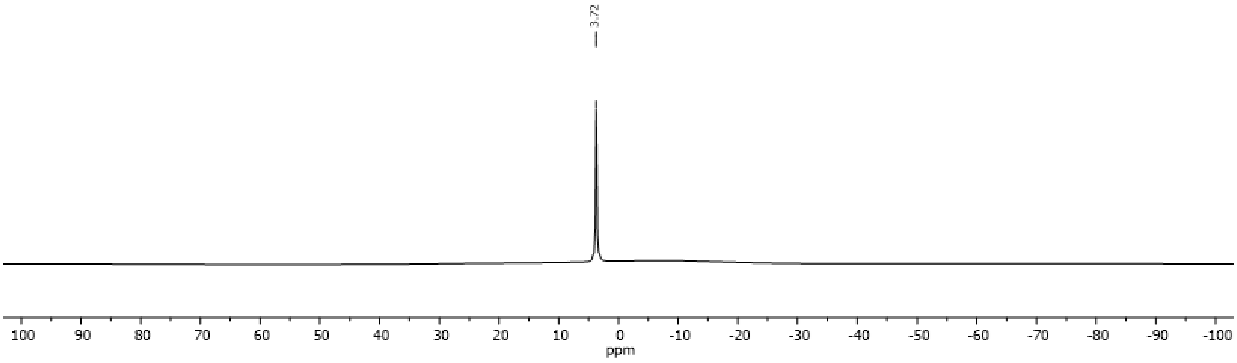

Figure S35: ^{11}B NMR spectrum of **3**·Py (160.5 MHz, CD_2Cl_2).

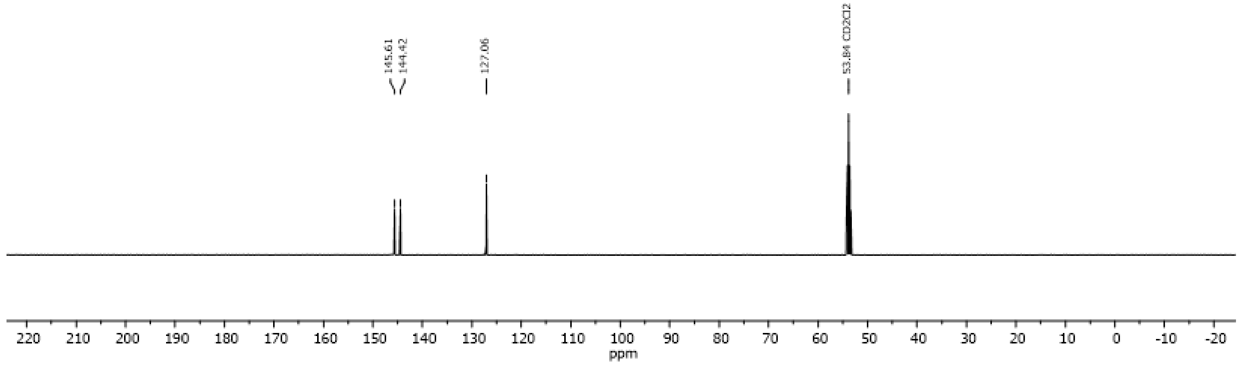

Figure S36: $^{13}C\{^{1}H\}$ NMR spectrum of **3**·Py (125.8 MHz, CD_2Cl_2).

2.14. NMR spectra of $Ph_3P{\cdot}Cl_2B–SiCl_3$ (3·PPh_3)

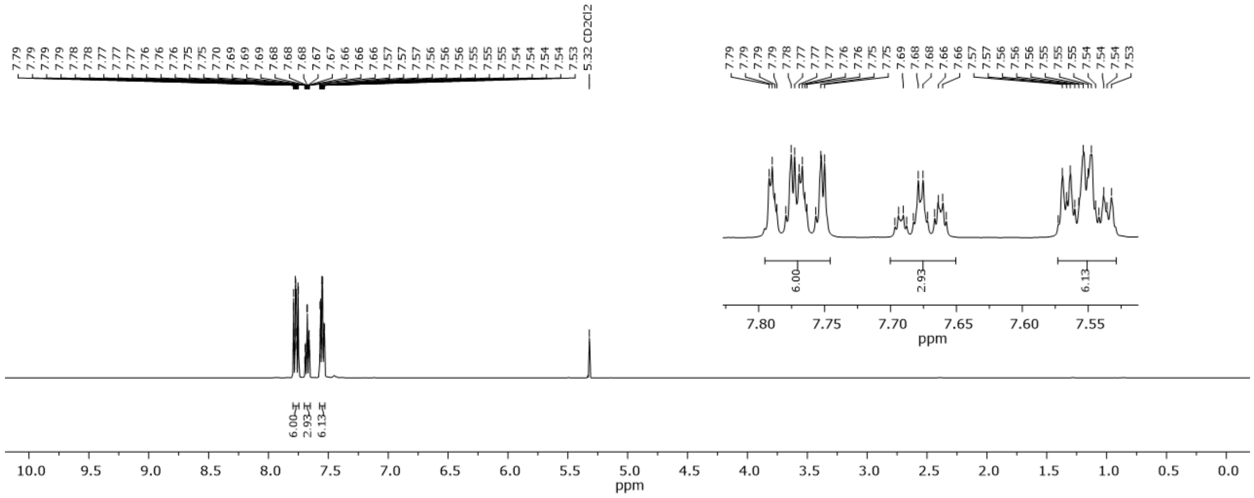

Figure S37: ^{1}H NMR spectrum of **3**·PPh_3 (500.2 MHz, CD_2Cl_2).

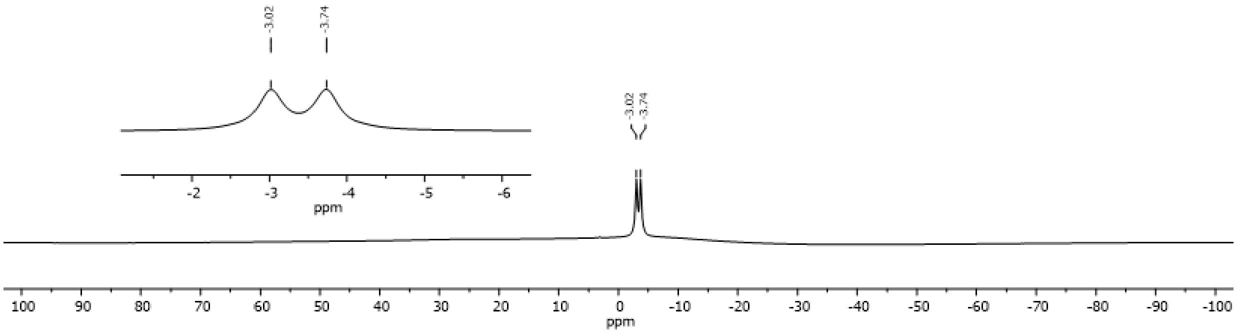

Figure S38: ^{11}B NMR spectrum of **3**·PPh_3 (160.5 MHz, CD_2Cl_2).

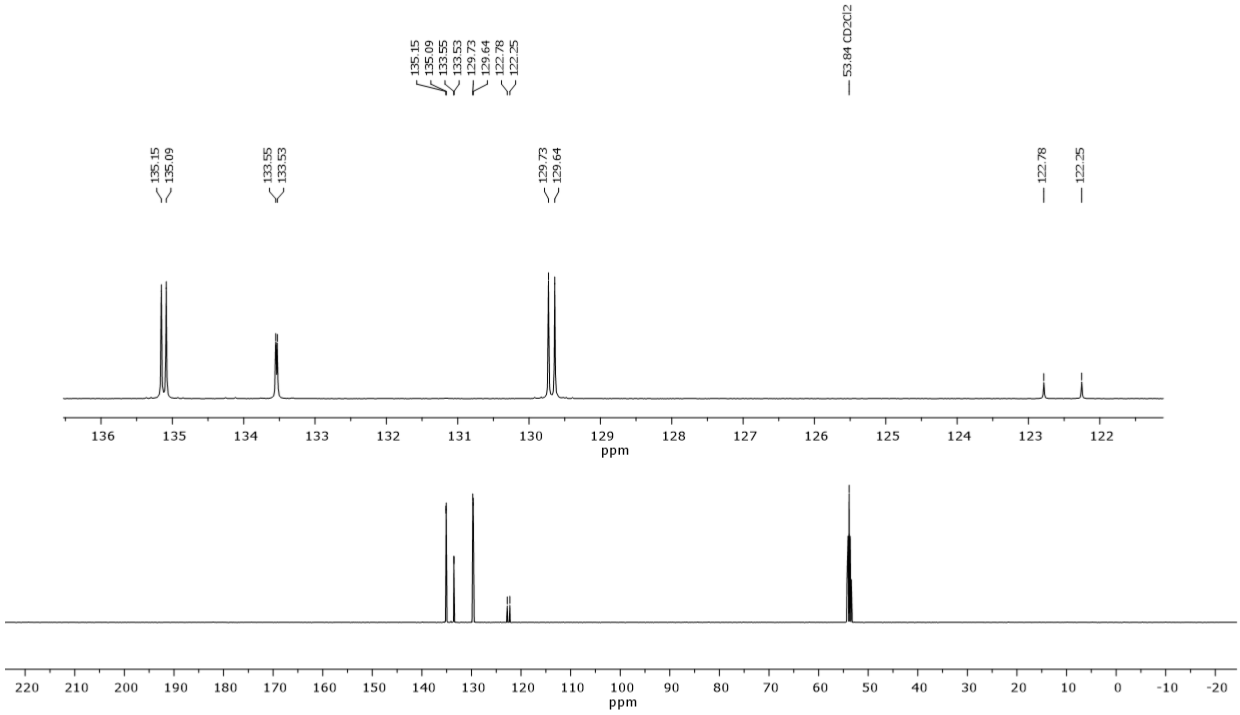

Figure S39: $^{13}C\{^{1}H\}$ NMR spectrum of **3**·PPh_3 (125.8 MHz, CD_2Cl_2).

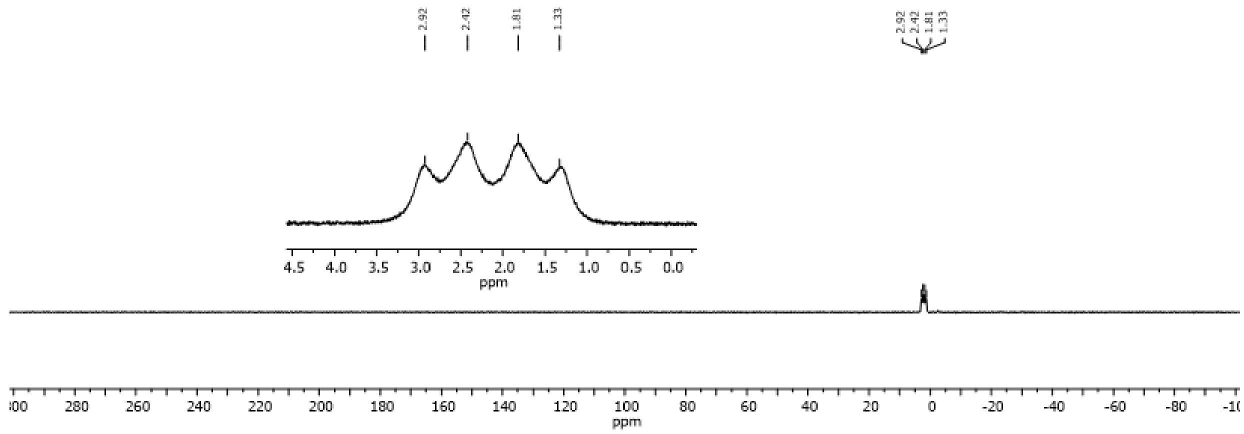

Figure S40: $^{31}P\{^{1}H\}$ NMR spectrum of **3**·PPh_3 (202.5 MHz, CD_2Cl_2).

2.15. NMR spectra of IDipp·Cl_2B–$SiCl_3$ (3·IDipp)

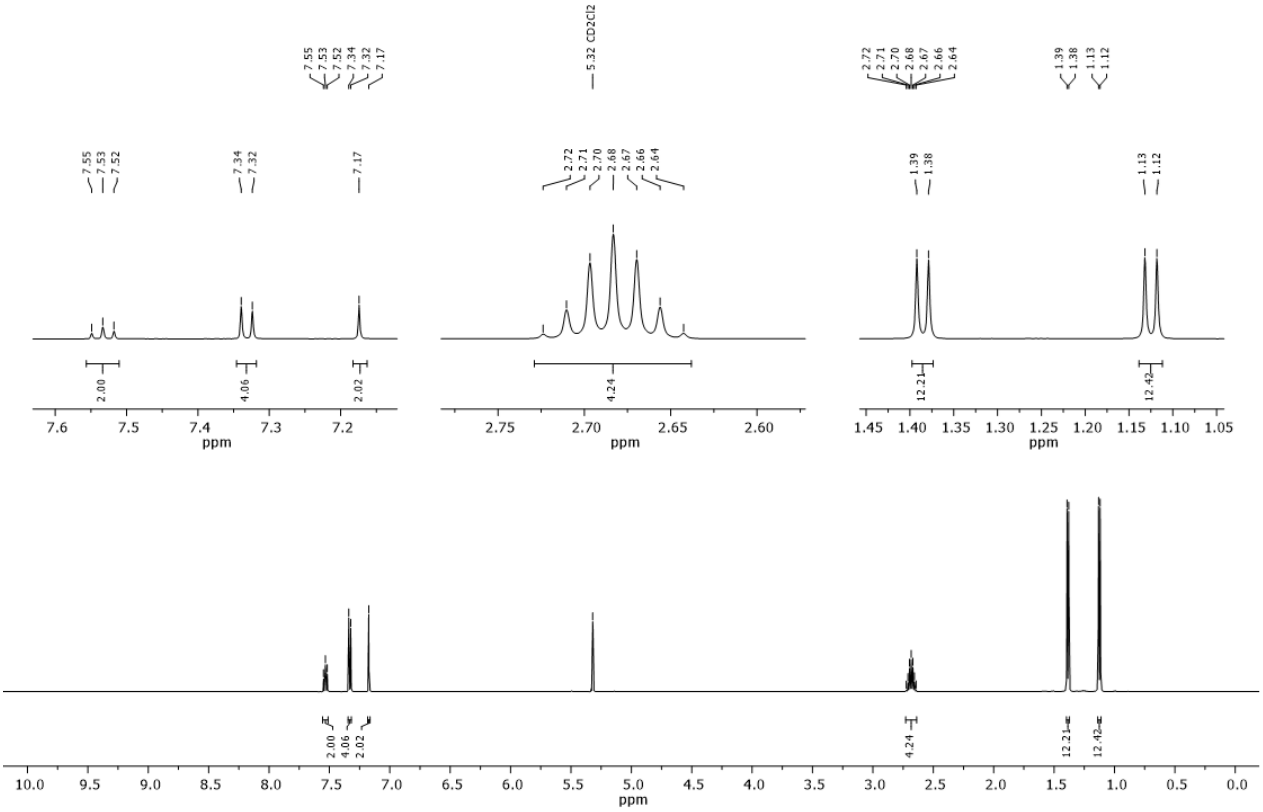

Figure S41: ^{1}H NMR spectrum of **3**·IDipp (500.2 MHz, CD_2Cl_2).

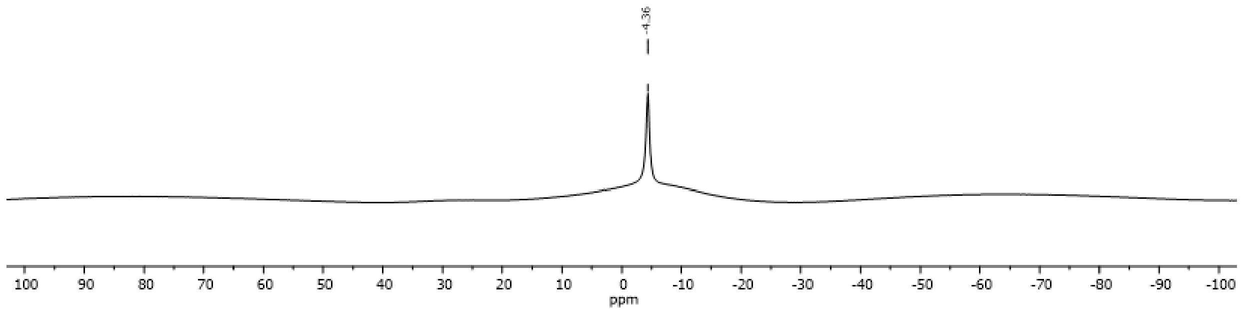

Figure S42: ^{11}B NMR spectrum of **3**·IDipp (160.5 MHz, CD_2Cl_2).

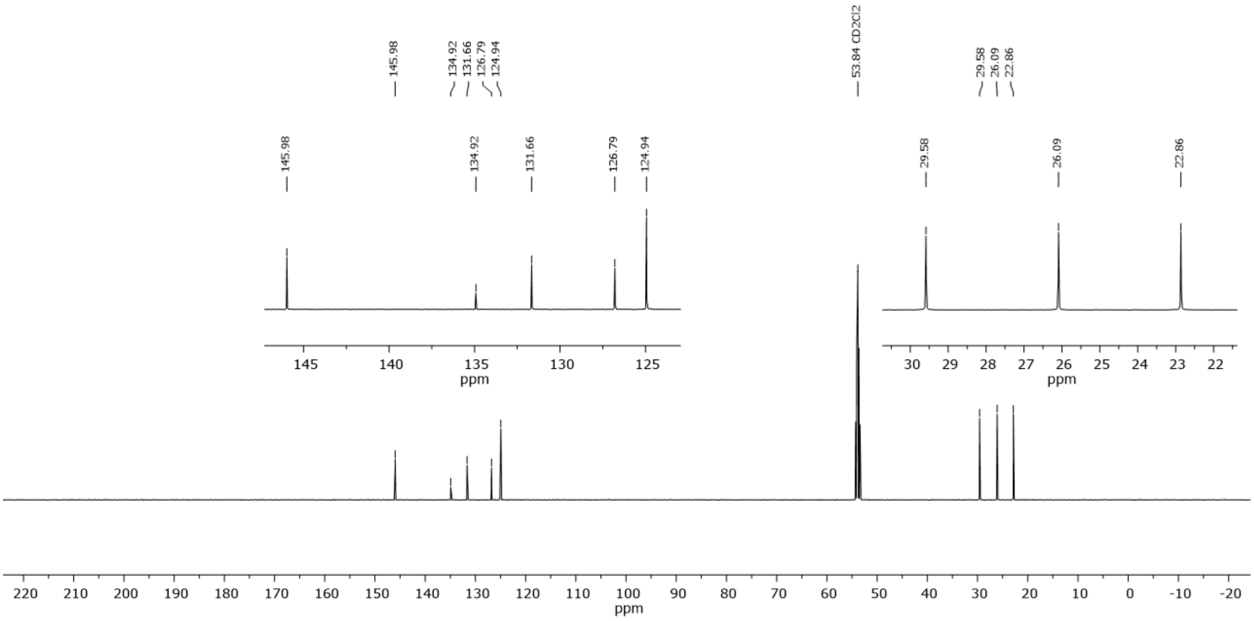

Figure S43: $^{13}C\{^{1}H\}$ NMR spectrum of **3**·IDipp (125.8 MHz, CD_2Cl_2).

2.16. NMR spectra of $BI_3 \cdot PPh_3$

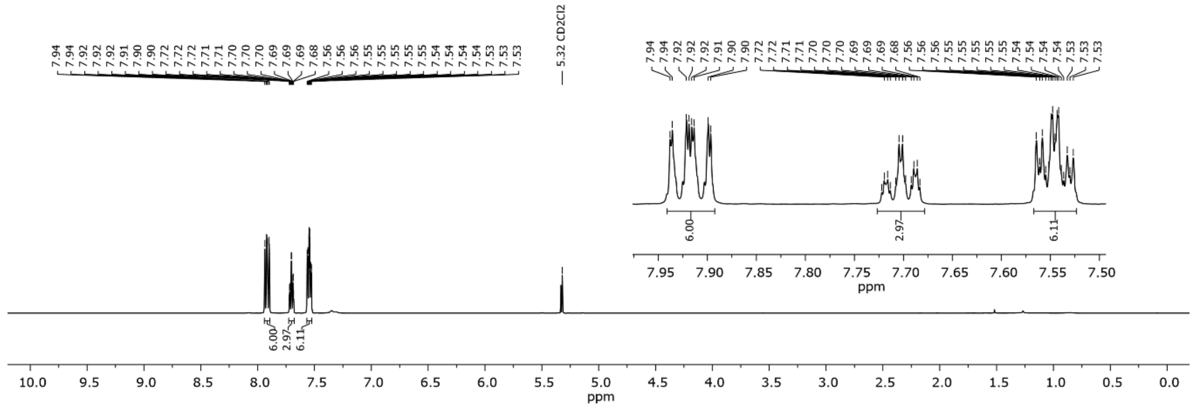

Figure S44: ^{1}H NMR spectrum of $BI_3 \cdot PPh_3$ (500.2 MHz, CD_2Cl_2).

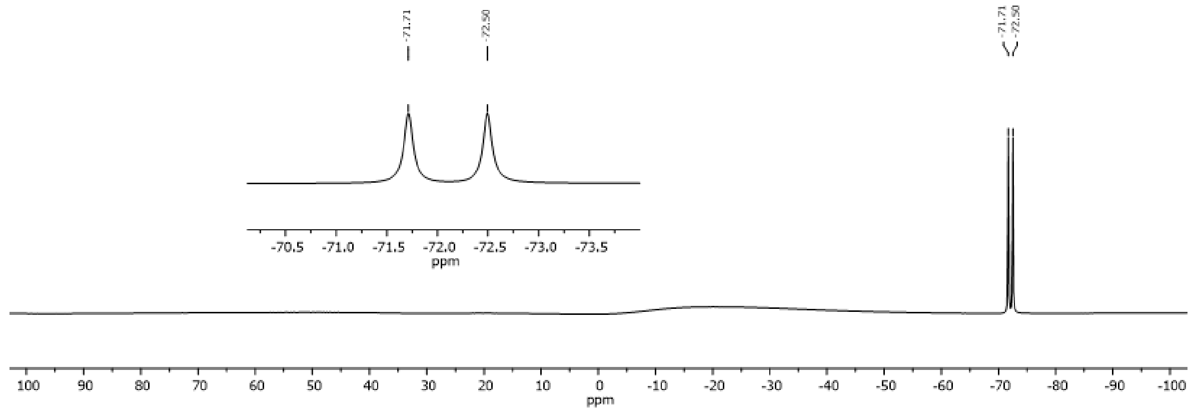

Figure S45: ^{11}B NMR spectrum of $BI_3 \cdot PPh_3$ (160.5 MHz, CD_2Cl_2).

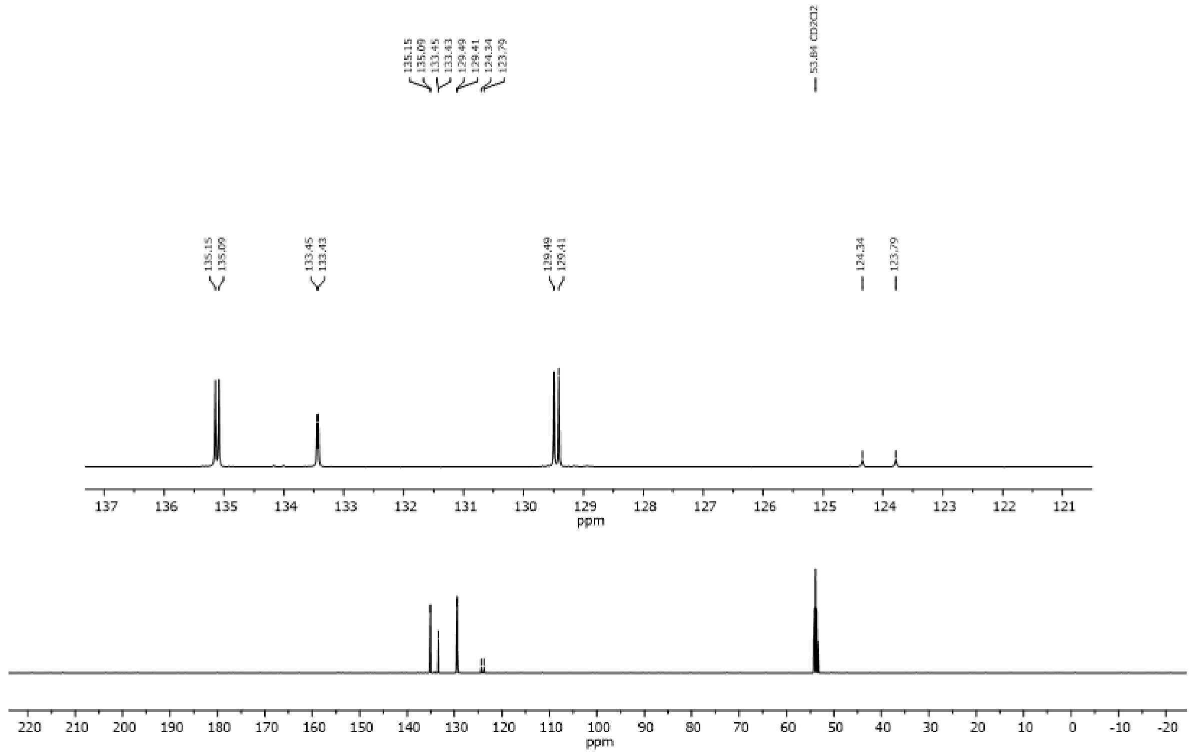

Figure S46: $^{13}C\{^{1}H\}$ NMR spectrum of $BI_3 \cdot PPh_3$ (125.8 MHz, CD_2Cl_2).

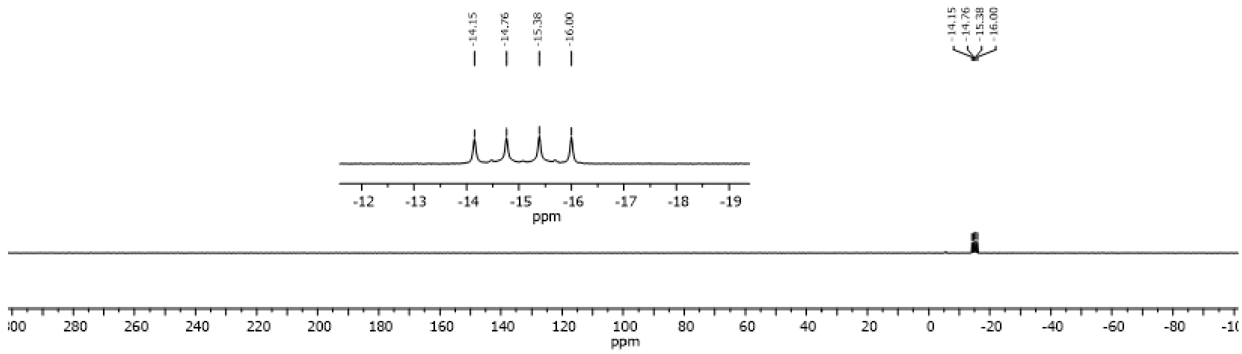

Figure S47: $^{31}P\{^{1}H\}$ NMR spectrum of $BI_3{\cdot}PPh_3$ (202.5 MHz, CD_2Cl_2).

2.17. NMR spectra of $BI_3{\cdot}$IDipp

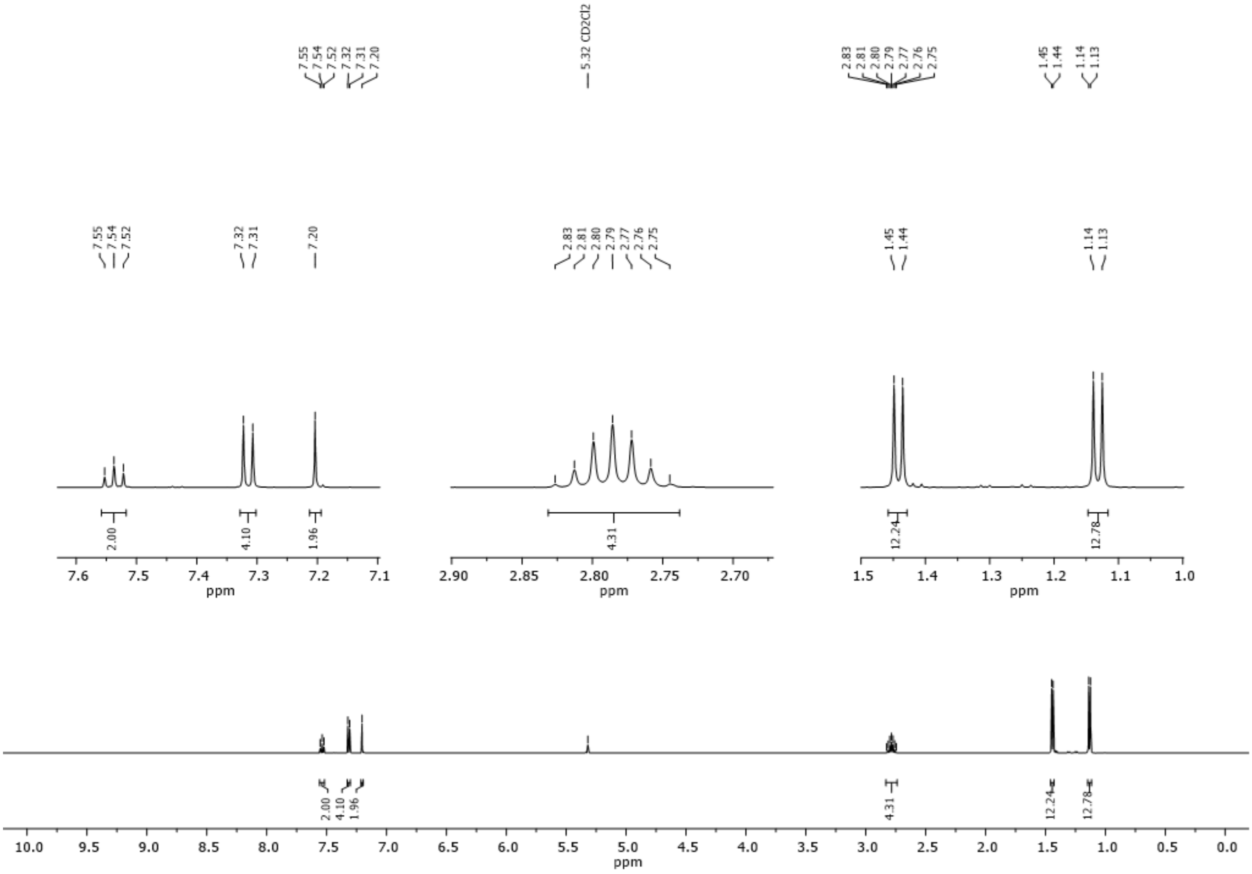

Figure S48: ^{1}H NMR spectrum of $BI_3{\cdot}$IDipp (500.2 MHz, CD_2Cl_2).

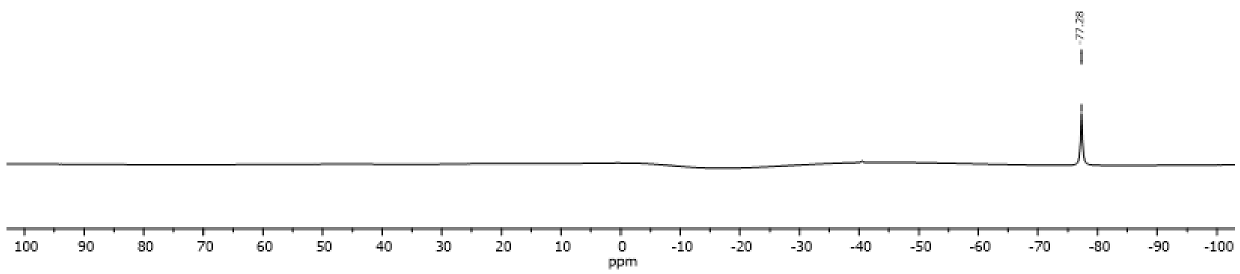

Figure S49: ^{11}B NMR spectrum of BI_3·IDipp (160.5 MHz, CD_2Cl_2).

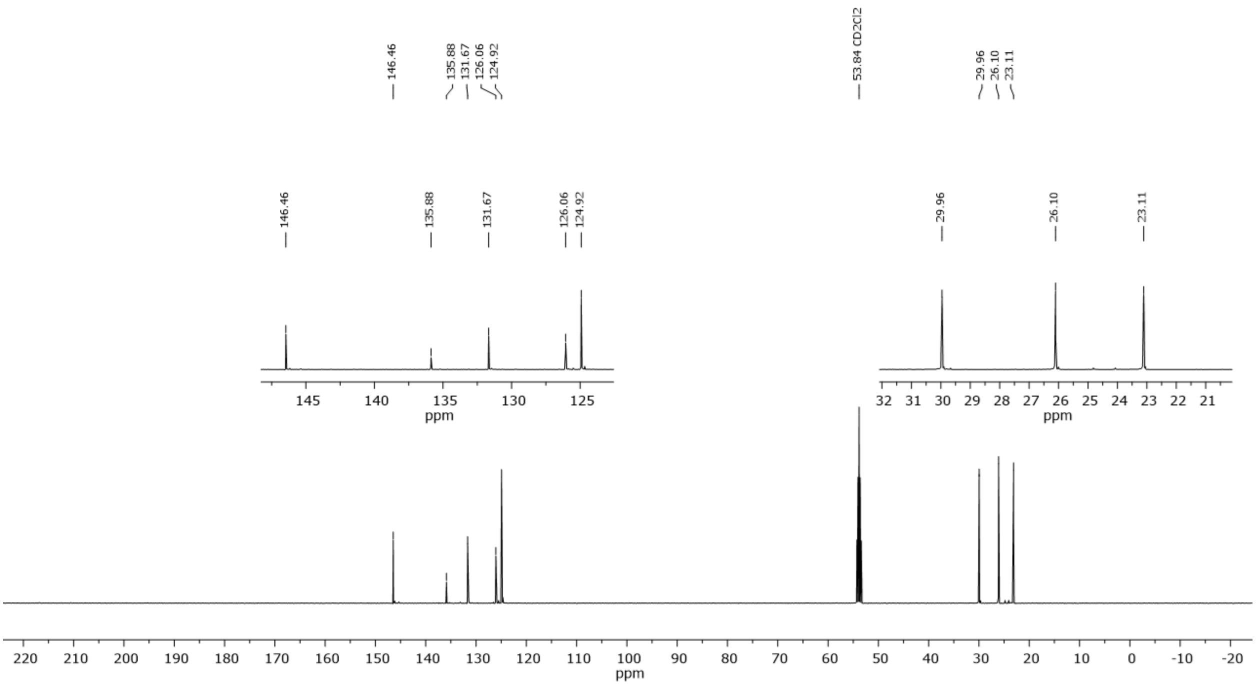

Figure S50: $^{13}C\{^1H\}$ NMR spectrum of BI_3·IDipp (125.8 MHz, CD_2Cl_2).

2.18. NMR spectra of $Me_2S{\cdot}I_2B–C_2H_4–SiI_3$ ($5{\cdot}SMe_2$)

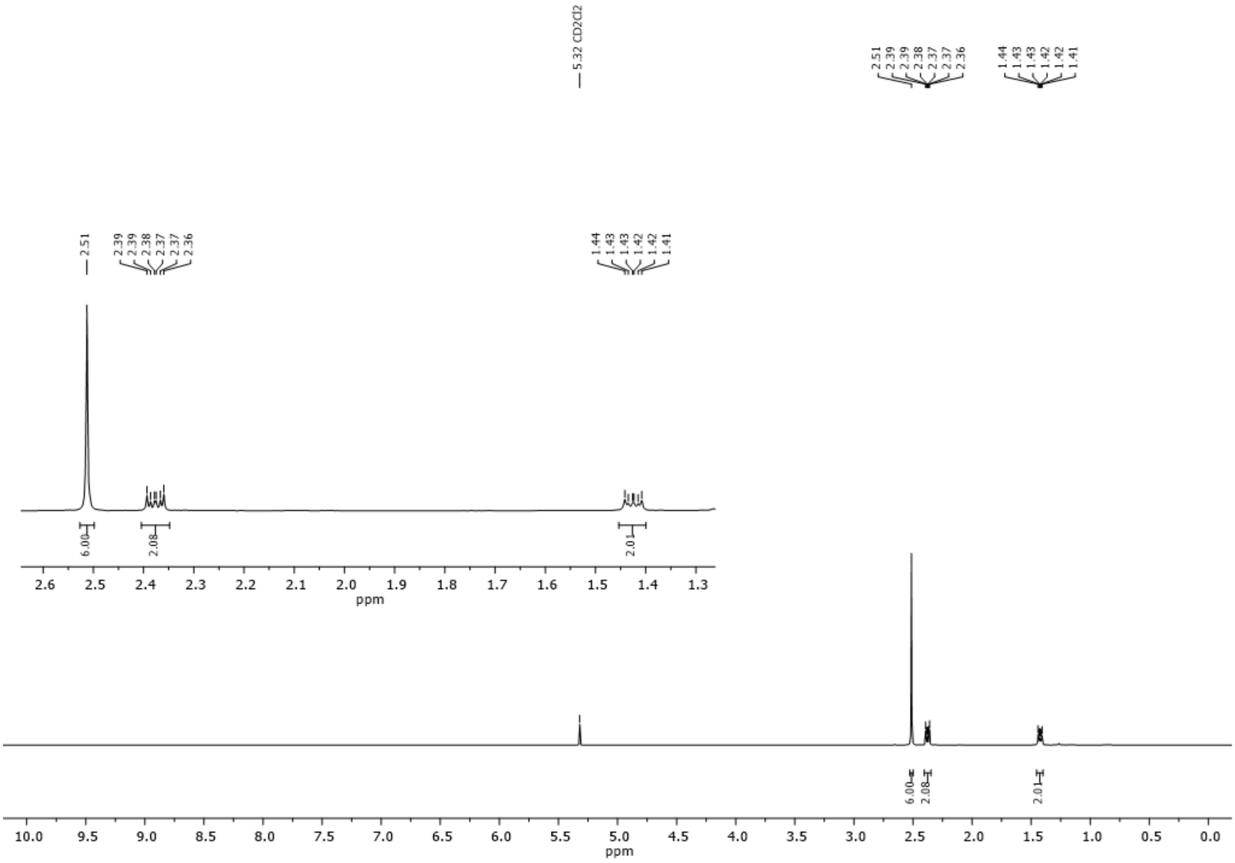

Figure S51: ^{1}H NMR spectrum of **5**·SMe_2 (500.2 MHz, CD_2Cl_2).

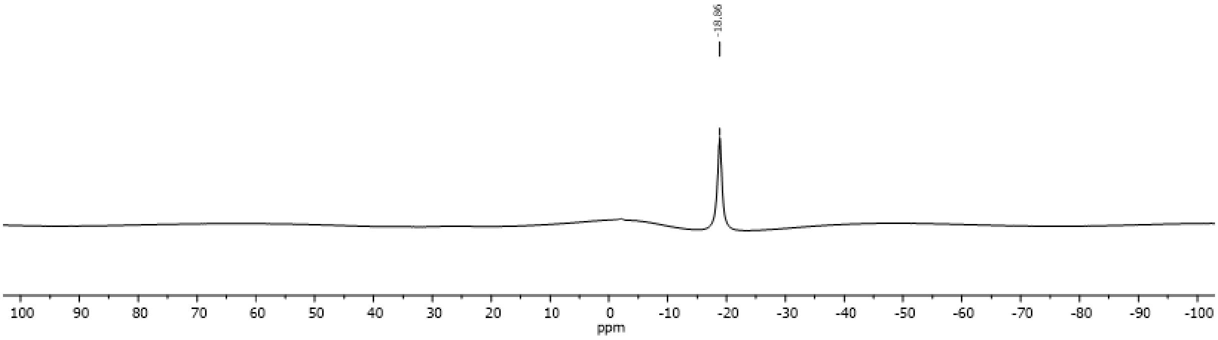

Figure S52: ^{11}B NMR spectrum of **5**·SMe_2 (160.5 MHz, CD_2Cl_2).

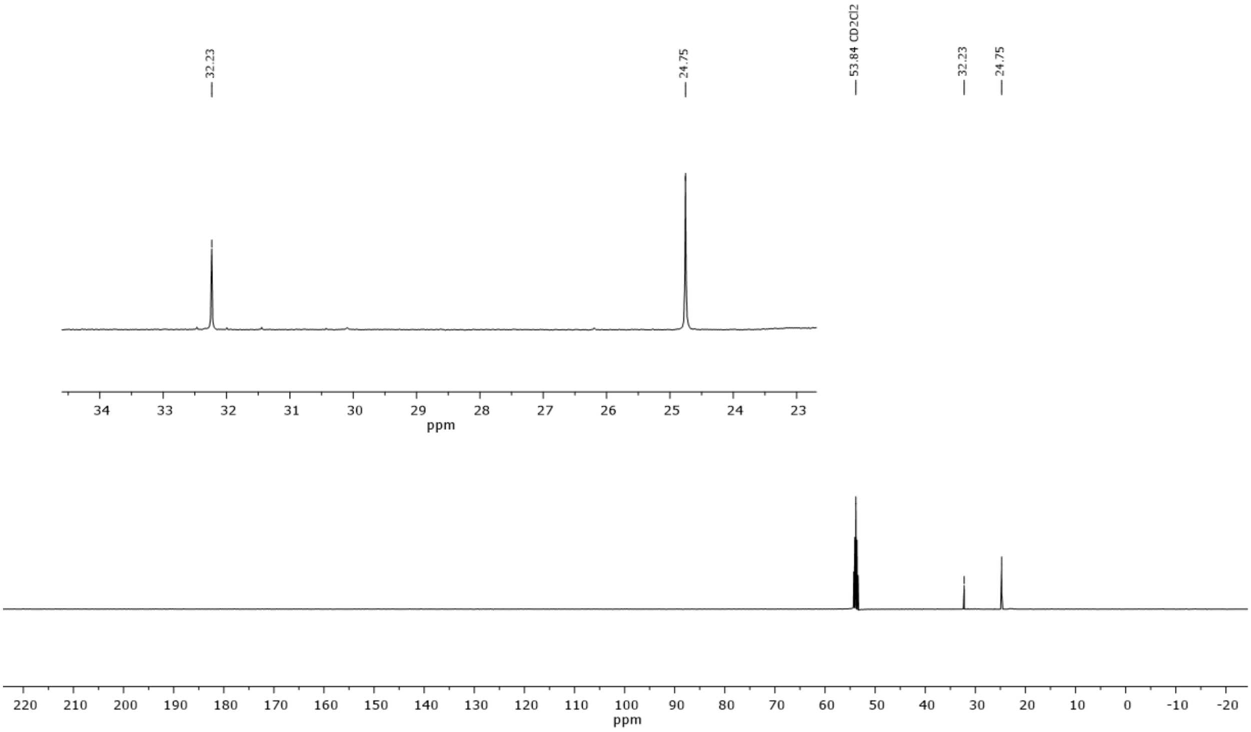

Figure S53: $^{13}C\{^{1}H\}$ NMR spectrum of **5**·SMe_2 (125.8 MHz, CD_2Cl_2).

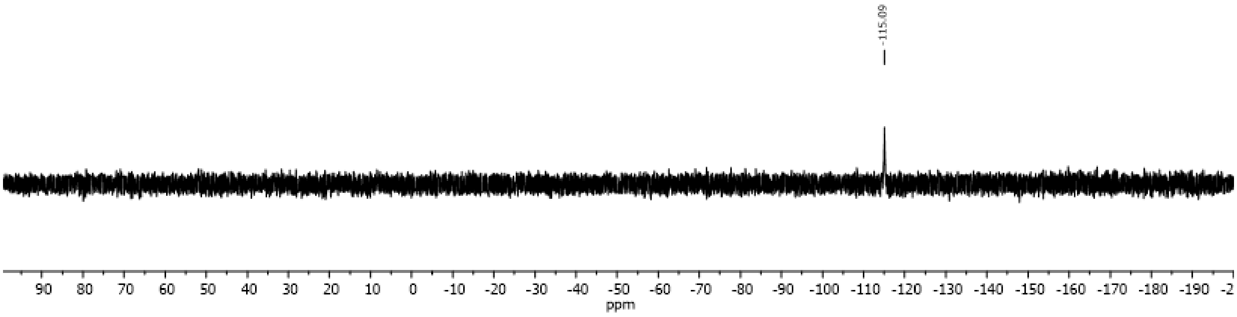

Figure S54: ^{29}Si NMR spectrum of **5**·SMe_2 (99.4 MHz, CD_2Cl_2).

2.19. NMR spectra of $Py{\cdot}I_2B–C_2H_4–SiI_3$ (5·Py)

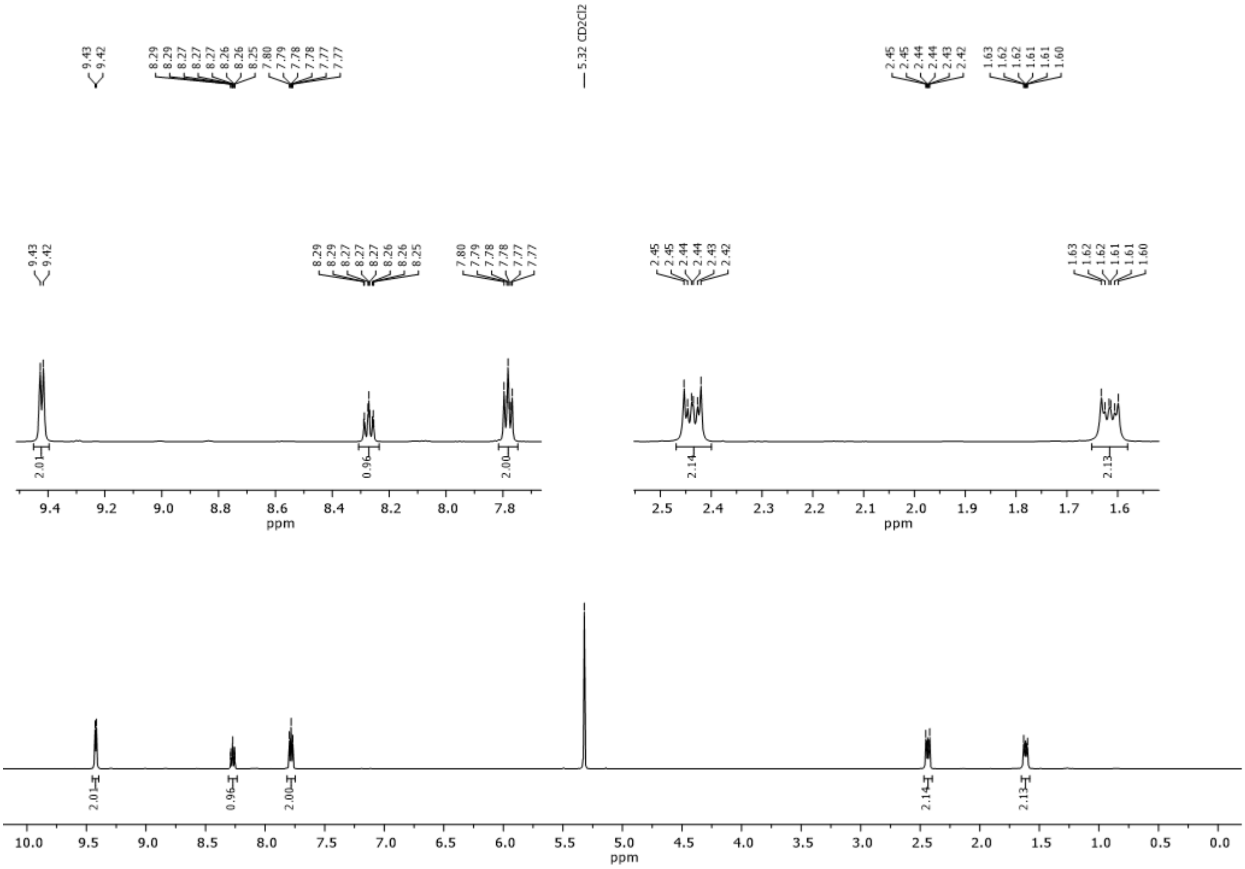

Figure S55: ^{1}H NMR spectrum of **5**·Py (500.2 MHz, CD_2Cl_2).

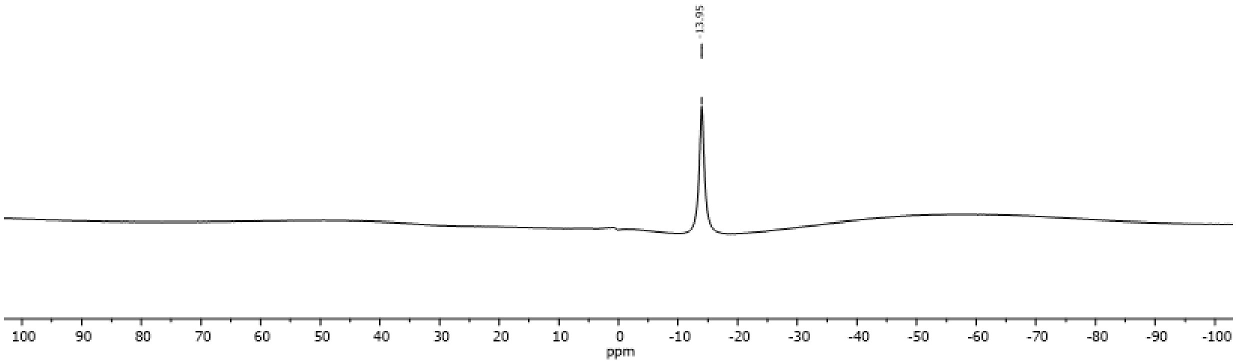

Figure S56: ^{11}B NMR spectrum of **5**·Py (160.5 MHz, CD_2Cl_2).

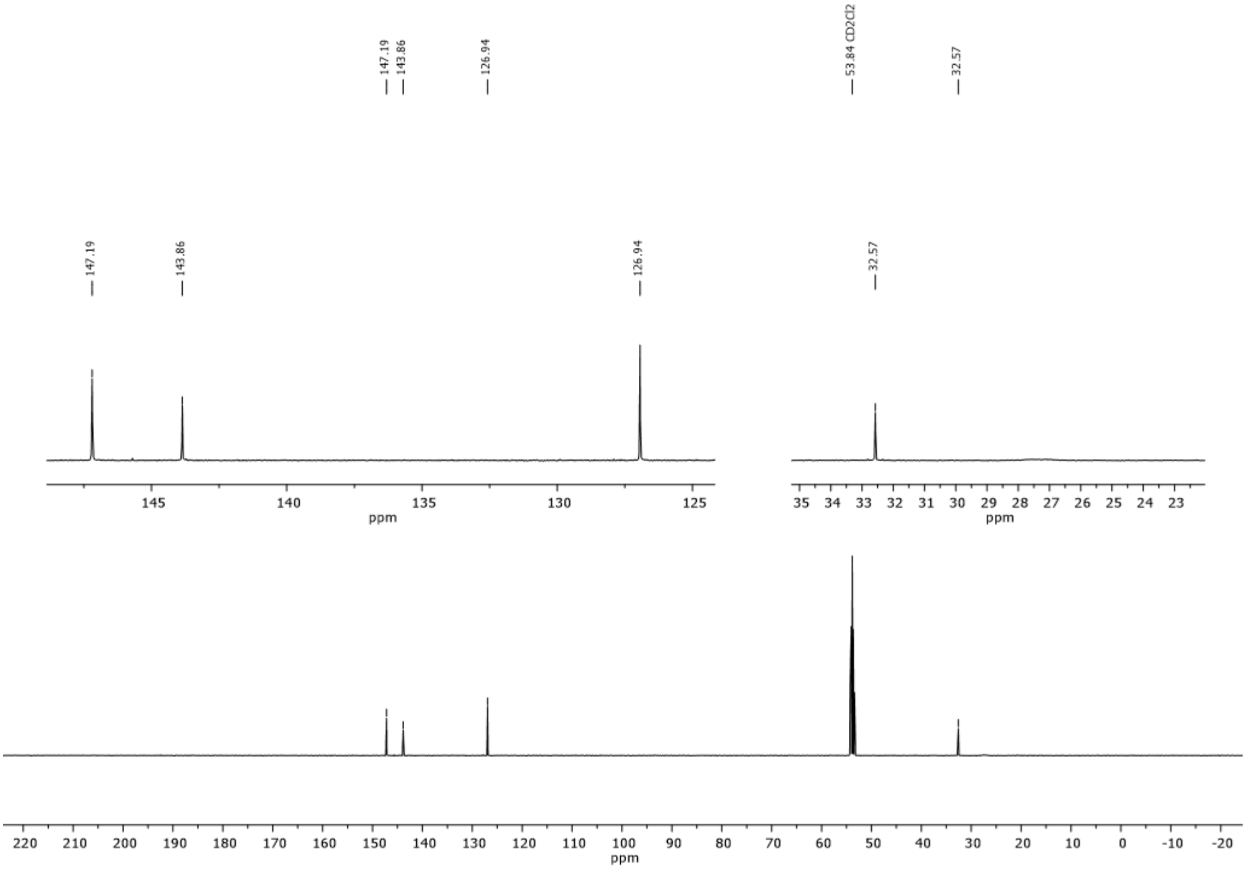

Figure S57: $^{13}C\{^1H\}$ NMR spectrum of **5**·Py (125.8 MHz, CD_2Cl_2).

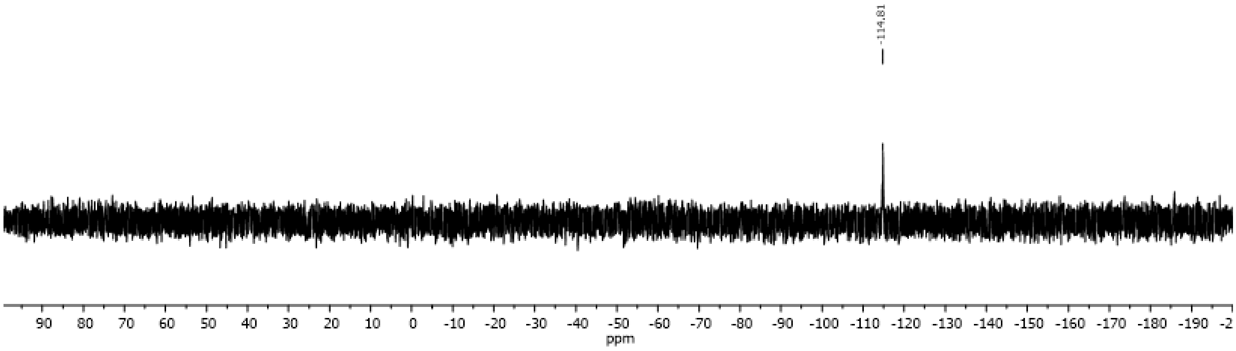

Figure S58: ^{29}Si NMR spectrum of **5**·Py (99.4 MHz, CD_2Cl_2).

2.20. NMR spectra recorded on the reaction mixture of $Ph_3P{\cdot}I_2B{-}SiI_3$ (**2**·PPh_3) with ethylene

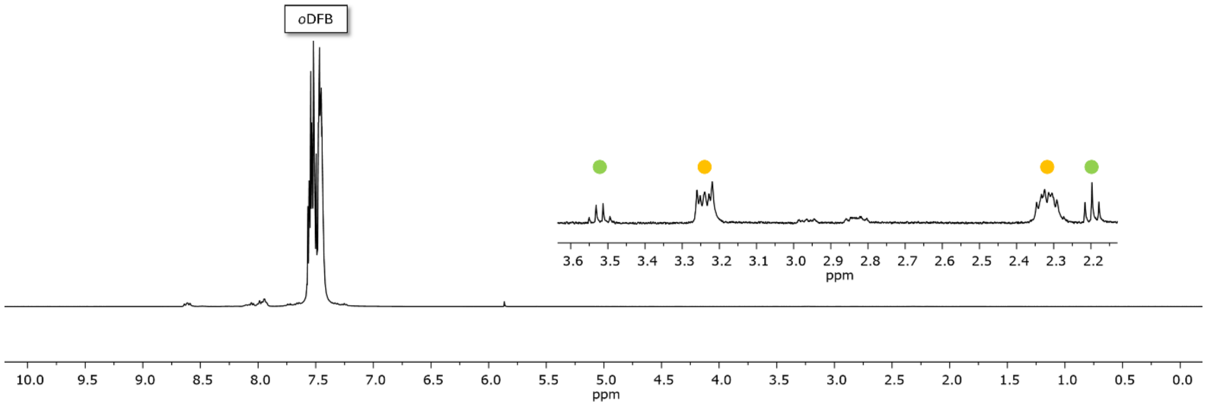

Figure S59: ^{1}H NMR spectrum of the reaction mixture of **2**·PPh_3 with ethylene, recorded after heating to 120 °C for 20 d (400.3 MHz, *o*DFB). *Note:* The signal set marked in green corresponds to $[Et_4N]$[**1**], while the set of signals marked in orange is assumed to belong to the corresponding silaboration product of the reaction.

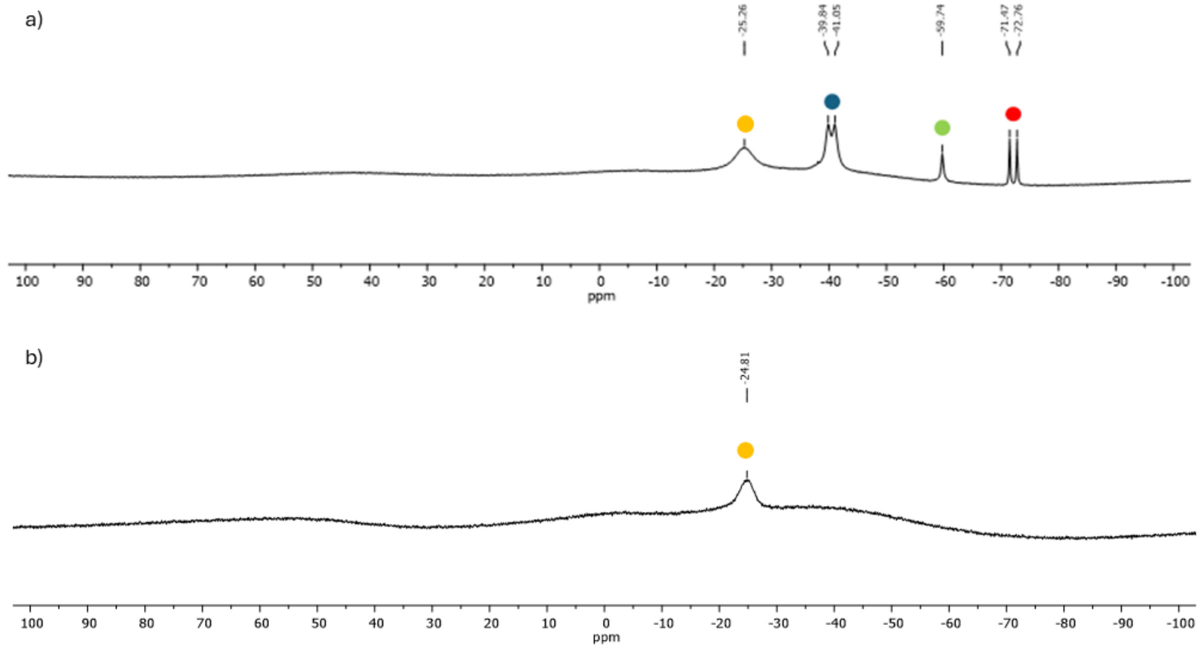

Figure S60: (a) ^{11}B NMR spectrum of the reaction mixture of **2**·PPh_3 with ethylene, recorded after heating to 120 °C for 20 d (96.3 MHz, *o*DFB). *Note:* The spectrum shows the signal of **2**·PPh_3 (blue), of $[Et_4N]$[**1**] (green), and of $BI_3{\cdot}PPh_3$ (red). The signal at –25.3 ppm (orange) corresponds to the ethylene silaboration product. (b) ^{11}B NMR spectrum of the reaction mixture of **5**·SMe_2 with PPh_3, recorded after 1 h at rt (96.3 MHz, *o*DFB). *Note:* The spectrum shows a signal at –24.8 ppm (orange), assignable to **5**·PPh_3.

2.21. NMR spectrum recorded on the reaction mixture of IDipp·I_2B–SiI_3 (2·IDipp) with ethylene

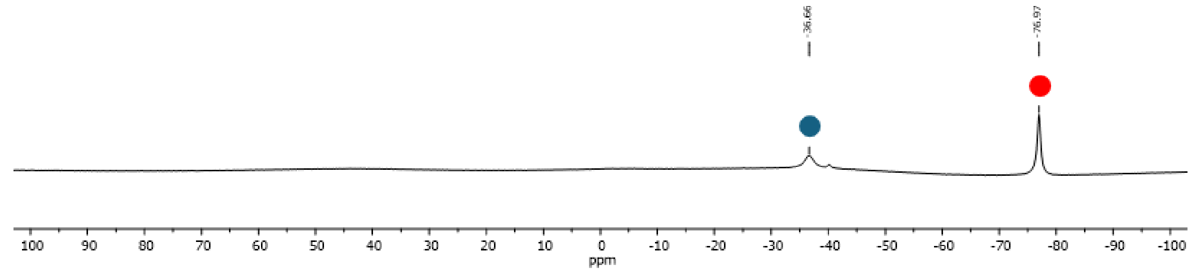

Figure S61: ^{11}B NMR spectrum of the reaction mixture of **2**·IDipp with ethylene, recorded after heating to 100 °C for 6 d (96.3 MHz, *o*DFB). *Note:* The spectrum shows a signal for **2**·IDipp (blue) and a signal for BI_3·IDipp (red).

2.22. NMR spectra recorded on the reaction mixture of IDipp·I_2B–SiI_3 (2·IDipp) with 2,3-dimethyl-1,3-butadiene

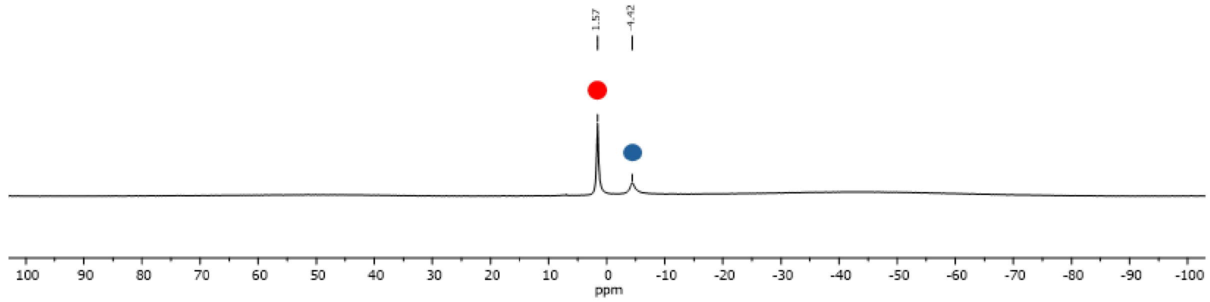

Figure S62: ^{11}B NMR spectrum of the reaction mixture of **2**·IDipp with 2,3-dimethyl-1,3-butadiene, recorded after heating to 100 °C for 8 d (96.3 MHz, CD_2Cl_2). *Note:* The spectrum shows a signal for IDipp·Cl_2B–SiCl_3 (**3**·IDipp; blue) and a signal for BCl_3·IDipp (red).[S13]

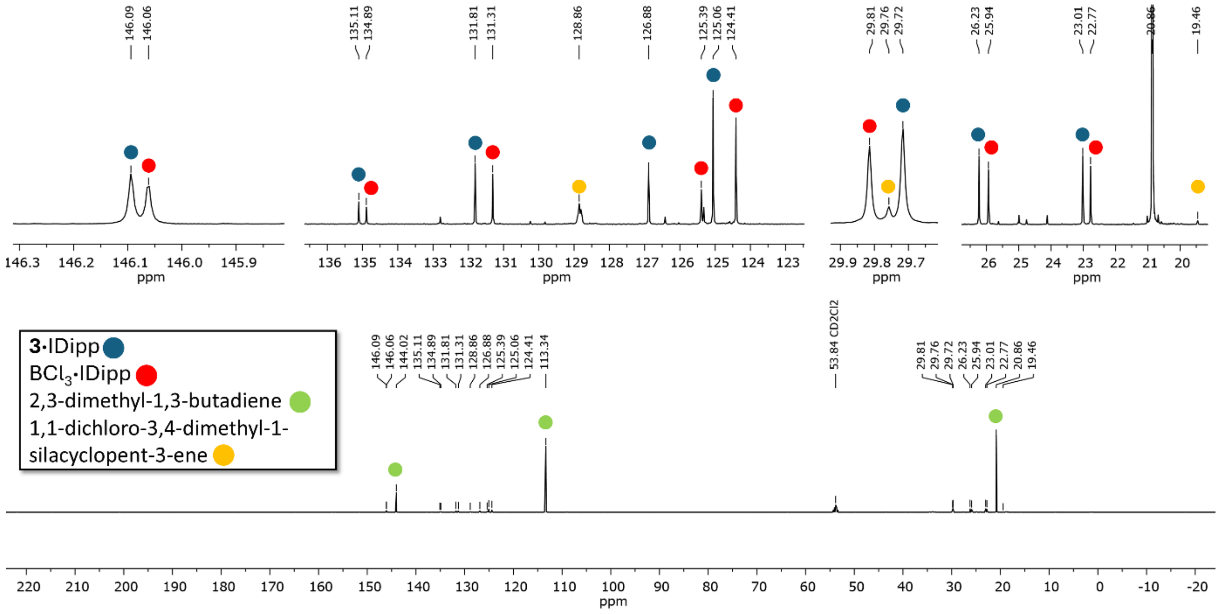

Figure S63: ^{13}C NMR spectrum of the reaction mixture of **2**·IDipp with 2,3-dimethyl-1,3-butadiene, recorded after heating to 100 °C for 8 d (125.8 MHz, CD_2Cl_2). *Note:* The spectrum shows signals corresponding to **3**·IDipp, BCl_3·IDipp,[S13] 2,3-dimethyl-1,3-butadiene,[S24,25] and 1,1-dichloro-3,4-dimethyl-1-silacyclopent-3-ene.[S14–16]

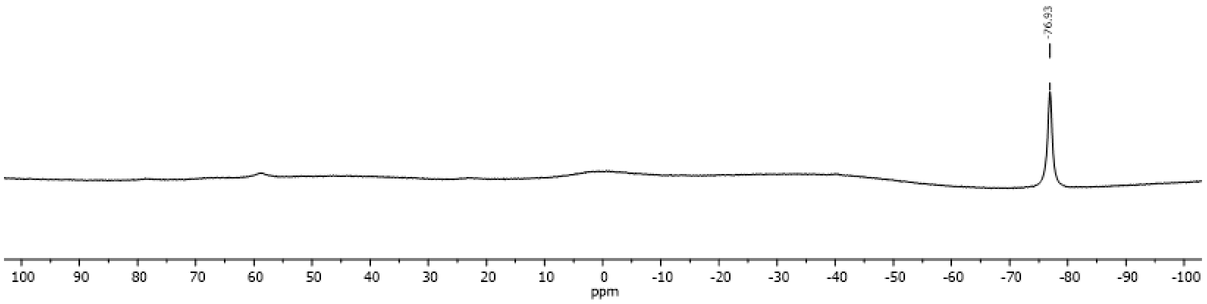

Figure S64: ^{11}B NMR spectrum of the reaction mixture of **2**·IDipp with 2,3-dimethyl-1,3-butadiene, recorded after heating to 100 °C for 10 d (96.3 MHz, *o*DFB).

2.23. NMR spectra recorded on the reaction mixture of $Me_2S{\cdot}Cl_2B–SiCl_3$ (3·SMe_2) with ethylene

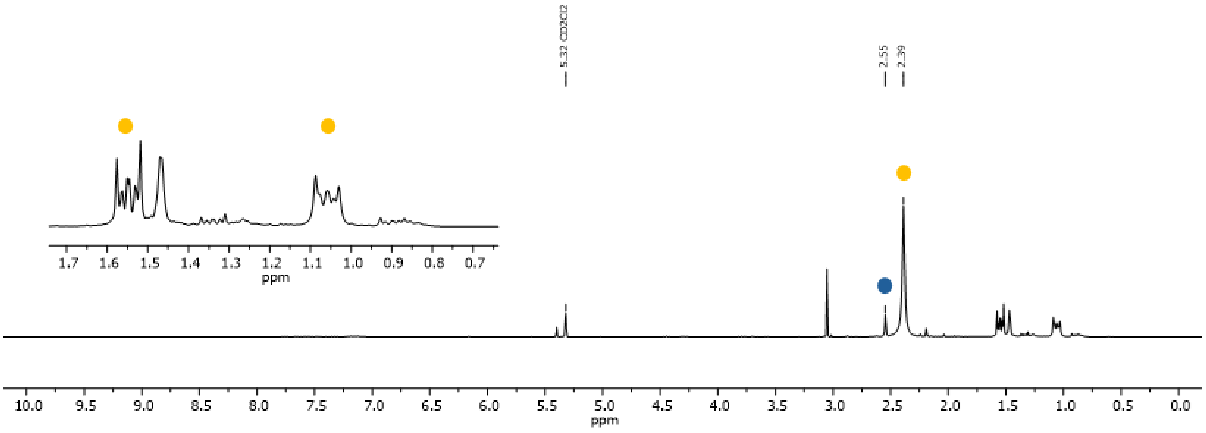

Figure S65: ^{1}H NMR spectrum of the reaction mixture of **3**·SMe_2 with ethylene, recorded after heating to 80 °C for 31 d (300.0 MHz, CD_2Cl_2). *Note:* The spectrum shows a set of signals in the alkyl region, which is diagnostic for a $Cl_2B–(C_2H_4)–SiCl_3$·Do moiety (orange); the signal of residual **3**·SMe_2 is also detectable (blue).

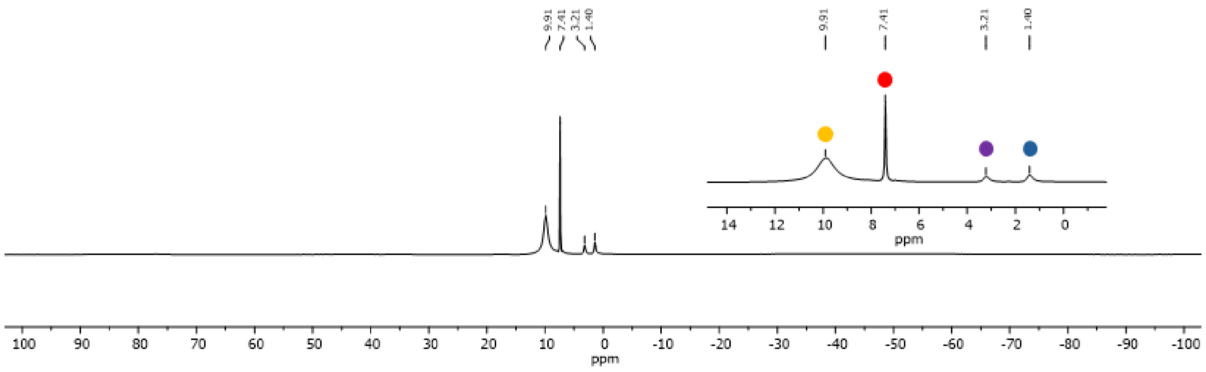

Figure S66: ^{11}B NMR spectrum of the reaction mixture of **3**·SMe_2 with ethylene, recorded after heating to 80 °C for 31 d (96.3 MHz, CD_2Cl_2). *Note:* The spectrum shows signals for **3**·SMe_2 (blue), a yet unknown compound (purple), and $BCl_3{\cdot}SMe_2$ (red).[S17] The signal at 9.9 ppm (orange) likely arises from the ethylene silaboration product.

2.24. NMR spectrum recorded on the reaction mixture of $Py{\cdot}Cl_2B{-}SiCl_3$ (3·Py) with ethylene

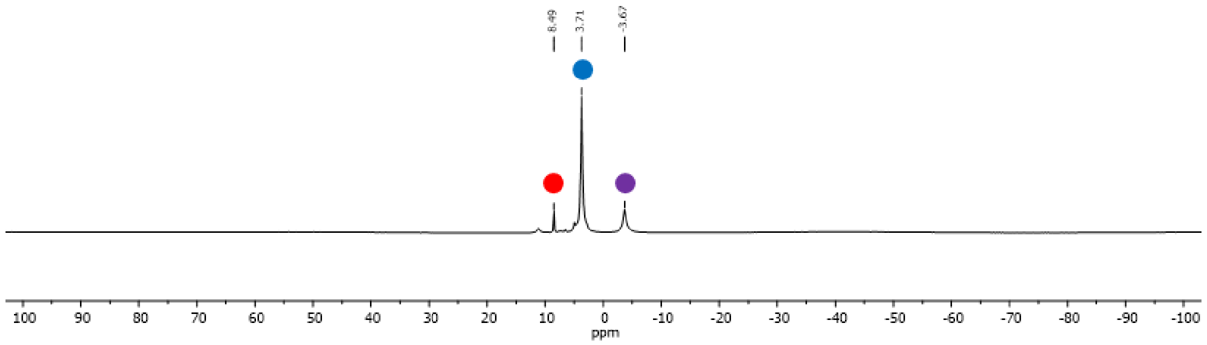

Figure S67: ^{11}B NMR spectrum of the reaction mixture of **3**·Py with ethylene, recorded after heating to 120 °C for 7 d, then to 140 °C for 1 d, and finally to 160 °C for 1 d (96.3 MHz, *o*DFB). *Note:* The spectrum shows signals for **3**·Py (blue), a yet unknown compound (purple), and for $BCl_3{\cdot}Py$ (red).[S18]

2.25. NMR spectra of $BCl_3{\cdot}Py$

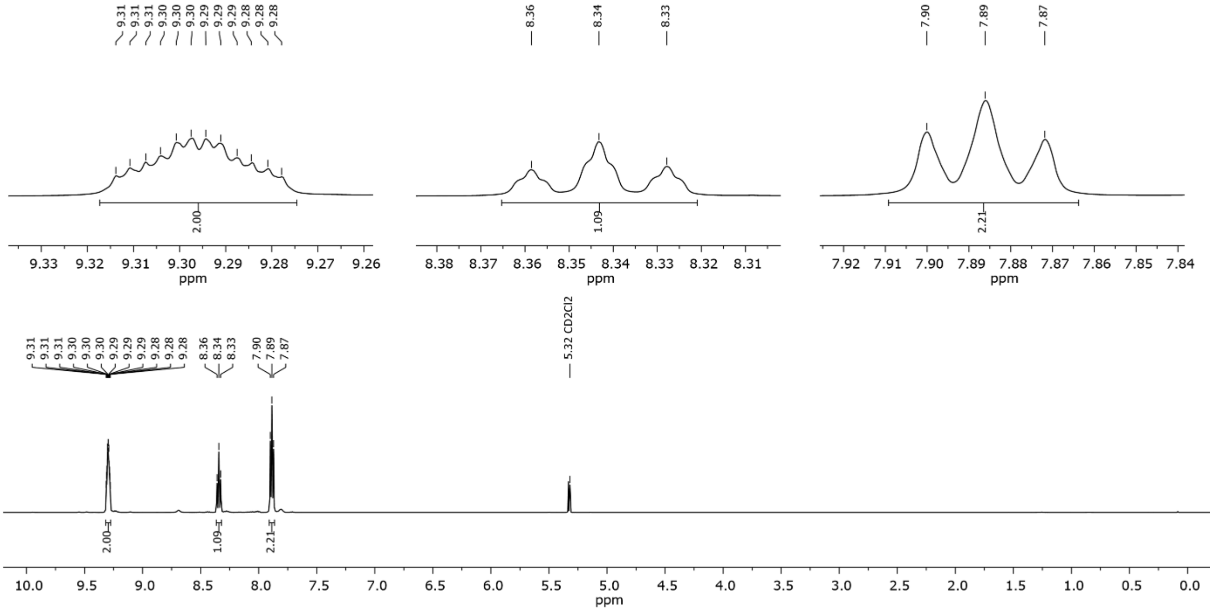

Figure S68: ^1H NMR spectrum of $BCl_3{\cdot}Py$ (500.2 MHz, CD_2Cl_2).

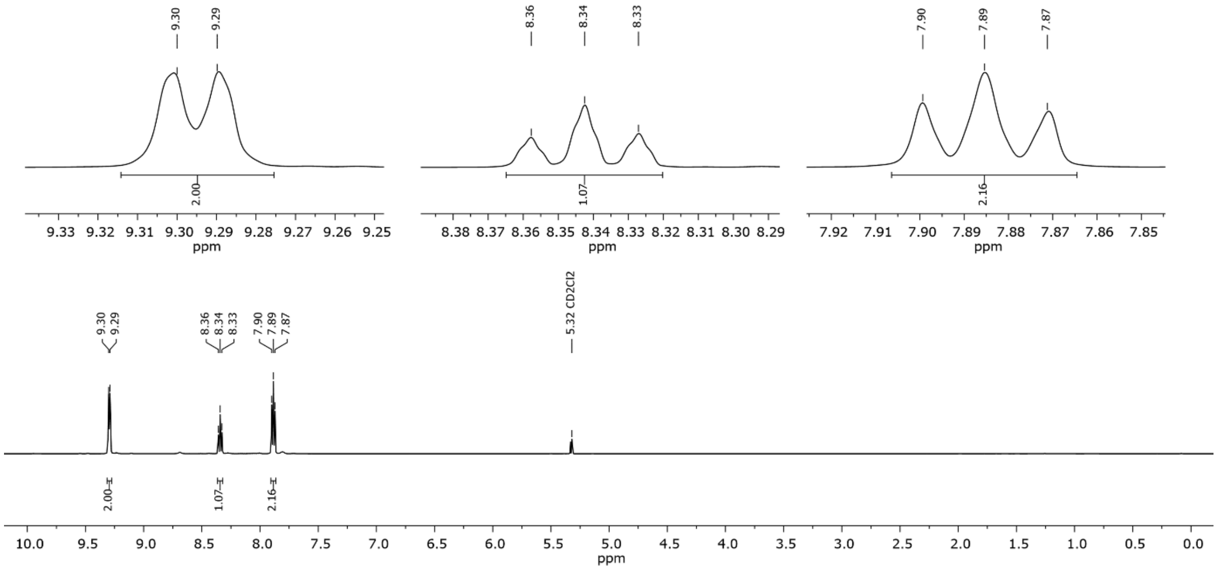

Figure S69: $^{1}H\{^{11}B\}$ NMR spectrum of BCl_3·Py (500.2 MHz, CD_2Cl_2).

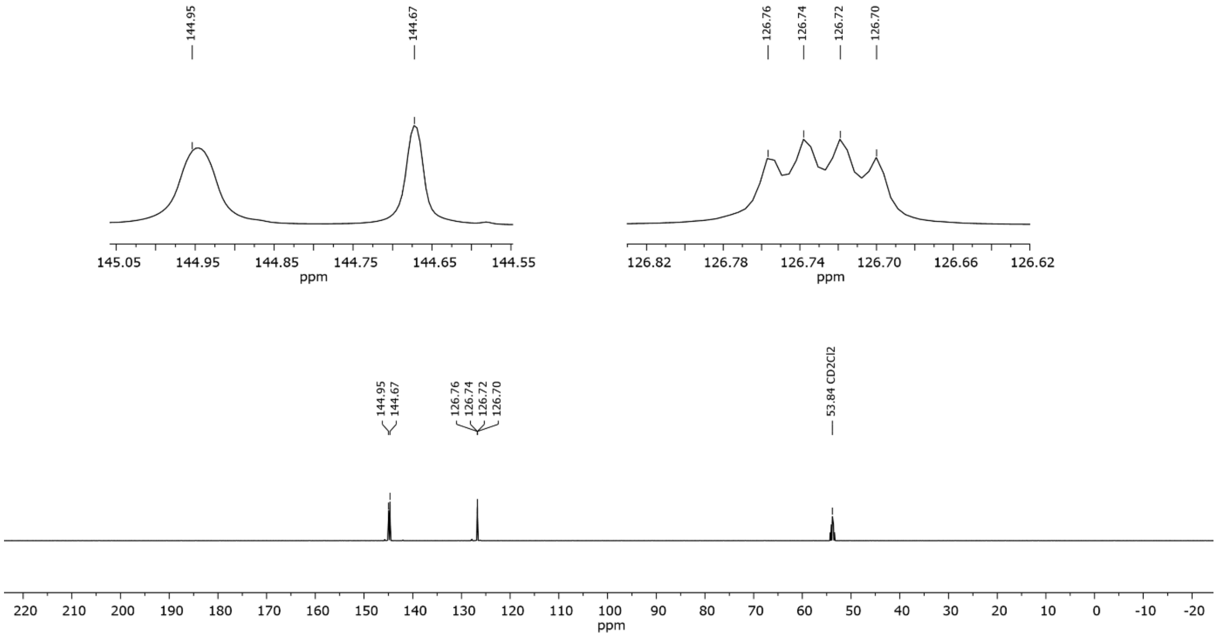

Figure S70: $^{13}C\{^{1}H\}$ NMR spectrum of BCl_3·Py (125.8 MHz, CD_2Cl_2).

2.26. NMR spectrum recorded on the reaction mixture of $Ph_3P{\cdot}Cl_2B–SiCl_3$ (**3**·PPh_3) with ethylene

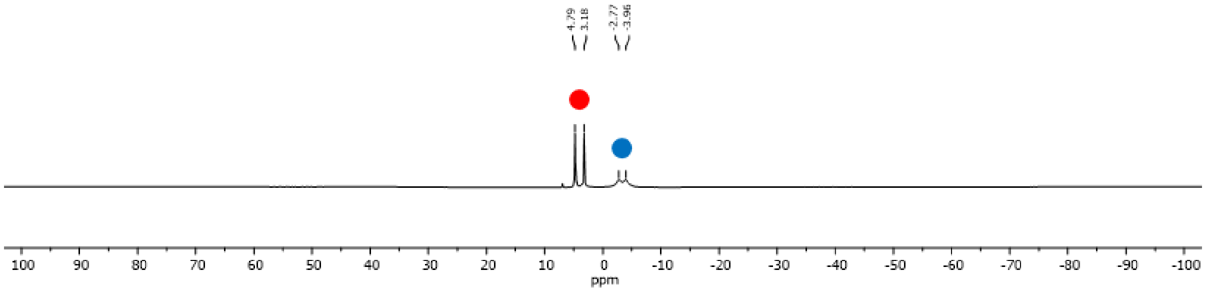

Figure S71: ^{11}B NMR spectrum of the reaction mixture of **3**·PPh_3 with ethylene, recorded after heating to 120 °C for 17 d (96.3 MHz, *o*DFB). *Note:* The spectrum shows signals for **3**·PPh_3 (blue) and $BCl_3{\cdot}PPh_3$ (red).[S19,20]

2.27. NMR spectrum recorded on the reaction mixture of IDipp·$Cl_2B–SiCl_3$ (**3**·IDipp) with ethylene

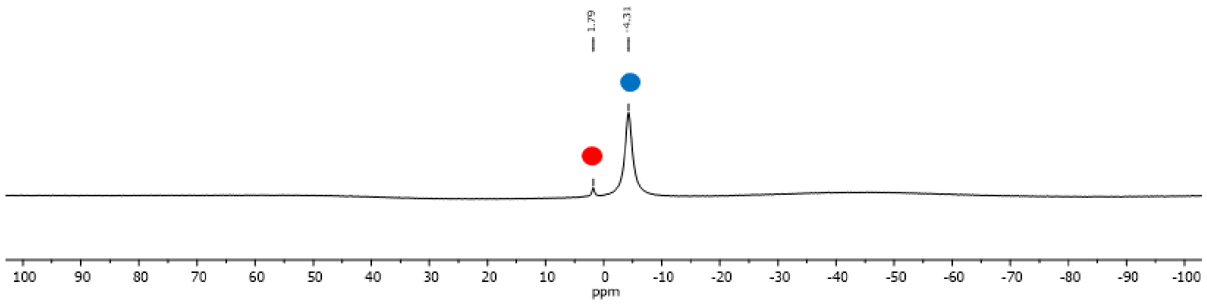

Figure S72: ^{11}B NMR spectrum of the reaction mixture of **3**·IDipp with ethylene, recorded after heating to 120 °C for 7 d, then to 140 °C for 1 d, and finally to 160 °C for 1 d (96.3 MHz, *o*DFB). *Note:* The spectrum shows signals for **3**·IDipp (blue) and BCl_3·IDipp (red).[S13]

2.29. NMR spectra of $(I_2B–)(I_3Si–)C_6H_{10}$ (7)

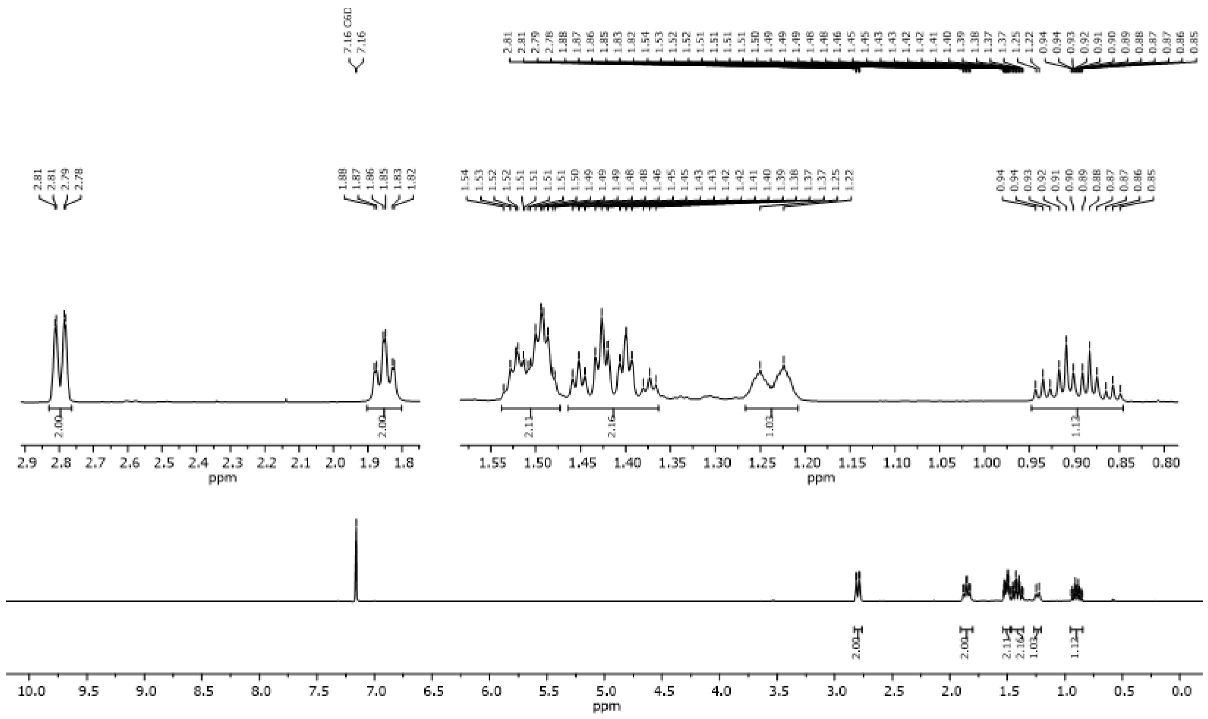

Figure S73: ^1H NMR spectrum of **7** (500.2 MHz, C_6D_6).

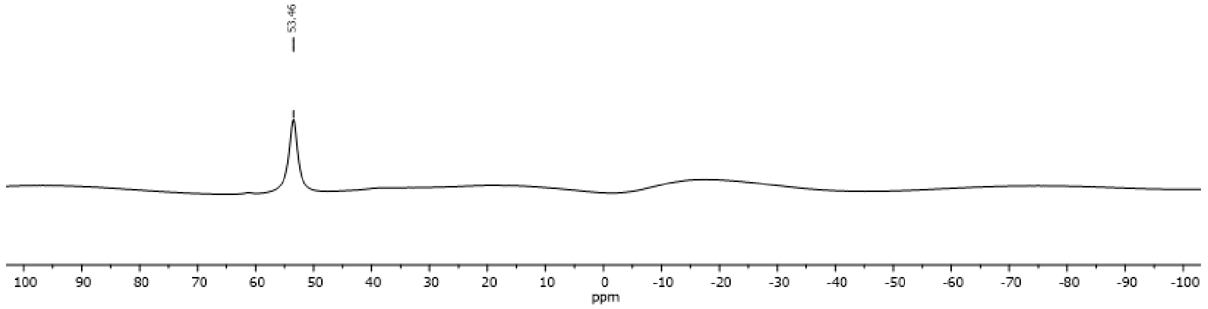

Figure S74: ^{11}B NMR spectrum of **7** (160.5 MHz, C_6D_6).

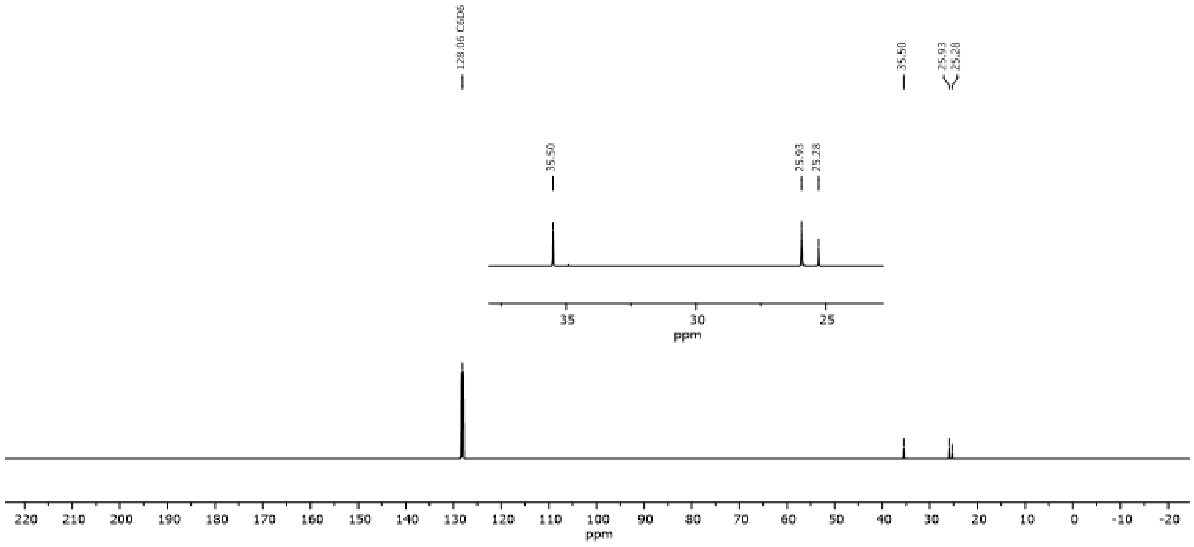

Figure S75: $^{13}C\{^1H\}$ NMR spectrum of **7** (125.8 MHz, C_6D_6).

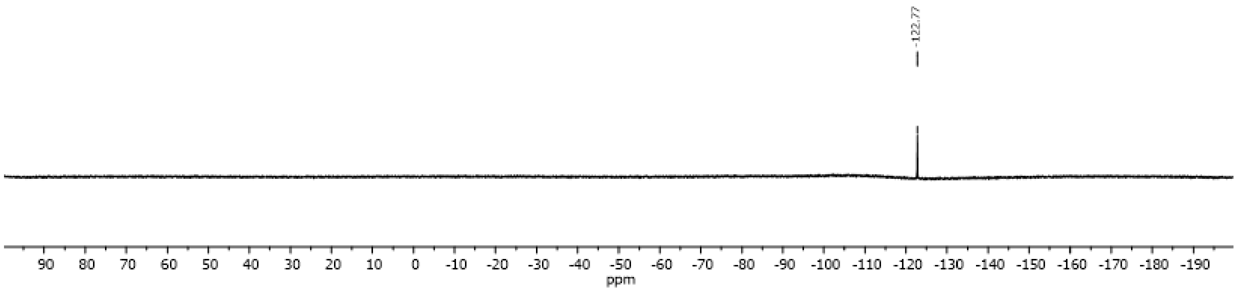

Figure S76: ^{29}Si NMR spectrum of **7** (99.4 MHz, C_6D_6).

2.30. NMR spectra recorded on the reaction mixture of $Me_2S{\cdot}I_2B–SiI_3$ (2·SMe_2) with cyclohexene

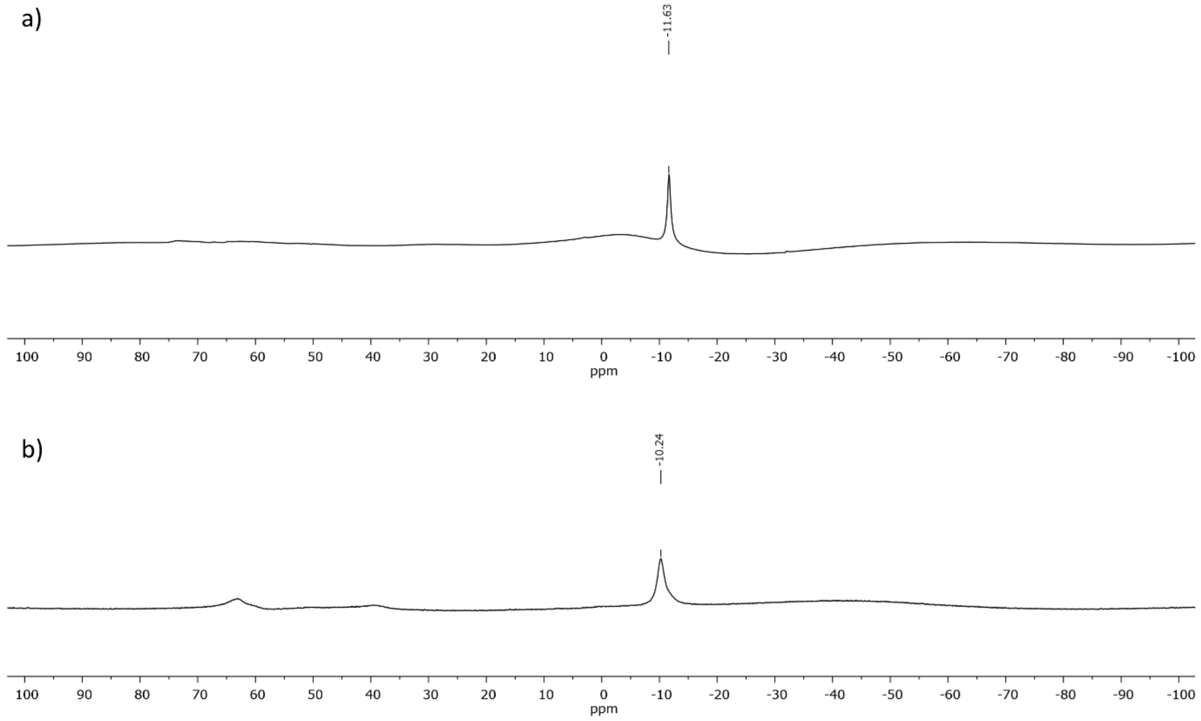

Figure S77: (a) ^{11}B NMR spectrum of the reaction mixture of **2**·SMe_2 with cyclohexene, recorded after heating to 120 °C for 24 d (160.5 MHz, *o*DFB). (b) ^{11}B NMR spectrum of the reaction mixture of **7** with SMe_2, recorded after 1 d at rt (96.3 MHz, *o*DFB).

2.31. NMR spectra of $Me_2S{\cdot}I_2B{-}C_2H_4{-}I$

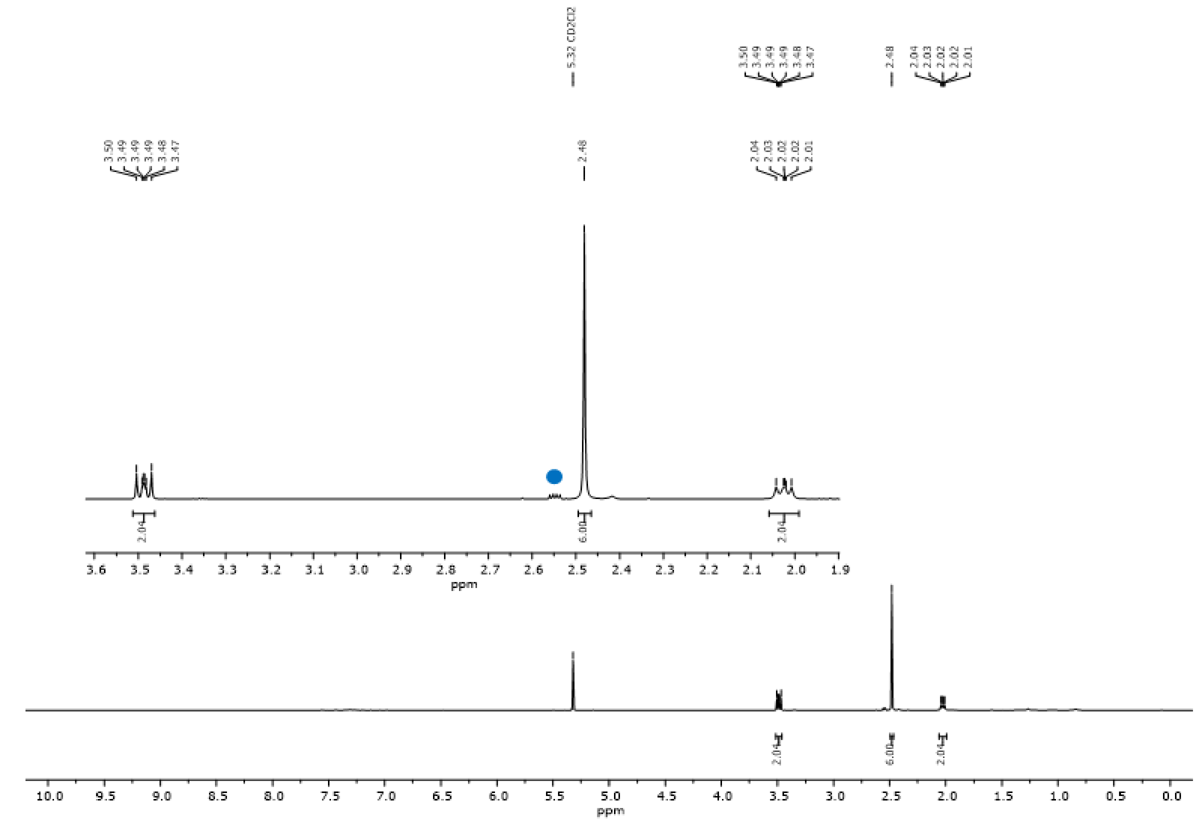

Figure S78: ^{1}H NMR spectrum of $Me_2S{\cdot}I_2B{-}C_2H_4{-}I$ (500.2 MHz, CD_2Cl_2). *Note:* Traces of the starting material, $BI_3{\cdot}SMe_2$, were found to co-crystallize with the product and remain detectable in the spectrum (blue), comprising approximately 5% of the sample as estimated from the integration of the NMR signals.

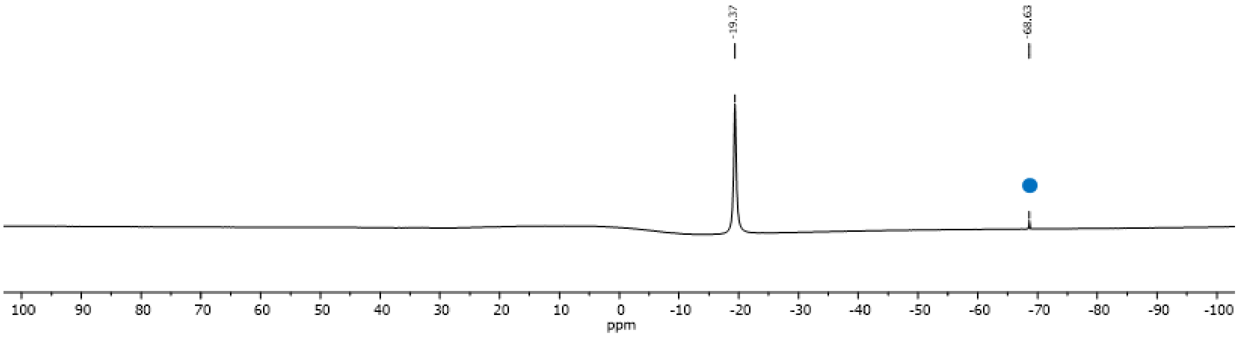

Figure S79: ^{11}B NMR spectrum of $Me_2S{\cdot}I_2B{-}C_2H_4{-}I$ (160.5 MHz, CD_2Cl_2). *Note:* Traces of the starting material, $BI_3{\cdot}SMe_2$, were found to co-crystallize with the product and remain detectable in the spectrum (blue).

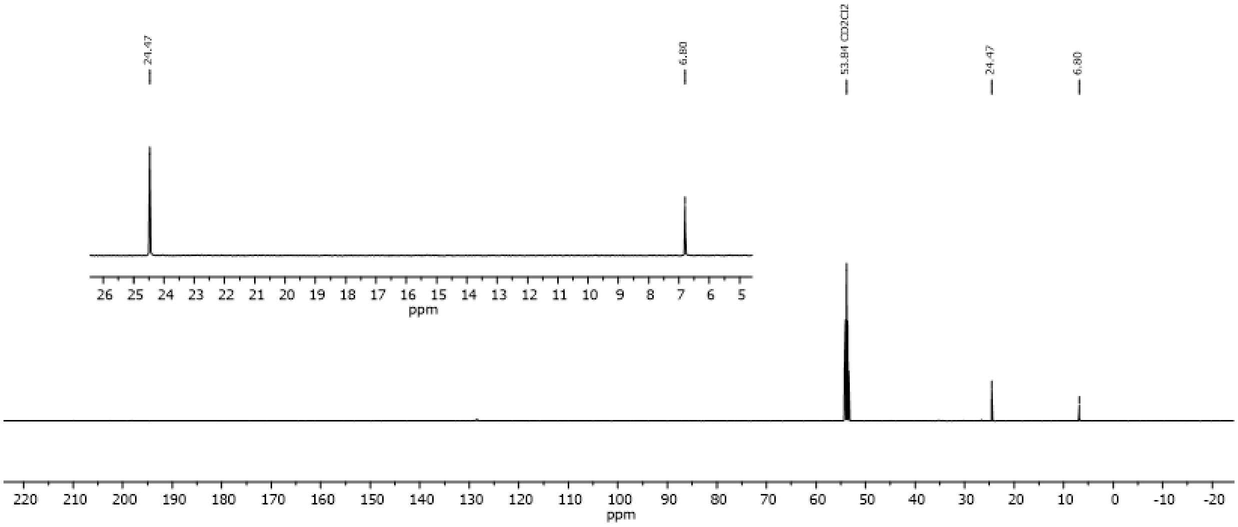

Figure S80: $^{13}C\{^{1}H\}$ NMR spectrum of $Me_2S{\cdot}I_2B{-}C_2H_4{-}I$ (125.8 MHz, CD_2Cl_2).

2.32. NMR spectra of 8

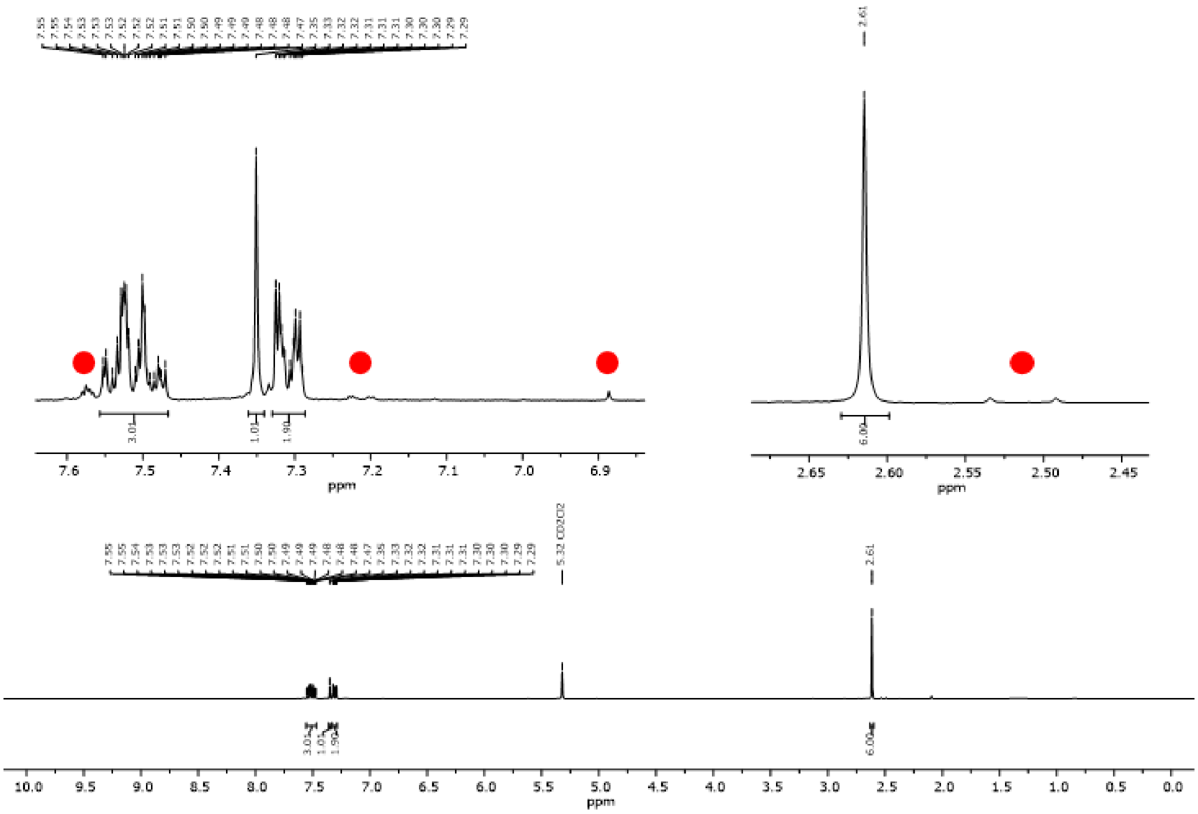

Figure S81: ^{1}H NMR spectrum of **8** (300.0 MHz, CD_2Cl_2). *Note:* The spectrum reveals the presence of a second compound, exhibiting a set of signals consistent with a species of similar connectivity as **8** (red). Integration of the NMR signals indicates that this component accounts for approximately 5% of the sample.

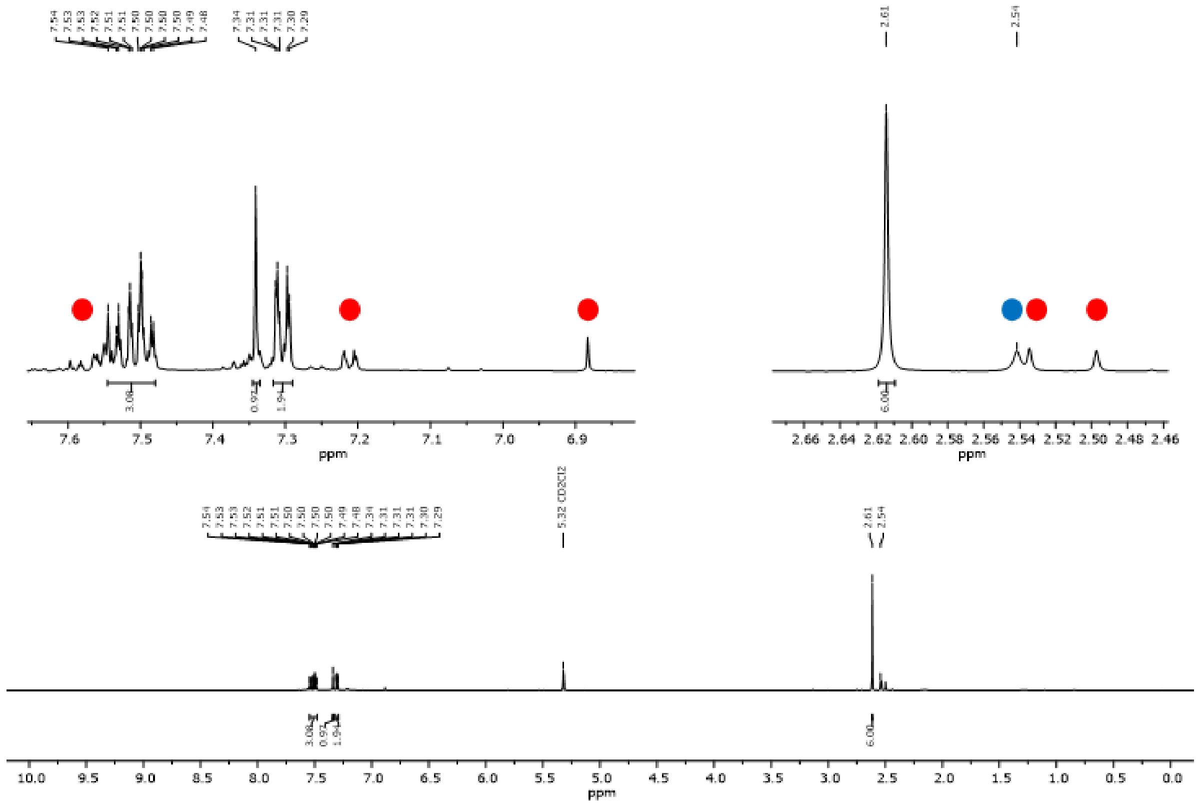

Figure S82: ^{1}H NMR spectrum of **8**, recorded after 1 d in solution (500.3 MHz, CD_2Cl_2). *Note:* The spectrum reveals the presence of a second compound, exhibiting a set of signals consistent with a species of similar connectivity as **8** (red) and a signal corresponding to the starting material **3**·SMe_2 (blue).

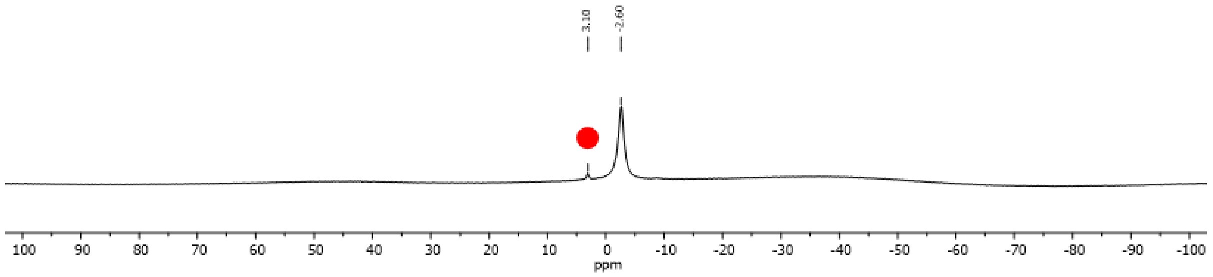

Figure S83: ^{11}B NMR spectrum of **8** (96.3 MHz, CD_2Cl_2). *Note:* The spectrum reveals the presence of a second compound (red).

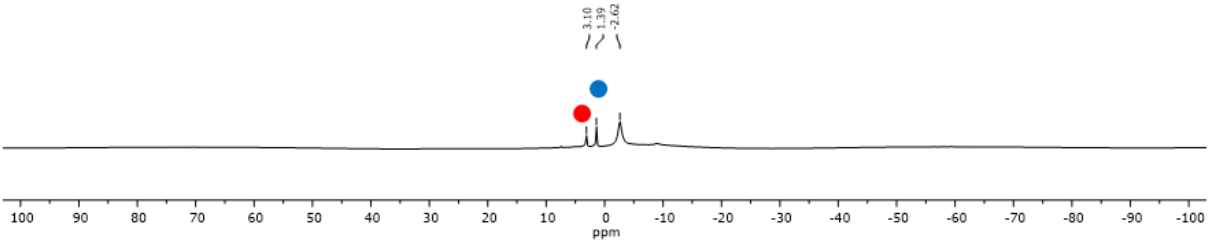

Figure S84: ^{11}B NMR spectrum of **8**, recorded after 1 d in solution (160.5 MHz, CD_2Cl_2). *Note:* The spectrum reveals the presence of a second compound (red) and a signal corresponding to the starting material **3**·SMe_2 (blue).

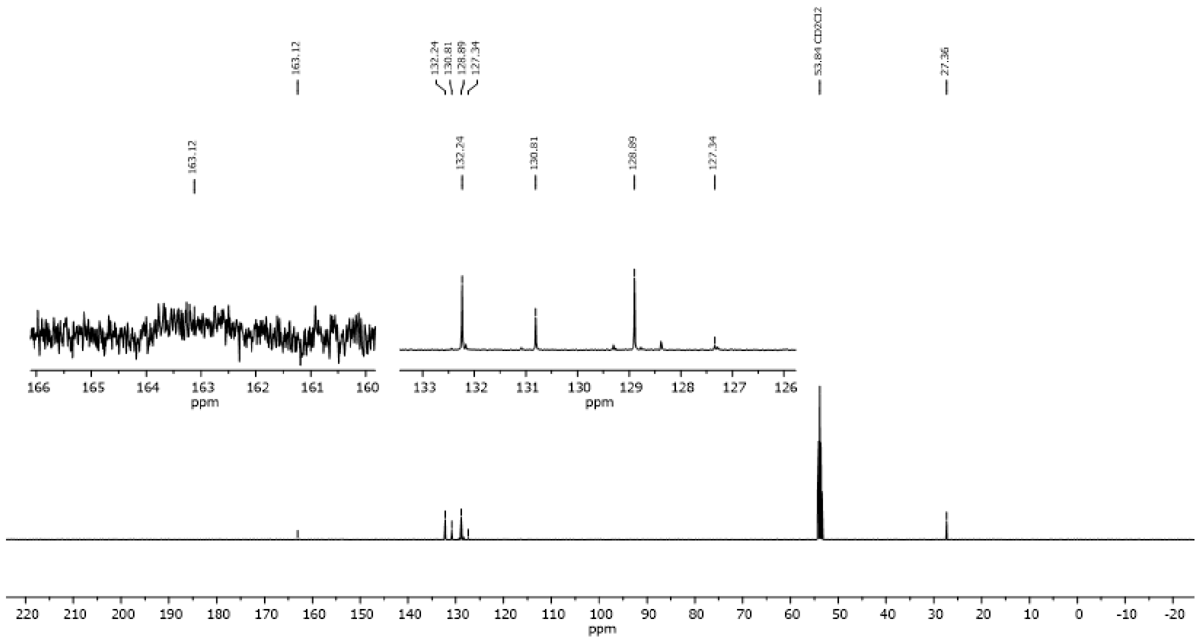

Figure S85: $^{13}C\{^{1}H\}$ NMR spectrum of **8** (125.8 MHz, CD_2Cl_2).

3. Plots of mass spectra

3.1. Mass spectrum of $Me_2S{\cdot}I_2B–SiI_3$ (2·SMe_2)

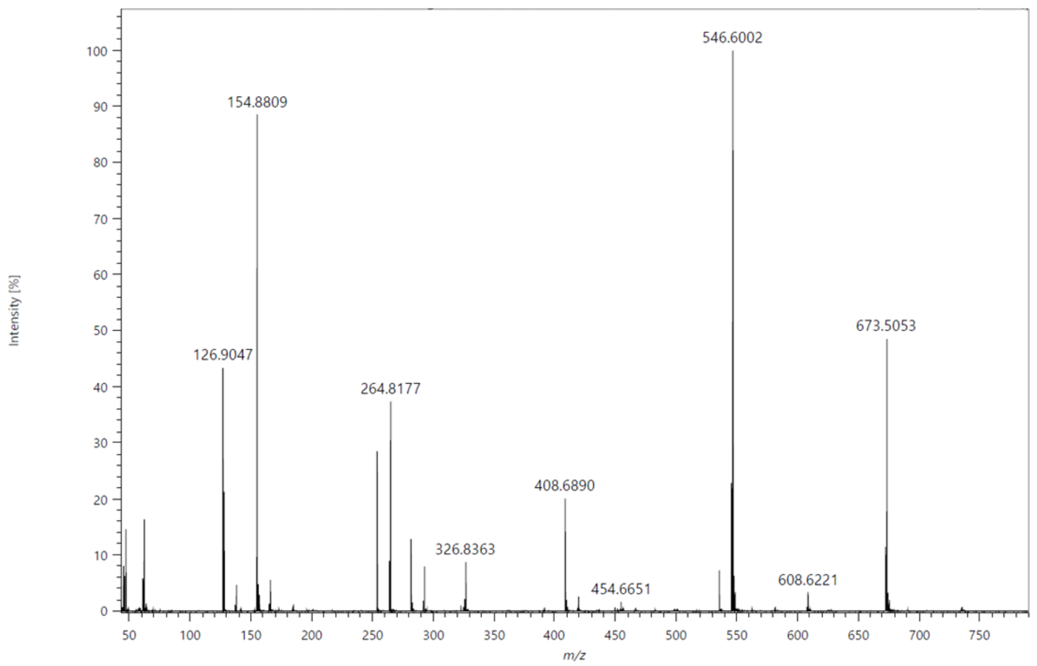

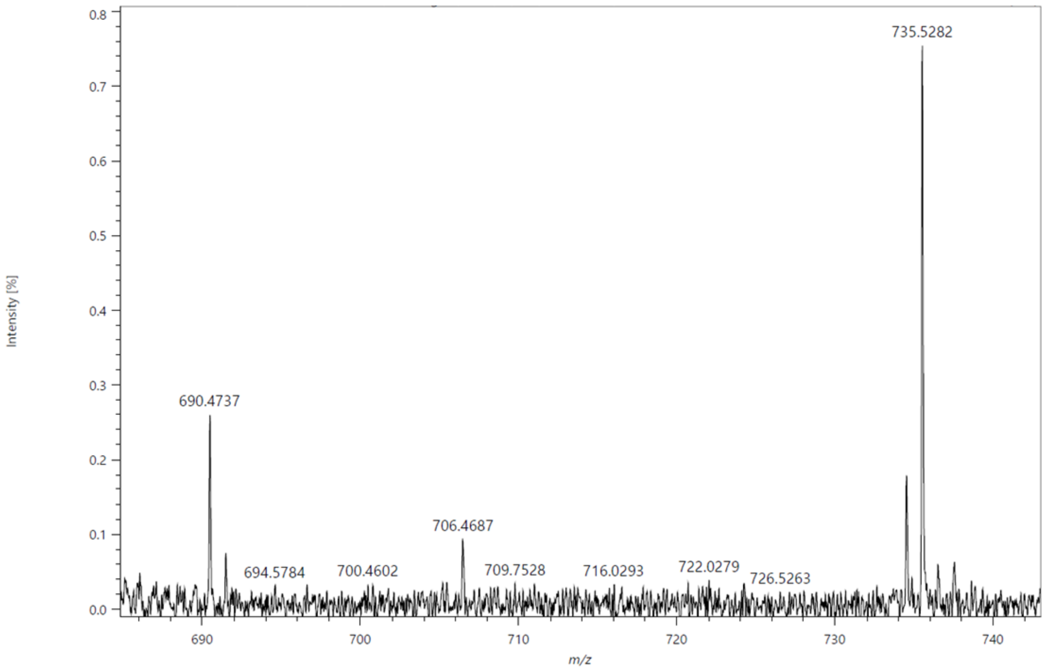

Figure S86: EI(+) mass spectrum of **2**·SMe_2. (top: full spectrum; bottom: section of the spectrum showing a signal at 735.5282 *m*/*z* corresponding to $[C_2H_6BI_5SSi]^{\bullet+}$).

3.2. Mass spectrum of Py·I_2B–SiI_3 (2·Py)

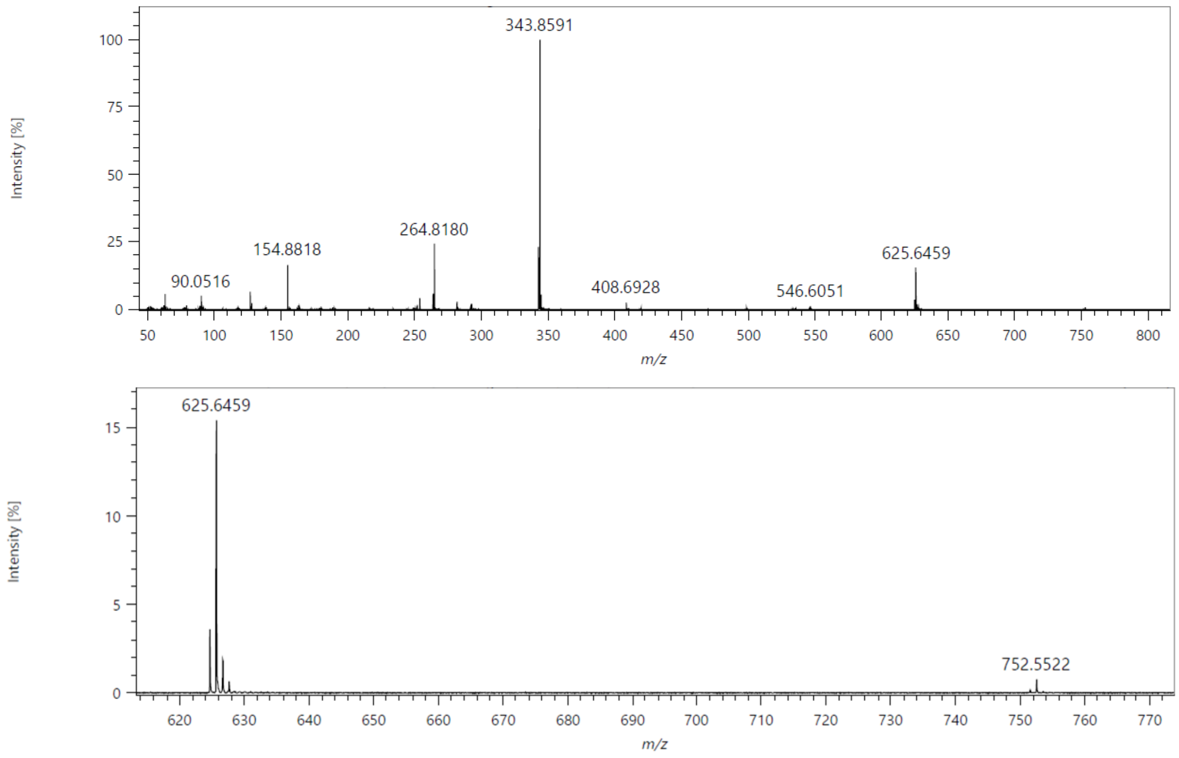

Figure S87: EI(+) mass spectrum of **2**·Py. (top: full spectrum; bottom: section of the spectrum showing a signal at 752.5522 *m*/*z* corresponding to $[C_5H_5BI_5NSi]^{\bullet+}$).

3.3. Mass spectrum of $Ph_3P{\cdot}I_2B{-}SiI_3$ (2·PPh_3)

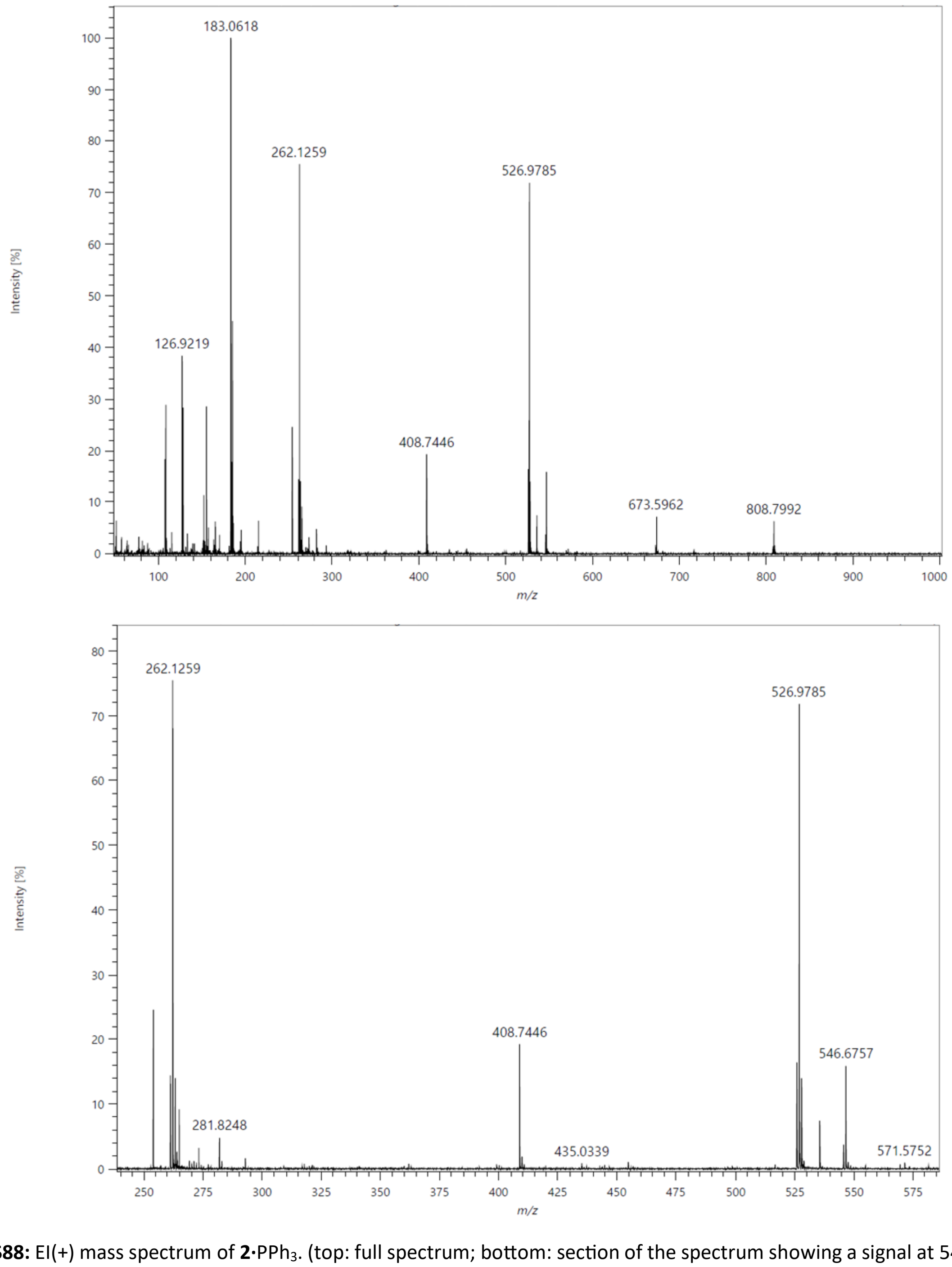

Figure S88: EI(+) mass spectrum of **2**·PPh_3. (top: full spectrum; bottom: section of the spectrum showing a signal at 546.6757 *m*/*z* corresponding to $[BI_4Si]^+$).

3.4. Mass spectrum of IDipp·I_2B–SiI_3 (2·IDipp)

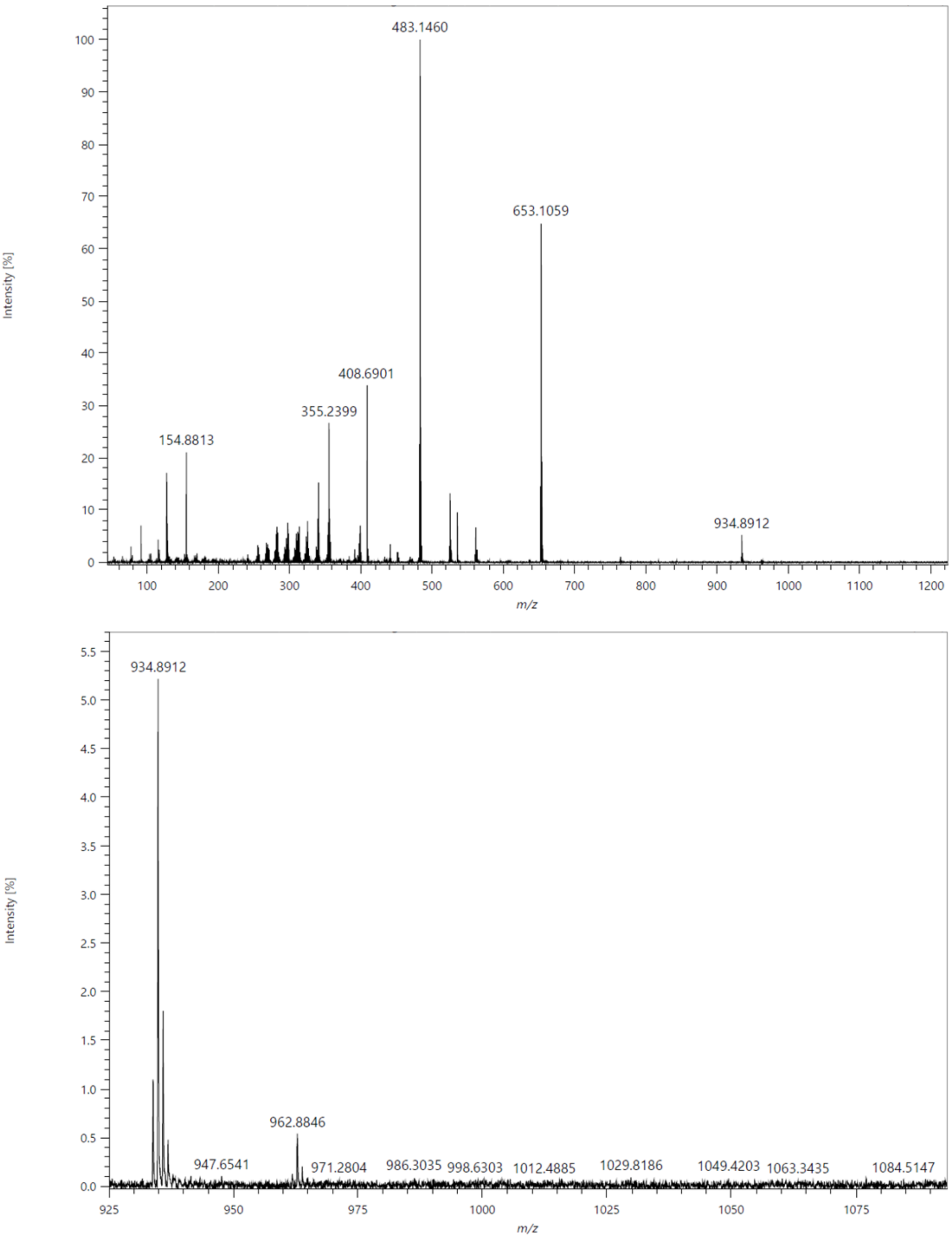

Figure S89: EI(+) mass spectrum of **2**·IDipp. (top: full spectrum; bottom: section of the spectrum showing a signal at 934.8912 *m*/*z* corresponding to $[C_{27}H_{36}BI_4N_2Si]^+$).

3.5. Mass spectrum of $Cl_2B–SiCl_3$ (3)

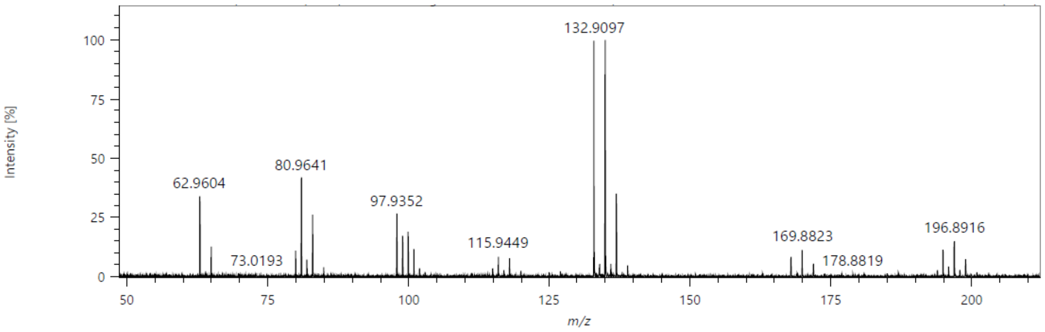

Figure S90: Section of the EI(+) mass spectrum of **3**.

3.6. Mass spectrum of $[Et_4N][Cl_3B–SiCl_3]$

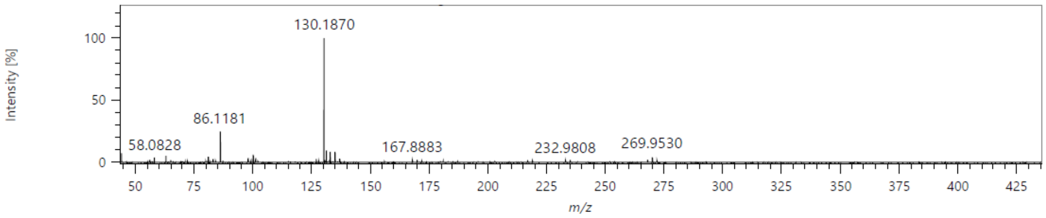

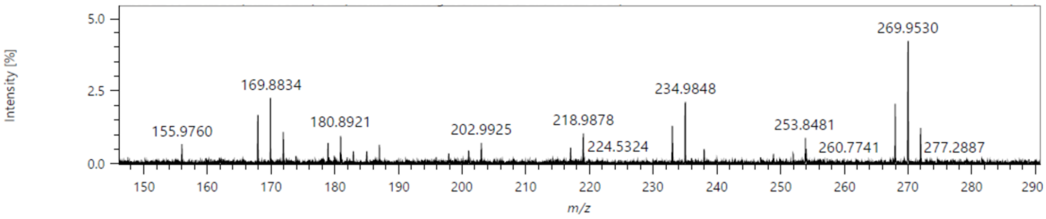

Figure S91: EI(+) mass spectrum of $[Et_4N][Cl_3B–SiCl_3]$. (top: full spectrum; bottom: section of the spectrum showing a signal at 180.8921 *m/z* corresponding to $[BCl_4Si]^+$ and a signal at 169.8834 *m/z* corresponding to $[Cl_4Si]^{\bullet+}$).

3.7. Mass spectrum of $Me_2S{\cdot}Cl_2B–SiCl_3$ (3·SMe₂)

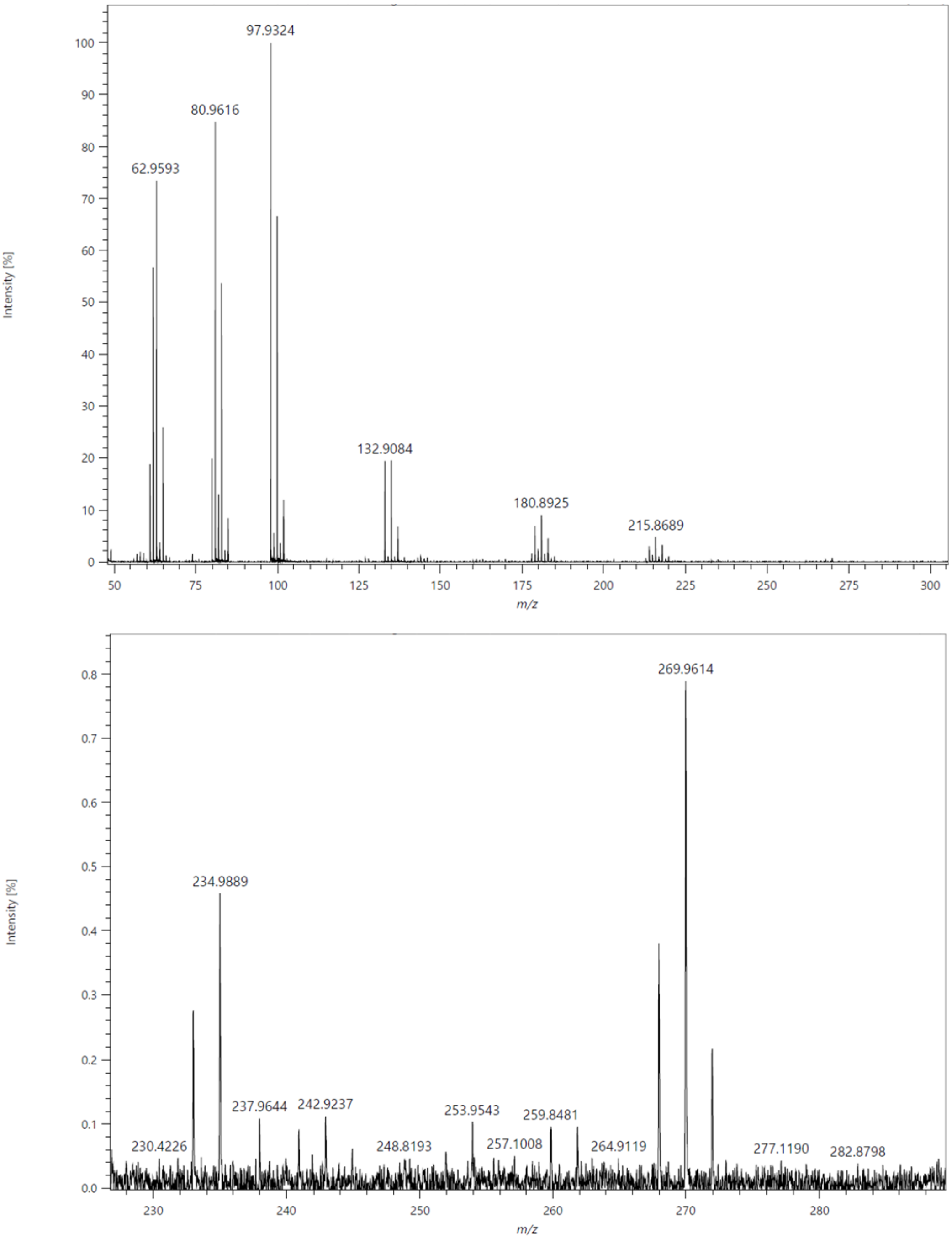

Figure S92: EI(+) mass spectrum of **3**·SMe_2. (top: full spectrum; bottom: section of the spectrum showing a signal at 242.9237 *m*/*z* corresponding to $[C_2H_6BCl_4SSi]^+$).

3.8. Mass spectrum of Py·$Cl_2B–SiCl_3$ (3·Py)

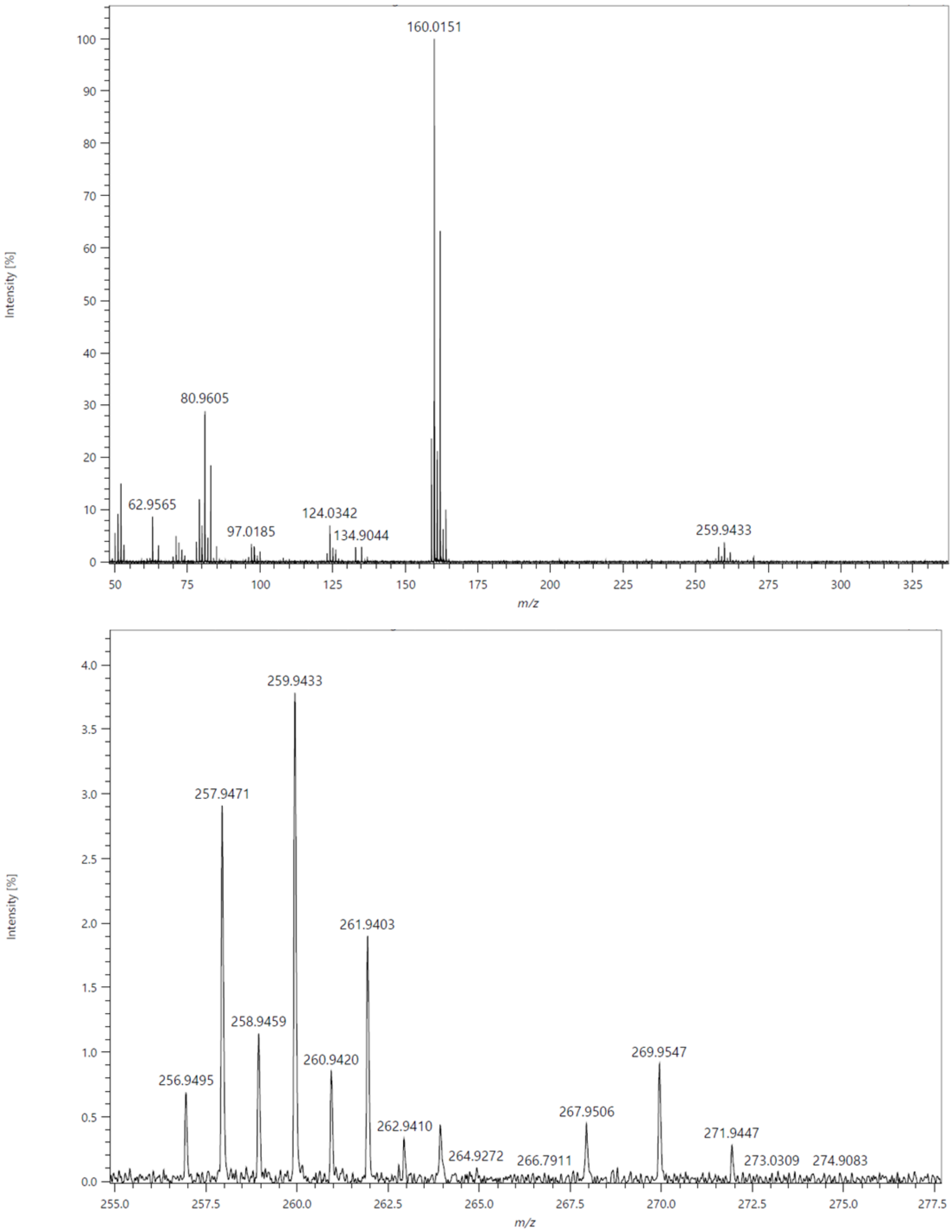

Figure S93: EI(+) mass spectrum of **3**·Py. (top: full spectrum; bottom: section of the spectrum showing a signal at 259.9433 *m*/*z* corresponding to $[C_5H_5BCl_4NSi]^+$).

3.9. Mass spectrum of $Ph_3P·Cl_2B–SiCl_3$ (3·PPh_3)

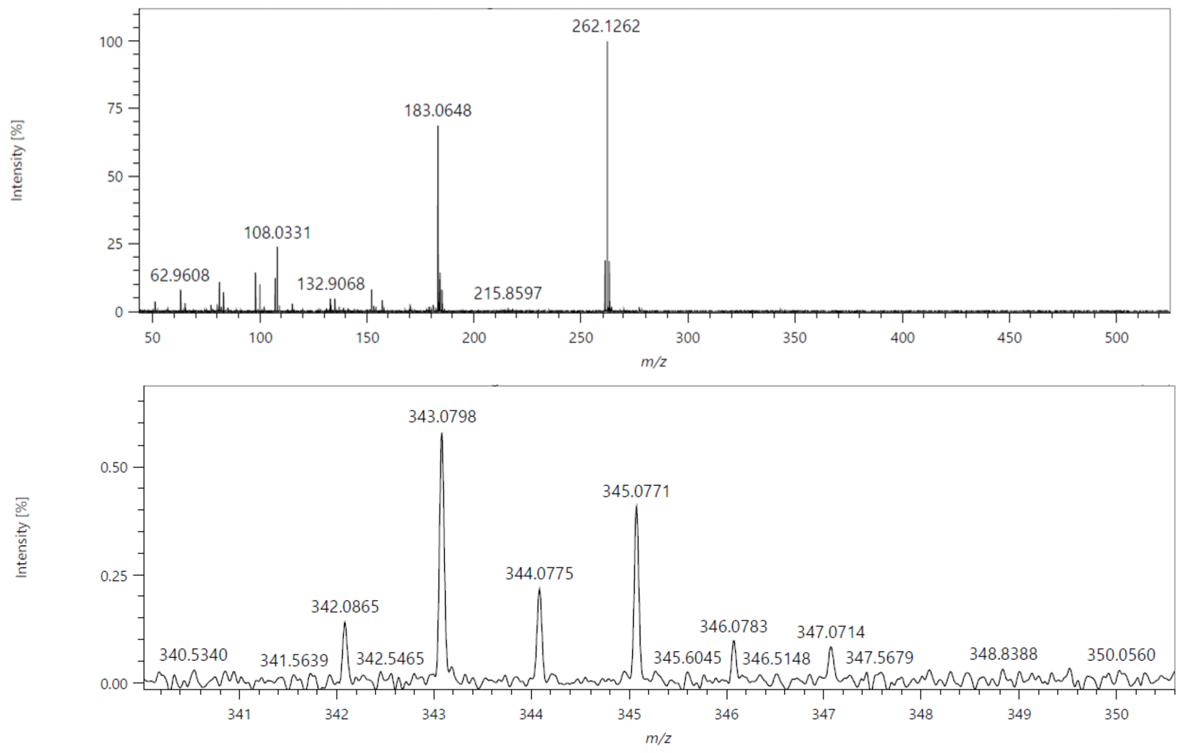

Figure S94: EI(+) mass spectrum of **3**·PPh_3. (top: full spectrum; bottom: section of the spectrum showing a signal at 343.0789 *m*/*z* corresponding to $[C_{18}H_{15}BCl_2P]^+$).

3.10. Mass spectrum of IDipp·Cl_2B–$SiCl_3$ (3·IDipp)

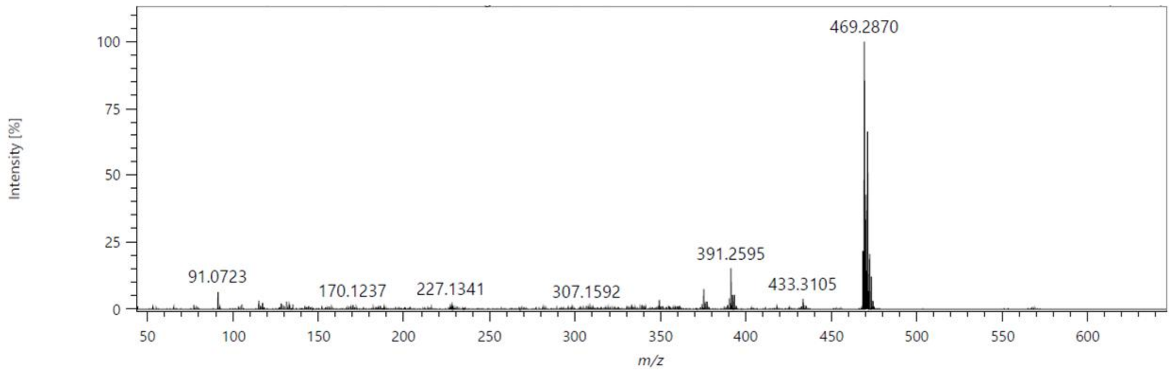

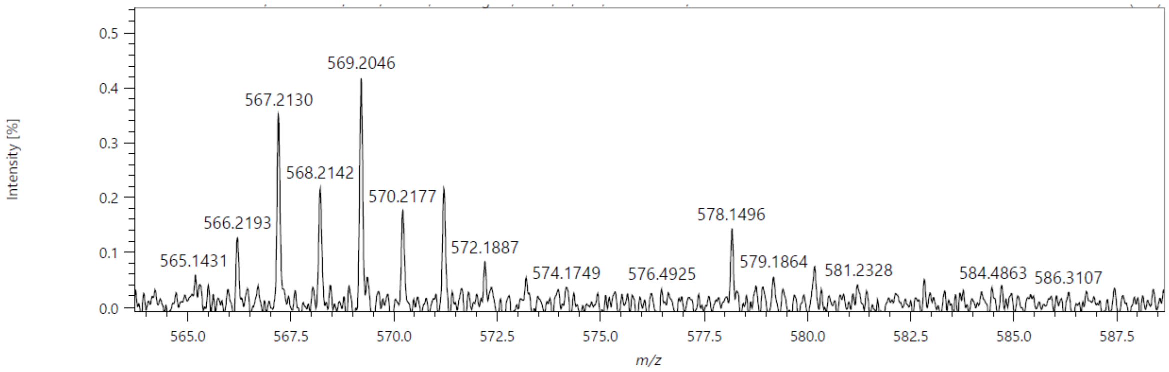

Figure S95: EI(+) mass spectrum of **3**·IDipp. (top: full spectrum; bottom: section of the spectrum showing a signal at 569.2046 *m/z* corresponding to $[C_{27}H_{36}BCl_4N_2Si]^+$).

3.11. Mass spectrum of BI_3·PPh_3

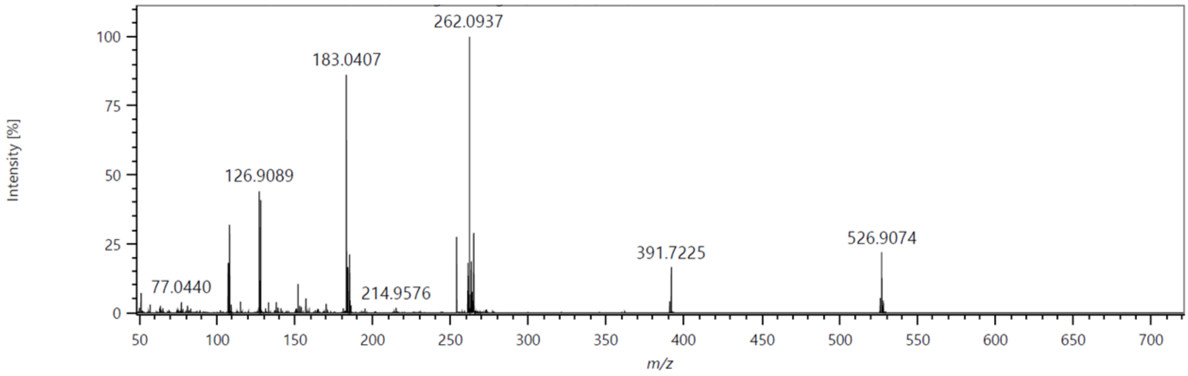

Figure S96: EI(+) mass spectrum of BI_3·PPh_3.

3.12. Mass spectrum of BI_3·IDipp

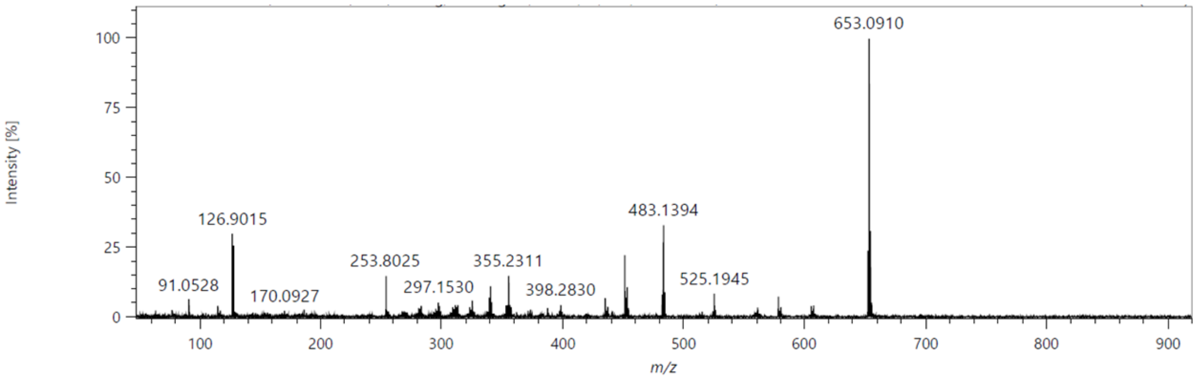

Figure S97: EI(+) mass spectrum of BI_3·IDipp.

3.13. Mass spectrum of Py·I_2B–C_2H_4–SiI_3 (5·Py)

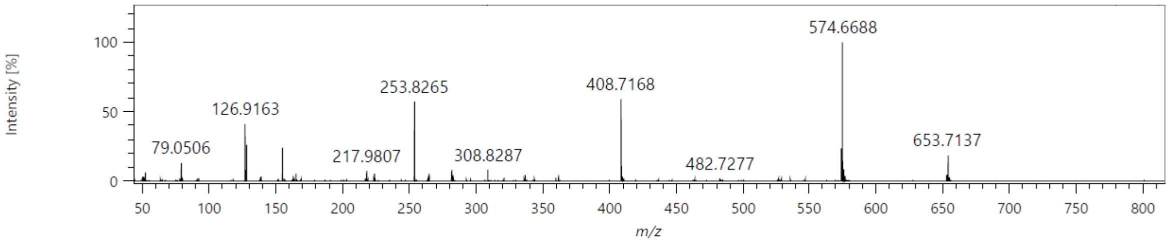

Figure S98: EI(+) mass spectrum of **5**·Py.

3.14. Mass spectrum of (I_2B–)(I_3Si–)C_6H_{10} (7)

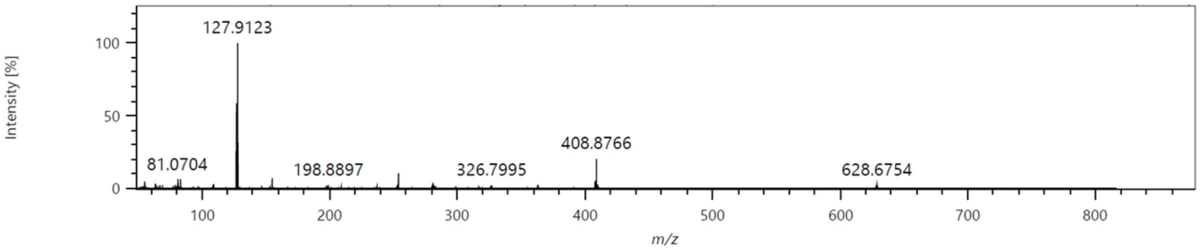

Figure S99: EI(+) mass spectrum of **7**.

3.15. Mass spectrum of Me_2S·I_2B–C_2H_4–I

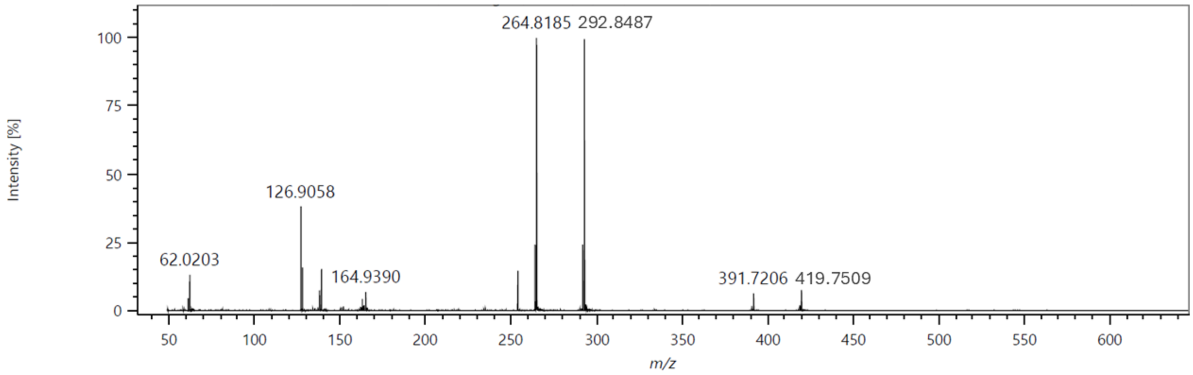

Figure S100: EI(+) mass spectrum of Me_2S·I_2B–C_2H_4–I.

3.16. Mass spectrum of 8

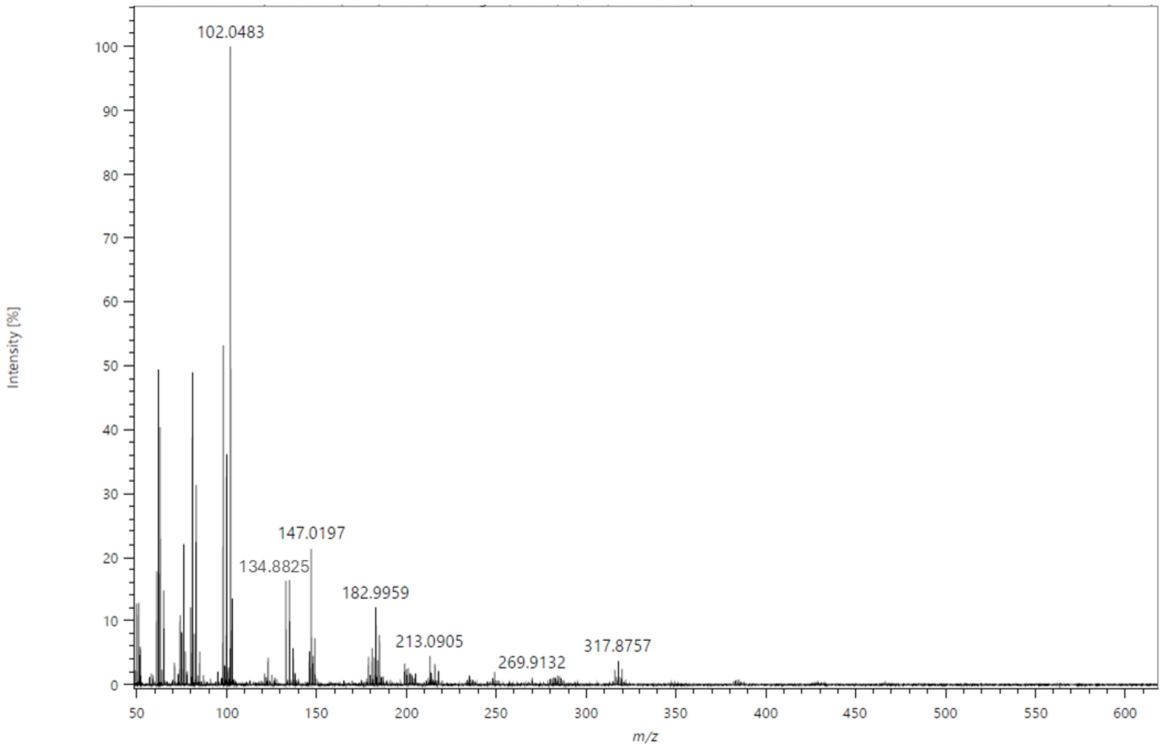

Figure S101: EI(+) mass spectrum of **8**.

4. Single-crystal X-ray structure analysis

General considerations

Single-crystal diffraction data were collected at –100 °C on a *STOE IPDS II* two-circle diffractometer equipped with a *Genix 3D HS* microfocus MoK_α X-ray source (λ = 0.71073 Å). The data finalization, including empirical absorption corrections, was done using the *CrysAlisPro* software v.1.171.42.43a (Rigaku Oxford Diffraction, 2022). The structures were solved using the SHELXT program and refined against $|F|^2$ with full-matrix least-squares techniques using the program *SHELXL*-2018/3.[S26–28] All H atoms were located geometrically and refined riding on the pivot atom.

In the disordered structure of $[Et_4N][Cl_3B–SiCl_3]$ (see chapter 4.7), the B and Si atoms share the same position, which could be refined with two split sites separated by 0.185 Å, yielding a structural model with reasonable geometry. The anisotropic displacement parameters of the B and Si atoms were equated using the EADP instruction to avoid correlations during structure refinement.

The crystal structures of **5**·SMe_2 and **8** (see chapters 4.14 and 4.18) were determined from twinned crystals and refined over all identified domains using the *SHELXL* 'HKLF 5' format data set generated by *CrysAlisPro*. For **5**·SMe_2, two domains were found by careful analysis of the reciprocal space using the *CrysAlisPro* software. Due to the non-centrosymmetric (polar) nature of the structure (space group *Pn*), racemic twinning cannot be excluded at this stage. To be able to take racemic twinning into consideration, we generated two more domains by applying a (–1 0 0 / 0 –1 0 / 0 0 –1) matrix using the *HKLF5tools* software.[S29] The relative weights of the 2nd, 3rd and 4th domains were refined as 0.089, 0.387, and 0.121, respectively. The twinned structure **8** is centrosymmetric, with a twin law (–1 0 0 / 0 –1 0 / 0 0 1). The relative weight of the minor domain proved to be 14.5% and did not allow reliable refinement over both components because of its small contribution. Therefore, we decided to exclude all non-overlapping reflections belonging to this domain from the refinement using the *HKLF5tools* software.[S29]

Both the crystals of $[Et_4N][(I_{2.03}/Cl_{0.97})B–SiI_3]$ (Cl/I ratio estimated after the refinement of the site occupancy factors with fixed isotropic parameters that were then fixed during the anisotropic refinement) and of **2**·Py are racemic twins (see chapters 4.1 and 4.3 respectively). In each case, there are only two domains, with the twin batches refined to 79:21(4)% and 64(7):36%, respectively.

Compounds **2**·SMe_2, **2**·PPh_3, and **7** are dimorphic, and all represent packing polymorphs. The α- and β-phases were denoted according to the density rule: the denser modification corresponds to the α-phase, while the less dense modification is denoted as the β-phase.

Deposition Numbers CCDC-2470869-2470889 contain the supplementary crystallographic data for this paper. These data can be obtained free of charge via the joint Cambridge Crystallographic Data Centre (CCDC) and Fachinformationszentrum Karlsruhe Access Structures service. Crystallographic data and parameters of the diffraction experiments are given in Tables S5-S15.

Table S5: Selected crystallographic data for $[Et_4N][(I_{2.03}/Cl_{0.97})B–SiI_3]$.

	$[Et_4N][(I_{2.03}/Cl_{0.97})B–SiI_3]$
CCDC-Code	CCDC-2470886
Chemical formula	$C_8H_{20}BCl_{0.97}I_{5.03}NSi$
M_r	841.61
Crystal system, space group	Orthorhombic, $P2_12_12_1$
Temperature (K)	173
a, *b*, *c* (Å)	12.2698(4), 12.9350(5), 13.0533(5)
α, β, γ (°)	90, 90, 90
V (Å^3)	2071.68(12)
Z	4
F(000)	1508
D_x (Mg m^{-3})	2.698
Radiation type	Mo *K*α
μ (mm^{-1})	7.72
Crystal shape	Elongated plate
Color	Colorless
Crystal size (mm)	0.39 × 0.12 × 0.07
Absorption correction	Multi-scan
T_{min}, T_{max}	0.031, 1.000
No. of measured, independent and observed [$I > 2\sigma(I)$] reflections	46719, 6351, 6080
R_{int}	0.079
Θ_{max} (°)	30.6
Range of *h*, *k*, *l*	$h = -17 \rightarrow 17$, $k = -18 \rightarrow 18$, $l = -18 \rightarrow 18$
$R[F^2 > 2\sigma(F^2)]$, $wR(F^2)$, S	0.031, 0.074, 1.05
No. of reflections	6351
No. of parameters	159
$\Delta\rho_{max}$, $\Delta\rho_{min}$ (e Å^{-3})	1.07, -1.25
Absolute structure parameter	0.21(4)

Computer programs: *X-AREA* (*Stoe & Cie*, 2001), *CrysAlis PRO* 1.171.42.43a (*Rigaku OD*, 2022), *SHELXS* (G. M. Sheldrick, 1997), *SHELXT* (G. M. Sheldrick, 2015), *SHELXL-2018*/3 (Sheldrick, 2018).

Table S6: Selected crystallographic data for **2**·SMe_2.

	**2**·SMe_2 (α-)	**2**·SMe_2 (β-)
CCDC-Code	CCDC-2470873	CCDC-2470874
Chemical formula	$C_2H_6BI_5SSi$	$C_2H_6BI_5SSi$
M_r	735.53	735.53
Crystal system, space group	Monoclinic, $P2_1/n$	Monoclinic, $P2_1/n$
Temperature (K)	173	173
a, *b*, *c* (Å)	8.43800(17), 26.5897(5), 12.7476(3)	8.41865(16), 13.8073(3), 12.3034(2)
α, β, γ (°)	90, 96.835(2), 90	90, 91.4990(18), 90
V (Å^3)	2839.77(10)	1429.64(4)
Z	8	4
F(000)	2544	1272
D_x (Mg m^{-3})	3.441	3.417
Radiation type	Mo *K*α	Mo *K*α
μ (mm^{-1})	11.14	11.06
Crystal shape	Plate	Plate
Color	Colorless	Colorless
Crystal size (mm)	0.19 × 0.06 × 0.03	0.38 × 0.17 × 0.09
Absorption correction	Multi-scan	Multi-scan
T_{min}, T_{max}	0.122, 1.000	0.058, 0.093
No. of measured, independent and observed [$I > 2\sigma(I)$] reflections	37793, 6774, 5943	22612, 3533, 3297
R_{int}	0.071	0.081
Θ_{max} (°)	27.9	28.3
Range of *h*, *k*, *l*	*h* = -11→10, *k* = -35→35, *l* = -16→16	*h* = -11→11, *k* = -18→18, *l* = -16→14
$R[F^2 > 2\sigma(F^2)]$, $wR(F^2)$, S	0.034, 0.094, 1.05	0.033, 0.085, 1.09
No. of reflections	6774	3533
No. of parameters	185	94
$\Delta\rho_{max}$, $\Delta\rho_{min}$ (e Å^{-3})	1.57, -2.20	1.82, -1.73

Computer programs: *X-AREA* (*Stoe & Cie*, 2001), *CrysAlis PRO* 1.171.42.43a (*Rigaku OD*, 2022), *SHELXS* (G. M. Sheldrick, 1997), *SHELXT* (G. M. Sheldrick, 2015), *SHELXL-2018*/3 (Sheldrick, 2018).

Table S7: Selected crystallographic data for **2**·Py and **2**·IDipp.

	**2**·Py	**2**·IDipp
CCDC-Code	CCDC-2470872	CCDC-2470869
Chemical formula	$C_5H_5BI_5NSi$	$C_{27}H_{36}BI_5N_2Si$
M_r	752.50	1061.98
Crystal system, space group	Orthorhombic, *Pna*2$_1$	Triclinic, *P*1
Temperature (K)	173	173
a, *b*, *c* (Å)	13.9353(3), 8.42736(19), 13.3513(2)	9.9369(3), 10.2194(2), 17.2209(4)
α, β, γ (°)	90, 90, 90	90.8586(18), 95.7957(19), 106.056(2)
V (Å^3)	1567.94(5)	1670.27(7)
Z	4	2
F(000)	1304	992
D_x (Mg m^{-3})	3.188	2.112
Radiation type	Mo *K*α	Mo *K*α
μ (mm^{-1})	9.96	4.71
Crystal shape	Prism	Plate
Color	Colorless	Colorless
Crystal size (mm)	0.24 × 0.23 × 0.15	0.26 × 0.23 × 0.13
Absorption correction	Multi-scan	Multi-scan
T_{min}, T_{max}	0.261, 1.000	0.490, 1.000
No. of measured, independent and observed [$I > 2\sigma(I)$] reflections	16700, 3849, 3718	26704, 7087, 6871
R_{int}	0.058	0.038
Θ_{max} (°)	28.3	26.7
Range of *h*, *k*, *l*	*h* = -18→16, *k* = -11→11, *l* = -17→17	*h* = -12→12, *k* = -12→12, *l* = -21→21
$R[F^2 > 2\sigma(F^2)]$, $wR(F^2)$, S	0.032, 0.078, 1.04	0.030, 0.084, 1.05
No. of reflections	3849	7087
No. of parameters	231	333
$\Delta\rho_{max}$, $\Delta\rho_{min}$ (e Å^{-3})	0.89, -0.95	1.35, -2.51
Absolute structure parameter	0.64(7)/0.36	–

Computer programs: *X-AREA* (*Stoe & Cie*, 2001), *CrysAlis PRO* 1.171.42.43a (*Rigaku OD*, 2022), *SHELXS* (G. M. Sheldrick, 1997), *SHELXT* (G. M. Sheldrick, 2015), *SHELXL-2018*/3 (Sheldrick, 2018).

Table S8: Selected crystallographic data for **2**·PPh_3.

	**2**·PPh_3 (α-)	**2**·PPh_3 (β-)
CCDC-Code	CCDC-2470870	CCDC-2470871
Chemical formula	$C_{18}H_{15}BI_5PSi$	$C_{18}H_{15}BI_5PSi$
M_r	935.67	935.67
Crystal system, space group	Triclinic, $P\bar{1}$	Monoclinic, $P2_1/c$
Temperature (K)	173	173
a, b, c (Å)	9.4185(4), 10.7141(5), 12.9896(4)	16.2424(2), 21.4873(3), 14.3571(2)
α, β, γ (°)	75.377(3), 78.132(3), 84.148(3)	90, 96.933(1), 90
V (Å^3)	1239.49(9)	4974.07(12)
Z	2	8
F(000)	844	3376
D_x (Mg m^{-3})	2.507	2.499
Radiation type	Mo Kα	Mo Kα
μ (mm^{-1})	6.39	6.34
Crystal shape	Prism	Plate
Color	Colorless	Colorless
Crystal size (mm)	0.16 × 0.13 × 0.06	0.10 × 0.07 × 0.05
Absorption correction	Multi-scan	Multi-scan
T_{min}, T_{max}	0.702, 1.000	0.041, 0.063
No. of measured, independent and observed [$I > 2σ(I)$] reflections	21203, 7151, 6456	60760, 10970, 7706
R_{int}	0.036	0.049
$Θ_{max}$ (°)	30.0	27.1
Range of h, k, l	h = -13→13, k = -15→15, l = -18→18	h = -20→20, k = -27→27, l = -18→18
$R[F^2 > 2σ(F^2)]$, $wR(F^2)$, S	0.036, 0.094, 1.04	0.025, 0.052, 0.80
No. of reflections	7151	10970
No. of parameters	235	469
$Δρ_{max}$, $Δρ_{min}$ (e Å^{-3})	2.22, -2.74	1.35, -0.69

Computer programs: *X-AREA* (*Stoe & Cie*, 2001), *CrysAlis PRO* 1.171.42.43a (*Rigaku OD*, 2022), *SHELXS* (G. M. Sheldrick, 1997), *SHELXT* (G. M. Sheldrick, 2015), *SHELXL-2018*/3 (Sheldrick, 2018).

Table S9: Selected crystallographic data for **4** and $[Et_4N][Cl_3B–SiCl_3]$.

	**4**	$[Et_4N][Cl_3B–SiCl_3]$
CCDC-Code	CCDC-2470879	CCDC-2470885
Chemical formula	$B_2I_{12}Si_3$	$C_8H_{20}BCl_5NSi$
M_r	1628.69	381.85
Crystal system, space group	Monoclinic, $P2_1$	Orthorhombic, *Pbcn*
Temperature (K)	173	173
a, *b*, *c* (Å)	7.6585(4), 14.3673(6), 12.0598(7)	11.4943(4), 12.1239(4), 12.3124(5)
α, β, γ (°)	90, 107.507(6), 90	90, 90, 90
V (Å^3)	1265.50(12)	1715.81(11)
Z	2	4
F(000)	1376	784
D_x (Mg m^{-3})	4.274	1.478
Radiation type	Mo *K*α	Mo *K*α
μ (mm^{-1})	14.81	1.05
Crystal shape	Plate	Plate
Color	Colorless	Colorless
Crystal size (mm)	0.08 × 0.08 × 0.04	0.08 × 0.07 × 0.04
Absorption correction	Multi-scan	Multi-scan
T_{min}, T_{max}	0.416, 1.000	0.792, 1.000
No. of measured, independent and observed [$I > 2\sigma(I)$] reflections	17036, 5575, 4768	20540, 1898, 1598
R_{int}	0.050	0.072
Θ_{max} (°)	27.1	27.1
Range of *h*, *k*, *l*	*h* = -9→9, *k* = -18→18, *l* = -15→15	*h* = -13→14, *k* = -15→15, *l* = -15→15
$R[F^2 > 2\sigma(F^2)]$, $wR(F^2)$, S	0.038, 0.080, 1.02	0.029, 0.074, 1.06
No. of reflections	5575	1898
No. of parameters	166	83
$\Delta\rho_{max}$, $\Delta\rho_{min}$ (e Å^{-3})	1.24, -1.55	0.44, -0.17
Absolute structure parameter	0.04(5)	–

Computer programs: *X-AREA* (*Stoe & Cie*, 2001), *CrysAlis PRO* 1.171.42.43a (*Rigaku OD*, 2022), *SHELXS* (G. M. Sheldrick, 1997), *SHELXT* (G. M. Sheldrick, 2015), *SHELXL-2018*/3 (Sheldrick, 2018).

Table S10: Selected crystallogaphic data for **3**·SMe_2 and **3**·Py.

	**3**·SMe_2	**3**·Py
CCDC-Code	CCDC-2470878	CCDC-2470877
Chemical formula	$C_2H_6BCl_5SSi$	$C_5H_5BCl_5NSi$
M_r	278.28	295.25
Crystal system, space group	Monoclinic, $P2_1/n$	Monoclinic, $P2_1/c$
Temperature (K)	173	173
a, *b*, *c* (Å)	7.1191(3), 16.0988(7), 9.7847(3)	8.53823(16), 9.89175(18), 13.7811(3)
α, β, γ (°)	90, 95.647(3), 90	90, 97.3526(19), 90
V (Å^3)	1115.98(7)	1154.35(4)
Z	4	4
F(000)	552	584
D_x (Mg m^{-3})	1.656	1.699
Radiation type	Mo *K*α	Mo *K*α
μ (mm^{-1})	1.53	1.31
Crystal shape	Plate	Plate
Color	Colorless	Colorless
Crystal size (mm)	0.10 × 0.07 × 0.05	0.36 × 0.30 × 0.06
Absorption correction	Multi-scan	Multi-scan
T_{min}, T_{max}	0.960, 1.000	0.591, 1.000
No. of measured, independent and observed [$I > 2\sigma(I)$] reflections	13109, 2361, 2053	18205, 3532, 3290
R_{int}	0.051	0.040
Θ_{max} (°)	26.8	30.6
Range of *h*, *k*, *l*	*h* = -9→9, *k* = -20→20, *l* = -12→12	*h* = -12→12, *k* = -13→14, *l* = -19→16
$R[F^2 > 2\sigma(F^2)]$, $wR(F^2)$, S	0.032, 0.082, 1.05	0.029, 0.077, 1.05
No. of reflections	2361	3532
No. of parameters	93	118
$\Delta\rho_{max}$, $\Delta\rho_{min}$ (e Å^{-3})	0.55, -0.43	0.51, -0.44

Computer programs: *X-AREA* (*Stoe & Cie*, 2001), *CrysAlis PRO* 1.171.42.43a (*Rigaku OD*, 2022), *SHELXS* (G. M. Sheldrick, 1997), *SHELXT* (G. M. Sheldrick, 2015), *SHELXL-2018*/3 (Sheldrick, 2018).

Table S11: Selected crystallographic data for **3**·PPh_3 and **3**·IDipp.

	**3**·PPh_3	**3**·IDipp
CCDC-Code	CCDC-2470876	CCDC-2470875
Chemical formula	$C_{18}H_{15}BCl_5PSi$	$C_{27}H_{36}BCl_5N_2Si$
M_r	478.42	604.73
Crystal system, space group	Triclinic, $P\bar{1}$	Trigonal, $P3_2$
Temperature (K)	173	173
a, *b*, *c* (Å)	10.3063(7), 13.1717(10), 17.0060(13)	17.5229(2), 17.5229(2), 26.2872(5)
α, β, γ (°)	102.565(6), 106.946(6), 96.664(6)	90, 90, 120
V (Å^3)	2115.3(3)	6990.2(2)
Z	4	9
F(000)	968	2844
D_x (Mg m^{-3})	1.502	1.293
Radiation type	Mo *K*α	Mo *K*α
μ (mm^{-1})	0.82	0.53
Crystal shape	Needle	Prism
Color	Colorless	Colorless
Crystal size (mm)	0.19 × 0.04 × 0.03	0.20 × 0.14 × 0.11
Absorption correction	Multi-scan	Multi-scan
T_{min}, T_{max}	0.981, 1.000	0.720, 1.000
No. of measured, independent and observed [$I > 2\sigma(I)$] reflections	20045, 9911, 7434	81147, 18347, 16709
R_{int}	0.055	0.076
Θ_{max} (°)	27.90	26.0
Range of *h*, *k*, *l*	*h* = -13→13, *k* = -17→17, *l* = -22→22	*h* = -21→21, *k* = -21→21, *l* = -32→32
$R[F^2 > 2\sigma(F^2)]$, $wR(F^2)$, S	0.047, 0.120, 1.02	0.036, 0.084, 1.03
No. of reflections	9911	18347
No. of parameters	469	997
$\Delta\rho_{max}$, $\Delta\rho_{min}$ (e Å^{-3})	0.48, -0.44	0.30, -0.19
Absolute structure parameter	–	-0.05(2)

Computer programs: *X-AREA* (*Stoe & Cie*, 2001), *CrysAlis PRO* 1.171.42.43a (*Rigaku OD*, 2022), *SHELXS* (G. M. Sheldrick, 1997), *SHELXT* (G. M. Sheldrick, 2015), *SHELXL-2018*/3 (Sheldrick, 2018).

Table S12: Selected crystallographic data for **5**·SMe_2 and **5**·Py.

	**5**·SMe_2	**5**·Py
CCDC-Code	CCDC-2470880	CCDC-2470881
Chemical formula	$C_4H_{10}BI_5SSi$	$C_7H_9BI_5NSi$
M_r	763.58	780.55
Crystal system, space group	Monoclinic, *Pn*	Monoclinic, $P2_1/n$
Temperature (K)	173	173
a, *b*, *c* (Å)	7.2014(4), 10.4366(5), 10.9255(5)	6.88451(12),10.11785(18), 24.8575(4)
α, β, γ (°)	90, 91.266(5), 90	90, 96.7665(17), 90
V ($Å^3$)	820.95(7)	1719.43(5)
Z	2	4
F(000)	668	1368
D_x (Mg m^{-3})	3.089	3.015
Radiation type	Mo *K*α	Mo *K*α
μ (mm^{-1})	9.64	9.09
Crystal shape	Plank	Needle
Color	Colorless	Colorless
Crystal size (mm)	0.41 × 0.08 × 0.03	0.35 × 0.05 × 0.03
Absorption correction	Multi-scan	Multi-scan
T_{min}, T_{max}	0.284, 1.000	0.474, 1.000
No. of measured, independent and observed [$I > 2\sigma(I)$] reflections	5249, 5249, 4305	30907, 4630, 4328
R_{int}	0.074	0.045
Θ_{max} (°)	28.3	29.1
Range of *h*, *k*, *l*	*h* = -9→9, *k* = -13→13, *l* = -14→14	*h* = -9→8, *k* = -13→13, *l* = -34→34
$R[F^2 > 2\sigma(F^2)]$, $wR(F^2)$, *S*	0.043, 0.133, 1.06	0.023, 0.059, 1.12
No. of reflections	5249	4628
No. of parameters	114	136
$\Delta\rho_{max}$, $\Delta\rho_{min}$ (e $Å^{-3}$)	1.78, -1.21	1.34, -0.82
Absolute structure parameter	0.03(12)	–

Computer programs: *X-AREA* (*Stoe & Cie*, 2001), *CrysAlis PRO* 1.171.42.43a (*Rigaku OD*, 2022), *SHELXS* (G. M. Sheldrick, 1997), *SHELXT* (G. M. Sheldrick, 2015), *SHELXL-2018*/3 (Sheldrick, 2018).

Table S13: Selected crystallographic data for $BI_3{\cdot}PPh_3$ and $BI_3{\cdot}$IDipp.

	$BI_3{\cdot}PPh_3$	$BI_3{\cdot}$IDipp
CCDC-Code	CCDC-2470888	CCDC-2470887
Chemical formula	$C_{18}H_{15}BI_3P$	$C_{27}H_{36}BI_3N_2$
M_r	653.78	780.09
Crystal system, space group	Trigonal, *R*3	Monoclinic, $P2_1/c$
Temperature (K)	173	173
a, *b*, *c* (Å)	14.68163(19), 14.68163(19), 15.7976(3)	16.8190(9), 12.8372(5), 16.3668(7)
α, β, γ (°)	90, 90, 120	90, 118.691(6), 90
V (Å^3)	2948.97(10)	3099.9(3)
Z	6	4
F(000)	1812	1504
D_x (Mg m^{-3})	2.209	1.671
Radiation type	Mo *K*α	Mo *K*α
μ (mm^{-1})	4.85	3.04
Crystal shape	Plate	Block
Color	Colorless	Colorless
Crystal size (mm)	0.15 × 0.12 × 0.05	0.10 × 0.07 × 0.03
Absorption correction	Multi-scan	Multi-scan
T_{min}, T_{max}	0.393, 1.000	0.864, 1.000
No. of measured, independent and observed [$I > 2\sigma(I)$] reflections	17002, 2003, 1905	37375, 6597, 5983
R_{int}	0.043	0.059
Θ_{max} (°)	30.5	26.8
Range of *h*, *k*, *l*	*h* = -20→20, *k* = -20→20, *l* = -22→22	*h* = -21→21, *k* = -15→16, *l* = -20→20
$R[F^2 > 2\sigma(F^2)]$, $wR(F^2)$, *S*	0.017, 0.044, 1.09	0.039, 0.092, 1.18
No. of reflections	2003	6597
No. of parameters	70	306
$\Delta\rho_{max}$, $\Delta\rho_{min}$ (e Å^{-3})	0.68, -0.48	1.22, -1.60

Computer programs: *X-AREA* (*Stoe & Cie*, 2001), *CrysAlis PRO* 1.171.42.43a (*Rigaku OD*, 2022), *SHELXS* (G. M. Sheldrick, 1997), *SHELXT* (G. M. Sheldrick, 2015), *SHELXL-2018*/3 (Sheldrick, 2018).

Table S14: Selected crystallographic data for **7**.

	**7** (α-)	**7** (β-)
CCDC-Code	CCDC-2470882	CCDC-2470883
Chemical formula	$C_6H_{10}BI_5Si$	$C_6H_{10}BI_5Si$
M_r	755.54	755.54
Crystal system, space group	Orthorhombic, *Pbca*	Orthorhombic, $Pca2_1$
Temperature (K)	173	173
a, *b*, *c* (Å)	8.79053(14), 14.6868(2), 24.6134(4)	15.8306 (4), 7.30808 (16), 13.7822 (4)
α, β, γ (°)	90, 90, 90	90, 90, 90
V ($Å^3$)	3177.72(8)	1594.48 (7)
Z	8	4
F(000)	2640	1320
D_x (Mg m^{-3})	3.159	3.147
Radiation type	Mo *K*α	Mo *K*α
μ (mm^{-1})	9.83	9.79
Crystal shape	Block	Prism
Color	Colorless	Colorless
Crystal size (mm)	0.23 × 0.22 × 0.15	0.19 × 0.07 × 0.06
Absorption correction	Multi-scan	multi-scan
T_{min}, T_{max}	0.368, 1.000	0.699, 1.000
No. of measured, independent and observed [$I > 2\sigma(I)$] reflections	56285, 3250, 3058	15001, 3812, 3694
R_{int}	0.074	0.051
Θ_{max} (°)	26.4	0.658
Range of *h*, *k*, *l*	*h* = -10→10, *k* = -18→18, *l* = -30→30	*h* = -20→17, *k* = -9→9, *l* = -18→18
$R[F^2 > 2\sigma(F^2)]$, $wR(F^2)$, *S*	0.025, 0.062, 1.10	0.025, 0.057, 1.07
No. of reflections	3250	3812
No. of parameters	119	119
$\Delta\rho_{max}$, $\Delta\rho_{min}$ (e $Å^{-3}$)	1.85, -1.46	0.90, -0.46
Absolute structure parameter	–	0.43(5)

Computer programs: *X-AREA* (*Stoe & Cie*, 2001), *CrysAlis PRO* 1.171.42.43a (*Rigaku OD*, 2022), *SHELXS* (G. M. Sheldrick, 1997), *SHELXT* (G. M. Sheldrick, 2015), *SHELXL-2018*/3 (Sheldrick, 2018).

Table S15: Selected crystallographic data for $Me_2S{\cdot}I_2B–C_2H_4–I$ and **8**.

	$Me_2S{\cdot}I_2B–C_2H_4–I$	**8**
CCDC-Code	CCDC-2470889	CCDC-2470884
Chemical formula	$C_4H_{10}BI_3S$	$C_{10}H_{12}BCl_5SSi$
M_r	481.69	380.41
Crystal system, space group	Triclinic, $P\bar{1}$	Monoclinic, $P2_1/c$
Temperature (K)	173	173
a, *b*, *c* (Å)	7.2744(4), 8.7817(4), 8.9556(5)	12.8770(9), 9.8069(6), 13.5196(10)
α, β, γ (°)	85.843(4), 85.310(4), 85.904(4)	90, 102.896(7), 90
V (Å^3)	567.45(5)	1664.2(2)
Z	2	4
F(000)	428	768
D_x (Mg m^{-3})	2.819	1.518
Radiation type	Mo *K*α	Mo *K*α
μ (mm^{-1})	3.04	1.05
Crystal shape	Triangular prism	Block
Color	Colorless	Colorless
Crystal size (mm)	0.20 × 0.18 × 0.06	0.11 × 0.08 × 0.06
Absorption correction	Multi-scan	Multi-scan
T_{min}, T_{max}	0.451, 1.000	0.775, 1.000
No. of measured, independent and observed [$I > 2\sigma(I)$] reflections	14495, 3472, 3351	3701, 3701, 2103
R_{int}	0.041	0.102
Θ_{max} (°)	30.5	25.7
Range of *h*, *k*, *l*	*h* = -10→10, *k* = -12→12, *l* = -12→12	*h* = -15→15, *k* = -11→11, *l* = -16→16
$R[F^2 > 2\sigma(F^2)]$, $wR(F^2)$, S	0.024, 0.064, 1.09	0.037, 0.068, 0.72
No. of reflections	3472	3701
No. of parameters	85	190
$\Delta\rho_{max}$, $\Delta\rho_{min}$ (e Å^{-3})	1.66, -1.10	0.31, -0.23

Computer programs: *X-AREA* (*Stoe & Cie*, 2001), *CrysAlis PRO* 1.171.42.43a (*Rigaku OD*, 2022), *SHELXS* (G. M. Sheldrick, 1997), *SHELXT* (G. M. Sheldrick, 2015), *SHELXL-2018*/3 (Sheldrick, 2018).

4.1. Crystal Structure of $[Et_4N][(I_{2.03}/Cl_{0.97})B–SiI_3]$

The ionic compound $[Et_4N][(I_{2.03}/Cl_{0.97})B–SiI_3]$ crystallizes as a solvent-free phase in the chiral orthorhombic space group $P2_12_12_1$ (No. 19). It is isostructural to the known periodinated silylborane $[Et_4N]$[**1**].[S10] The crystal is a racemic twin with relative weights of the twin domains of 79:21%. Both cation and anion occupy general crystallographic positions (Figure S102 a). All three terminal halogen positions at the B atoms are shared between I and Cl in variable Cl/I ratios of 0.5412/0.4588, 0.1563/0.8437, and 0.275/0.725 (Figure S102 b). Therefore, single crystals of the compound are a solid solution of chlorinated and iodinated species, resulting in an averaged composition of $[Et_4N][(I_{2.03}/Cl_{0.97})B–SiI_3]$.

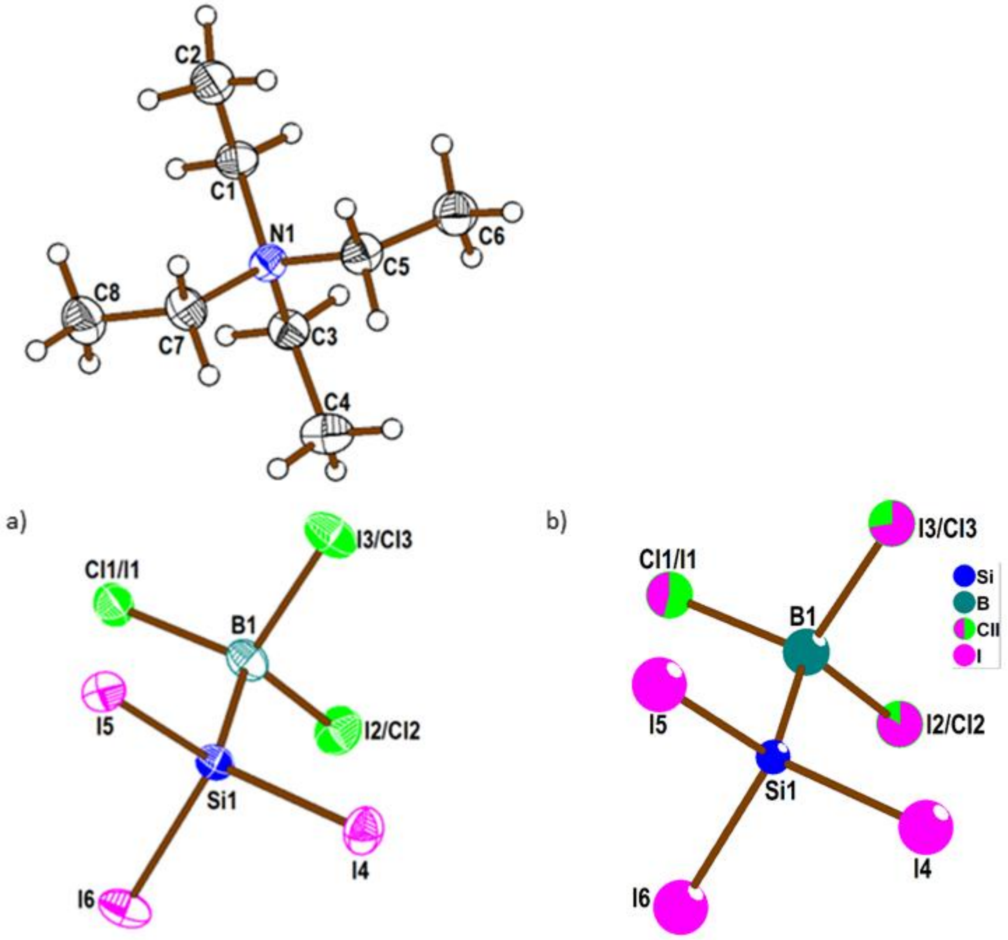

Figure S102: (a) Molecular structure of $[Et_4N][(I_{2.03}/Cl_{0.97})B–SiI_3]$ in the solid state. Atomic displacement ellipsoids are drawn at the 50% probability level. (b) the disorder of the terminal halogen atoms depicted as two-color sectors.

4.2. Crystal Structure of dimorphic $Me_2S \cdot I_2B–SiI_3$ (**2**·SMe_2)

Compound **2**·SMe_2 crystallizes solvent-free in two true polymorphic modifications. According to the density rule, the denser modification is denoted as α-, while the less dense modification is denoted as β-. Both polymorphs crystallize in the monoclinic space group $P2_1/n$ (No. 14) with two and one crystallographically unique molecules in general position(s), respectively (Figure S103 a, b). The difference between the polymorphs can be considered as the formal structure-superstructure relation with $b_\alpha \approx ½\, b_\beta$ and a decrease in the monoclinic angle by 4° when going from the α- to the β-phase. The molecules in both modifications, as well as two unique molecules in the α-polymorph, are geometrically very close to each other (Figure S103 c).

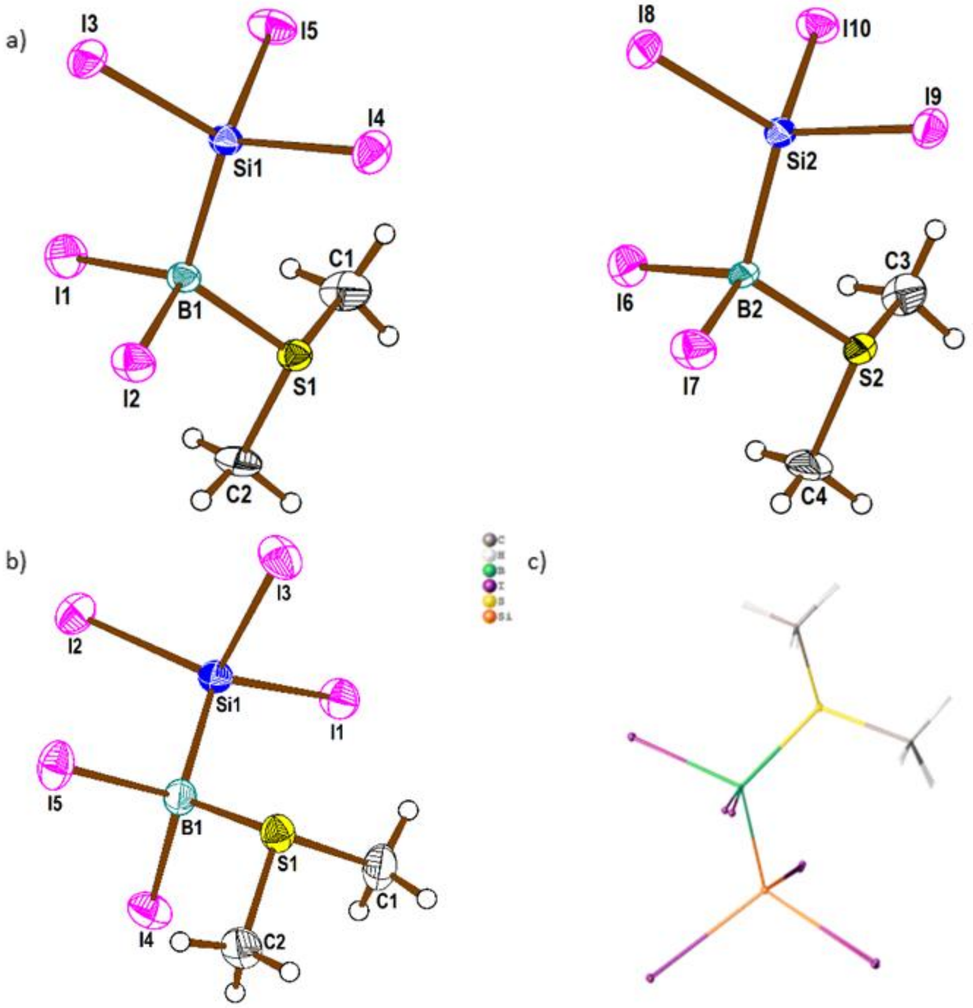

Figure S103: Molecular structures of (a) two crystallographically unique molecules **2**·SMe_2 in the α-form and (b) one unique molecule in the β-form in the solid state. Atomic displacement ellipsoids are drawn at the 50% probability level. (c) Overlay of the crystallographically unique molecules in both polymorphic modifications with the best atom-to-atom fit.

4.3. Crystal Structure of Py·I_2B–SiI_3 (2·Py)

Compound **2**·Py crystallizes as a solvent-free phase in the polar orthorhombic space group *Pna*2_1 (No. 33). The crystal is a racemic twin with the relative weights of the domains of 64:36%. Moreover, the structure is fully disordered with two crystallographically unique, geometrically almost identical, molecules overlapping with the relative weights of 0.594(7)/0.406 (Figure S104).

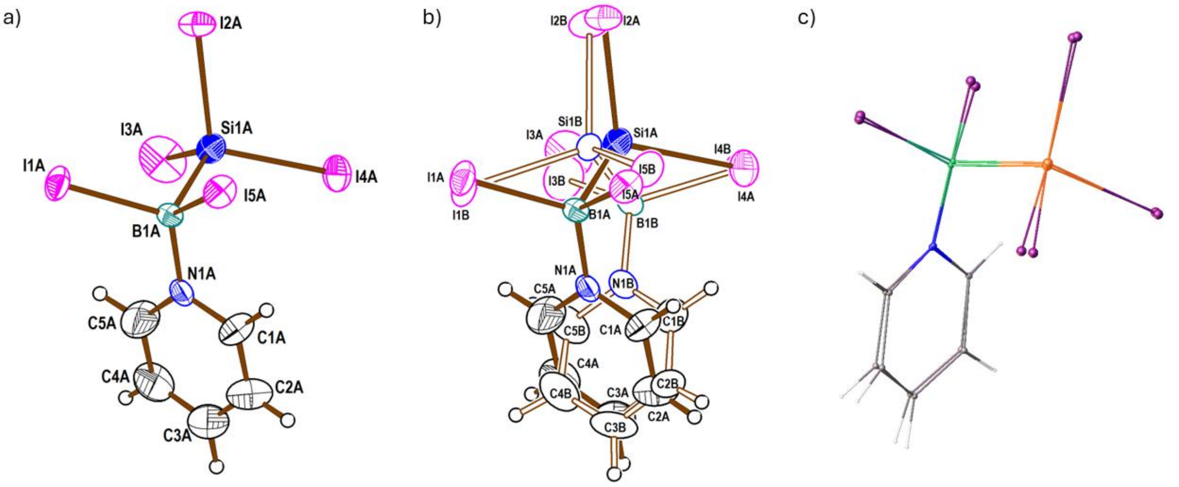

Figure S104: (a) Molecular structure of **2**·Py in the solid state. Atomic displacement ellipsoids are drawn at the 50% probability level. (b) Corresponding disorder model. Atoms labelled by endings 'A' and 'B' correspond to the major (64%) and minor (36%) disorder components, respectively. The minor disorder component is shown by empty bonds and ellipses. (c) Overlay of the two disordered molecules with the best atom-to-atom fit.

4.4. Crystal Structure of dimorphic $Ph_3P{\cdot}I_2B–SiI_3$ ($2{\cdot}PPh_3$)

Compound **2**·PPh_3 has two solvent-free true polymorphic modifications. According to the density rule, the denser triclinic modification (space group *P*1̄, No. 2) is denoted as α-, while the less dense modification (space group $P2_1/c$, No. 14) is denoted as β-. No obvious unit cell transformation ($V_\alpha \approx ¼ V_\beta$) between polymorphs suggests itself (Table S8). In both cases crystallographically unique molecules (one in α- and two in β-form) occupy general positions (Figure S105 a, b). The geometrical characteristics of the molecule in both polymorphous modifications are similar and differ mostly in the slightly different rotation degree of the phenyl rings (Figure S105 c).

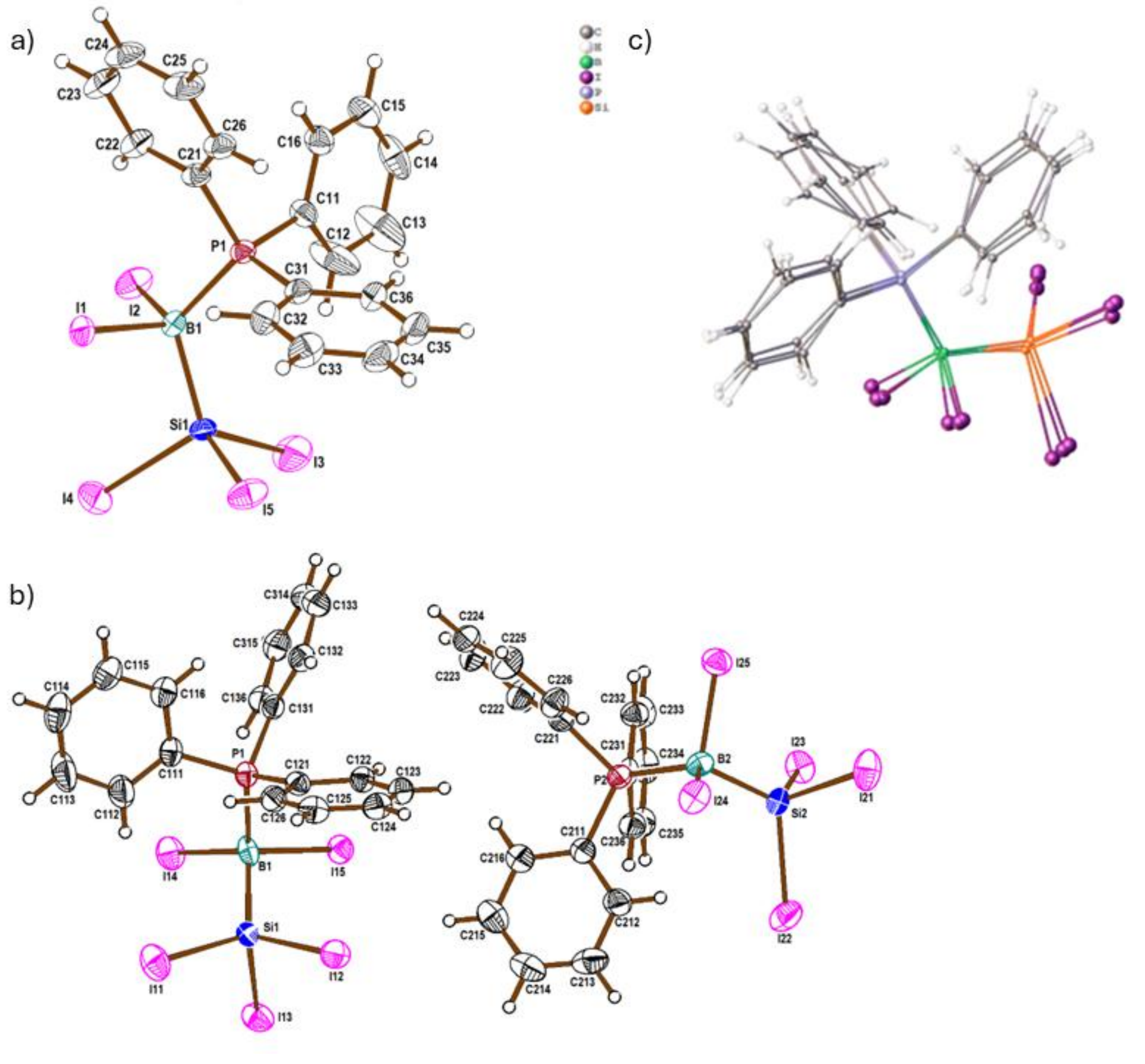

Figure S105: Molecular structure of **2**·PPh_3 (a) in the α-form and (b) in the β-form in the solid state. Atomic displacement ellipsoids are drawn at the 50% probability level. (c) Overlay of all crystallographically unique molecules in both polymorphic modifications with the best atom-to-atom fit.

4.5. Crystal Structure of IDipp·$I_2B–SiI_3$ (2·IDipp)

Compound **2·**IDipp crystallizes as a solvent-free phase in the triclinic space group *P*1 (No. 2) with one crystallographically unique molecule in a general position (Figure S106).

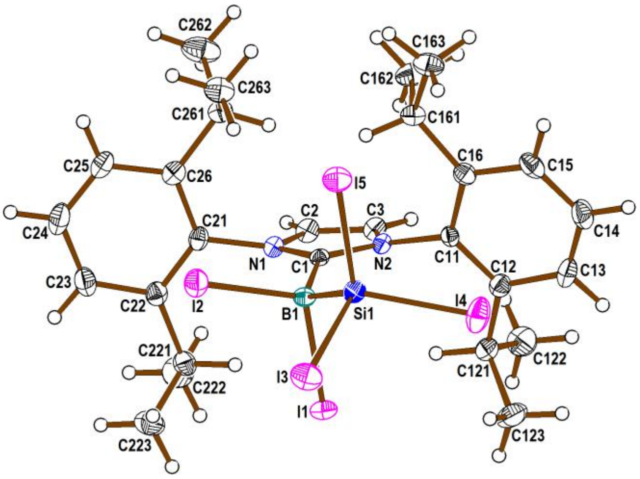

Figure S106: Molecular structure of **2·**IDipp in the solid state. Atomic displacement ellipsoids are drawn at the 50% probability level.

4.6. Crystal Structure of $(I_2B–SiI_3)_2·SiI_2$ (4)

Compound **4** crystallizes as a solvent-free phase in the chiral polar space group $P2_1$ (No. 4) within the monoclinic crystal system, with one crystallographically unique molecule in a general position (Figure S107). The entire $BSiI_3$ fragment is disordered over two positions, with relative weights of 73:27% (Figure S107). Due to the minimal contribution of the boron atoms to the reflection intensities, their anisotropic displacement parameters were constrained to be equal and restrained using the *EADP* and *ISOR* instructions, respectively. The crystallographic disorder is accompanied by an 'inversion' of the configuration of the boron atom B2 relative to the entire structure, resulting in the overlap of two diastereomers at the same crystallographic position. Furthermore, refinement of the absolute structure (Flack) parameter confirms that the entire crystal is homochiral, meaning that only these two diastereomers are present in crystal of **4** selected for the diffraction study.

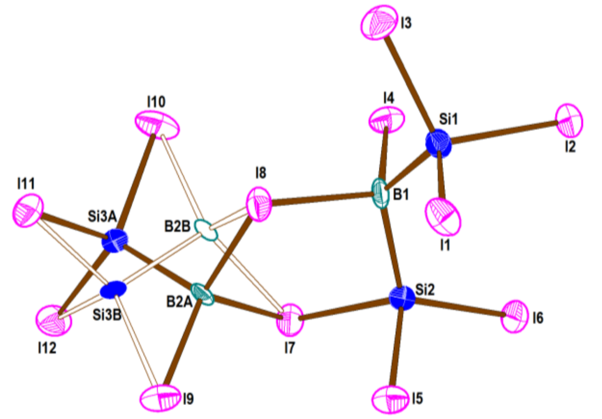

Figure S107: Molecular structure of **4** in the solid state. Atomic displacement ellipsoids are drawn at the 50% probability level. The minor position of the $BSiI_3$ fragment is shown with empty ellipses and bonds.

4.7. Crystal Structure of $[Et_4N][Cl_3B–SiCl_3]$

The ionic solvent-free compound $[Et_4N][Cl_3B–SiCl_3]$ crystallizes in the orthorhombic space group *Pbcn* (No. 60) with both the $[Et_4N]^+$ cation and the $[Cl_3BSiCl_3]^-$ anion laying at the 2-fold axis parallel to the *b* axis (Figure S108). At that, the anion is disordered over two symmetrically equivalent positions over the 2-fold axis passing through the middle of the B–Si bond. As a result, the crystallographic positions of the B and Si atoms nearly coincide, while the corresponding Cl atoms do not exhibit splitting. Therefore, the crystallographically unique part of the anion consists of one 'halve' of the structure, one B/Si site with 50:50% occupancy and three Cl positions (Figure S108).

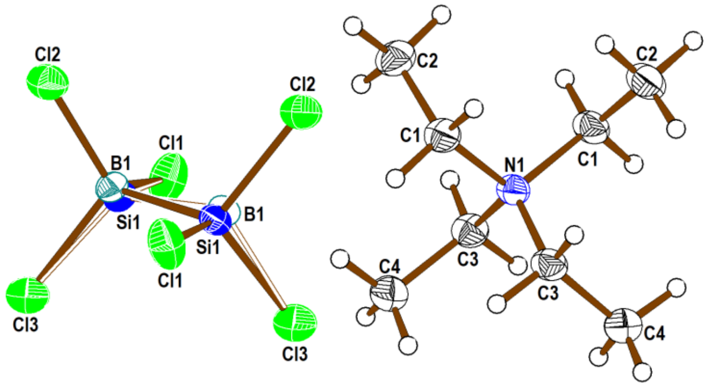

Figure S108: Molecular structure of $[Et_4N][Cl_3B–SiCl_3]$ in the solid state. Atomic displacement ellipsoids are drawn at the 50% probability level. Disorder pattern for the anion is shown by empty bonds and ellipses.

4.8. Crystal Structure of $Me_2S{\cdot}Cl_2B{-}SiCl_3$ (3·SMe₂)

Compound **3**·SMe_2 crystallizes as a solvent-free phase in the monoclinic space group $P2_1$/n (No. 14) with one crystallographically unique molecule in a general position (Figure S109).

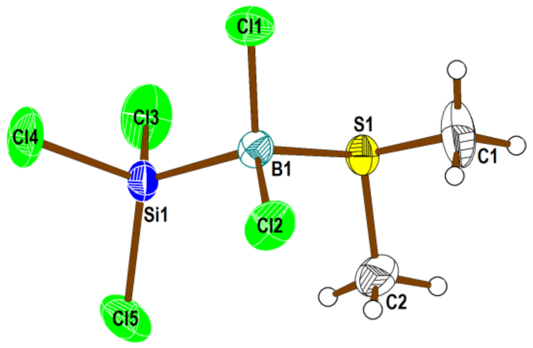

Figure S109: Molecular structure of **3**·SMe_2 in the solid state. Atomic displacement ellipsoids are drawn at the 50% probability level.

4.9. Crystal Structure of $Py{\cdot}Cl_2B{-}SiCl_3$ (3·Py)

Compound **3**·Py crystallizes as a solvent-free phase in the monoclinic space group $P2_1/c$ (No. 14) with one crystallographically unique molecule in a general position (Figure S110).

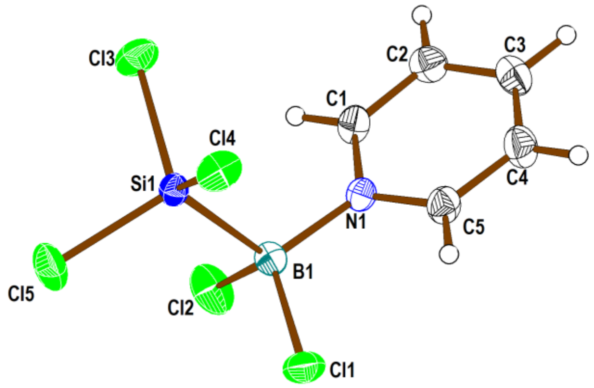

Figure S110: Molecular structure of **3**·Py in the solid state. Atomic displacement ellipsoids are drawn at the 50% probability level.

4.10. Crystal Structure of $Ph_3P{\cdot}Cl_2B–SiCl_3$ ($\mathbf{3}{\cdot}PPh_3$)

Compound **3**·PPh_3 crystallizes as a solvent-free phase in the triclinic space group *P*1 (No. 2) with two crystallographically unique molecules in general positions (Figure S111 a). Both molecules have a very similar structure (Figure S111 b).

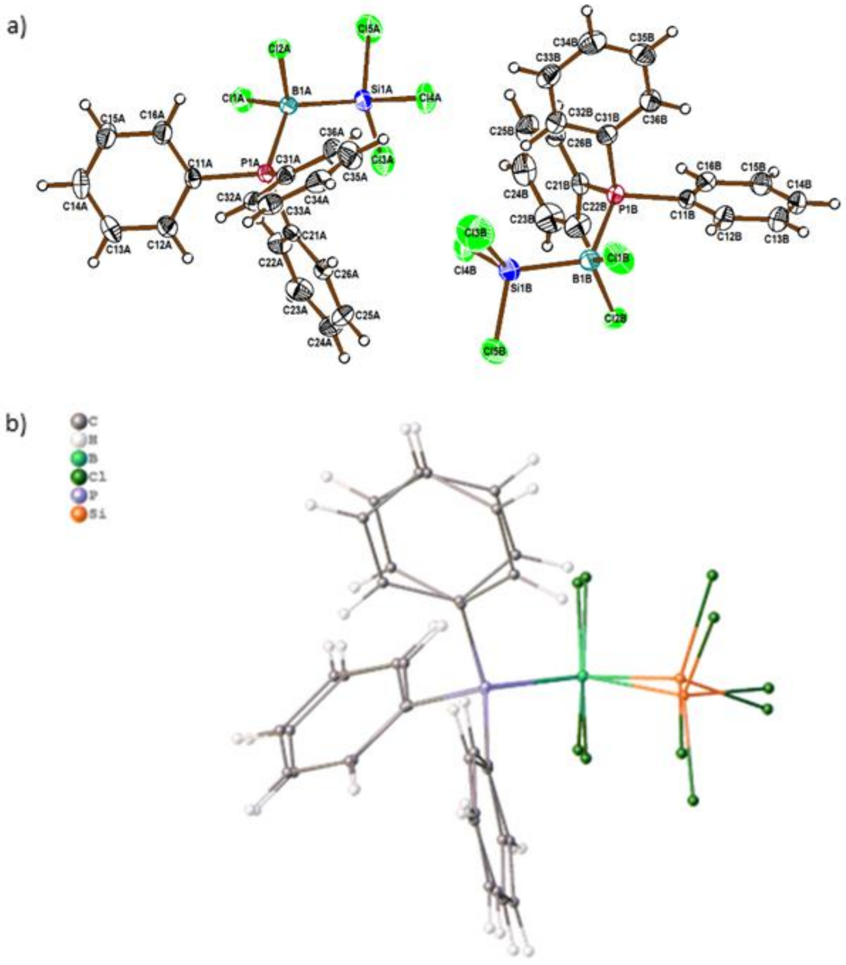

Figure S111: (a) Molecular structures of two crystallographically unique molecules of **3**·PPh_3 in the solid state. Atomic displacement ellipsoids are drawn at the 50% probability level. (b) Overlay of the two crystallographically unique molecules with maximal atom-to-atom fit.

4.11. Crystal Structure of IDipp·Cl_2B–$SiCl_3$ (3·IDipp)

Compound **3·**IDipp crystallizes as a solvent-free phase in the chiral polar trigonal space group $P3_2$ (No. 145) with three crystallographically unique molecules in general positions (Figure S112 a-c). They have similar geometrical characteristics (Figure S112 d).

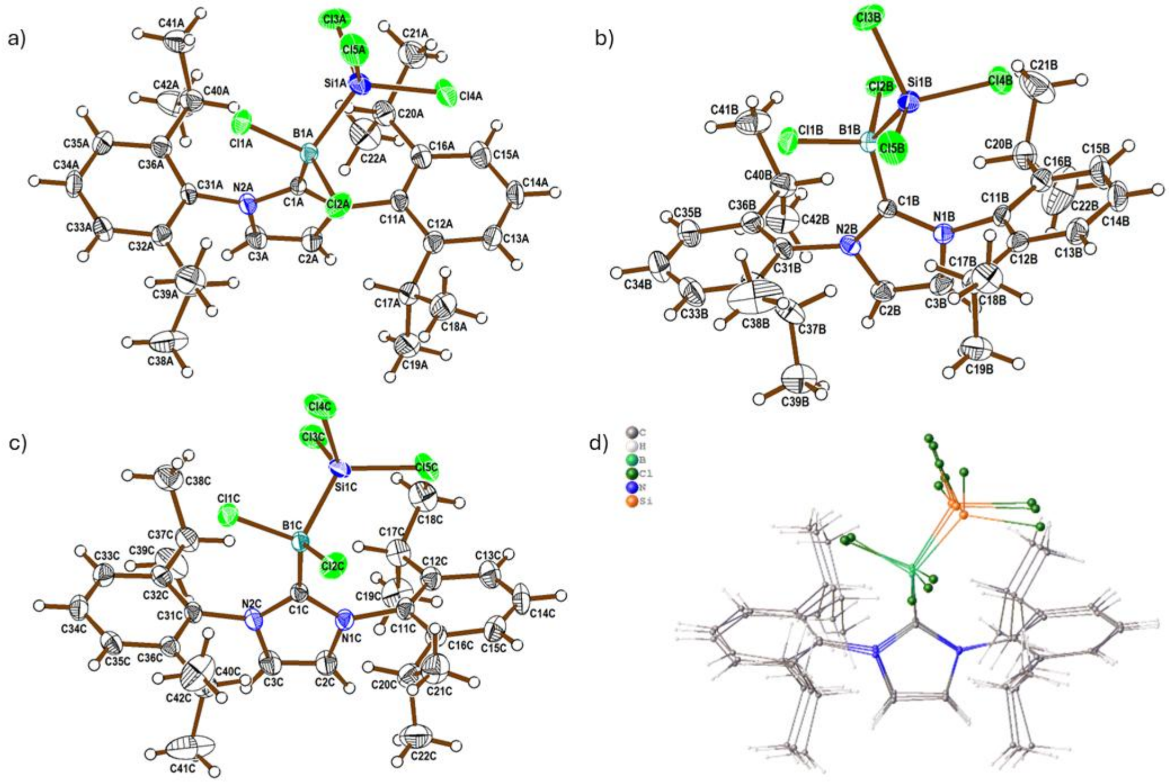

Figure S112: (a-c) Molecular structures of three crystallographically unique molecules of **3·**IDipp in the solid state. Atomic displacement ellipsoids are drawn at the 50% probability level. (d) Overlay of the three crystallographically unique molecules with maximal atom-to-atom fit.

4.12. Crystal Structure of BI_3·PPh_3

BI_3·PPh_3 crystallizes as a solvent-free phase in the trigonal space group *R3* (No. 148) with one crystallographically unique molecule at the 3-fold axis (Figure S113).

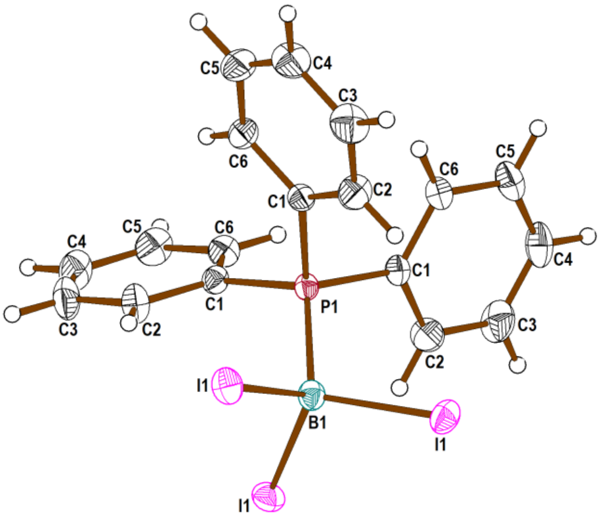

Figure S113: Molecular structure of BI_3·PPh_3 in the solid state. Atomic displacement ellipsoids are drawn at the 50% probability level.

4.13. Crystal Structure of BI_3·IDipp

BI_3·IDipp crystallizes as a solvent-free phase in the monoclinic space group $P2_1/c$ (No. 14) with one crystallographically unique molecule in a general position (Figure S114).

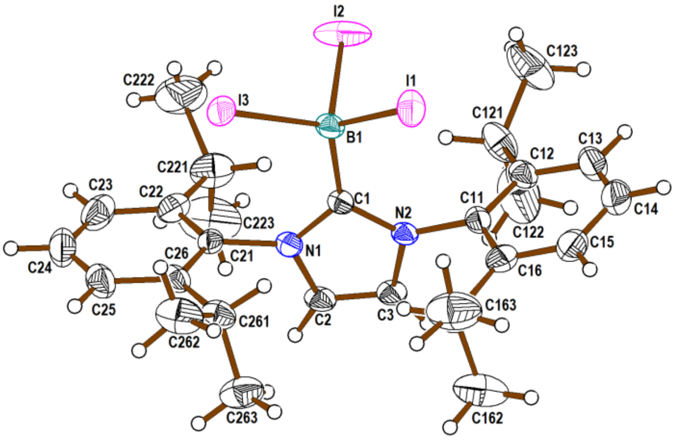

Figure S114: Molecular structure of BI_3·IDipp in the solid state. Atomic displacement ellipsoids are drawn at the 50% probability level.

4.14. Crystal Structure of $Me_2S·I_2B–C_2H_4–SiI_3$ (5·SMe2)

5·SMe2 crystallizes in the polar monoclinic space group *Pn* (No. 7) as a solvent free phase. The crystal consists of four types of twin domains, combining non-merohedry and racemic twinning (see chapter 4 - general considerations). The molecule occupies a general crystallographic position (Figure S115).

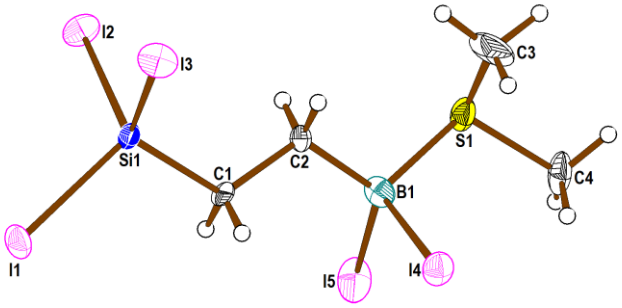

Figure S115: Molecular structure of **5**·SMe2 in the solid state. Atomic displacement ellipsoids are drawn at the 50% probability level.

4.15. Crystal Structure of $Py·I_2B–C_2H_4–SiI_3$ (5·Py)

5·Py crystallizes as a solvent-free phase in the monoclinic space group $P2_1/n$ (No. 14) with one crystallographically unique molecule in a general position (Figure S116).

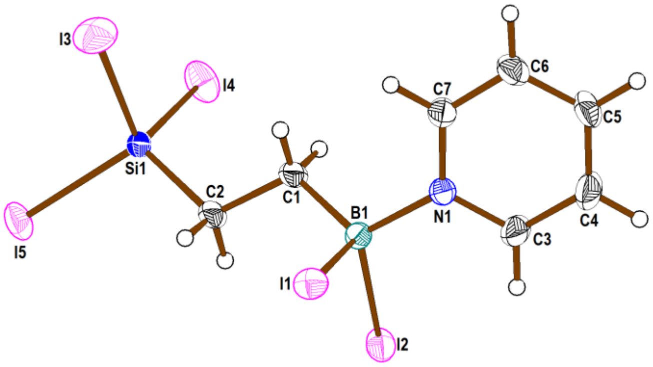

Figure S116: Molecular structure of **5**·Py in the solid state. Atomic displacement ellipsoids are drawn at the 50% probability level.

4.16. Crystal Structure of $(I_2B–)(I_3Si–)C_6H_{10}$ (7)

7 crystallizes as a solvent-free phase in two orthorhombic polymorphic modifications, α-**7** in space group *Pbca* (No. 61) and β-**7** in space group *Pca*2_1 (No. 29). In both cases, there is one crystallographically unique molecule in a general position (Figure S117).

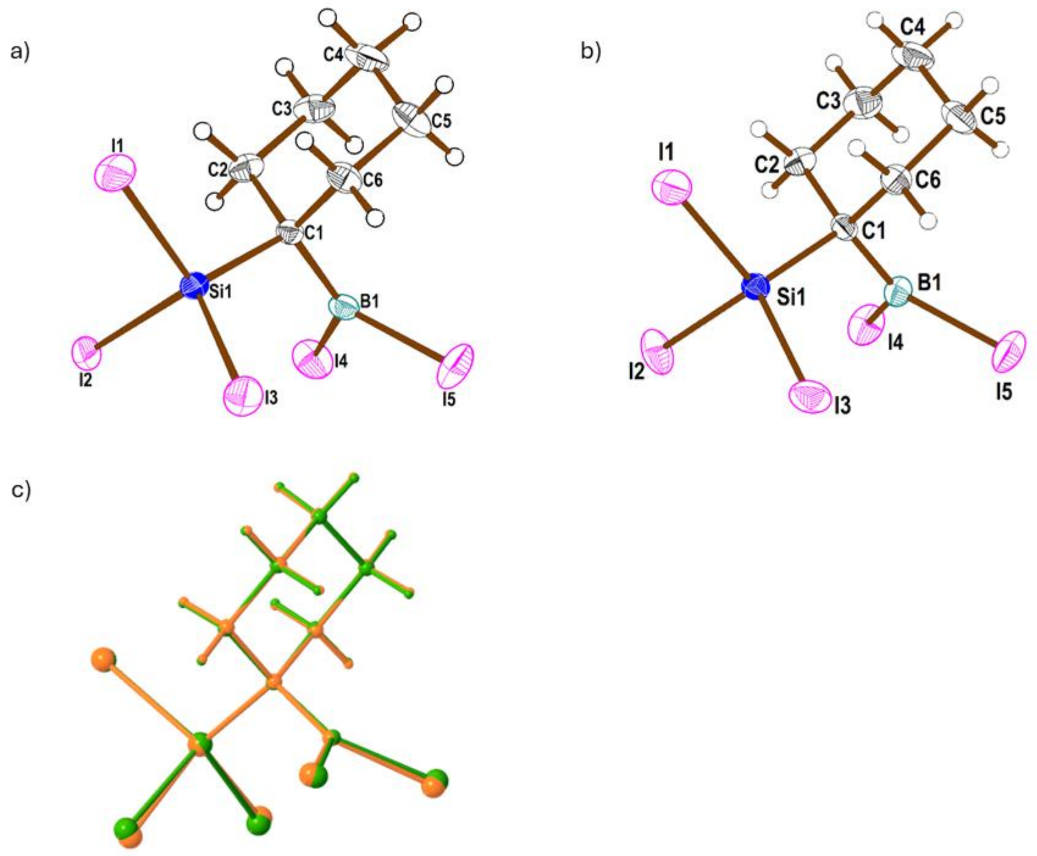

Figure S117: Molecular structure in two polymorphic forms of **7**: (a) α-**7** and (b) β-**7** in the solid state. Atomic displacement ellipsoids are drawn at the 50% probability level. (c) Overlay of the molecules in α- (orange) and β- (green) modifications with the best atom-to-atom fit.

4.17. Crystal Structure of $Me_2S{\cdot}I_2B–C_2H_4–I$

$Me_2S{\cdot}I_2B–C_2H_4–I$ crystallizes as a solvent-free phase in the monoclinic space group $P2_1/c$ (No. 14) with one crystallographically unique molecule in a general position (Figure S118).

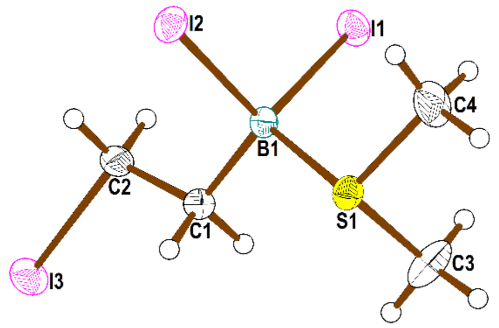

Figure S118: Molecular structure of $Me_2S{\cdot}I_2B–C_2H_4–I$ in the solid state. Atomic displacement ellipsoids are drawn at the 50% probability level.

4.18. Crystal Structure of 8

8 crystallizes as a solvent-free phase in the triclinic crystal system, space group $P\bar{1}$ (No. 2) (Figure S119) with one crystallographically unique molecule in a general position. The terminal phenyl group is disordered over two close positions with the relative weights refined as 0.754(12) to 0.246. As a result, some restraints must be applied during the structure refinement. Thus, due to the rather low contribution of the minor disorder component to the diffraction intensities, the C–C bond distances were softly restrained using a SADI instruction. In addition, the atomic displacement parameters of very close atomic positions C11A and C11B (separated by only ~0.3 Å) were forced to be equal using an EADP instruction. The C3 atom correlated with the split Ph group was impossible to split and refine with a reasonable geometry. Therefore, this disordered position was described with a reasonably large atomic displacement ellipsoid.

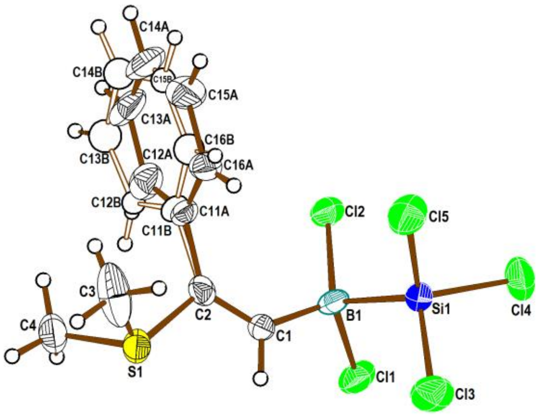

Figure S119: Molecular structure of **8** in the solid state. Atomic displacement ellipsoids are drawn at the 50% probability level. The minor position of the phenyl group is shown by the empty ellipses and bonds.

5. X-ray powder diffraction

General considerations

To determine the phase purity of the bulk material of **2**·SMe_2, **5**·SMe_2, and **7**, X-ray powder diffraction was performed at room temperature and the experimental diffractograms were compared with the simulated diffractograms from the single-crystal data collected at 173 K. Data were collected in Debye-Scherrer geometry on a *STOE STADI-P* transmission diffractometer equipped with a curved Ge(111) monochromator and a *Dectris Mythen 1K detector* (PSD) and processed with the *STOE WinX^{POW}* software.[S30] $MoK_{\alpha 1}$ radiation (λ = 0.7093 Å) was used. The sample was placed in capillary mark tube made from borosilicate glass no. 50 (Ø = 0.5 mm) and sealed under an Ar atmosphere. The capillary was mounted on the diffractometer and rotated during the measurement. The 2θ range was 2–70°, moving the detector in steps of 0.5° (2θ) with an exposure time of 20 s/step.

5.1. X-ray powder diffractometry on $Me_2S{\cdot}I_2B{-}SiI_3$ (2·SMe_2)

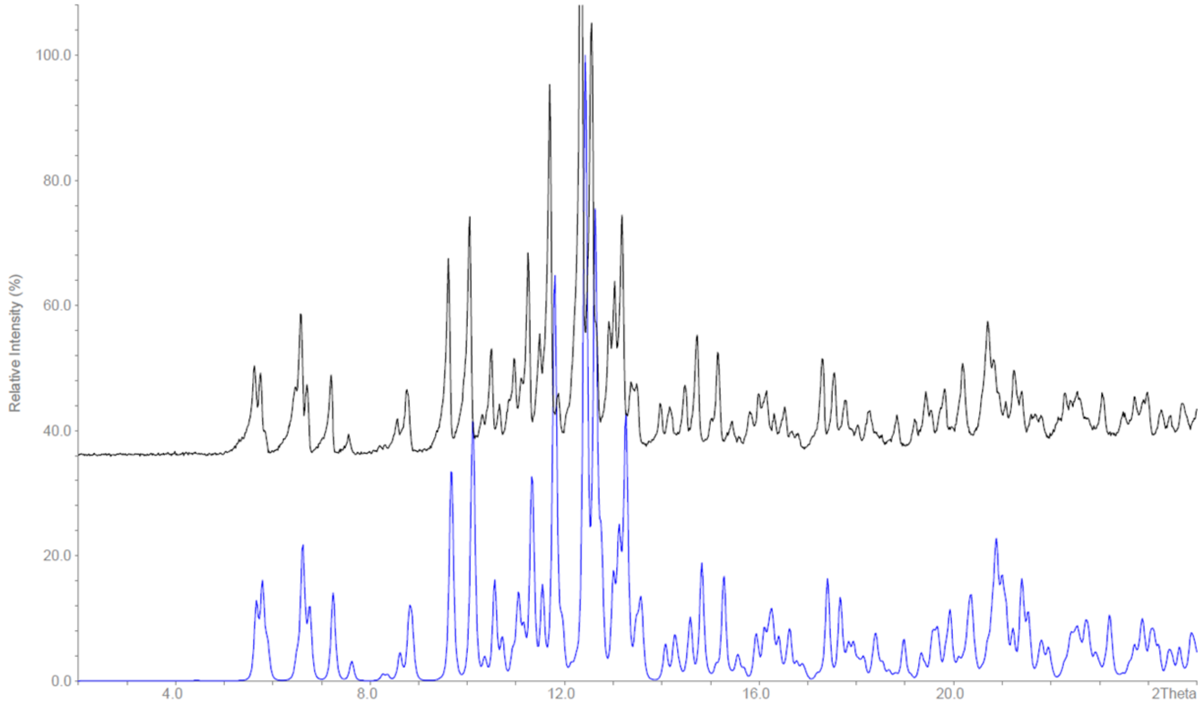

Figure S120: The recorded X-ray powder diffraction pattern (black; measured at rt) and the predicted pattern based on the single-crystal X-ray diffraction data (blue; measured at –100 °C) of β-**2**·SMe_2.

5.2. X-ray powder diffractometry on $Me_2S{\cdot}I_2B{-}C_2H_4{-}SiI_3$ (5·SMe2)

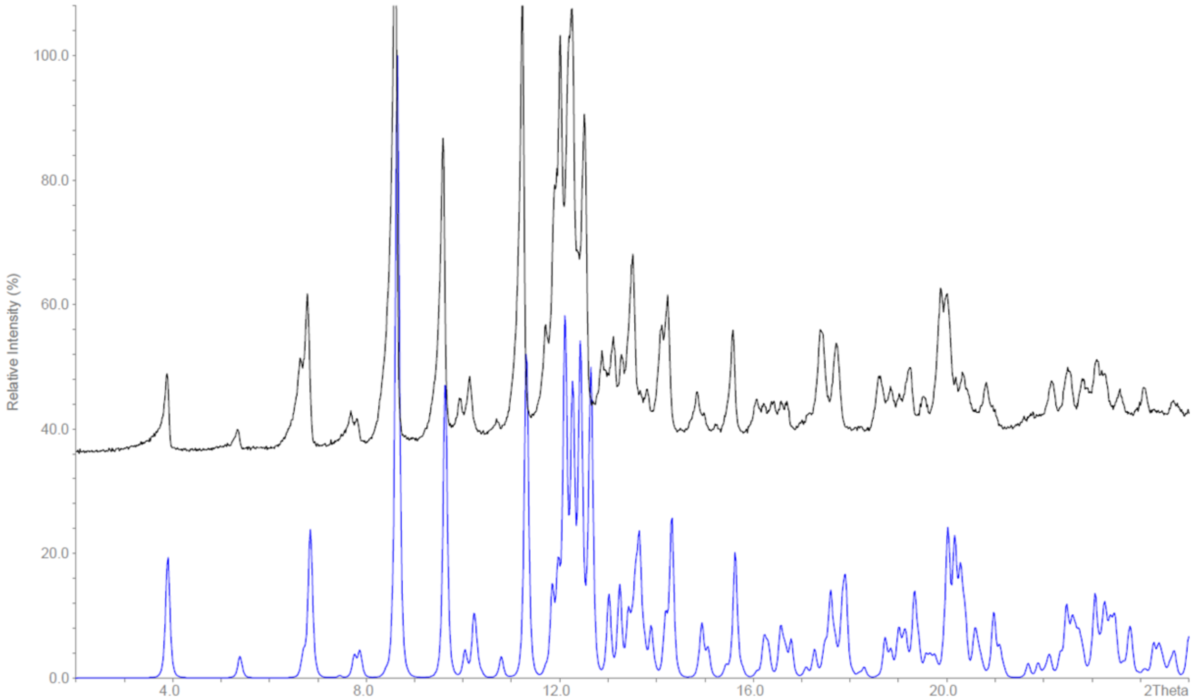

Figure S121: The recorded X-ray powder diffraction pattern (black; measured at rt) and the predicted pattern based on the single-crystal X-ray diffraction data (blue; measured at –100 °C) of **5**·SMe_2.

5.3. X-ray powder diffractometry on $(I_2B{-})(I_3Si{-})C_6H_{10}$ (7)

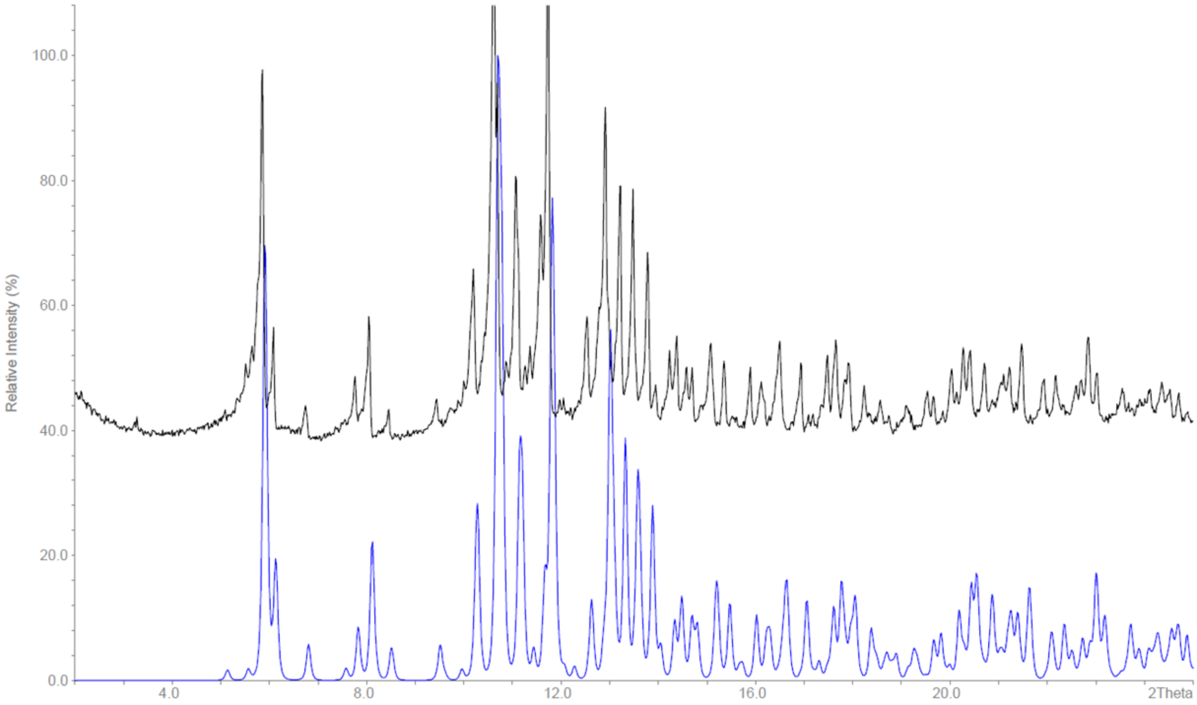

Figure S122: The recorded X-ray powder diffraction pattern (black; measured at rt) and the predicted pattern based on the single-crystal X-ray diffraction data (blue; measured at –100 °C) of β-**7**.

6. Computational details

All DFT calculations were performed using *Gaussian 16, Revision B.01*.[S31] Graphical representations of molecular geometries were produced with the *CYLview20* software.[S32] Graphical representations of frontier orbitals (HOMO/LUMO) and natural bond orbitals were produced with GaussView 6.0.16.[S33]

To determine appropriate methods for optimizing the structures investigated herein, we performed geometry optimizations of **2**·SMe_2 considering five distinct DFT functionals including implicit solvation by the solvent model based on density (SMD; solvent = DCM; ε = 8.930)[S34], namely B3LYP[S35,36]-D3(BJ)[S37,38], BP86[S39,40]-D3(BJ), M062X[S41]-D3, PBE0[S42,43]-D3(BJ) and ωB97X-D.[S44] The Ahlrich's def2-SVP or def2-SVPD basis set was used in these calculations.[S45,46] The optimized results were compared to the respective X-ray crystal structure of **2**·SMe_2. A comparison of the root-mean-square deviation (RMSD) values, which were obtained excluding hydrogen atoms, are shown in Table S16. Overall, SMD(DCM)/PBE0-D3(BJ)/def2-SVPD performed best among the tested theory levels. Optimized geometries were confirmed to be the desired minimum energy structures or transition states by vibrational frequency analysis.

Table S16: Root-mean-square deviations (RMSDs) between DFT-optimized geometries of **2**·SMe_2 and its experimentally determined solid-state structure using various functionals and basis sets.

**2**·SMe_2	B3LYP-D3(BJ)	BP86-D3(BJ)	M062X-D3	PBE0-D3(BJ)	ωB97X-D
def2-SVP	0.08109733	0.07145311	0.05769571	0.04662624	0.05566557
def2-SVPD	0.07626054	0.06675085	0.06494409	**0.04121097**	0.06165401

Single-point energy calculations at the SMD(DCM)/PBE0-D3(BJ)/def2-QZVPPD level were conducted to compute the free energies of the species along the proposed mechanistic pathway.[S45,46] This functional was selected to maintain consistency with the level of theory used for geometry optimization, and because PBE0-D3(BJ) is known to perform well for systems with similar characteristics.[S10] All free energy values were calculated for the corresponding experimental temperature (25 °C) and included a concentration correction accounting for the change in standard states going from gas phase to condensed phase.[S34,47,48]

6.1. Computed mechanism of the reaction of 2·SMe_2 with ethylene

The first step in the reaction of **2**·SMe_2 with C_2H_4 requires a vacant coordination site, i.e., a free B-p_z orbital, to enable π-complex formation with C_2H_4. The Gibbs free energy for donor dissociation (**2**·SMe_2 → **2** + SMe_2) is endergonic by ΔG = 14.6 kcal/mol. Since the overall reaction barrier appears to be significantly influenced by the presence of trace amounts of BI_3, the ΔG for SMe_2 abstraction by BI_3 (**2**·SMe_2 + BI_3→ **2** + BI_3·SMe_2) was also calculated.

Figure S134 in chapter 6.1.7 compares both reactions. The addition of BI_3 lowers the overall energy required to generate the active silaboration species **2** by 9.8 kcal/mol, resulting in a total ΔG of only 4.8 kcal/mol. This significant reduction in activation energy facilitates the 1,2-silaboration reaction under ambient conditions.

6.1.1. Adduct formation of 2 with C_2H_4

The next step involves formation of a π-complex between **2** and C_2H_4. To model this interaction, C_2H_4 was positioned in close proximity to the vacant B-p_z orbital. Under these conditions, a minimum structure for the complex **2**·C_2H_4 was located (Figure S123). The Gibbs free energy change for this step is 5.4 kcal/mol indicating a slightly endergonic process, likely due to significant rehybridization at the B center. For a NBO analysis of **2**·C_2H_4 and comparison with **2**·C_6H_{10} see section 6.3.

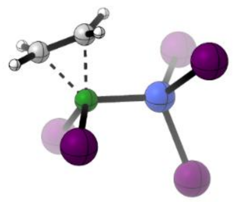

Figure S123: Optimized structure of **2**·C_2H_4.

6.1.2. 1,2-silaboration

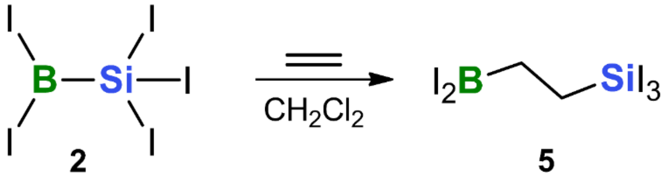

2 **5**

Starting from the optimized **2**·C_2H_4 complex, a relaxed scan was performed by stepwise elongation of the B–Si bond. This procedure yielded a structure from which the TS for the 1,2-silaboration step could be located upon reoptimization. The resulting TS exhibits a pronounced imaginary frequency of 205.5*i* cm^{-1}, characteristic for the B−Si bond cleavage and C−C bond activation process (Figure S124).

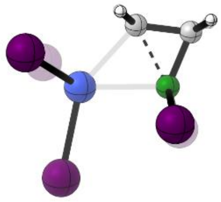

Figure S124: Optimized TS for the 1,2-silaboration of C_2H_4.

The IRC calculation confirms a concerted process in which C–B and C–Si bonds are formed simultaneously, leading directly to the 1,2-silaboration product (Figure S125). The overall barrier for the 1,2-silaboration pathway, starting from **2**·SMe_2, amounts to 15.7 kcal/mol, and the formation of product **5** is exergonic by –21.7 kcal/mol.

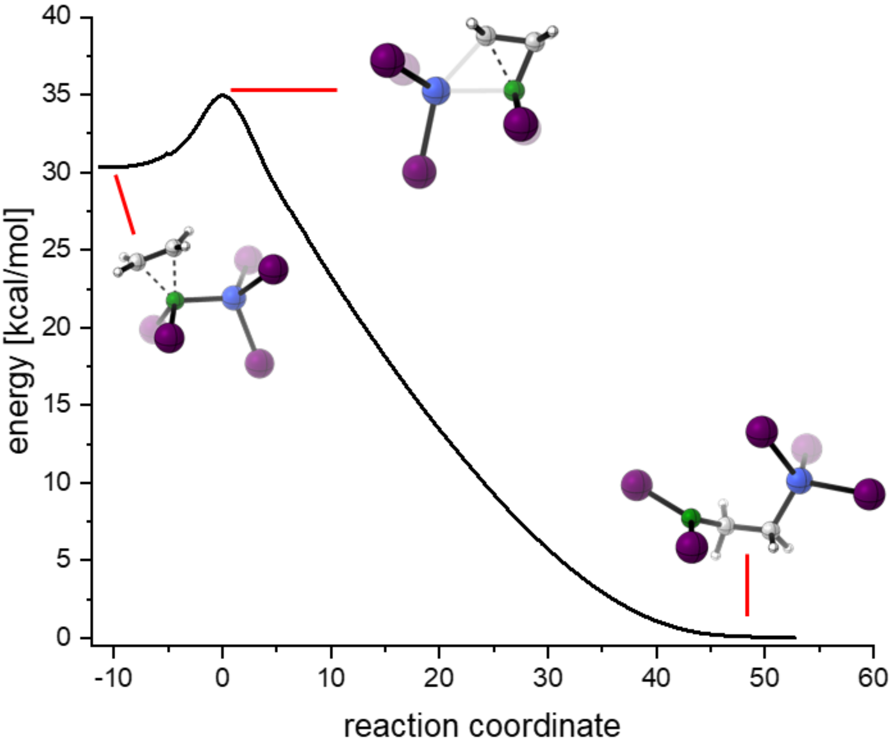

Figure S125: The IRC scan reveals the concerted formation of B–C and Si–C bonds during the 1,2-silaboration of C_2H_4.

6.1.3. 1,2-iodoboration

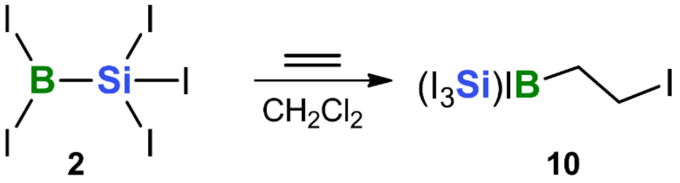

A plausible alternative pathway is 1,2-iodoboration. To rationalize its absence in the reaction of **2** with C_2H_4, this pathway was also investigated by DFT calculations. Starting from the optimized **2**·C_2H_4 complex, a relaxed scan was carried out by stepwise elongation of the B–I bond. This approach yielded a structure from which the TS for 1,2-iodoboration could be located upon reoptimization (Figure S126). The resulting TS exhibits a pronounced imaginary frequency of 217.6*i* cm^{-1}, corresponding to the bond reorganization along the reaction coordinate.

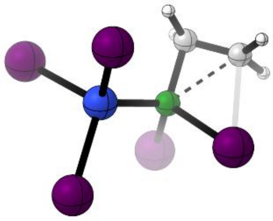

Figure S126: Optimized TS for the 1,2-iodoboration of C_2H_4.

Performing the IRC scan starting from the optimized transition state reveals simultaneous C–B and C–Si bond formation (Figure S127). This process ultimately yields the desired 1,2-iodoboration product. The overall barrier for the 1,2-iodoboration pathway, starting from **2**·SMe_2, is 14.3 kcal/mol; however, the reaction is endergonic by +3.7 kcal/mol, which explains why this reaction is not observed experimentally.

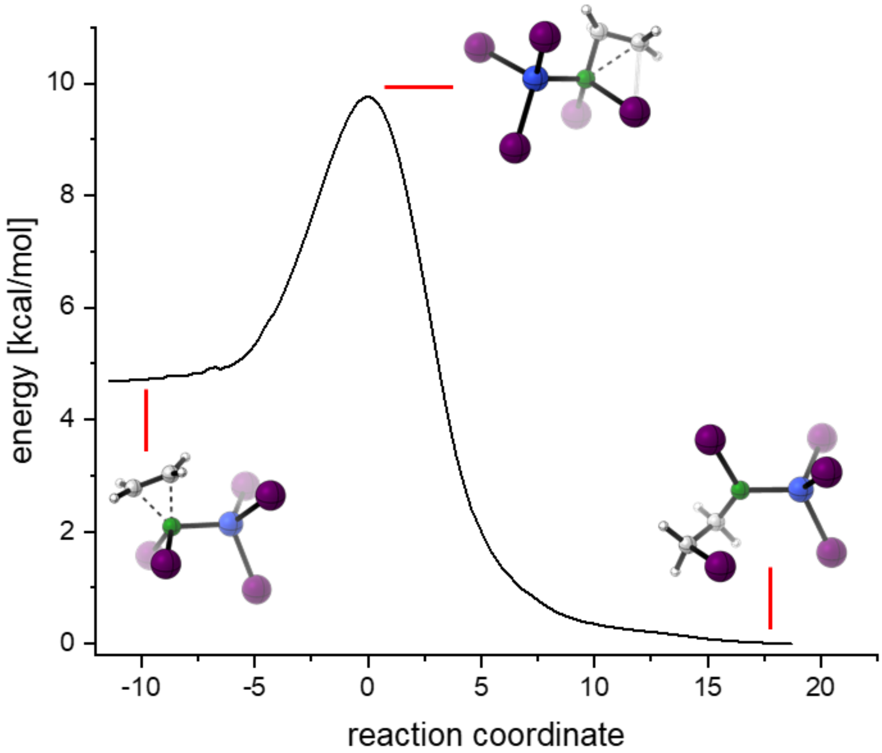

Figure S127: The IRC scan reveals the concerted formation of the B–C and I–C bonds during the 1,2-haloboration of C_2H_4.

6.1.4. 1,2-thiaboration

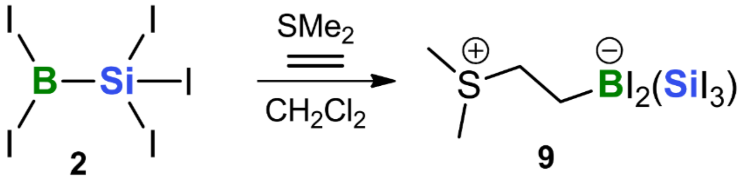

2 **9**

1,2-Thiaboration represents a chemically plausible alternative pathway. To rationalize its absence in the reaction of **2**·SMe_2 with C_2H_4, this pathway was also explored using DFT calculations. The preoptimized complex **2**·C_2H_4 and an SMe_2 molecule were placed in close proximity, with an initial S···C distance of 3.0 Å. This distance was chosen based on literature reports of related FLP systems displaying comparable reactivity at similar distances in the transition state.[S49] Geometry optimization under constraints yielded a structure from which the TS for the thiaboration pathway could be located (Figure S128). Successful reoptimization of this TS required freezing the S···C distance at 2.29 Å. The resulting TS exhibits a pronounced imaginary frequency of 159.5*i* cm^{-1}.

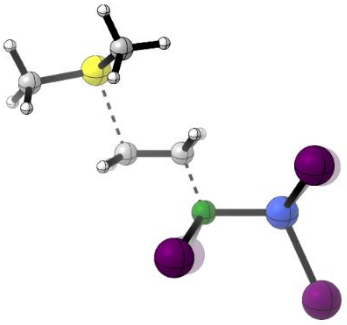

Figure S128: Optimized TS for the 1,2-thiaboration of C_2H_4.

An IRC calculation starting from the optimized transition state reveals a concerted mechanism involving simultaneous C–S and C–B bond formation, ultimately leading to the 1,2-thiaboration product (Figure S129). Notably, product formation is observed in both directions along the IRC path, likely reflecting the nearly barrierless nature of the reaction when free SMe_2 is present.

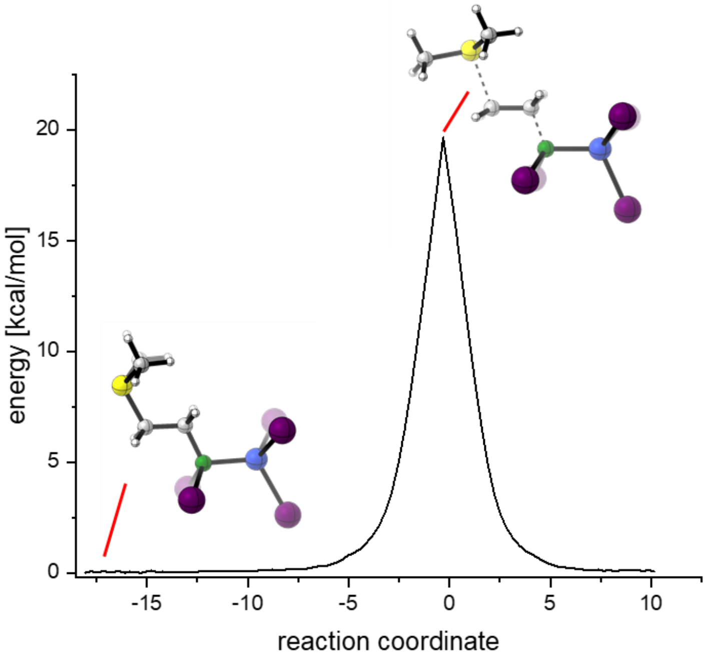

Figure S129: The IRC scan reveals the concerted formation of the B–C and S–C bonds during the 1,2-thiaboration of C_2H_4.

Due to the absence of free SMe_2 under the reaction conditions, the reaction sequence was evaluated computationally with either free SMe_2 or **2**·SMe_2 serving as the SMe_2 donor (Figure S130).

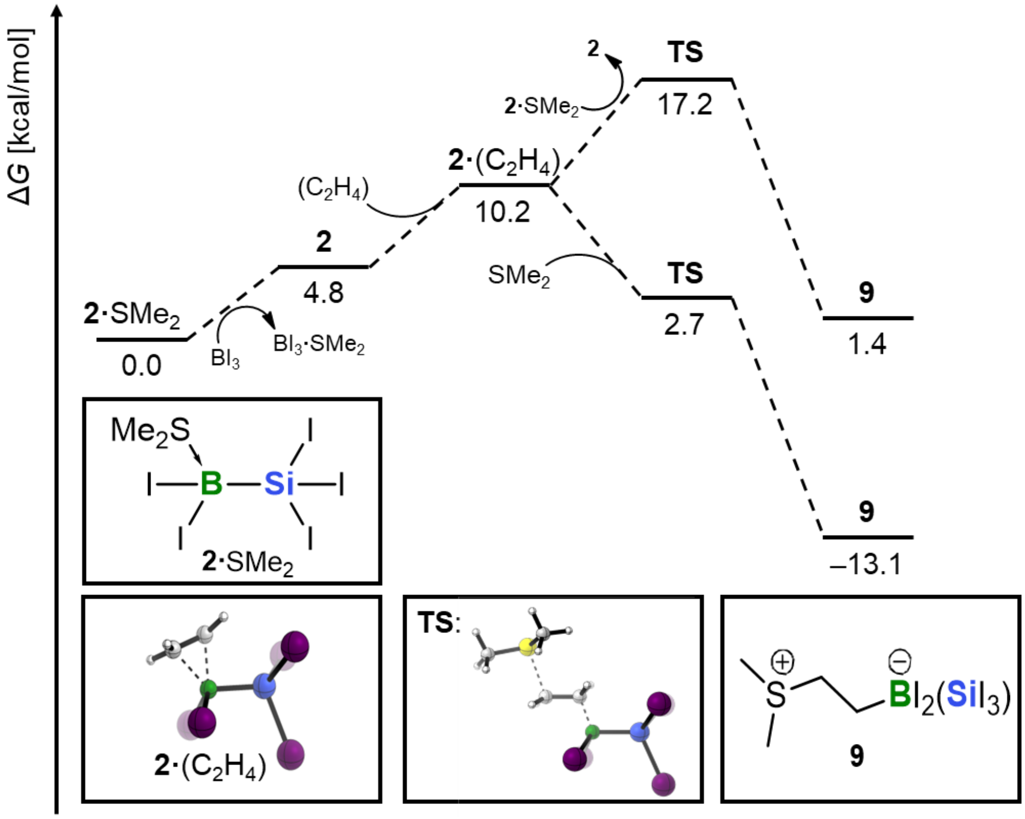

Figure S130: Schematic representation of the thiaboration pathway when (a) free SMe_2 is present or (b) **2**·SMe_2 acts as the SMe_2 donor. Since **2**·SMe_2 is the only plausible SMe_2 source under the applied reaction conditions, the 1,2-thiaboration becomes endergonic, which explains why it is not observed in this case.

Figure S130 illustrates the following trend: In the reaction of **2**·C_2H_4 with free SMe_2, the process proceeds without a barrier and is overall exergonic by –13.1 kcal/mol, making it thermodynamically feasible under ambient conditions. However, this reaction is not observed experimentally due to two key factors: (i) no free SMe_2 is present under the applied conditions, and (ii) even in the presence of excess SMe_2, formation of the stable **2**·SMe_2 complex would be strongly favored over all other pathways, thereby preventing any further reactivity.

To obtain a more realistic representation of the system, the reaction was also modeled using **2**·SMe_2 as the SMe_2 source. Under these conditions, the process proceeds with an activation barrier of 7.0 kcal/mol. While this barrier is accessible at room temperature, the overall reaction is slightly endergonic (ΔG = +1.4 kcal/mol), which accounts for the absence of 1,2-thiaboration products in the reaction of **2**·SMe_2 with C_2H_4.

6.1.5. 1,1-silaboration

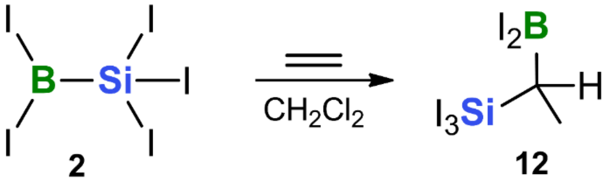

Reactions of **2** with C_6H_{10} show a 1,1-silaboration product instead of the observed 1,2-silaboration product in the C_2H_4 case (For more information see chapter 6.2). To elucidate why no 1,1-silaboration is observed in reactions of **2**·SMe_2 with C_2H_4, the analogous reaction pathway, as was found for the reaction of **2** with C_6H_{10}, was calculated.

Starting from the **2**·C_2H_4 complex, the TS for a 1,2-hydride shift was located via a stepwise scan involving elongation of the C–H bond. During this constrained optimization, the B–Si bond distance was fixed. A structure obtained from this scan was subsequently reoptimized under tight convergence criteria, allowing identification of the TS (Figure S131). The TS exhibits a pronounced imaginary frequency of 510.8*i* cm^{-1}.

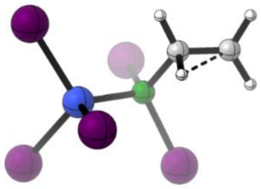

Figure S131: Optimized TS for the 1,2-hydride shift in **2**·C_2H_4.

An IRC scan starting from the optimized transition state reveals hydride migration leading to the formation of a carbenium ion coordinated to a formally negatively charged boron atom.

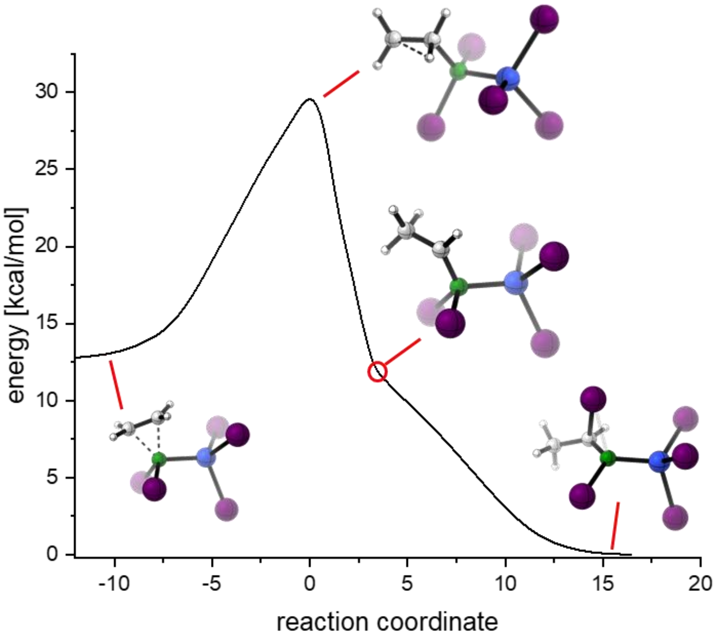

Figure S132: IRC scan starting from the optimized transition state, showing hydride migration and formation of a carbenium ion coordinated to a formally anionic boron center. The scan terminates at the 1,1-haloboration product.

The overall barrier for this reaction, starting from **2**·SMe_2, amounts to 25.2 kcal/mol and is therefore not observed despite the reaction being overall exergonic.

The proposed intermediate—formed immediately after the 1,2-hydride shift and featuring a carbenium ion coordinated to the formally negatively charged boron atom—could not be located as a minimum on the potential energy surface. IRC scans and relaxed scans (Figure S132) reveal that from this intermediate the iodide shift proceeds barrierlessly, as the reaction path continuously descends without any energy increase, not indicating a further transition state. Consequently, all geometry optimizations led directly to the 1,1-silaboration or iodoboration product, indicating that this step is barrierless.

Due to the prohibitively high barrier for the initial 1,2-hydride shift under the applied conditions, further transition state searches along this pathway were deemed unnecessary and chemically irrelevant.

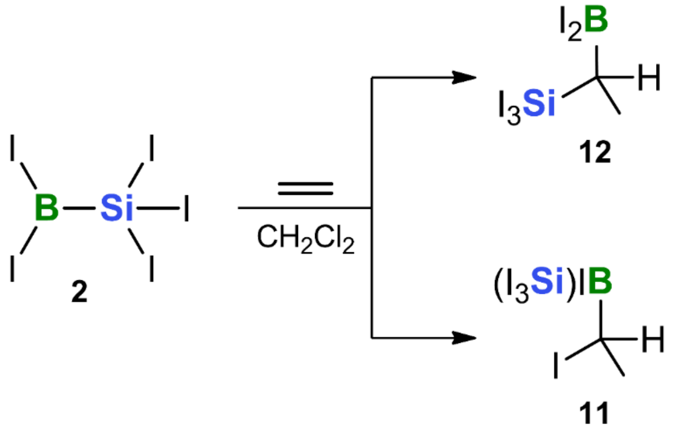

Nevertheless, both the 1,1-silaboration **12** and 1,1-iodoboration **11** products were computed. The corresponding reaction sequences, originating from the respective TS for the 1,2-hydride shift and assuming barrierless 1,2-iodide and -silyl shifts, are depicted in Figure S134 in chapter 6.1.7.

6.1.6. Δ*G* profiles of SMe_2 adduct formations

Since the experimentally observed product is consistently isolated as the SMe_2-stabilized adduct, the corresponding Δ*G* values for SMe_2 coordination were subsequently calculated. As no free SMe_2 is present under the applied reaction conditions, **2**·SMe_2 was considered the most plausible SMe_2 donor. Figure S133 compares the Δ*G* values for the formation of SMe_2 adducts with all calculated products, either via coordination of free SMe_2 (yellow) or by SMe_2 abstraction from **2**·SMe_2 (red). All starting compounds were set to a relative energy of 0.0 kcal/mol and Δ*G* values for SMe_2 adduct formation via both pathways were evaluated.

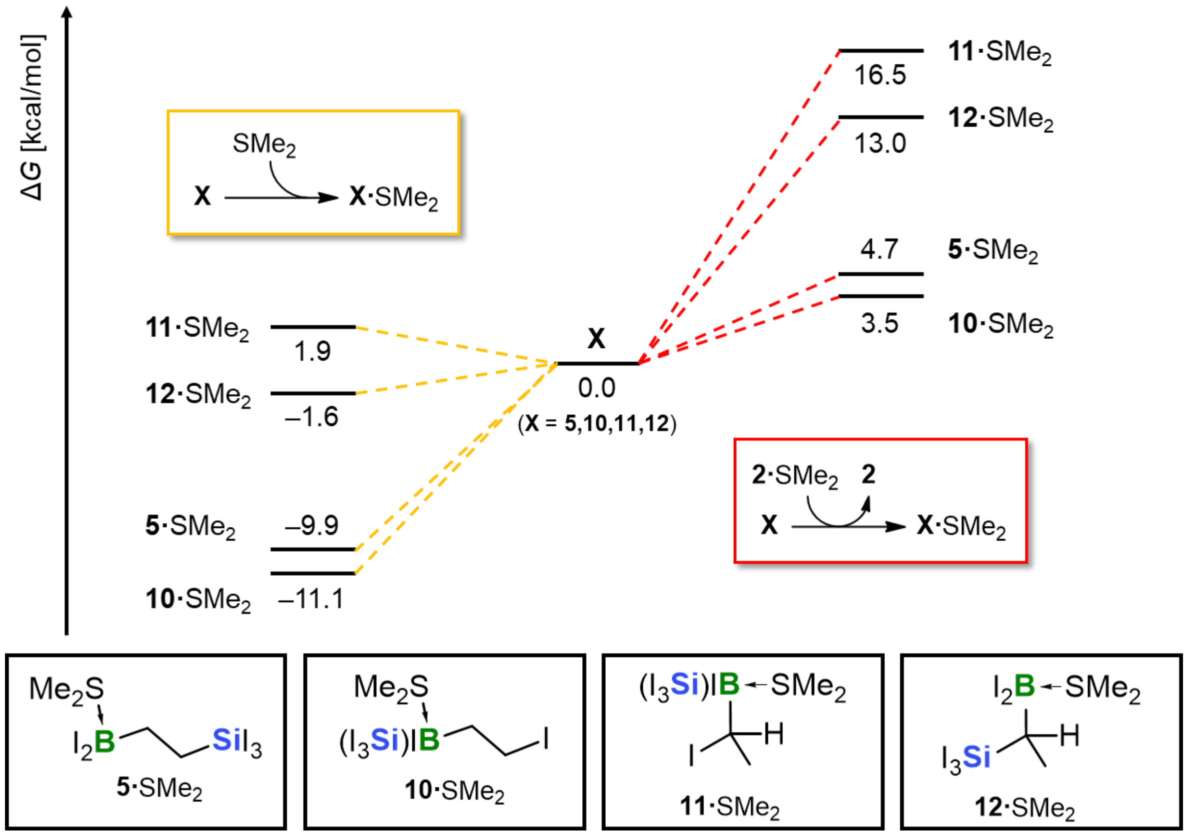

Figure S133: Comparison of Δ*G* values for SMe_2 adduct formation with all calculated products, either via free SMe_2 coordination (yellow) or SMe_2 abstraction from **2**·SMe_2 (red). All starting compounds are set to 0.0 kcal/mol.

The SMe_2 adduct formations with the 1,1-products are approximately thermoneutral (Δ*G* = −1.6 and 1.9 kcal/mol, respectively) even when free SMe_2 is available. This is likely due to increased steric hindrance in the corresponding adducts.

Notably, the energy required for SMe_2 abstraction from **2**·SMe_2 by the experimentally relevant 1,2-product **5** is comparable to that required for abstraction by BI_3 (Δ*G* = 4.7 and 4.8 kcal/mol, respectively). As a result, once a small amount of free **2** is generated through initial activation of **2**·SMe_2 by BI_3, it reacts with C_2H_4 to form the 1,2-silaboration product **5**. This species can then abstract SMe_2 from **2**·SMe_2, thereby regenerating free **2**. At this stage, the reaction becomes autocatalytic.

6.1.7. Summarized mechanism of 2·SMe₂ with C_2H_4

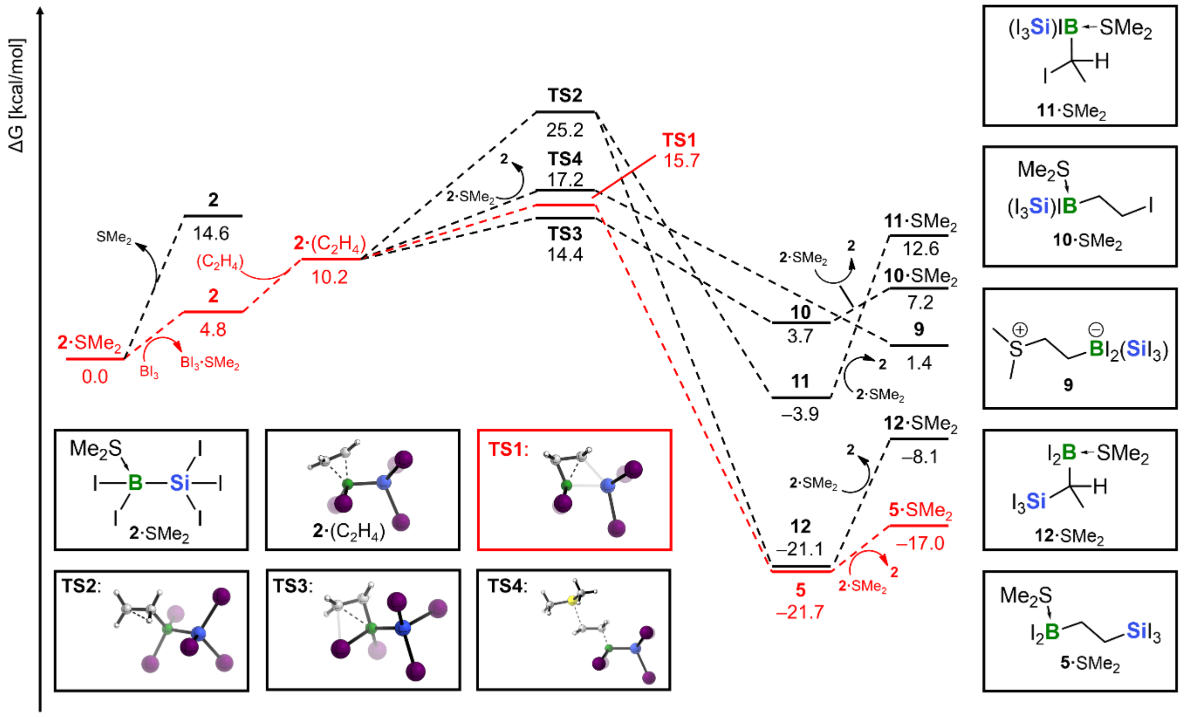

Figure S134: Computed reaction pathways for the transformation of **2**·SMe₂ with C_2H_4. While several pathways exhibit activation barriers that are surmountable at room temperature, only the experimentally observed 1,2-silaboration pathway (highlighted in red) is both kinetically feasible and strongly exergonic (Δ*G* = –17.0 kcal/mol).

6.2. Computed mechanism of the reaction of 2 with cyclohexene

In the following, all reactions were computed starting from free **2**. This assumption is justified by solid-state NMR data, which revealed that a mixture of Li[**1**] and **2**/LiI is already present in the solid state (see chapter 2.4). Upon addition of a donor (in this case, C_6H_{10}), LiI elimination is further favored. Therefore, this initial step was not considered explicitly, and all subsequent calculations were performed starting from free **2**.

6.2.1. Adduct formation of 2 with C_6H_{10}

The first step involves formation of a complex between **2** and C_6H_{10}. To model this interaction, C_6H_{10} was positioned in close proximity to the vacant B–p_z orbital. Under these conditions, a minimum structure for the complex **2**·C_6H_{10} was located (Figure S135). The Gibbs free energy change for this step is 11.3 kcal/mol, indicating a slightly endergonic process, likely due to significant reorganization at the boron center and considerable steric demand within the complex. Notably, the optimized structure is not a classical π-complex but rather a σ-complex, as evidenced by increased tetragonalization of the α-carbon atom relative to **2**·C_2H_4. In contrast, the β-carbon remains trigonal planar, consistent with carbenium ion character. For a detailed NBO analysis of **2**·C_6H_{10} and its comparison with **2**·C_2H_4, see section 6.3.

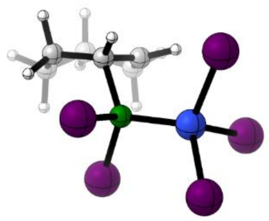

Figure S135: The optimized structure of **2**·C_6H_{10} reveals a zwitterionic species in which the β-carbon exhibits carbenium ion character, while the α-carbon is significantly sp³-hybridized.

6.2.2. 1,2 hydride shift in 2·C_6H_{10} and subsequent 1,2 silyl shift

To obtain the 1,1-silaboration product **7**, a hydride shift must occur at some point in the reaction pathway. As already discussed in section 6.2.1, the complex **2**·C_6H_{10} possesses all the prerequisites for such a hydride shift to proceed directly as the initial step of this reaction sequence. Starting from the optimized structure of **2**·C_6H_{10}, a relaxed scan was performed by stepwise increasing the C−H distance while simultaneously freezing the B−Si bond. A structure was obtained from which the TS for the 1,2-hydride shift could be identified after reoptimization (Figure S136). The TS exhibits an imaginary frequency of 146.1*i* cm^{-1}.

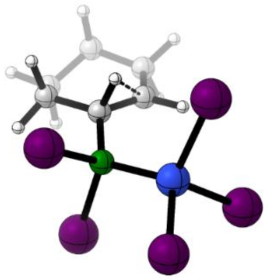

Figure S136: Optimized TS for the 1,2-hydride migration in complex **2**·C_6H_{10}.

An IRC scan starting from the optimized transition state reveals hydride migration leading to the formation of a carbenium ion coordinated to a formally negatively charged boron atom (Figure S137).

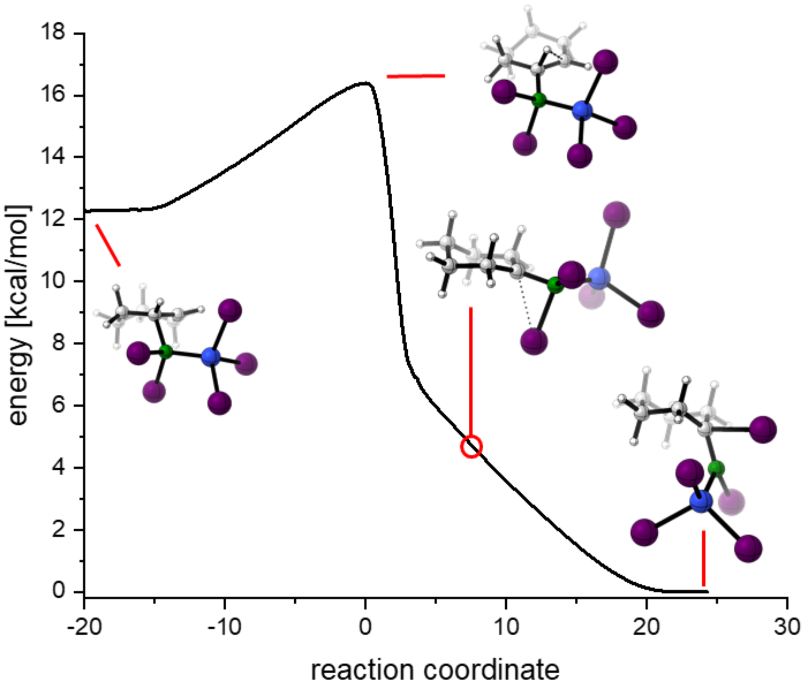

Figure S137: IRC scan following the hydride migration step, revealing a barrierless 1,2-iodide shift leading to the 1,1-iodoboration product **14**. A stable intermediate (**Int1**), likely stabilized by the β-silicon effect, forms directly after hydride migration and precedes the iodide shift.

The IRC following the hydride migration step reveals an immediate, barrier-free 1,2-iodide shift. This process is confirmed to be barrierless by both IRC and relaxed scans. The overall formation of the 1,1-iodoboration product **14** is slightly endergonic by 1.5 kcal/mol. When the positions of the migrating iodide and the SiI_3 group are swapped, subsequent geometry optimization yields a minimum structure, indicating the formation of a relatively stable intermediate after the hydride shift, likely further stabilized by the β-Si effect. Notably, the minimum structure for **Int1** is 7.9 kcal/mol lower in energy than the starting complex **2**·C_6H_{10}. The overall barrier for this hydride shift, starting from compound **2,** is 15.2 kcal/mol, indicating that the reaction is feasible at rt.

To obtain the final product **7** of the 1,1-silaboration, a subsequent 1,2-silanide shift must occur. Starting from the optimized intermediate species **Int1**, we performed a relaxed scan by stepwise lowering the $C^+\cdots SiI_3$ distance. A structure was obtained from which the TS for the 1,2-silanide shift could be identified after reoptimization (Figure S138). The TS exhibits an imaginary frequency of 150.4*i* cm^{-1}.

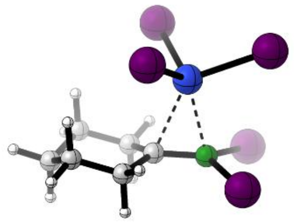

Figure S138: Optimized TS for the 1,2-silyl shift starting from **Int1**.

An IRC scan starting from the optimized transition state reveals silanide migration leading to the formation of compound **7** (Figure S139). The barrier for this reaction starting from **Int1** is 4.6 kcal/mol, and the reaction is exergonic by −14.3 kcal/mol.

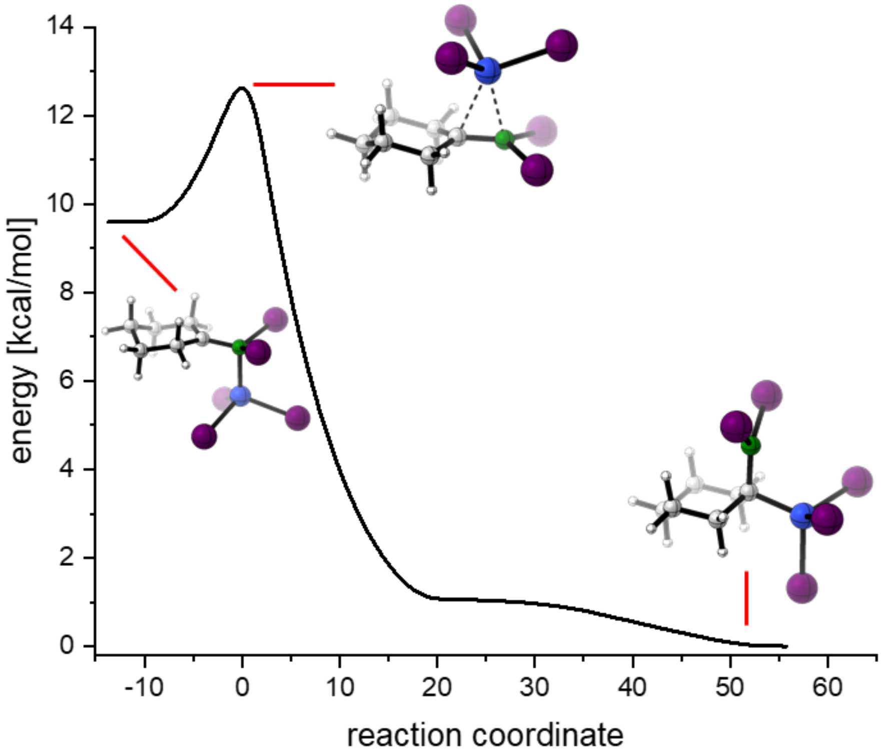

Figure S139: IRC scan starting from the optimized TS, showing silanide migration leading to the formation of compound **7**.

6.2.3. Transition state for 1,2-silaboration in 2·C_6H_{10}

In reactions of **2**·SMe_2 with C_2H_4, only the 1,2-silaboration product is observed. In contrast, the reaction of **2** with C_6H_{10} furnishes exclusively the 1,1-silaboration product, with no detectable formation of the 1,2-isomer. A quantum chemical evaluation of this process was therefore undertaken to elucidate the origin of this pronounced selectivity.

To locate the TS for the 1,2-silaboration of C_6H_{10}, the preoptimized σ-complex **2**·C_6H_{10} was used as the starting point for a relaxed scan in which the B–Si bond was gradually elongated. From this scan, a structure was obtained, from which the TS could be identified after reoptimization (Figure S140). The TS exhibits an imaginary frequency of 223.4*i* cm^{-1}.

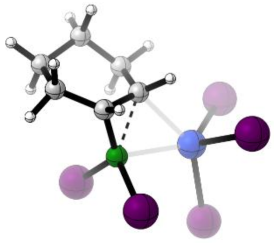

Figure S140: Optimized TS for the 1,2-silaboration of C_6H_{10}.

An IRC scan starting from the optimized transition state reveals concerted C–B and C–Si bond formation, leading to the formation of the 1,2-silaboration product **13** (Figure S141). The barrier for this reaction, starting from **2**, is 27.6 kcal/mol and is thus not feasible under ambient conditions. Although the overall reaction is exergonic (ΔG = –7.5 kcal/mol), the activation barrier is prohibitively high, likely due to significant steric hindrance in the TS that prevents optimal orbital overlap.

In conclusion, the absence of 1,2-silaboration in the reaction of **2** with C_6H_{10} can be attributed to this kinetic limitation, as the barrier is 12.4 kcal/mol higher than the highest barrier along the competing 1,1-silaboration pathway (see section 6.2.4).

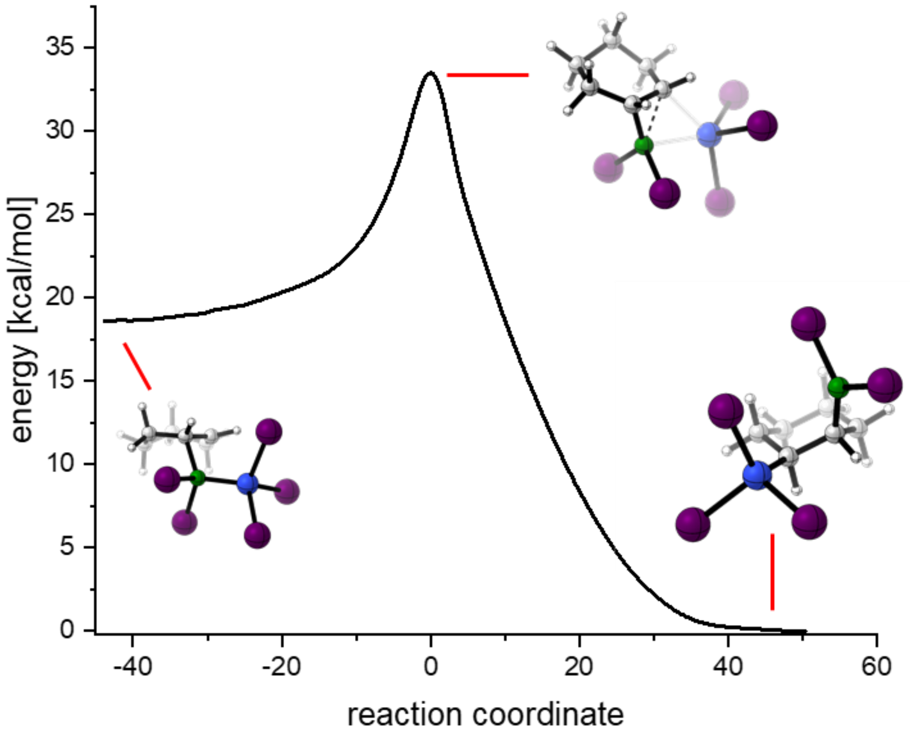

Figure S141: The IRC scan starting from the optimized transition state reveals the concerted formation of B–C and Si–C bonds during the 1,2-silaboration of C_6H_{10}.

6.2.4. Summarized mechanism of the reaction of 2·with C_6H_{10}

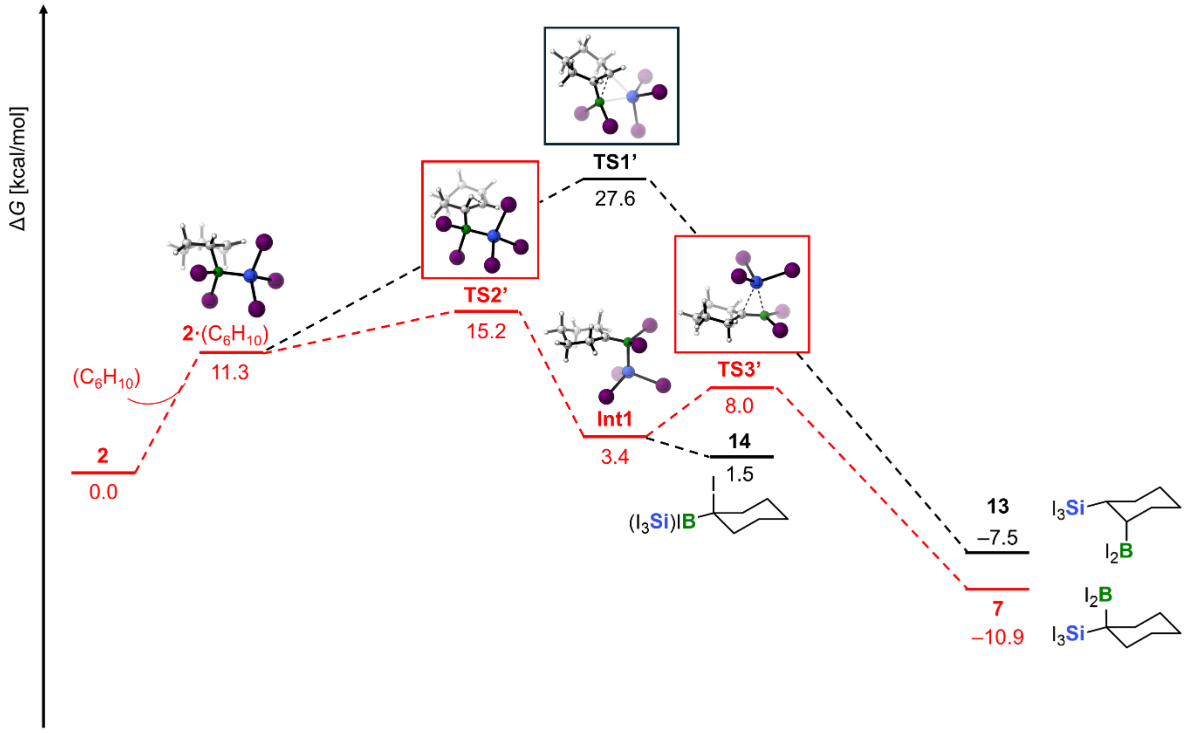

Figure S142: Computed reaction pathways for the transformation of **2** with C_6H_{10}. The pathway leading to the experimentally observed 1,1-silaboration product **7** (red) features a maximum barrier of 15.2 kcal/mol and is exergonic by −10.9 kcal/mol, rendering it both feasible at room temperature and thermodynamically favorable. In contrast, the pathway leading to the experimentally unobserved 1,2-silaboration product **13** (black) has a significantly higher activation barrier of 27.6 kcal/mol. Although the formation of **13** is exergonic by −7.5 kcal/mol, the barrier is prohibitively high under the applied conditions, explaining why this product is not observed.

6.3. Natural bond orbital (NBO) analysis

The bonding situations in the complexes **2**·C_2H_4 and **2**·C_6H_{10} differ fundamentally. To assess the key donor–acceptor interactions in each structure, Natural Bond Orbital (NBO)[S50] analyses were performed. These analyses provide insight into the origins of the distinct bonding patterns and the factors that stabilize each complex. Furthermore, donor–acceptor interactions in intermediate **Int1** and in the final 1,1-silaboration product **7** were also investigated.

All NBO analyses were carried out using NBO 6.0[S51] at the PBE0-D3(BJ)/def2-SVPD level of theory.

6.3.1. NBO analysis of 2·C_2H_4

In the **2**·C_2H_4 complex, both carbon atoms carry notable negative partial charges (−0.487 e and −0.429 e), indicating electron accumulation at the C=C bond. NBO analysis confirms predominant sp^2 hybridization at both C sites and a π-bond with >90% p-character, consistent with an intact double bond. The boron center interacts primarily with the π-system, forming a π-complex, as evidenced by minimal rehybridization and preservation of the C=C geometry—unlike the σ-complex observed in the **2**·C_6H_{10} analog. Selected donor–acceptor interactions from the NBO analysis are summarized in Table S17. Further details explaining the unusually high E(2) value for the π → B hyperconjugation interaction are provided in Section 6.3.2, where the bonding situation of the complex is further elucidated through Intrinsic Bond Orbital (IBO)[S52] analysis.

Table S17: Selected E(2) values for donor–acceptor interactions in **2**·C_2H_4 from NBO analysis. Level of theory: PBE0-D3(BJ)/def2-SVPD.

**donor**	**acceptor**	**E(2) [kcal/mol]**
σ B–Si	π* C=C	22.6
σ B–I	π* C=C	9.3
σ C=C	vacant sp^3 at B	23.1
π C=C	vacant sp^3 at B	313

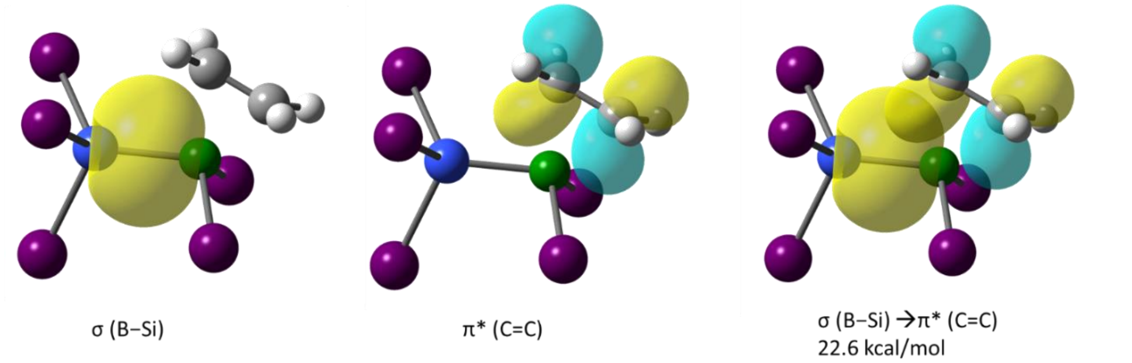

Figure S143: Donor (left; σ B–Si) and acceptor orbitals (middle; π* C=C), as well as the corresponding donor-acceptor interaction (right), derived from Natural Bond Orbital (NBO) analysis.

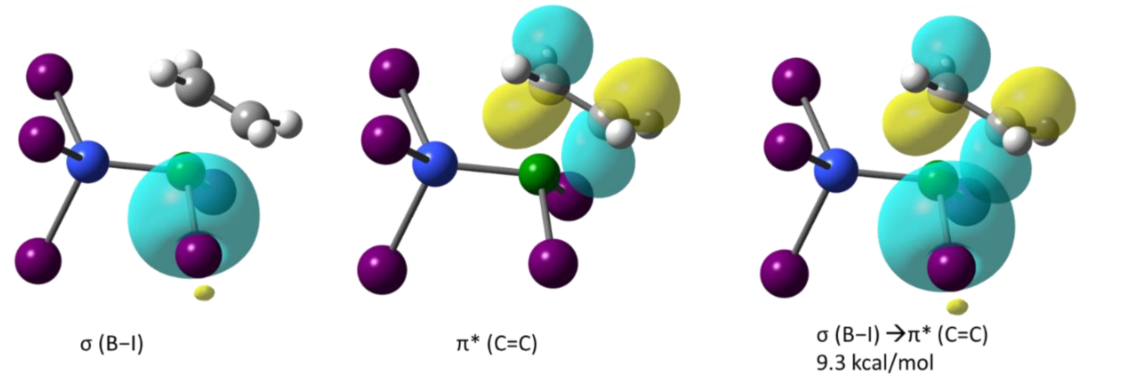

Figure S144: Donor (left; σ B–I) and acceptor orbitals (middle; π* C=C), as well as the corresponding donor-acceptor interaction (right), derived from Natural Bond Orbital (NBO) analysis.

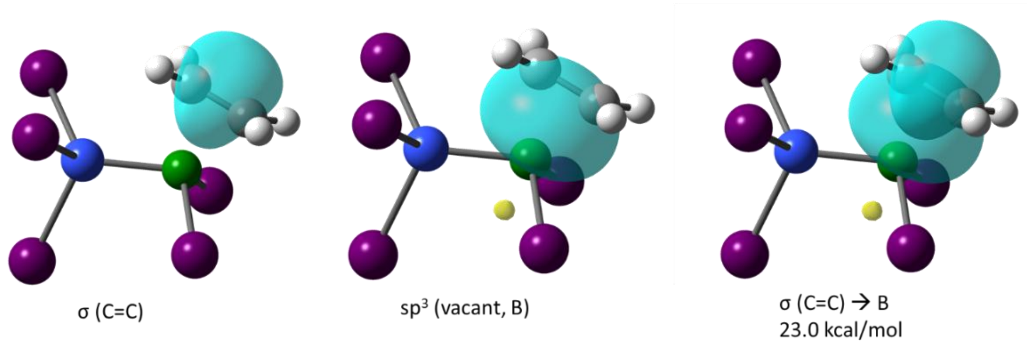

Figure S145: Donor (left; σ C=C) and acceptor orbitals (middle; vacant sp^3 at B), as well as the corresponding donor-acceptor interaction (right), derived from Natural Bond Orbital (NBO) analysis.

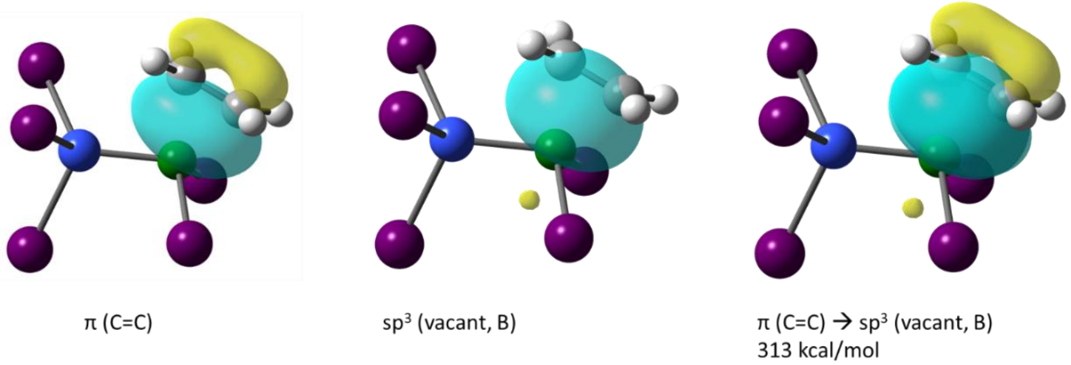

Figure S146: Donor (left; π C=C) and acceptor orbitals (middle; vacant sp^3 at B), as well as the corresponding donor-acceptor interaction (right), derived from Natural Bond Orbital (NBO) analysis.

6.3.2. IBO analysis of 2·C_2H_4

The bonding interaction between the boron atom and the π system of C_2H_4 in **2**·C_2H_4 was further examined through IBO calculations using IboView. The self-consistent field setup employed the program's default (PBE0-D3(BJ)/def2-TZVP), which has been shown to have minimal influence on the IBO localization procedure. Orbital localization was performed using the IBO method (exponent 2), with orbital division as the input wavefunction. The resulting IBO analysis indicates that the C=C (π) → B interaction is best described as formation of a two-electron–three-center (2e3c) bond (see Figure S147), with the bonding orbital being composed of 29.4% contribution from B, 35.4% from C(1), and 34.7% from C(2)—a slight deviation from the ideal equal sharing among the three centers. This description aligns well with the NBO results and the large E(2) value associated with the donor–acceptor interaction between the C=C π system and the vacant sp^3 orbital on B. Taken together, these findings strongly support the presence of a 2e3c bond in **2**·C_2H_4.

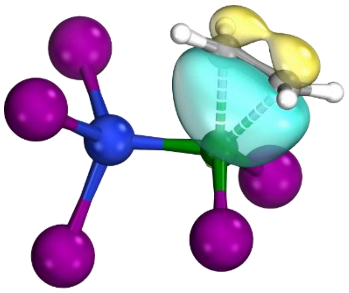

Figure S147: IBO representation of the C=C (π) → B interaction illustrating the formation of a two-electron–three-center (2e3c) bond. The bonding orbital comprises 29.4% contribution from B, 35.4% from C(1), and 34.7% from C(2).

6.3.3. NBO analysis of 2·C_6H_{10}

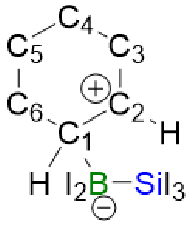

Figure S148: Numbering scheme for complex **2**·C_6H_{10}. For clarity, only the relevant hydrogen atoms on C(1) and C(2) are shown.

In the present complex, the α-carbon bonded to boron carries a partial negative charge of −0.503 e, while the adjacent β-carbon exhibits a positive charge of +0.089 e and possesses a vacant p_z orbital, indicative of carbocation character. The bond angles around the α-carbon (~348°) suggest a hybridization state intermediate between sp^2 and sp^3, with a tendency toward sp^3. NBO analysis reveals disruption of the original π-bond between the α- and β-carbons, consistent with σ-complex formation via direct boron coordination at the α-carbon. This bonding mode contrasts with that observed in the **2**·C_2H_4 complex, where coordination occurs through the π-system of the double bond rather than via a σ-bond. Selected donor–acceptor interactions from the NBO analysis are summarized in Table S18.

As expected, hyperconjugation from all axial C–H bonds adjacent to the carbocation exhibits significantly higher E(2) interaction energies compared to the equatorial C–H substituents.

Table S18: Selected E(2) values for donor–acceptor interactions in **2**·C_6H_{10} from NBO analysis. Level of theory: PBE0-D3(BJ)/def2-SVPD.

**Donor**	**acceptor**	**E(2) [kcal/mol]**
σ B–Si	vacant p at $C(\beta)^+$	4.7
σ C(1)–B^-(ax.)	vacant p at $C(\beta)^+$	92.7
σ C(1)–H(eq.)	vacant p at $C(\beta)^+$	6.2
σ C(3)–H(ax.)	vacant p at $C(\beta)^+$	19.5
σ C(3)–H(eq.)	vacant p at $C(\beta)^+$	5.9

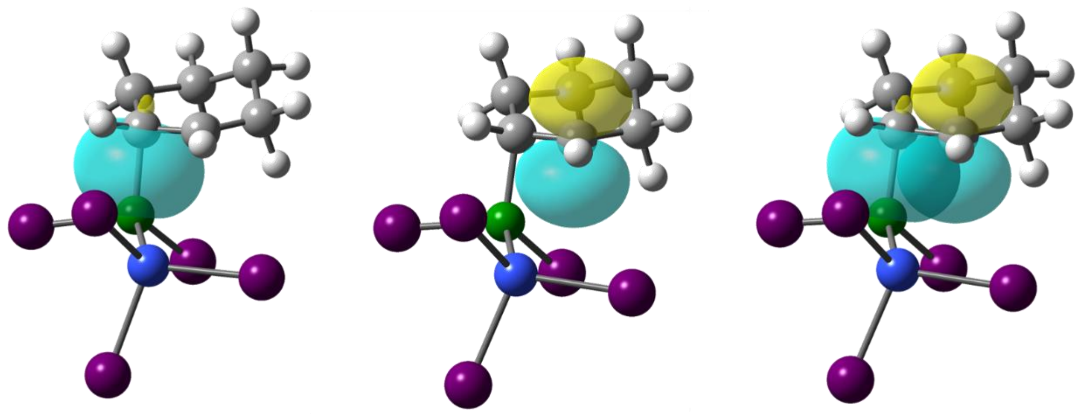

σ (C(1)–B^-) | p_z (vacant, C^+) | σ (C–B^-) → p_z (vacant, C^+) 92.7 kcal/mol

Figure S149: Donor (left; σ C(1)–B^-) and acceptor orbitals (middle; vacant p_z at C^+), as well as the corresponding donor-acceptor interaction (right), derived from Natural Bond Orbital (NBO) analysis.

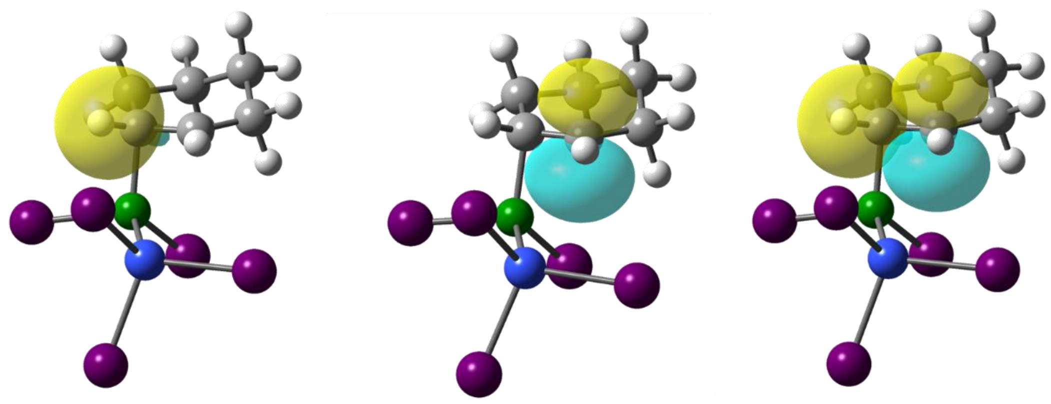

σ C(1)–H(eq.) | p_z (vacant, C^+) | σ (C–H) → p_z (vacant, C^+) 6.2 kcal/mol

Figure S150: Donor (left; σ C(1)–H(eq.)) and acceptor orbitals (middle; vacant p_z at C^+), as well as the corresponding donor-acceptor interaction (right), derived from Natural Bond Orbital (NBO) analysis.

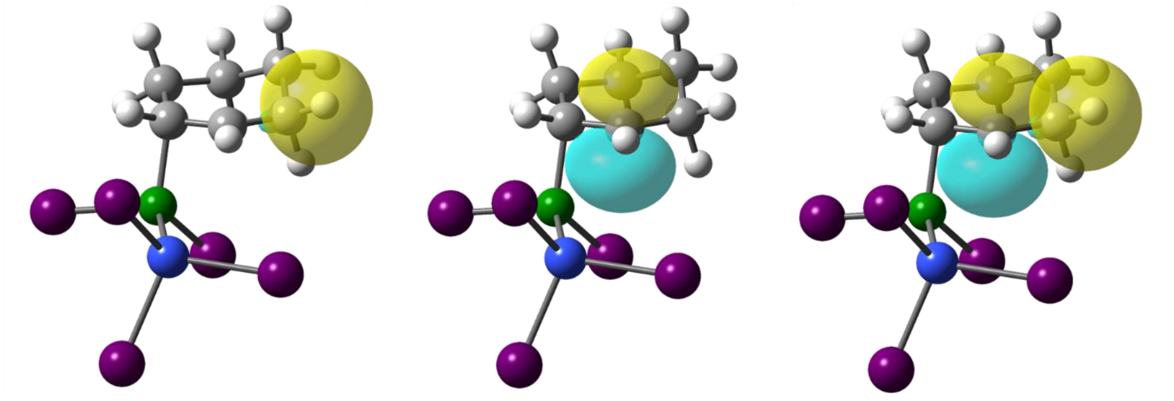

σ C(3)–H(eq.) | p_z (vacant, C^+) | σ (C–H) → p_z (vacant, C^+) 5.9 kcal/mol

Figure S151: Donor (left; σ C(3)–H(eq.)) and acceptor orbitals (middle; vacant p_z at C^+), as well as the corresponding donor-acceptor interaction (right), derived from Natural Bond Orbital (NBO) analysis.

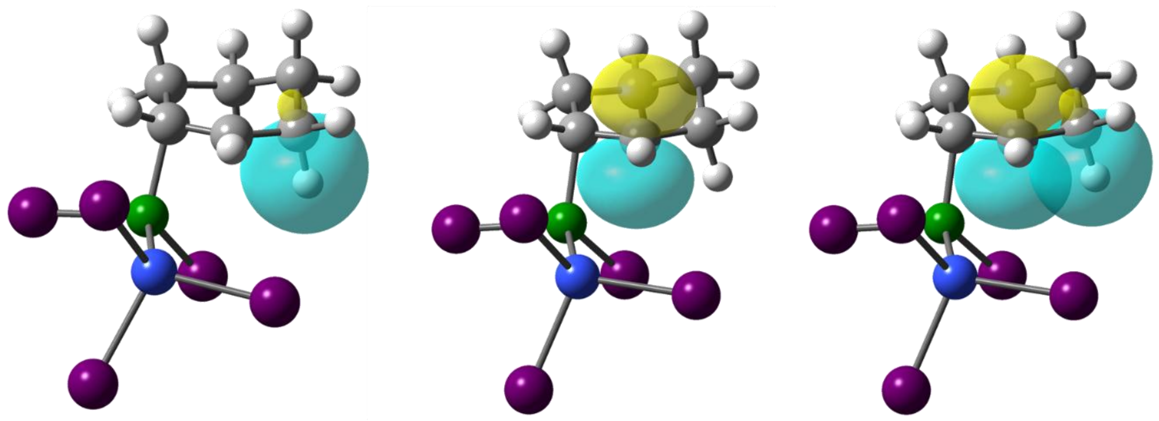

Figure S152: Donor (left; σ C(3)–H(ax.)) and acceptor orbitals (middle; vacant p_z at C^+), as well as the corresponding donor-acceptor interaction (right), derived from Natural Bond Orbital (NBO) analysis.

6.3.4. NBO analysis of Int1

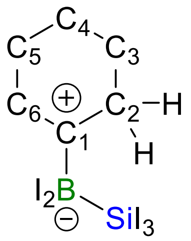

Figure S153: Numbering scheme for complex **Int1**. For clarity, only the relevant hydrogen atoms on C(2) are shown.

Int1 contains a carbocation bonded to a formally negatively charged boron atom. To assess the stabilization of this carbocation, NBO analysis was conducted. The most significant E(2) values are summarized in Table S19.

Table S19: Selected E(2) values for donor–acceptor interactions in **Int1** from NBO analysis. Level of theory: PBE0-D3(BJ)/def2-SVPD.

**donor**	**acceptor**	**E(2) [kcal/mol]**
σ B^-–Si	vacant p at $C(\alpha)^+$	41.4
σ C(2)–H(ax.)<br>σ C(6)–H(ax.)	vacant p at $C(\alpha)^+$	2 x 29.1
σ C(2)–H(eq.)<br>σ C(6)–H(eq.)	vacant p at $C(\alpha)^+$	2 x 3.8

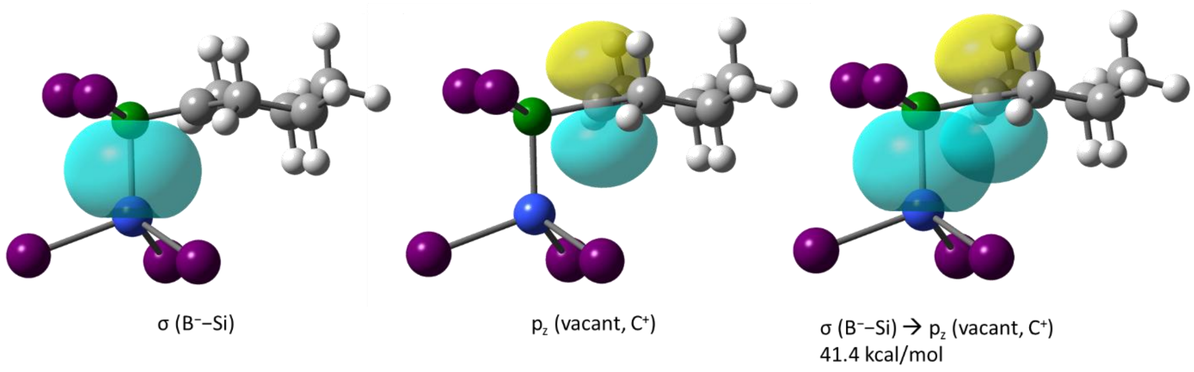

Figure S154: Donor (left; σ B⁻–Si) and acceptor orbitals (middle; vacant p_z at C⁺), as well as the corresponding donor-acceptor interaction (right), derived from Natural Bond Orbital (NBO) analysis.

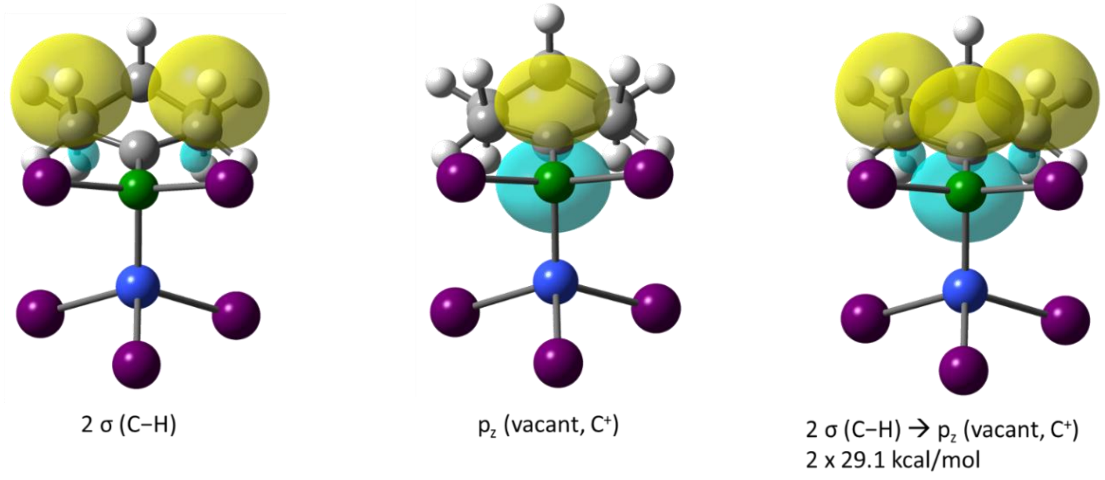

Figure S155: Donor (left; 2 x σ C–H) and acceptor orbitals (middle; vacant p_z at C⁺), as well as the corresponding donor-acceptor interaction (right), derived from Natural Bond Orbital (NBO) analysis.

6.3.5. NBO analysis of 7

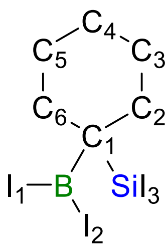

Figure S156: Numbering scheme for compound **7**.

The 1,1-silaboration product **7** features a formally vacant p_z orbital at the boron center. To understand why none of the silicon-bound iodine atoms engage in electron donation to this vacant orbital, via formation of a four-membered ring, we performed a NBO analysis of **7** to assess the electronic effects that stabilize the electron-deficient boron site. The associated most relevant E(2) values can be seen in Table S20.

Table S20: Selected E(2) values for donor–acceptor interactions in **7** from NBO analysis. Level of theory: PBE0-D3(BJ)/def2-SVPD.

**donor**	**acceptor**	**E(2) [kcal/mol]**
LP I(1)	vacant p_z at B	44.2
LP I(2)	vacant p_z at B	44.2
σ C(1)−Si	vacant p_z at B	16.0

6.3.6 NBO analysis of 3·C_6H_{10}

We propose, among other factors, steric effects and strong Lewis acidity as contributing reasons for the asymmetric formation of complex **2**·C_6H_{10} via σ-donation. To support this hypothesis, we calculated the adduct formation of **3** with C_6H_{10}, which is expected to represent an intermediate case between **2**·C_2H_4 and **2**·C_6H_{10}.

To determine appropriate methods for optimizing the structure of **3**·C_6H_{10} investigated herein, we performed geometry optimizations of **3**·SMe_2 considering the same five distinct DFT functionals used to benchmark **2**·SMe_2 including implicit solvation by the solvent model based on density (SMD; solvent = DCM; ε = 8.930). The Ahlrich's def2-SVP or def2-SVPD basis set was used in these calculations. The optimized results were compared to the respective X-ray crystal structure of **3**·SMe_2. A comparison of the root-mean-square deviation (RMSD) values, which were obtained excluding hydrogen atoms, are shown in Table S21. Overall, SMD(DCM)/M062X-D3/def2-SVP performed best among the tested theory levels. Optimized geometries were confirmed to be the desired minimum energy structures or transition states by vibrational frequency analysis.

Table S21: Root-mean-square deviations (RMSDs) between DFT-optimized geometries of **3**·SMe_2 and its experimentally determined solid-state structure using various functionals and basis sets.

**3**·SMe_2	B3LYP-D3(BJ)	BP86-D3(BJ)	M062X-D3	PBE0-D3(BJ)	ωB97X-D
def2-SVP	0.16119348	0.13452501	**0.06505766**	0.10465045	0.09936391
def2-SVPD	0.11786722	0.09766416	0.07845293	0.08181271	0.0792488

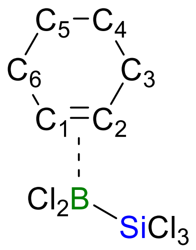

Figure S157: Numbering scheme for **3**·C_6H_{10}.

The NBO analysis of **3**·C_6H_{10} reveals that **3** is indeed coordinated primarily through a π → B interaction (E(2) = 130 kcal/mol). The torsion angle (C(2)–C(1)–B–Si) is nearly orthogonal at 83°, which can be attributed to the steric demand of the cyclohexene ligand. Since the Cl substituents in this complex occupy significantly less space compared to complex **2**·C_6H_{10}, a symmetric adduct formation seems to be favored (atom charges obtained from NBO calculations: C(1) = –0.25, C(2) = –0.14, and B = 0.02 e.).

Moreover, as no zwitterionic intermediate is formed in this case, the subsequent hydride shift—required for the 1,1-silaboration—is expected to be significantly less favorable. This may explain why such reactivity is not observed in reactions of **3** with C_6H_{10}.

Table S22: Selected E(2) values for donor–acceptor interactions in **3**·C_6H_{10} from NBO analysis. Level of theory: M062X-D3/def2-SVP.

donor	acceptor	E(2) [kcal/mol]
σ C(1)=C(2)	vacant p at B	9.5
π C(1)=C(2)	vacant p at B	130.2
σ C(1)−H	vacant p at B	12.3
σ C(2)−H	vacant p at B	6.1

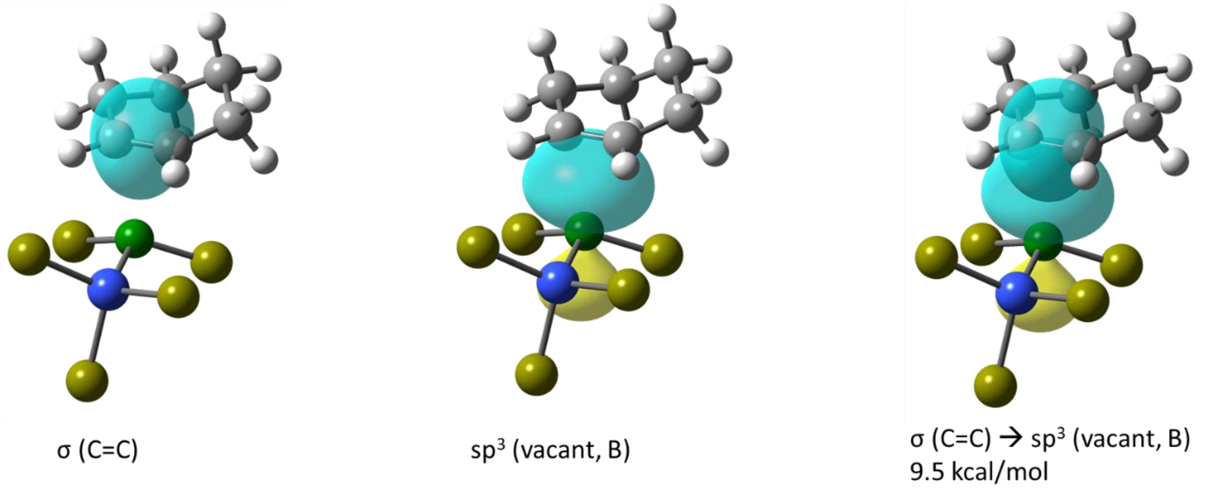

Figure S158: Donor (left; σ C=C) and acceptor orbitals (middle; vacant sp^3 at B), as well as the corresponding donor-acceptor interaction (right), derived from Natural Bond Orbital (NBO) analysis.

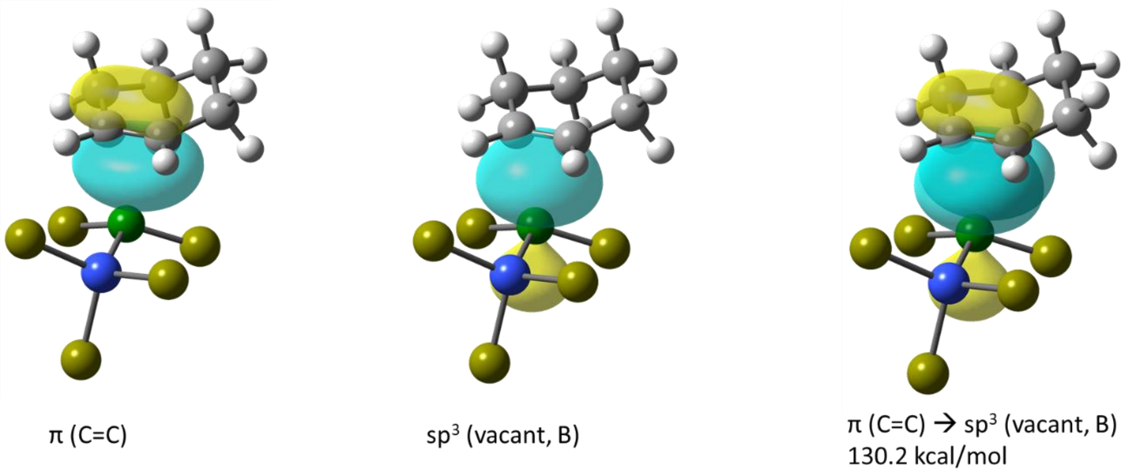

Figure S159: Donor (left; π C=C) and acceptor orbitals (middle; vacant sp^3 at B), as well as the corresponding donor-acceptor interaction (right), derived from Natural Bond Orbital (NBO) analysis.

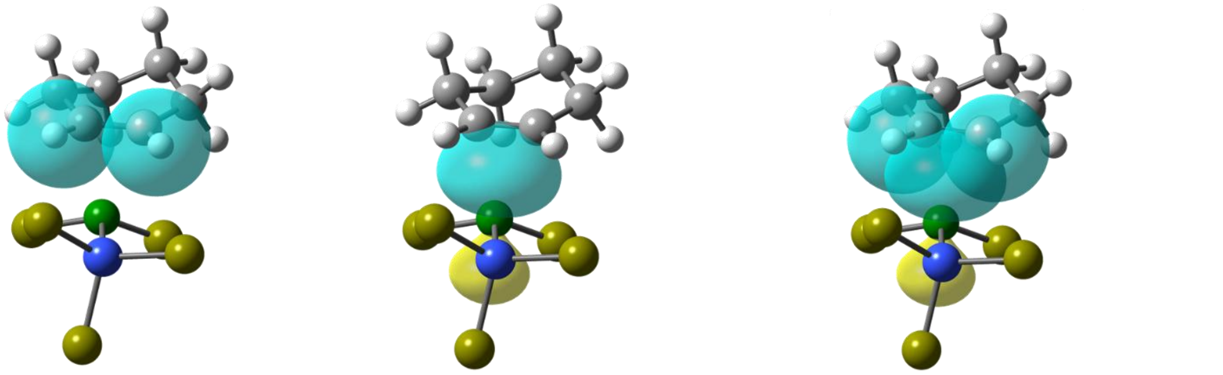

σ (C(1)−H)
σ (C(2)−H)

sp^3 (vacant, B)

2 σ (C−H) → sp^3 (vacant, B)
12.3 + 6.1 kcal/mol

Figure S160: Donor (left; 2 x σ C−H) and acceptor orbitals (middle; vacant sp^3 at B), as well as the corresponding donor-acceptor interaction (right), derived from Natural Bond Orbital (NBO) analysis.

6.4. Computed structures and corrected free energies

C_2H_4

G_{298} = -78,48742176 Hartree

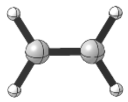

SMe_2

G_{298} = -477,7875158 Hartree

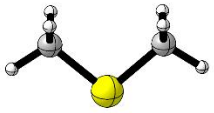

BI_3

G_{298} = -918,0982285 Hartree

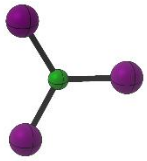

C_6H_{10}

G_{298} = -234,3428695 Hartree

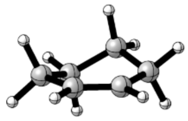

2·SMe_2

G_{298} = -2280,863974 Hartree

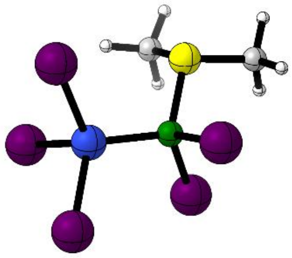

2

G_{298} = -1803,053269 Hartree

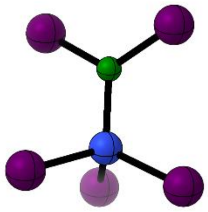

$BI_3 \cdot SMe_2$

$\boldsymbol{G}_{298}$ = -1395,901258 Hartree

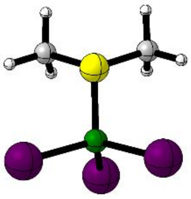

$2 \cdot C_2H_4$

$\boldsymbol{G}_{298}$ = -1881,532094 Hartree

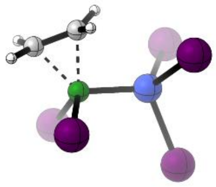

TS1

$\boldsymbol{G}_{298}$ = -1881,523294 Hartree

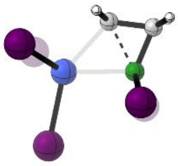

TS2

$\boldsymbol{G}_{298}$ = -1881,50817 Hartree

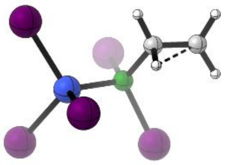

TS3

$\boldsymbol{G}_{298}$ = -1881,525428 Hartree

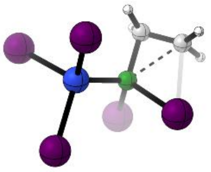

TS4

$\boldsymbol{G}_{298}$ = -2359,331596 Hartree

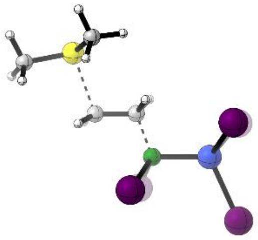

5

$\boldsymbol{G}_{298}$ = -1881,582877 Hartree

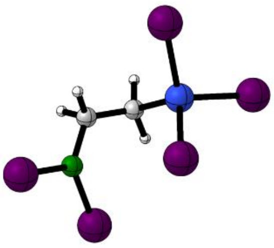

9

$\boldsymbol{G}_{298}$ = -2359,356775 Hartree

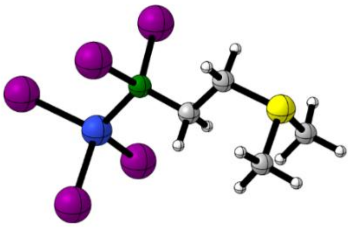

10

$\boldsymbol{G}_{298}$ = -1881,542543 Hartree

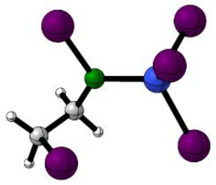

11

$\boldsymbol{G}_{298}$ = -1881,554476 Hartree

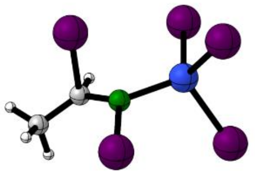

12

$\boldsymbol{G}_{298}$ = -1881,581978 Hartree

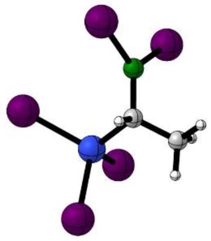

5·SMe₂

$\boldsymbol{G}_{298}$ = -2359,386088 Hartree

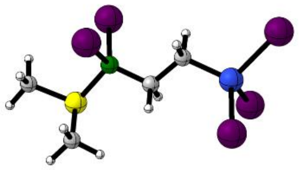

10·SMe₂

$\boldsymbol{G}_{298}$ = -2359,347714 Hartree

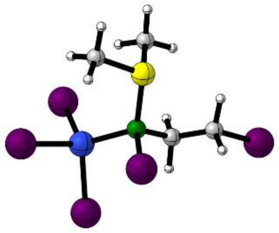

11·SMe₂

$\boldsymbol{G}_{298}$ = -2359,338927 Hartree

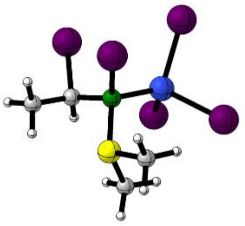

12·SMe₂

$\boldsymbol{G}_{298}$ = -2359,372011 Hartree

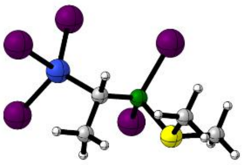

2·C₆H₁₀

$\boldsymbol{G}_{298}$ = -2037,3781 Hartree

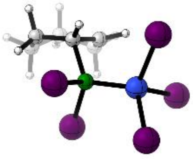

TS1’

$\boldsymbol{G}_{298}$ = -2037,352104 Hartree

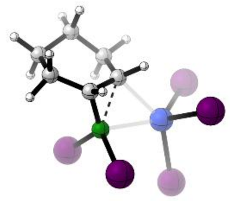

TS2’

$\boldsymbol{G}_{298}$ = -2037,37189 Hartree

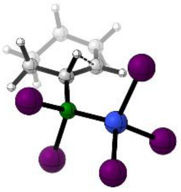

TS3’

$\boldsymbol{G}_{298}$ = -2037,383277 Hartree

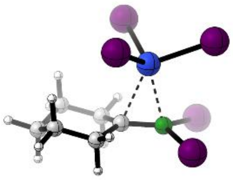

Int1

$\boldsymbol{G}_{298}$ = -2037,390634 Hartree

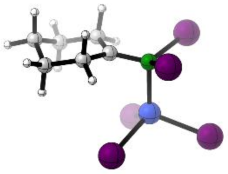

14

$\boldsymbol{G}_{298}$ = -2037,393632 Hartree

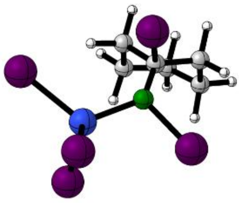

7

$\boldsymbol{G}_{298}$ = -2037,413415 Hartree

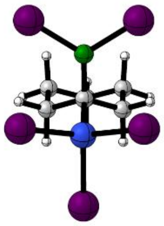

13

$\boldsymbol{G}_{298}$ = -2037,408047 Hartree

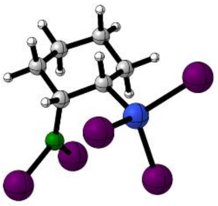

7. References

[S1] I. Krossing, "The Facile Preparation of Weakly Coordinating Anions: Structure and Characterisation of Silverpolyfluoroalkoxyaluminates $AgAl(OR_F)_4$, Calculation of the Alkoxide Ion Affinity" *Chem. Eur. J.* **2001**, *7*, 490–502.

[S2] G. R. Fulmer, A. J. M. Miller, N. H. Sherden, H. E. Gottlieb, A. Nudelman, B. M. Stoltz, J. E. Bercaw, K. I. Goldberg, "NMR Chemical Shifts of Trace Impurities: Common Laboratory Solvents, Organics, and Gases in Deuterated Solvents Relevant to the Organometallic Chemist" *Organometallics* **2010**, *29*, 2176–2179.

[S3] S. Hayashi, K. Hayamizu, "Shift References in High-Resolution Solid-State NMR" *Bull. Chem. Soc. Jpn.* **1989**, *62*, 2429–2430.

[S4] S. Hayashi, K. Hayamizu, "Chemical Shift Standards in High-Resolution Solid-State NMR (1) ^{13}C, ^{29}Si, and ^{1}H Nuclei" *Bull. Chem. Soc. Jpn.* **1991**, *64*, 685–687.

[S5] A. E. Aliev, K. D. M. Harris, D. C. Apperley, "High-resolution Solid-state ^{13}C and ^{29}Si NMR Investigations of the Dynamic Properties of Tetrakis(trimethylsilyl)silane" *Chem. Commun.* **1993**, 251–253.

[S6] T. H. J. Niedermeyer, M. Strohalm, "mMass as a Software Tool for the Annotation of Cyclic Peptide Tandem Mass Spectra" *PLoS One* **2012**, *7*, e44913.

[S7] M. Strohalm, M. Hassman, B. Košata, M. Kodíček, "*mMass* data miner: an open source alternative for mass spectrometric data analysis" *Rapid Commun. Mass Spectrom.* **2008**, *22*, 905–908.

[S8] M. Strohalm, D. Kavan, P. Novák, M. Volný, V. Havlíček, "*mMass* 3: A Cross-Platform Software Environment for Precise Analysis of Mass Spectrometric Data" *Anal. Chem.* **2010**, *82*, 4648–4651.

[S9] A. G. Briggs, "The Preparation of Very Pure Boron Iodide" *Naturwissenschaften* **1990**, *77*, 595–597.

[S10] J. Teichmann, M. Bursch, B. Köstler, M. Bolte, H. W. Lerner, S. Grimme, M. Wagner, "Trapping Experiments on a Trichlorosilanide Anion: a Key Intermediate of Halogenosilane Chemistry" *Inorg. Chem.* **2017**, *56*, 8683–8688.

[S11] H. Nöth, B. Wrackmeyer, in *Nuclear Magnetic Resonance Spectroscopy of Boron Compounds* (Eds.: P. Diehl, E. Fluck, R. Kosfeld), Springer, Berlin, **1978**, p. 125.

[S12] N. Sen, N. Parvin, S. Tothadi, S. Khan, "Reactivity of $(TMS)_2N(\eta^1\text{-}Cp^*)Si{=}Si(\eta^1\text{-}Cp^*)N(TMS)_2$ toward the Halides of Groups 13–15" *Organometallics* **2021**, *40*, 1874–1883.

[S13] S. Muthaiah, D. C. H. Do, R. Ganguly, D. Vidović, "Counterion Dependence on the Synthetic Viability of NHC-stabilized Dichloroborenium cations" *Organometallics* **2013**, *32*, 6718–6724.

[S14] T. J. Barton, N. Tillman, "Mechanism of the Decomposition of Silacyclobutane to Silylene and Propene" *J. Am. Chem. Soc* **1987**, *109*, 6711–6716.

[S15] S. H. Kang, J. S. Han, M. E. Lee, B. R. Yoo, I. N. Jung, "Phosphonium Chloride Induced Dichlorosilylene Transfer from Trichlorosilane" *Organometallics* **2003**, *22*, 2551–2553.

[S16] S. H. Hong, S. I. Hyun, I. N. Jung, W. S. Han, M. H. Kim, H. Yun, S. W. Nam, S. O. Kang, "Phosphine-Catalyzed Si–C Coupling of Bissilylmethanes: Preparation of Cyclic $(Cl_2SiCH_2)_2$ and Linear $Cl_2Si(CH_2SiCl_3)_2$ via Silylene and Silene Intermediates" *Organometallics* **2010**, *29*, 687–691.

[S17] H. Nöth, B. Wrackmeyer, in *Nuclear Magnetic Resonance Spectroscopy of Boron Compounds* (Eds.: P. Diehl, E. Fluck, R. Kosfeld), Springer, Berlin, **1978**, p. 372.

[S18] P. N. Gates, E. J. Mclauchlan, E. F. Mooney, "N.M.R. studies of donor-acceptor interaction in boron trihalide complexes–I. The use of boron-11 chemical shifts as a measure of donor-acceptor interaction" *Spectrochim. Acta* **1965**, *21*, 1445–1448.

[S19] E. Muylle, G. P. Van Der Kelen, E. G. Claeys, "NMR study of donor-acceptor complexes–IV. ^{11}B NMR of arylphosphine and-arsine boronhalide complexes" *Spectrochim. Acta A* **1976**, *32*, 1149–1154.

[S20] T. R. Durkin, E. P. Schram, "Reactions of Tris(triphenylphosphine)platinum(0). II. Preparation and Properties of Boron Trichloride and Aluminum Trimethyl Adducts of Platinum(0)" *Inorg. Chem.* **1972**, *11*, 1054–1059.

[S21] A. G. Briggs, R. Piercy, "The ultraviolet absorption spectrum of boron monoiodide (BI)" *Spectrochim. Acta A* **1973**, *29*, 851–853.

[S22] N. Wiberg, A. Holleman, *Lehrbuch Der Anorganischen Chemie,* De Gruyter, Berlin, **2007**.

[S23] M. A. Dureen, C. C. Brown, D. W. Stephan, "Deprotonation and Addition Reactions of Frustrated Lewis Pairs with Alkynes" *Organometallics* **2010**, *29*, 6594–6607.

[S24] S. Fliszar, G. Cardinal, "Charge distributions and chemical effects. XXXV. Polyunsaturated hydrocarbons, energy calculations from C-13 nmr spectra" *Can. J. Chem.* **1984**, *62*, 2748–2754.

[S25] M. Stöcker, M. Klessinger, K. Wilhelm, "13C,13C Coupling Constants of Conjugated Dienes" *Org. Magn. Reson.* **1981**, *17*, 153–155.

[S26] G. M. Sheldrick, "A short history of *SHELX*" *Acta Crystallogr. Sect. A* **2008**, *64*, 112–122.

[S27] G. M. Sheldrick, "*SHELXT* – Integrated space-group and crystal-structure determination" *Acta Crystallogr. Sect. A* **2015**, *71*, 3–8.

[S28] G. M. Sheldrick, "Crystal structure refinement with *SHELXL*" *Acta Crystallogr. Sect. C* **2015**, *71*, 3–8.

[S29] S. I. Ivlev, M. Conrad, F. Kraus, "HKLF5Tools: a program for processing diffraction data of non-merohedrally twinned crystals" *Z. Kristallogr. - Cryst. Mater.* **2019**, *234*, 415–418.

[S30] *WinX*^{POW}; Stoe & Cie.: Darmstadt, Germany, **2011**.

[S31] M. J. Frisch, G. W. Trucks, H. B. Schlegel, G. E. Scuseria, M. A. Robb, J. R. Cheeseman, G. Scalmani, V. Barone, G. A. Petersson, H. Nakatsuji, X. Li, M. Caricato, A. V Marenich, J. Bloino, B. G. Janesko, R. Gomperts, B. Mennucci, H. P. Hratchian, J. V Ortiz, D. J. Fox, Gaussian 16, Revision B.01, **2016**.

[S32] C. Y. Legault, CYLview, 1.0b, **2009**.

[S33] R. Dennington, T. Keith, J. Millam, Semichem Inc., Shawnee Mission, GaussView, Version 6.1.1, **2019**.

[S34] A. V. Marenich, C. J. Cramer, D. G. Truhlar, "Universal Solvation Model Based on Solute Electron Density and on a Continuum Model of the Solvent Defined by the Bulk Dielectric Constant and Atomic Surface Tensions" *J. Phys. Chem. B* **2009**, *113*, 6378–6396.

[S35] C. Lee, W. Yang, R. G. Parr, "Development of the Colic-Salvetti correlation-energy formula into a functional of the electron density" *Phys. Rev. B* **1988**, *37*, 785–789.

[S36] A. D. Becke, "Density-functional thermochemistry. III. The role of exact exchange" *J. Chem. Phys.* **1993**, *98*, 5648–5652.

[S37] S. Grimme, J. Antony, S. Ehrlich, H. Krieg, "A consistent and accurate *ab initio* parametrization of density functional dispersion correction (DFT-D) for the 94 elements H-Pu" *J. Chem. Phys.* **2010**, *132*, 154104.

[S38] S. Grimme, S. Ehrlich, L. Goerigk, "Effect of the Damping Function in Dispersion Corrected Density Functional Theory" *J. Comput. Chem.* **2011**, *32*, 1456–1465.

[S39] J. P. Perdew, "Density-functional approximation for the correlation energy of the inhomogeneous electron gas" *Phys. Rev. B* **1986**, *33*, 8822–8824.

[S40] A. D. Becke, "Density-functional exchange-energy approximation with correct asymptotic behavior" *Phys. Rev. A* **1988**, *38*, 3098–3100.

[S41] Y. Zhao, D. G. Truhlar, "The M06 suite of density functionals for main group thermochemistry, thermochemical kinetics, noncovalent interactions, excited states, and transition elements: Two new functionals and systematic testing of four M06-class functionals and 12 other functionals" *Theor Chem Account* **2008**, *120*, 215–241.

[S42] J. P. Perdew, K. Burke, M. Ernzerhof, "Generalized Gradient Approximation Made Simple" *Phys. Rev. Lett.* **1997**, *78*, 1396.

[S43] C. Adamo, V. Barone, "Toward reliable density functional methods without adjustable parameters: The PBE0 model" *J. Chem. Phys.* **1999**, *110*, 6158–6170.

[S44] J. Da Chai, M. Head-Gordon, "Long-range corrected hybrid density functionals with damped atom–atom dispersion corrections" *Phys. Chem. Chem. Phys.* **2008**, *10*, 6615–6620.

[S45] F. Weigend, R. Ahlrichs, "Balanced basis sets of split valence, triple zeta valence and quadruple zeta valence quality for H to Rn: Design and assessment of accuracy" *Phys. Chem. Chem. Phys.* **2005**, *7*, 3297–3305.

[S46] B. P. Pritchard, D. Altarawy, B. Didier, T. D. Gibson, T. L. Windus, "New Basis Set Exchange: An Open, Up-to-Date Resource for the Molecular Sciences Community" *J. Chem. Inf. Model.* **2019**, *59*, 4814–4820.

[S47] A. M. Walsh, R. F. Loring, "Time resolved stimulated light scattering from a solvated chromophore: A molecular dynamics study" *J. Chem. Phys.* **1991**, *94*, 7575–7587.

[S48] C. P. Kelly, C. J. Cramer, D. G. Truhlar, "SM6: A Density Functional Theory Continuum Solvation Model for Calculating Aqueous Solvation Free Energies of Neutrals, Ions, and Solute–Water Clusters" *J. Chem. Theory Comput.* **2005**, *1*, 1133–1152.

[S49] A. Stirling, A. Hamza, T. A. Rokob, I. Pápai, "Concerted attack of frustrated Lewis acid-base pairs on olefinic double bonds: A theoretical study" *Chem. Commun.* **2008**, 3148–3150.

[S50] F. Weinhold, C. R. Landis, E. D. Glendening, "What is NBO analysis and how is it useful?" *Int. Rev. Phys. Chem.* **2016**, *35*, 399–440.

[S51] E. D. Glendening, C. R. Landis, F. Weinhold, "NBO 6.0: Natural Bond Orbital Analysis Program" *J. Comput. Chem.* **2013**, *34*, 1429–1437.

[S52] G. Knizia, "Intrinsic Atomic Orbitals: An Unbiased Bridge Between Quantum Theory and Chemical Concepts" *J. Chem. Theory Comput.* **2013**, *9*, 4834–4843.

Computed_structures.xyz
This file cannot be rendered in this PDF. Please download the source file.
